# Distinct CD16a features on human NK cells observed by flow cytometry correlate with increased ADCC

**DOI:** 10.1038/s41598-024-58541-6

**Published:** 2024-04-04

**Authors:** Maria C. Rodriguez Benavente, Zainab A. Hakeem, Alexander R. Davis, Nathan B. Murray, Parastoo Azadi, Emily M. Mace, Adam W. Barb

**Affiliations:** 1 Department of Biochemistry and Molecular Biology, University of Georgia, 120 E. Green St., 30602 Athens, GA Georgia; 2 Complex Carbohydrate Research Center, University of Georgia, Athens, GA Georgia; 3 Department of Pediatrics, Columbia University Irving Medical Center, New York, NY USA; 4 Department of Chemistry, University of Georgia, Athens, GA Georgia

**Keywords:** *N*-glycosylation, Natural killer cell, ADCC, Fc γ receptor IIIa, CD16a, Lymphocyte activation, Antibodies, Glycobiology

## Abstract

Natural killer (NK) cells destroy tissue that have been opsonized with antibodies. Strategies to generate or identify cells with increased potency are expected to enhance NK cell-based immunotherapies. We previously generated NK cells with increased antibody-dependent cell mediated cytotoxicity (ADCC) following treatment with kifunensine, an inhibitor targeting mannosidases early in the *N*-glycan processing pathway. Kifunensine treatment also increased the antibody-binding affinity of Fc γ receptor IIIa/CD16a. Here we demonstrate that inhibiting NK cell *N*-glycan processing increased ADCC. We reduced *N*-glycan processing with the CRIPSR-CAS9 knockdown of MGAT1, another early-stage *N*-glycan processing enzyme, and showed that these cells likewise increased antibody binding affinity and ADCC. These experiments led to the observation that NK cells with diminished *N*-glycan processing capability also revealed a clear phenotype in flow cytometry experiments using the B73.1 and 3G8 antibodies binding two distinct CD16a epitopes. We evaluated this “affinity profiling” approach using primary NK cells and identified a distinct shift and differentiated populations by flow cytometry that correlated with increased ADCC.

## Introduction

Natural killer cells are early responders that destroy virally infected cells and malignant tissue^1^. Accordingly, the occurrence of genetic polymorphisms that impaired NK cell gene expression correlated with poorer prognosis in the treatment of solid tumors^2^. Low NK cell activity also correlated with increased cancer risk^3^ and increased risk of recurrence^4,5^. Improving the NK cell response, either through enhancing NK cell responses or identifying features of NK cells with superior cytotoxic potential, is widely expected to improve treatment efficacy and durability.

Engineered NK cells are being explored to complement the body’s natural response in part because contemporary treatment regimens impair NK cell responses^6^. As of this writing, we are aware of four current clinical trials involving “high-affinity” NK (haNK) cells paired with a therapeutic monoclonal antibody (mAb) to improve antibody-dependent cell-mediated cytotoxicity (ADCC) and target cell destruction (Refs.^7,8^ and clinicaltrials.gov). Both these engineered NK cells and endogenous NK cells are activated by antibody-mediated clustering of Fc γ Receptor IIIa (FcγRIIIa/CD16a). CD16a and ADCC are important components of treatments with many mAbs, including rituximab and trastuzumab^9,10^.

The affinity of Fc-mediated antibody binding to CD16a affects both ADCC potency and therapeutic efficacy. The haNK cells utilize the tighter binding CD16a V158 variant. NK cells expressing the F158 variant exhibit lower responses to mAbs^11–13^. Antibody engineering to increase the affinity likewise increases cell response^14^. Our laboratory identified a new strategy to increase antibody-binding affinity. The composition of carbohydrate moieties attached to the CD16a asparagine residues (*N*-glycans) affected affinity, with minimally remodeled *N*-glycans termed “oligomannose” types providing higher antibody-binding affinity^15,16^ (for a review of protein *N*-glycosylation, see^17^). Notably, both lower-affinity highly remodeled “complex-type” glycans and high affinity oligomannose-type *N*-glycans appeared at the key CD16a N162 glycosylation site on NK cells isolated from healthy donors^18^. Furthermore, the level of high and low affinity CD16a forms differed dramatically among different donors.

NK cell glycan processing likewise affects both antibody binding at the cell surface and effector function. YTS-CD16a NK cells, treated with kifunensine, exhibited both increased CD16a affinity and ADCC potency^19^. YTS cells are derived from the YT cell line isolated from a 15 year old patient; YTS-CD16a cells are engineered to express the CD16a V158 allotype^20,21^. The ADCC increases were observed using either rituximab with Raji B cells or trastuzumab with SK-BR3 cells. Primary human NK cells also demonstrated increased ADCC upon treatment with kifunensine that blocks a specific mannose trimming step and produces primarily oligomannose-type *N*-glycans^19^. It is possible that an off-target kifunensine effect, rather than *N*-glycan biosynthesis, is responsible for increased NK cell ADCC. Here we evaluate this possibility by creating an NK clone with reduced *N*-glycan processing.

The variability of CD16a processing by NK cells, revealing an abundance of high affinity glycoforms for some donors, suggests that ADCC activation is regulated by *N*-glycan processing in the NK cell. Approaches to identify higher activity forms provides an ability to enrich these forms to evaluate in a treatment regimen, particularly where NK cell activity is reduced. The prior studies utilized rigorous cell isolation, protein purification and mass-spectrometry (MS)-based glycopeptide analysis to identify compositional variability. These methods, though sensitive, and not practical for screening NK cell variability in large populations due to the requirement of high numbers of cells (> 2 × 10^7^; Ref.^15^). Furthermore, MS-based methods do not directly characterize a functional parameter like the antibody-binding affinity of CD16a. Based on published studies, we anticipate that NK cells exhibit variability of antibody-binding affinity by CD16a, and this affinity affects ADCC potency. Here we demonstrate a method to distinguish NK cells with different antibody-binding affinities using flow cytometry, and show that NK cell CD16a affinity changes correlate with changes in NK cell ADCC.

## Results

### Polyclonal IgG decreases the ADCC of NK cells treated with kifunensine

**Figure 1:**
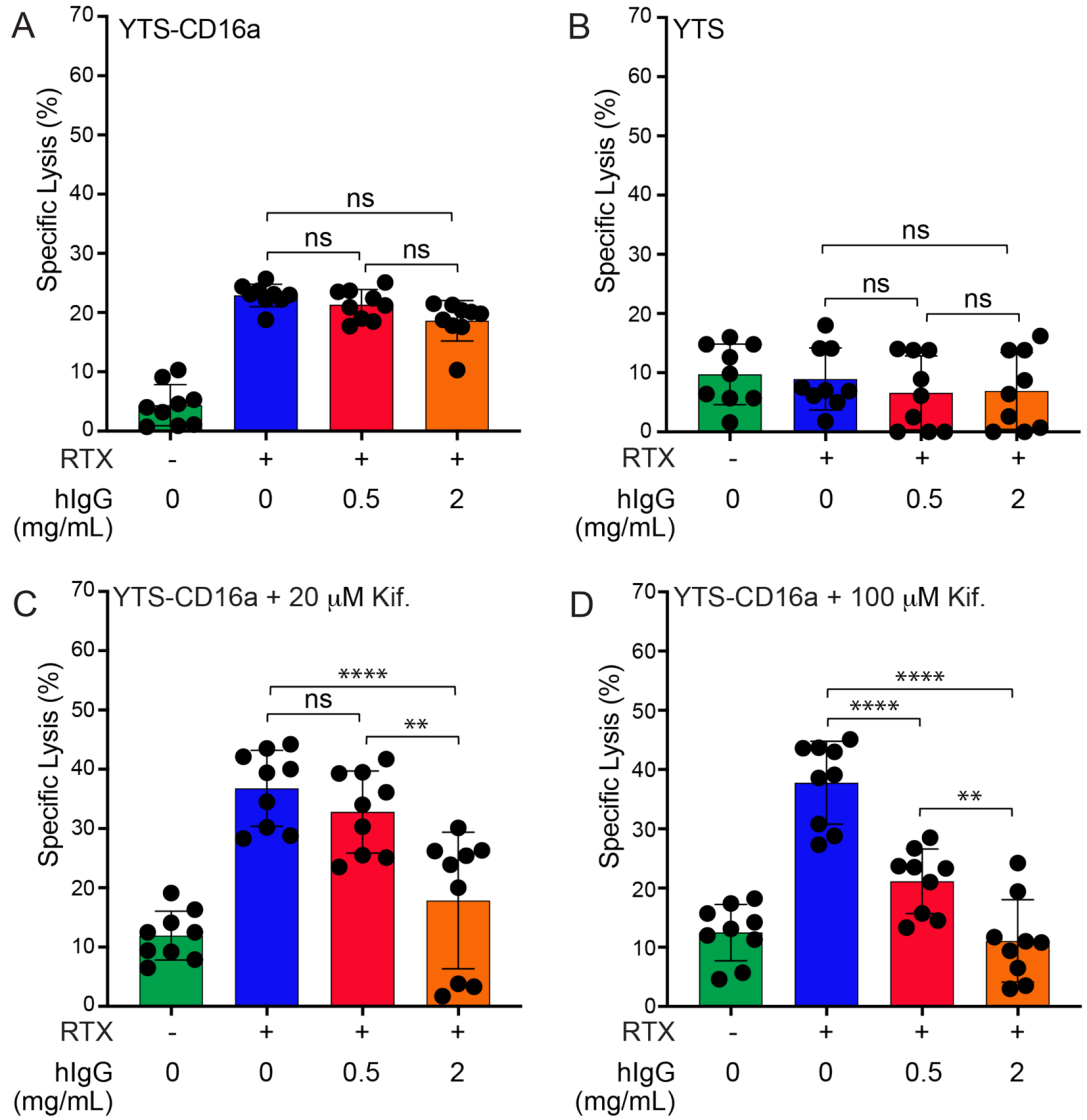
Polyclonal IgG reduced the ADCC (by TR-F) of kifunensine-treated YTS-CD16a cells. (**A**) YTS-CD16a cells show a slight trend in ADCC decrease only upon IgG blocking at high concentration (2 mg/mL), (**B**) YTS cells show no ADCC or IgG blocking due to lack of CD16a receptor, (**C**) Kifunensine-treated (Kif., 20 µM) YTS-CD16a cells show significant decrease in ADCC with IgG blocking at high concentration (2 mg/mL), (**D**) Kifunensine-treated (Kif., 100 µM) YTS-CD16a cells show significant decrease in ADCC with IgG blocking at both low and high concentrations (0.5 and 2 mg/mL). For all panels, data points represent the mean for three independent experiments collected on three different days at 20:1 E:T ratio, each with three replicates, ± SD by one-way ANOVA. ns, not significant; RTX, rituximab (20 µg/mL); **p < 0.01; ****p < 0.0001.

We previously reported that NK cells with minimally processed oligomannose-type *N*-glycans exhibited increased affinity for IgG1 Fc, and increased antibody-dependent cell-mediated cytotoxicity (ADCC)^15,16,19^. These assays, like many ADCC assays, were performed in a buffered medium that recapitulates the salinity and pH of the blood, but notably lacks serum proteins including polyclonal IgG (hereafter referred to as “blocking” IgG). We examined the impact of blocking IgG on the ADCC of YTS-CD16a cells to determine if serum IgG affects ADCC potency in this cell-based assay. We observed a slight, non-significant decrease in ADCC in the presence of 0.5 and 2 mg/mL blocking IgG (Fig. 1a, Fig. S1a). As previously reported, YTS cells which do not express CD16a receptor showed no ADCC with rituximab (RTX)^19^, nor IgG blocking following kifunensine treatment (p > 0.9999) (Fig. 1b, Fig. S1b).

**Figure 2:**
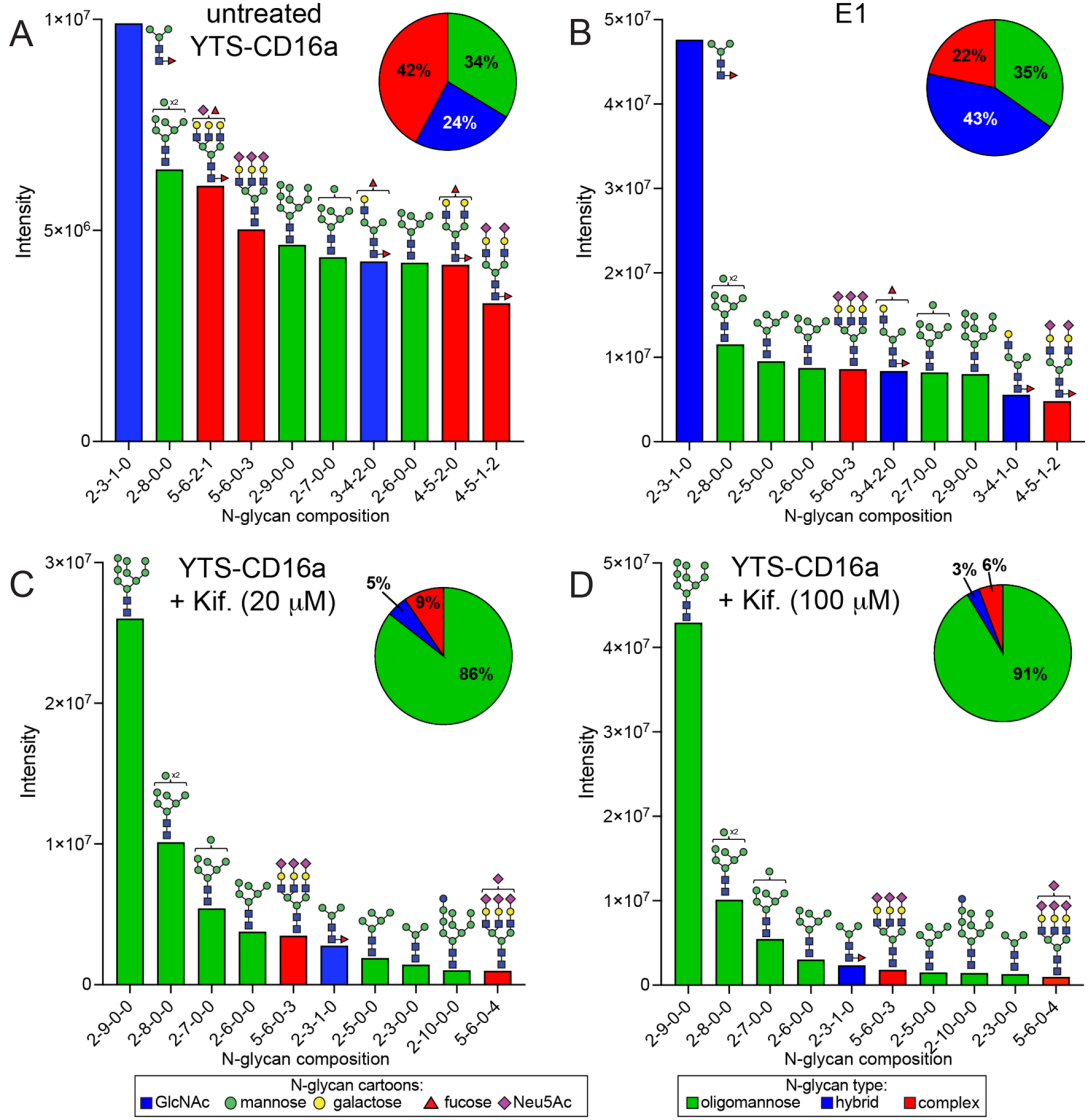
Minimally-processed *N*-glycans are found on NK cells with greater ADCC. Relative intensity of the ten most abundant *N*-glycans identified from YTS-CD16a cells using NSI-MS/MS. (**A**) Wild-type cells, (**B**) the MGAT1 knockdown clone E1, (**C**) cells treated with kifunensine (Kif., 20 µM), (**D**) cells treated with kifunensine (Kif., 100 µM). Pie charts represent the distribution of all *N*-glycan types identified. Cartoons indicate one possible glycan configuration consistent with the composition, isobaric species were not distinguished. Monosaccharide residues in the cartoons are defined by the CFG convention. The code for the glycan composition on the x-axes reports the numbers of the following residues as identified by MS (HexNAc-hexose-deoxyhexose-Neu5Ac).

We next evaluated the impact of increased IgG-binding affinity on the cell surface by treating YTS-CD16a cells with kifunensine. ADCC was significantly reduced at the high concentration of blocking IgG (2 mg/mL; p = 0.0087) at all target-to-effector (T:E) ratios used in this study for the 20 μM kifunensine treatment (Fig. 1c, Fig. S1c). The 100 μM kifunensine treatment similarly reduced ADCC at the higher blocking IgG concentration (2 mg/mL; p < 0.0001), but also with the lower concentration of blocking IgG (0.5 mg/mL; p = 0.0004; Fig. 1d, Fig. S1d). We confirmed the impact of kifunensine treatment on *N*-glycan processing using LC-NSI-MS/MS and MALDI-MS and found the increased abundance of minimally processed oligomannose *N*-glycans on the NK cell surface (Fig. 2, Figs. S2, S7).

We previously reported that two treatments that increase the antibody affinity for the NK cell surface, namely removing antibody fucosylation and restricting NK cell *N*-glycan processing, additively increased ADCC^19^. We next examined whether the impact of blocking IgG similarly reduced the ADCC of kifunensine-treated NK cells when using an afucosylated antibody (RTX-fuc). As expected, untreated YTS-CD16a cells showed decreased ADCC, albeit not significant (from 20.91 to 17.84%, p = 0.5926) with 2 mg/mL blocking IgG present (Fig. S3). YTS-CD16a cells treated with kifunensine, however, showed a significant ADCC decrease with RTX-fuc and blocking IgG (from 39.92 to 13.86%, p < 0.0001). Taken together, these data suggest that increased affinity of CD16a after kifunensine treatment enhances the blocking effect of IgG.

**Figure 3:**
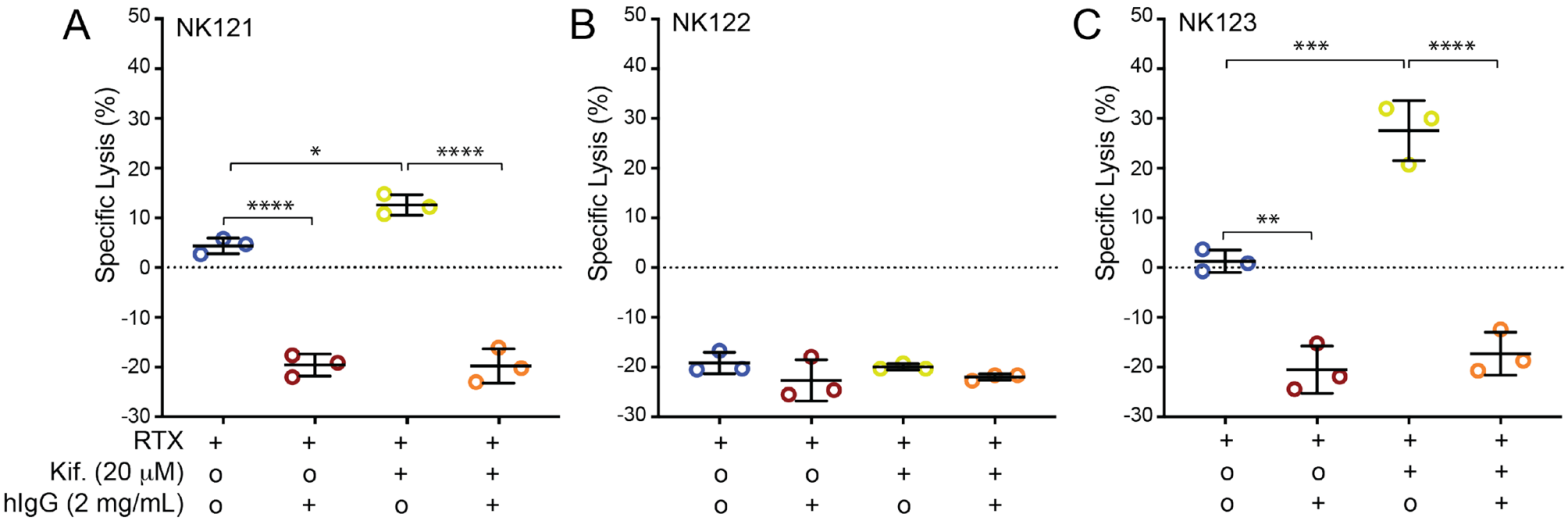
IgG blocks ADCC (by TR-F) of primary NK cells. (**A-C**) IgG blocking (2 mg/mL) of ADCC following treatment with kifunensine (Kif., 20 µM) for primary NK cells isolated from three donors at 20:1 E:T ratio. Means are indicated with a horizontal black line ± SD. RTX, rituximab (20 µg/mL). For all panels, one-way ANOVA; *p < 0.05; **p < 0.01; ***p < 0.001; ****p < 0.0001.

To determine if the observed sensitivity to blocking IgG decreased the effector function of primary human NK cells, we isolated NK cells from three healthy donors. Kifunensine treatment enhanced ADCC of the primary NK cells from two donors in the absence of blocking IgG. NK cells from NK121 and NK123 showed significant ADCC reduction (minimum of p = 0.0017) upon incubation with 2 mg/mL blocking IgG following kifunensine treatment (Fig. 3). The ADCC value becomes negative as a result of subtracting the target cell killing in the absence of antibody; in contrast to fresh primary cells these NK cells are cultured for 3 days in a medium lacking human serum and human antibodies which are presumed to block NK cell activity. Donor NK122 showed no increased specific lysis in the presence of the rituximab antibody and thus no sensitivity to blocking IgG upon kifunensine treatment. It is common to find variability in the effector function of primary NK cells from different donors^22^. It is worth mentioning that the NK cell donors used for this particular experiment came from one specific age range of the population, specifically 31–32 year old white males so it is possible that donors with different ages or genders may respond differently.

### MGAT1 knockdown introduces sensitivity to blocking IgG

**Figure 4:**
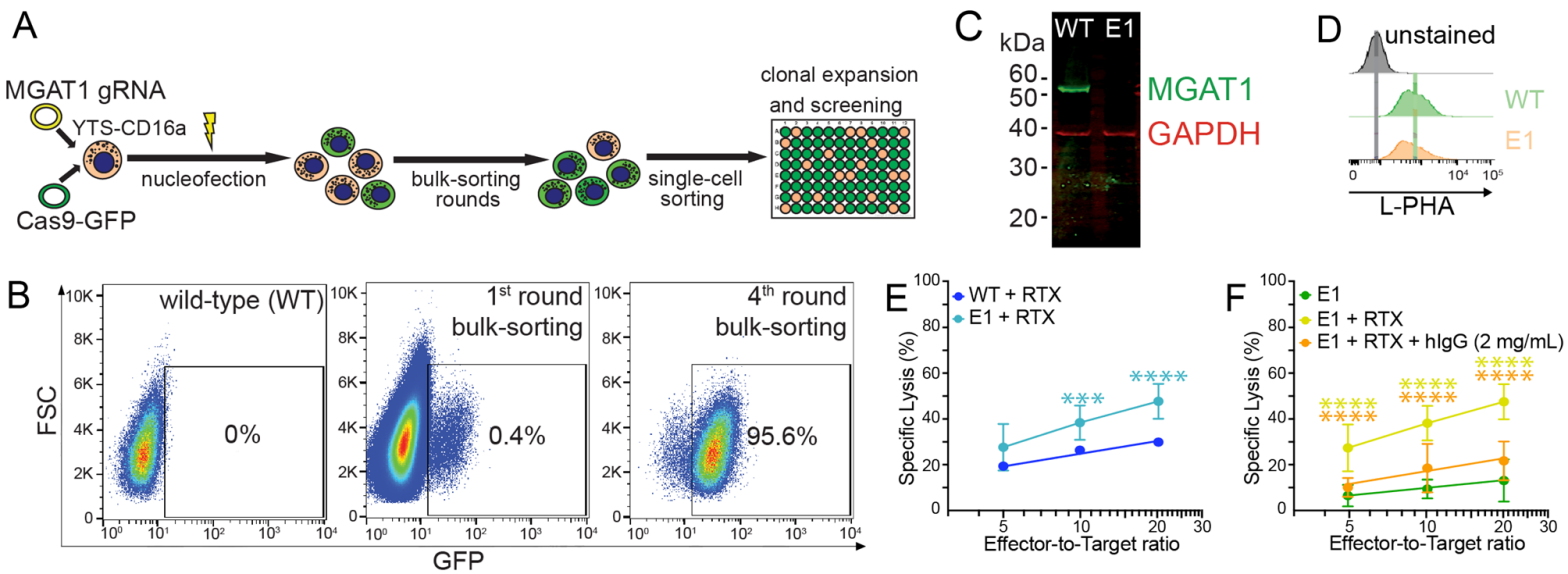
MGAT1 knockdown affects YTS-CD16a NK cell effector function and IgG binding sensitivity to CD16a receptor. (**A**) Schematic diagram of the dual CRISPR-Cas9 plasmid system and strategy used in this study to generate MGAT1 knockdown clones with the YTS-CD16a cell line, (**B**) Representative flow cytometry plots of the multiple rounds of bulk-sorting used in this study to increase GFP + YTS-CD16a cell population, (**C**) Western blot and densitometry of the MGAT1 knockdown clone E1, (**D**) Representative flow cytometry histograms showing decreased L-PHA lectin staining of MGAT1 knockdown clone E1, (**E**) ADCC (by TR-F) of the MGAT1 knockdown YTS-CD16a clone E1 and WT cells. Data shown include three independent experiments collected on different days, each with three replicates, by two-way ANOVA, (**F**) The MGAT1 knockdown clone E1 is likewise sensitive to blocking IgG (2 mg/mL) in ADCC (by TR-F) by two-way ANOVA. Data shown include three independent experiments collected on different days, each with three replicates. RTX, rituximab (20 µg/mL). For panels E and F, ***p < 0.001; ****p < 0.0001.

To confirm that the blocking IgG effect was due to *N*-glycan processing and not due to an off-target effect of kifunensine, we generated a YTS-CD16a clone deficient in MGAT1 activity. MGAT1 catalyzes the first step in the synthesis of highly processed hybrid and complex-type glycans^23,24^. Thus, inhibiting MGAT1 activity is expected to generate cells that express predominantly oligomannose-type *N*-glycans. We used an established CRISPR-CAS9-GFP strategy that targets the human MGAT1 N-terminal domain^25^ (Fig. 4a). After multiple bulk-sorting rounds, the GFP + population was increased to 95.6%, from which single cells were sorted and allowed to develop into stable cell populations (Fig. 4b). We selected one for further study, which was designated clone E1. Analyses of Western blots indicated significant MGAT1 reduction for the E1 clone and reduced staining with the fluorescently-labeled *Phaseolus vulgaris* hemagglutinin (L-PHA) that binds to complex *N*-glycans, downstream products of MGAT1^26^, specifically β1,6GlcNAc branches (Fig. 4c,d, Fig. S4).

Mass spectra of *N*-glycans released from the MGAT1 knockdown E1 whole cell lysate are consistent with reduced but not absent, MGAT1 activity. LC-NSI-MS/MS revealed increased abundance of hybrid-type *N*-glycans (43%) and decreased complex-type *N*-glycans (22%) when compared to the wild-type YTS-CD16A cells with predominant complex-type (42%) *N*-glycans (Fig. 2, Figs. S2, S7). Interestingly, E1 retained a comparable percentage of oligomannose-type *N*-glycans (35%) as the unmodified parent cell line (34%). E1 cells showed the fucosylated paucimannose form as the most abundant species, followed by the Man8 oligomannose-type *N*-glycan. There was more abundance in the hybrid-type *N*-glycans and less abundant presence of sialylated bi- and tri-antennary complex-type *N*-glycans. It is not surprising that the E1 clone is not completely lacking MGAT1 expression or activity because lymphocytes are reportedly highly sensitive to MGAT1 depletion^27,28^. It is interesting that DNA sequence analysis of the region surrounding the intended mutation site showed both mutated and wild type sequences (57% mutant heterozygous), likely indicating at least one mutation reducing MGAT1 expression that lies outside the site targeted by the CRISPR guide RNA.

We next assessed the cytotoxic capability of the MGAT1 knockdown on YTS-CD16a cells with ADCC assays. The E1 clone showed increased ADCC at the target-to-effector cell ratios of 1:10 (from 26 to 38%; p = 0.0003) and 1:20 (30 to 48%; p < 0.0001) when compared to WT YTS-CD16a (Fig. 4e). E1 was likewise sensitive to blocking IgG, with significant reductions in contrast to the parent YTS-CD16a cell line (p < 0.0001; Fig. 4f).

### IgG affinity profiling of NK cells

**Figure 5:**
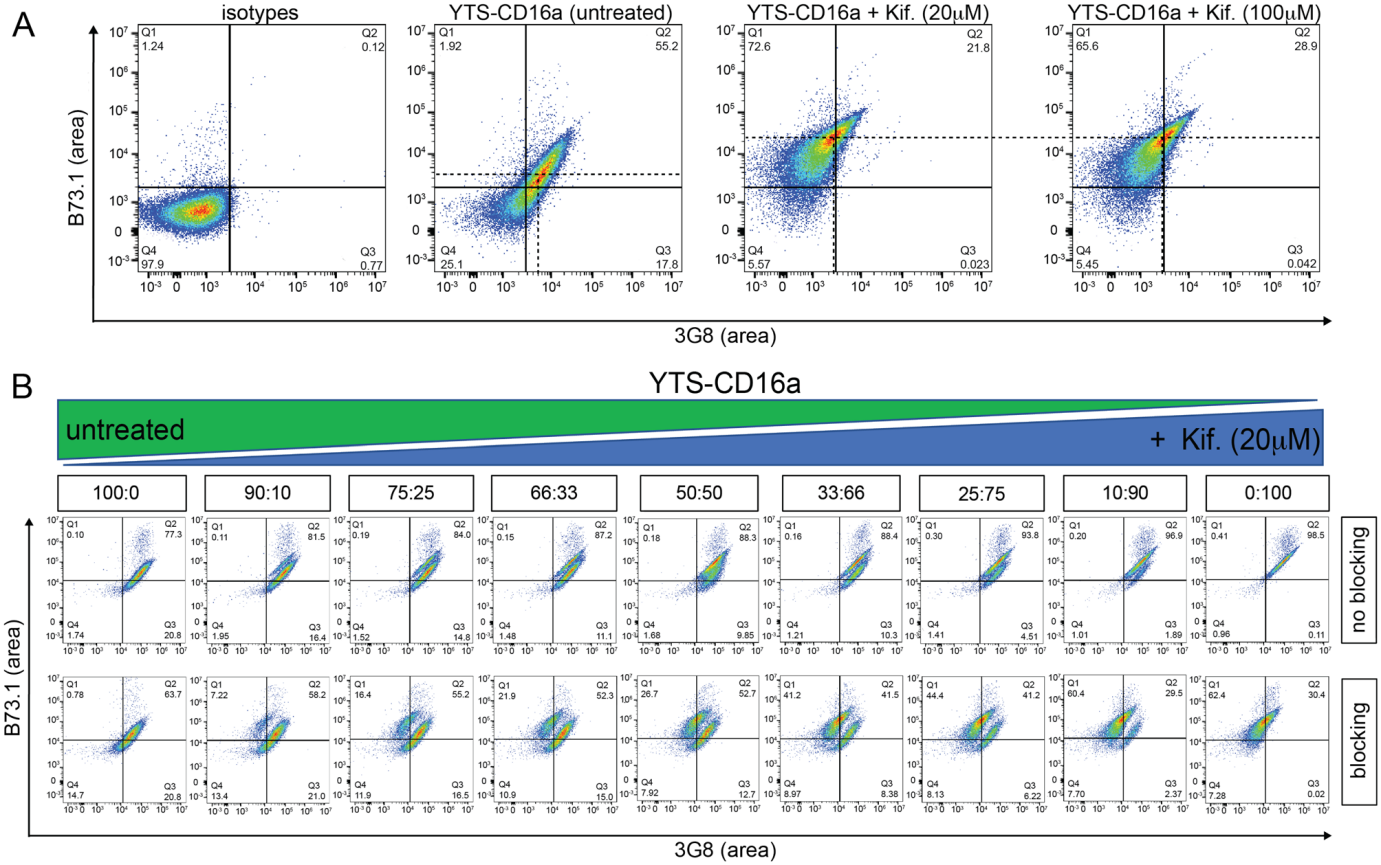
IgG sensitivity and blocking can differentiate distinct populations in YTS-CD6a cells based on CD16a epitope staining. (**A**) Representative flow cytometry plots of untreated and kifunensine (Kif.)-treated (20 and 100 µM) YTS-CD16a cells distinctly showing that the 3G8 binding of CD16a on YTS-CD16a cells (x axis) and B73.1 binding of CD16a (y-axis) is affected by IgG blocking and no significant difference is seen comparing both treatment concentrations. Dashed lines indicate the mean fluorescence intensities in each dimension. (**B**) Representative flow cytometry plots of untreated and kifunensine (Kif.)-treated (20 µM) YTS-CD16a cells gaging sensitivity in differentiating distinct populations based on *N*-glycan composition at varying percentages of WT and Kif-treated populations.

As noted, *N*-glycan processing decreases CD16a-mediated IgG binding affinity on the surface of YTS-CD16a cells compared to untreated cells^19^. Based on this, we examined if the sensitivity of CD16a to blocking IgG can be increased with a higher concentration of kifunensine treatment (100 μM). After 3 days of kifunensine treatment of YTS-CD16a cells at different concentrations (20 and 100 μM), cells were blocked with 10% human serum and stained for CD16a by two antibodies recognizing discrete epitopes: 3G8 and B73.1 (Fig. 5a). As expected, we noticed a reduction in staining with 3G8 for kifunensine treated YTS-CD16a cells upon human serum blocking due to the retention of IgG that competes for the 3G8 epitope^19^. We also noticed increased B73.1 staining in the kifunensine treated cells (Fig. 5a), despite the slight twofold decrease in affinity noted using surface plasmon resonance (Fig. S5). We observed no significant difference in CD16a staining with these antibodies between kifunensine treatment at 20 or 100 μM. This is consistent with the highly similar *N*-glycan composition determined by MS for these two treatments (Fig. 2c,d).

We next evaluated whether this “affinity profiling” approach based on differences in 3G8 and B73.1 binding could differentiate untreated vs kifunensine-treated YTS-CD16a populations by mixing various percentages of untreated and kifunensine-treated (20 μM) YTS-CD16a cells, as a potential surrogate for profiling mixed affinities with primary cells. Flow cytometry bivariate plots of the treated YTS-CD16a cells revealed distinct populations with the greater differences following blocking with polyclonal IgG (Fig. 5b). The profile of the treated cells with greater sensitivity to blocking IgG, shifted to the upper left of the plots indicating decreased binding to 3G8 and greater binding of B73.1. Thus, this flow cytometry method provides the ability to distinguish NK cells based upon IgG-binding affinity to CD16a, which we term “affinity profiling”.

**Figure 6:**
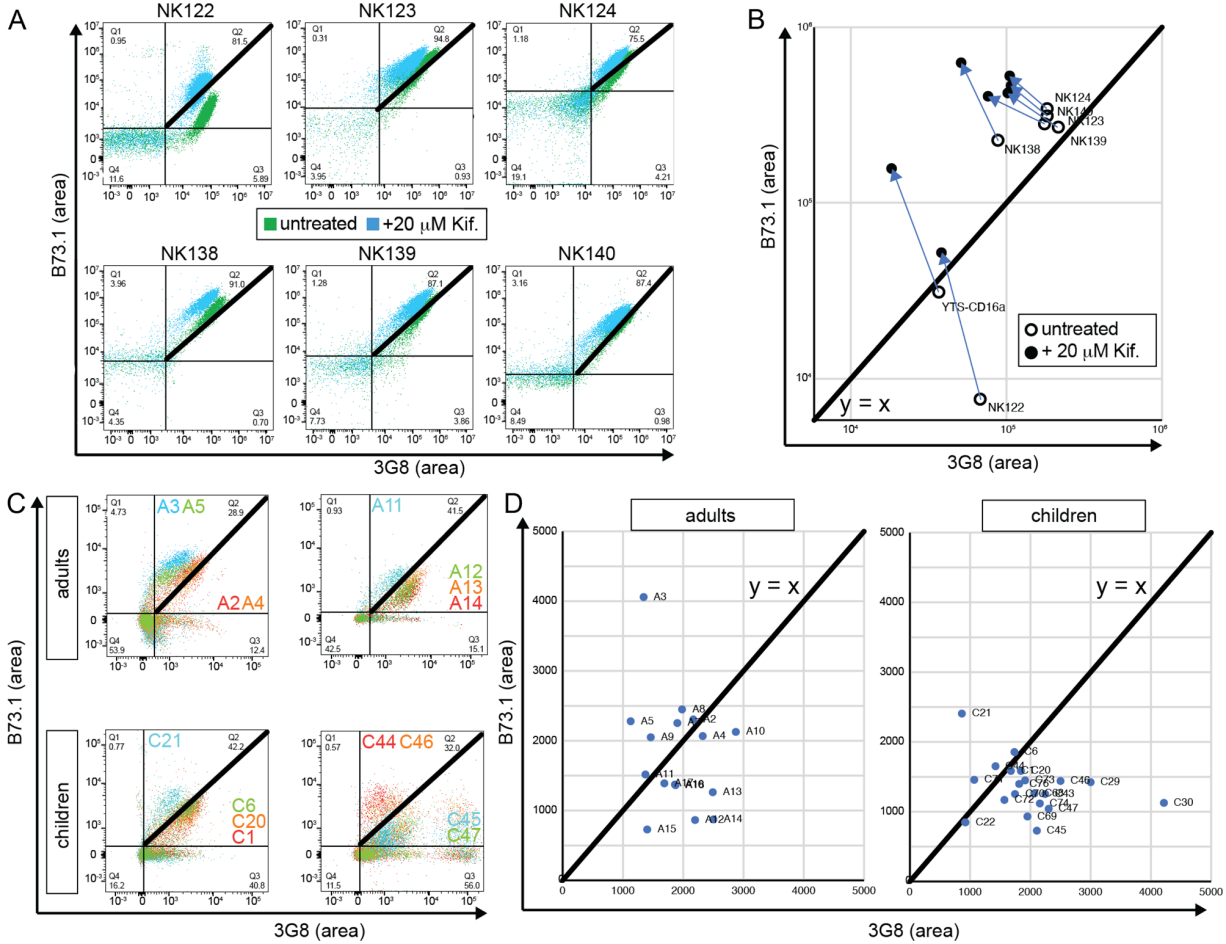
IgG affinity profiling of primary human NK cells. (**A**) Flow cytometry plots of primary NK cell donors showing decreased 3G8 staining for kifunensine (20 µM) cells after incubation with 10% human serum, (**B**) a plot of the mean fluorescence intensities (MFIs) from (A) showing increased B73.1 staining and decreased 3G8 staining associated with high affinity IgG binding following kifunensine treatment, (**C**) representative flow cytometry plots of primary NK cell donors of healthy adults and children showing considerable heterogeneity, with high affinity phenotype (44%) observed in adult population and less percentage in children population (20%), (**D**) MFIs for the B73.1+ and 3G8+ population plotted against a *heavy black* y = x line with NK cells exhibiting greater antibody-binding affinity expected above this line.

**Figure 7:**
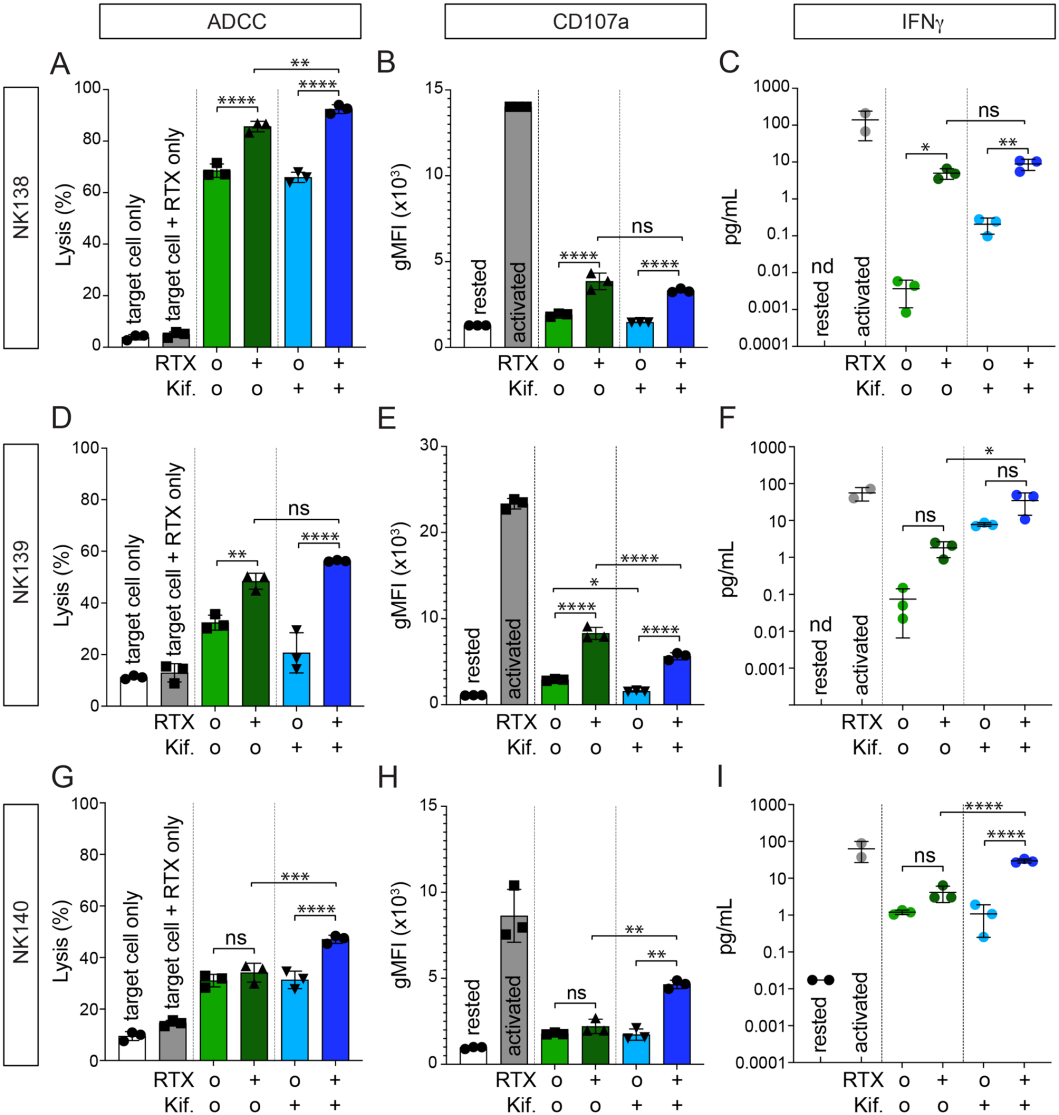
ADCC (by flow cytometry) following treatment with kifunensine for primary NK cells. (**A**,**D**,**G**) Lysis, (**B**,**E**,**H**) CD107a, (**C**,**F**,**I**) hIFNγ production of NK cells from donors NK138, NK139 and NK140 treated with kifunensine (“Kif.”; 20 µM) at 20:1T:E ratio. Means are indicated with a horizontal black line ± SD. RTX, rituximab (20 µg/mL). For all panels, one-way ANOVA, *p < 0.05; **p < 0.01; ***p < 0.001; ****p < 0.0001; *ns* not significant, *n.d*. not detected.

We then evaluated whether affinity profiling could differentiate untreated and (20 μM) kifunensine-treated primary NK cells from six healthy donors. All primary NK cells treated with kifunensine showed reduced L-PHA staining when compared to untreated primary cells from same donor indicating a reduction in *N*-glycan processing at the surface (Fig. S6). Moreover, all primary NK cells treated with kifunensine showed a population shift, and thus differentiated treated and untreated primary cells from same donor (Fig. 6a). Cells from each source demonstrated a clear shift following kifunensine treatment to greater staining with the B73.1 antibody and lower with 3G8 (Fig. 6b). Of these cells from six donors, NK122 showed no cytotoxicity under any conditions evaluated, but NK123 showed a strong increase (Fig. 3). Unfortunately, we were unable to examine the NK124 cells for activity, but NK cells from the NK138 and 140 donors showed increased ADCC following kifunensine treatment (NK139 was increased but fell short of significant at p < 0.05), as well as significantly increased IFNγ production for two of three donors tested (Fig. 7). Interestingly, we only found an increase in CD107a staining as a measure for NK cell degranulation following kifunensine treatment with one donor of three examined (NK140). Thus, the majority of cells in this panel showed both an increase in ADCC and shifts to the upper left in the affinity profiling analyses that is consistent with higher IgG-binding affinity.

Lastly, we applied the affinity profiling approach NK cells previously collected from a cohort of 16 healthy adults and 20 healthy children^29^. As expected, CD16 staining for the B73.1 and 3G8 epitopes was correlated for each donor (representative plots in Fig. 6c). However, an overlay of these plots revealed considerable heterogeneity. For example, seven of the 16 adult donors demonstrated affinity profiling results consistent with high affinity, evident from the MFI that was above the y = x line (showing greater B73.1 staining; Fig. 6d). By contrast, only four of 20 child donors revealed an MFI in this region, indicating generally lower affinity in NK cells from children. We did not observe any strong correlations between the affinity profiling and age or sex. It is interesting that we observed adult and child donors within the cohort with shifts in the plot to the lower right (greater 3G8 staining), which was not observed with our donor NK or YTS-CD16a cells. This shift may indicate greater processing, rather than less. These data indicate that the IgG affinity of CD16a in donor NK cells is variable, with shifts towards greater affinity more commonly observed with older donors than with children. By comparison to the functional studies presented above, these data indicate that a significant proportion of adult donors harbor NK cells with increased affinity for IgG.

## Discussion

Phenotype profiling of NK cells from healthy donors revealed substantial variability within both donor pools. Increased signal from the B73.1 antibody and decreased signal form the 3G8 antibody identified by flow cytometry correlated with increased ADCC and likely increased CD16a affinity for binding IgG1 through the Fc region. Thus, affinity profiling represents a simple approach to characterize a critical functional attribute of NK cells. This conclusion is supported with a wealth of data using kifunensine treated YTS-CD16a cells and primary NK cells. Furthermore, this conclusion is consistent with previous observations, including the identification of variability in CD16a *N*-glycan composition, notably at the N162 site that influences IgG Fc binding affinity^15,16,18^. In contrast to the N162 site, compositions at the remaining four *N*-glycosylation sites were remarkably similar among different donors, suggesting NK cells regulate the antibody-mediated immune response through modulating *N*-glycan composition. Lastly, the observation reported here that, on balance, children showed lower IgG affinity compared to adults is consistent with the observation that the abundance of oligomannose *N*-glycans, providing high IgG affinity on NK cells, increase with age and inflammation^30–32^. One weakness of the current study is a lack of donors with only the F158 allotype (Table S1), and thus it’s possible that these donors show a different response in the affinity profiling experiments. Furthermore, we found no correlations between donor genotype in these samples and NK cell functional or phenotypic differences potentially due to the relatively small sample size.

The phenotypic distinction of different NK cells, as noted, results from differences in IgG binding affinity. The data presented here are consistent with the previous observation that an inhibitor of *N*-glycan processing increased IgG affinity at the surface of NK cells and provided increased ADCC for both YTS-CD16a and primary NK cells^19^. Here we have demonstrated that the enhanced NK cells indeed have reduced *N*-glycan processing and greater IFNγ secretion (for two of three donors). Furthermore, we observed similar IgG binding affinity and ADCC potency in a NK clone with reduced MGAT activity, confirming that *N*-glycan processing rather than an off-target effect of kifunensine is likely responsible for the functional differences.

YTS-CD16a NK cells with reduced *N*-glycan processing and increased IgG binding affinity also proved susceptible to inhibition with non-specific serum IgG (Fig. 1). Surprisingly, the ADCC potency of primary NK cells from two donors showed strong inhibition, even in the absence of kifunensine (Fig. 3). It is possible the primary NK cells express factors increasing sensitivity to IgG; YTS cells do not naturally express CD16a, and thus differ from primary cells in multiple key ways^29^.

It is not clear to what extent the results of these experiments predict that IgG levels potentially inhibit ADCC in the body. It is known that tight IgG binding affinity of CD16a increases both in vitro ADCC and the patient response to monoclonal antibody therapies where ADCC is a key attribute; patients with the high affinity CD16a V158 allotype generally respond more favorably than those expressing the low affinity F158 variant^33–35^. Thus, the increased CD16a affinity does not inhibit efficacy of the monoclonal antibodies in vivo. This apparent discrepancy may result from a few key differences between the in vitro ADCC assays and antibody-dependent killing by NK cells in vivo. In one example, our ADCC assay is completed in 2 h, in contrast to killing in vivo that may last for days or more. Greater contact time between NK cells and an opsonized target is expected to promote greater CD16a engagement due to high valency of the interaction, and allow dissociation and replacement of the serum IgG that is solely a monovalent interaction. The multivalent interaction is expected to promote greater target killing and thus antibody efficacy.

The ongoing development of engineered NK cells demonstrates the promise of improved therapeutics, and a new landscape for improving ADCC. It would be challenging to modulate the glycan processing of endogenous NK cells for a number of reasons, however, modulating CD16a affinity or CD16a glycan processing through cell engineering prior to infusion is highly feasible. Our prior kinetic analysis of CD16a binding and present ADCC results suggest that the NK cells with high affinity CD16a glycoforms bind strongly and fast, and dissociate slowly from IgG. Thus, mixing engineered NK cells and target-specific IgG prior to infusing has the potential to provide a rapid response, with minimal inhibition from serum IgG.

In conclusion, we developed an affinity profiling strategy to evaluate functionally relevant differences in CD16a affinity for IgG, and identified clear differences among healthy donors. We also determined that *N*-glycan processing affects ADCC in NK cells, providing a novel pathway towards improving NK cell responses.

## Materials and methods

### Materials

All materials purchased from Millipore-Sigma unless otherwise noted.

### IgG purification

Human IgG (Athens Research & Technology, Inc) was purified using a Superdex 200 size exclusion column as previously described^19^.

### Cell culture

The natural killer cell lymphoblastic leukemia/lymphoma cell lines YTS and YTS-CD16a (V/V allotype) (a gift from Dr. Konrad Krzewski, NIH) cells were grown in RPMI 1640 medium supplemented with 10% FBS, 2 mM l-glutamine, 10 mM HEPES, 1 mM sodium pyruvate, 1 mM non-essential amino acids, and 50 U/mL penicillin/streptomycin. Raji cells were purchased from ATCC (#CCL-86) and were grown in RPMI 1640 medium supplemented with 10% FBS, 2 mM l-glutamine, and 50 U/mL penicillin/streptomycin. Raji and YTS cells were grown in suspension at 37 °C, 5% CO_2_. Trypan blue uptake was assessed to identify non-viable cells when measuring cell density of a culture using a TC-20 automated cell counter (Biorad).

### Primary NK cell isolation

NK cells were isolated from leukocyte reduction filters obtained from Shepeard Community Blood Centers (Augusta, GA), after the plateletpheresis procedure was performed on healthy donors. Donors signed consent forms permitting the use of donated blood products for research purposes. We do not directly enroll donors because we obtain deidentified materials from the Shepeard Blood Center, and thus donors were not directly involved in the study. Cell isolation procedures were performed within 24 h of the time the donors completed apheresis procedure. All NK cells were isolated from apheresis filters by negative selection and profiled accordingly as previously described, and viability was assessed by trypan blue, as well as Zombie Violet/Green^15,18,19^. Donor CD16a genotypes were determined as previously described^36^. NK cells were cultured in the YTS medium described above but supplemented 5 ng/mL of IL-15. The kifunensine group (20 μM; Cayman Chemicals) was treated for 3 days. Donor information can be found in Table S1. Male donors were selected because they generally provide greater number of NK cells needed for these studies. Primary NK cells were used fresh for functional assays and not cryopreserved.

### Time-resolved fluorescence-based (TR-F) ADCC assays

The DELFIA EuTDA Cytotoxicity Kit was used and optimized per manufacturer’s instructions (Perkin Elmer). The Eu-TDA DELFIA assay, which uses TR-F, is a non-radioactive alternative that offers similar sensitivity to the ^51^Cr release assay^37^, and performed as previously reported^19^. Briefly, untreated and kifunensine treated effector cells were added to a V-bottom 96-well plate and blocked with purified IgG (0–0.5–2 mg/mL) for 1 h at 37 °C, 5% CO_2_, and then antibody (rituximab (Selleckchem) or afucosylated rituximab (InvivoGen)) at 0–20 μg/mL was added. Raji cells were labeled with 5 μL of BATDA reagent at 1 × 10^6^ cells/mL in loading buffer (RPMI 1640 medium without phenol red, 10% FBS, 10 mM HEPES) for 30 min at 37 °C, 5% CO_2_. The cells were washed 5 × 10 mL with PBS, and resuspended at 2 × 10^5^ cells/mL in assay medium (RMPI 1640 medium, 10 mM HEPES). Target Raji cells (10,000 cells in 50 μL) were incubated with effector cells for 2 h at 37 °C, 5% CO_2_ at specified target-to-effector (T:E) ratios for each experiment in triplicate. The 96-well plate was then centrifuged for 5 min at 100×*g*, and 20 μL of each supernatant was then combined with 200 μL of europium solution in a separate flat-bottom 96-well plate, followed by a 15 min incubation with orbital shaking at RT. The fluorescent signal was measured with a Neo2 plate-reader (Biotek) with time-resolved fluorescence (TR-F) filter readout parameters. Controls in each experiment monitored Background, Maximum-to-Spontaneous signal ratio as well as Spontaneous Release (%). Specific Lysis (%) was calculated as: [(observed signal − spontaneous signal)/(maximum signal − spontaneous signal)] × 100. Maximum signal was obtained by incubating cells with 10 μL lysis buffer per manufacturer’s instructions. Statistical analysis was performed with Prism 6.09 (GraphPad software).

### Flow cytometry-based ADCC assays

Briefly, Raji cells were labeled with CellTrace™ Yellow (CTY) in PBS for 7 min in a bead bath using the CellTrace™ Yellow Cell Proliferation Kit (Invitrogen) as previously described^19,38^, then 10 mL of assay medium (RPMI 1640 medium, 10% FBS, 2 mM l-glutamine, 50 U/mL penicillin/streptomycin) were added and cells were incubated for an additional 5 min. The cells were then washed 5 × 10 mL with assay medium, and resuspended at 5 × 10^4^ cells/mL in assay medium. Target Raji cells (5000 cells, 100 μL) were incubated for 15 min with 0–20 μg/mL antibody (rituximab; Selleckchem) in a V-bottom 96-well plate, followed by addition of effector cells at 1:10 target-to-effector (T:E) ratios and further incubated for 2 h at 37 °C, 5% CO_2_ in triplicates. Effector cells were previously treated with kifunensine (20 μM) for 3 days. Cells were then washed with PBS+ azide and stained for viability using Zombie Violet (1:1000, BioLegend #423,114) for 30 min at RT. Cells were washed 3 × 5 min with PBS+ azide, and fixed with 1% PFA for 20 min at RT, followed by 3 × 5 min washes with PBS+ azide. A collection of 2500 events gated for CTY+ cells per sample were acquired on a CytoFLEX instrument (Beckman Coulter), and analysis performed using FlowJo 10 (BD Biosciences). Specific Lysis (%) was calculated as the percentage of dead CTY+ target cells. Controls in each experiment monitored viability upon labeling of target cells with CellTrace reagent, as well as viability upon incubation with antibody as well.

### MGAT1 knockdown YTS-CD16a cells

For the CRISPR knock-out generation, a nucleofection strategy was chosen since YTS cells are considered difficult-to-transfect cells. Two plasmids, one containing the Cas9-GFP system and the other the gRNA for human MGAT1^25^ were added to a total of 2 × 10^6^ YTS-CD16a cells suspended in 100 μL Nucleofector solution according to manufacturer’s protocol (Kit V, Lonza #VCA-1003). Plasmids were introduced using program O-017 on the Amaxa Nucleofection II System (Lonza). Cells were then incubated for 24 h, stained with propidium iodide (PI) for 15 min and bulk-sorted based on the following gating strategy: G1 (forward and side scatter population), G2 (singlets), G3 (PI^-^ viable cells), and G4 (GFP^+^) using a S3 Cell Sorter (Biorad). A second nucleofection of the previously bulk-sorted population was performed after cells had above 90% viability, followed by bulk-sorting to further enrich GFP^+^ cells. Cells were then grown for 14 days, then GFP^+^ cells were single-cell sorted into flat-bottom 96-well plates in pre-conditioned growth medium using a MoFlo Astrios EQ cell sorter (Beckman Coulter). Single-cell sorts were allowed to grow in 96-well plates in YTS pre-conditioned medium for 14 days, followed by clonal screening for MGAT1 expression by western blot, L-PHA and 3G8 staining by flow cytometry and genotyping analysis.

### Immunoblotting

A total of 3 × 10^6^ cells were collected and lysed by continuous pipetting in 120 μL of SDS-PAGE buffer A (50 mM TRIS, 1% SDS, pH 6.8, Halt protease inhibitor), vortexed briefly, then 1 mL of cold acetone was added, followed by an additional vortex step, then incubated on ice for 10 min. Samples were centrifuged at 4400*g* for 15 min at 4 °C. The protein pellet was resuspended in 50 mM NaOH, and protein concentration was determined using Pierce BCA Protein Kit. Samples of 25 μg total lysate were resolved on a 10% SDS–polyacrylamide gel and transferred onto a polyvinylidene difluoride membrane using the one-step electroblotting system (Pierce Power Station; ThermoFisher). The membrane was blocked with 5% dry milk in TBS Tween 20 (TBST) buffer for 1 h at RT. Following blocking, the membrane was stained for 18 h at 4 °C with anti-MGAT1 (rabbit, 1:1000, Abcam #ab180578) in 5% milk in TBST. The blot was washed 3 × 10 min with TBST and stained with secondary anti-rabbit AF800 (1:10,000, Invitrogen #A32735) in 5% milk in TBST for 1 h at room temperature. Then, blot was washed 3 × 15 min and blocked once more with 5% milk in TBST for 1 h at room temperature. The membrane was then stained for 1 h at room temperature with anti-hGAPDH (goat, 1:10,000, R&D Systems #AF57185P), followed by 3 × 10 min washes with TBST. The blot was then incubated for 1 h at RT with the anti-goat AF680 (1:10,000, Invitrogen #AF32860) secondary antibody in 5% milk TBST, and washed 3 × 15 min with TBST. The dual-labeled blot was imaged on an Odyssey CLx Image system (LICOR Biosciences). Densitometry was performed using ImageJ software.

### MGAT1 genotyping and indel analysis

Unmodified wild-type YTS-CD16a cells as well as cells with reduced MGAT1 levels (3 × 10^6^ cells) were pelleted and genomic DNA was isolated using QIAamp Blood Mini Kit per the manufacturer’s protocol (Qiagen). A PCR reaction with published primers encapsulating the chosen MGAT1 gRNA were used: 5ʹ-CCAGGATGCTGAAGAAGCAGTCT-3ʹ (forward), 5ʹ-TGGCTAACGATGATGGGGAAG-3ʹ (reverse)^25^. DNA was amplified using GoTaq Green MasterMix (Promega), and PCR products were purified by 1% agarose gel using SV Wizard PCR and Gel Clean-Up Kit (Promega). Purified DNA was sent for Next-Generation-Sequencing analysis by Amplicon-EZ (Azenta Life Sciences).

### Clonal screening by flow cytometry

A total of 500,000 effector cells were stained for viability using Zombie Violet (1:1000, BioLegend #423114) for 30 min at RT. Cells were washed 3 × 5 min with PBS+ azide, followed by 10 min blocking with 10% human serum at 4 °C. Cells were then stained with anti-hCD16 (3G8-APC, BioLegend #302011) or an isotype control (Iso-APC, BioLegend #400120) for 30 min at 4 °C. Cells were washed 3 × 5 min with PBS+ azide, and fixed with 1% PFA for 20 min at RT, followed by 3 × 5 min washes with PBS+ azide. Cells were then incubated with lectin L-PHA-AF594 (5 μg/mL, Invitrogen #L32456) for 20 min at 37 °C, and washed 3 × 5 min with PBS azide. A collection of 5 × 10^4^ events per sample were acquired on a CytoFLEX instrument (Beckman Coulter), and analysis performed using FlowJo 10 (BD Biosciences).

### Analysis of CD16a on NK cells using flow cytometry

A total of 500,000 YTS-CD16a cells containing various percentages (from 0 to 100%) of untreated and kifunensine (20 μM) treated cells were stained for viability using Zombie Green (1:1000, BioLegend #423111) for 30 min at RT. Cells were washed 3 × 5 min with PBS+ azide, followed by 10 min blocking with 10% human serum at 4 °C. Cells were then stained with anti-hCD16 antibodies (2 µg/mL 3G8-APC, BioLegend #302011; 2 µg/mL B73.1-BV421, BioLegend #360724) or their isotype controls (Iso-APC, BioLegend #400120; Iso-BV421, BioLegend #400158) for 30 min at 4 °C. Antibody concentrations for CD16 staining were chosen to be in great excess of the receptor concentration. For example, previous reports indicate ~ 25,000 CD16a/NK cell thus for 5 × 10^5^ cells/mL = 1.3 × 10^10^ CD16a/mL = 2.1 × 10^–11^ M. 3G8 (2 µg/mL) = 1.3 × 10^–8^ M, and the *K*_D_ = 3–6 × 10^–9^ M, thus we expect the vast majority of receptors to be bound with antibody. Cells were washed 3 × 5 min with PBS+ azide, and fixed with 1% PFA for 20 min at RT, followed by 3 × 5 min washes with PBS+ azide. A collection of 5 × 10^4^ events per sample were acquired on a CytoFLEX instrument (Beckman Coulter), and analysis performed using FlowJo 10 (BD Biosciences). The same hCD16a staining was done for isolated donor primary NK cells (NK122, NK123, NK124, NK138, NK139, NK140) with L-PHA staining as mentioned previously.

### PBMC data analysis for B73.1 and 3G8

Raw data from PBMCs isolated from healthy children and adults was acquired from a previously published study^29^. Demographic information on these donors is reported in Table S2. Dual staining analysis for CD16a based on B73.1 and 3G8 epitopes was performed using FlowJo 10 (BD Biosciences).

## CD107a quantitation

Briefly, effector cells were labeled with CellTrace™ Yellow (CTY) in PBS using the CellTrace™ Yellow Cell Proliferation Kit (Invitrogen) for 7 min at 37 °C as previously described^38,39^, then 10 mL of assay medium (RPMI 1640 medium, 10% FBS, 2 mM l-glutamine, 10 mM HEPES, 1 mM sodium pyruvate, 1 mM non-essential amino acids, 50 U/mL penicillin/streptomycin) were added and cells were incubated for an additional 5 min. The cells were then washed 5 × 10 mL with assay medium, and resuspended at 5 × 10^4^ cells/mL in assay medium. Target Raji cells (50,000 cells, 100 μL) were incubated for 15 min with 0–20 μg/mL antibody (rituximab; Selleckchem) in a V-bottom 96-well plate, followed by addition of effector cells at 1:1 target-to-effector (T:E) ratio, as well as anti-hCD107a (CD107a-FITC, BioLegend #328606) or the isotype control (Iso-FTIC, BioLegend #400108) and further incubated for a total of 1 h at 37 °C, 5% CO_2_ in triplicates. Effector cells were previously treated with kifunensine (20 μM) for 3 days. For positive controls, effector cells were activated with 1 μg/mL phorbol-myristate-acetate (PMA) and 1 μg/mL ionomycin. Following the first hour of incubation, 6 μg/mL GolgiStop and 10 μg/mL GolgiPlug were added to the sample wells and further incubated for 3 h at 37 °C, 5% CO_2_. Cells were then washed with PBS+ azide and stained for viability using Zombie Violet (1:1000, BioLegend #423114) for 30 min at RT. Cells were washed 3 × 5 min with PBS+ azide, and fixed with 1% PFA for 20 min at RT, followed by 3 × 5 min washes with PBS+ azide. A collection of 2500 events gated for CTY+ cells per sample were acquired on a CytoFLEX instrument (Beckman Coulter), and analysis performed using FlowJo 10 (BD Biosciences). CTY+ cells were further gated for viability, and viable cells were assessed for CD107a upregulation by FITC geometric mean fluorescence intensity analysis. Controls in each experiment monitored viability upon labeling of target cells with CellTrace reagent, as well as viability upon incubation with antibody as well.

### hIFNγ quantitation

Serum human IFNγ levels of isolated NK cell donors were examined by collecting supernatant from the ADCC assays and using IFNγ Human ProQuantum Immunoassay Kit (A35576, Invitrogen) using 20 μL volume reactions. Data was acquired on a StepOnePlus Real-Time PCR system (Applied Biosystems) and analyzed per manufacturer’s instructions by the ProQuantum cloud-based software.

### Mass spectrometry of permethylated *N*-glycans

NK cells were collected and boiled for 10 min at 100 °C, cooled down, and trypsin (Promega) was added prior to overnight incubation at 37 °C. Samples were lyophilized and resuspended in GlycoBuffer2 and PNGase F (New England Biolabs) was added and samples incubated overnight at 37 °C. Samples were lyophilized, then resuspended in 5% acetic acid (in water), and released *N*-glycans were eluted from C18 cartridges according to manufacturer’s instructions (Sep-Pak, 100 mg; Waters). Samples were lyophilized and permethylated as previously described^40^. The samples were analyzed on an Orbitrap Fusion Tribrid (ThermoFisher) connected in-line to a Thermo Ultimate RSLCnano chromatography system (ThermoFisher). A commercial LC-column (Acclaim PepMap 100 C18 (3 μm, 100 Å, 75  μm × 15 cm) was used for separation. A gradient including mobile phase A consisting of 98% water, 2% acetonitrile, and 1 mM sodium acetate and mobile phase B consisting of 20% water and 80% acetonitrile were used for a linear gradient from low to high percentage of mobile phase B within a 72-min gradient. The data dependent precursor ion scan was collected from m/z 600–2000 m/z at 120,000 resolution inOrbitrap mode and precursors at a time frame of 3 s were selected for subsequent MS/MS fragmentation (CID; 40%) at 15,000 resolution. Precursors with either an unknown charge state or +1 charge state were excluded, and dynamic exclusion was enabled for a 30 s duration. MS2 fragmentation patterns were evaluated using GlycoWorkBench software^41^. Mass spectrometry by MALDI was also performed on the samples and collected on a RapifleX Tissuetyper (Bruker) in linear positive mode with 2,5-dihydroxybenzoic acid used as a matrix. MS1 (LC-NSI-MS; MALDI) spectra for identified *N*-glycans can be found in Supplemental Data (Figs. S2, S7).

### Surface plasmon resonance

GFP-CD16a (1 µg/mL) expressed from HEK293F cells using Freestyle medium (ThermoFisher) was coupled to the surface of a Series S CM5 sensor chip on an Akta T200 instrument (Cytiva) in 10 mM sodium acetate pH 5.0. Following coupling, the sensor surface was equilibrated with 1 × phosphate buffered saline, 0.05% P20, pH 7.4; this buffer was used as the “running buffer” for all following experiments. Rituximab was likewise expressed from HEK293F cells. Affinities were estimated using a single cycle kinetics method where the sensor surface was washed (± 100 nM B73.1) for 450 s, followed by an additional 150 s of washing with running buffer. Five injection cycles of increasing rituximab concentrations in running buffer were applied to the surface as follows (150 s injection, 70 s wash with running buffer). The sensor surface was regenerated between experiments by applying 100 mM glycine, pH 3.0 for 30 s followed by equilibration with running buffer for 500 s. Affinities were determined by fitting the signal once an equilibrium had been established during the application of rituximab. Affinities were fitted with a 1:1 Langmuir binding isotherm.

### Statistical analyses

All statistical analyses were performed with Excel (Microsoft), Prism 6.09 (GraphPad software), or R Studio (Version 2023.12.0+369, Posit Software, PBC).

### Supplementary Information

Supplementary Information 1.

Supplementary Information 2.

## Data Availability

All data generated or analyzed during this study are included in this published article (and its Supplementary Information files). Additional information is available to the corresponding author on reasonable request.

## Abbreviations

NK: Natural killer
E:T: Effector-to-target
MFI: Mean fluorescence intensity
MGAT-1: Alpha-1,3-mannosyl-glycoprotein 2-beta-*N*-acetylglucosaminyltransferase
TR-F: Time-resolved fluorescence
MS: Mass spectrometry
LC: Liquid chromatography
NSI: Nanoelectrospray ionization
ADCC: Antibody-dependent cell-mediated cytotoxicity
mAb: Monoclonal antibody
*L-PHA*: *Phaseolus vulgaris* hemagglutinin
IFNγ: Interferon-gamma
