# Supplementary material for: Distinct CD16a features on human NK cells observed by flow cytometry correlate with increased ADCC
Source: Sci Rep. 2024 Apr 4;14:7938. doi: 10.1038/s41598-024-58541-6 (PMC10995120; doi:10.1038/s41598-024-58541-6)

Supplemental Materials for:

Distinct CD16a features on human NK cells observed by flow cytometry correlate with increased ADCC

Maria C. Rodriguez Benavente[1], Zainab A. Hakeem[1], Alexander R. Davis[1], Nathan B. Murray[2], Parastoo Azadi[1,2], Emily M. Mace[3], Adam W. Barb[1,2,4*]

[1]Department of Biochemistry and Molecular Biology, University of Georgia, Athens, GA

[2]Complex Carbohydrate Research Center, University of Georgia, Athens, GA

[3]Department of Pediatrics, Columbia University Irving Medical Center, New York, New York, USA

[4]Department of Chemistry, University of Georgia, Athens, GA

Table S1. Donor information for the NK cell activity assays.

Donor ID	Gender	Age (y)	Weight (#)	Self-reported Race	Date of Collection	NK Cells Isolated (x10^6)	CD16+, CD56+ (%)	CD16a allotype
NK121	M	31	227	white	6/22/22	36.2	89.1	V/V
NK122	M	32	225	white	7/7/22	40.4	85.0	V/V
NK123	M	34	243	black	7/7/22	42.9	89.5	V/F
NK124	M	32	231	white	7/21/22	46.0	81.2	V/V
NK138	M	66	232	white	4/19/23	148.0	94.4	V/F
NK139	M	55	268	white	4/28/23	194.5	96.9	V/V
NK140	M	71	235	white	5/4/23	193.0	90.3	V/F

Table S2. Donor information for the affinity profiling analysis.

Cohort	Donor	Gender	Age	Self-reported Race
adult	A2	M	25	white (non-hispanic)
adult	A3	F	50	white (non-hispanic)
adult	A4	M	42	asian
adult	A5	F	33	white (non-hispanic)
adult	A7	M	29	asian
adult	A8	F	32	asian
adult	A9	F	36	white (non-hispanic)
adult	A10	M	29	asian
adult	A11	F	24	white (non-hispanic)
adult	A12	M	25	black
adult	A13	M	22	white (non-hispanic)
adult	A14	F	23	white (non-hispanic)
adult	A15	M	22	asian
adult	A16	M	35	asian
adult	A17	M	37	asian
adult	A18	F	52	hispanic
children	C1	F	20	asian
children	C6	F	19	asian
children	C20	F	18	black
children	C21	F	18	black
children	C22	M	19	black
children	C29	F	11	black
children	C30	F	17	black
children	C43	F	15	hispanic
children	C44	F	19	white (non-hispanic)
children	C45	F	18	black
children	C46	M	18	asian
children	C47	F	18	hispanic
children	C68	F	5	white (non-hispanic)
children	C69	M	10	black
children	C70	M	12	black
children	C71	M	6	white (non-hispanic)
children	C72	F	16	hispanic
children	C73	M	11	hispanic
children	C74	M	13	hispanic
children	C76	M	14	hispanic

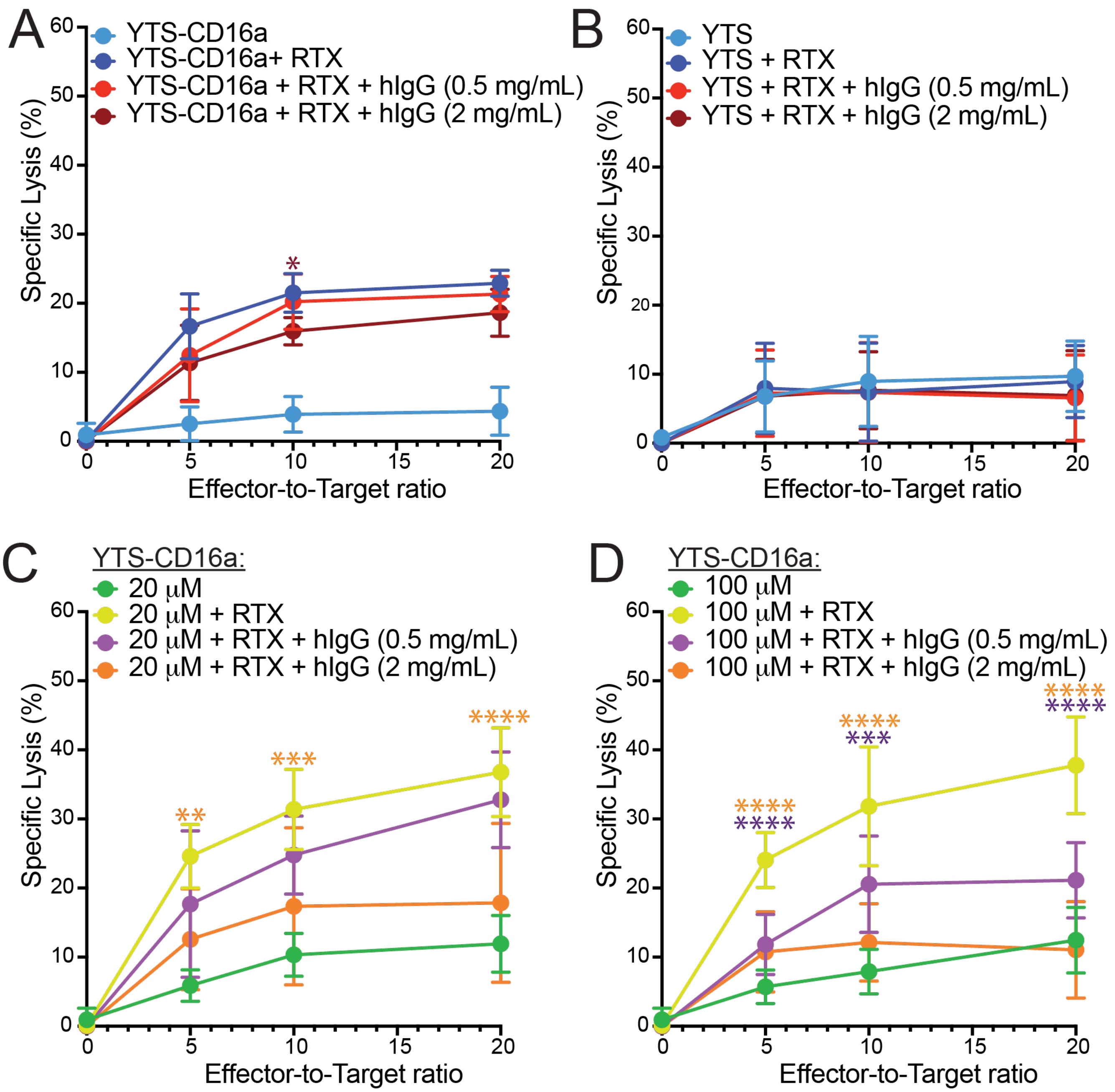

Supplemental Figure 1. Kifunensine treatment increased IgG sensitivity.
(**A**) YTS cells show no ADCC or IgG blocking due to lack of CD16a receptor. Data points represent the mean for three independent experiments collected on three different days, each with three replicates, ± SD, (**B**) YTS-CD16a cells show a slight trend in ADCC decrease only upon IgG blocking at high concentration (2 mg/mL), (**C**) Kifunensine-treated (20 μM) YTS-CD16a cells show significant decrease in ADCC with IgG blocking at high concentration (2 mg/mL). Data shown include three independent experiments collected on different days, each with three replicates, ± SD, (**D**) Kifunensine-treated (100 μM) YTS-CD16a cells show significant decrease in ADCC with IgG blocking at both low and high concentrations (0.5 and 2 mg/mL). Data shown include three independent experiments collected on different days, each with three replicates, ± SD. For all panels, *** $p < 0.001$, **** $p < 0.0001$.

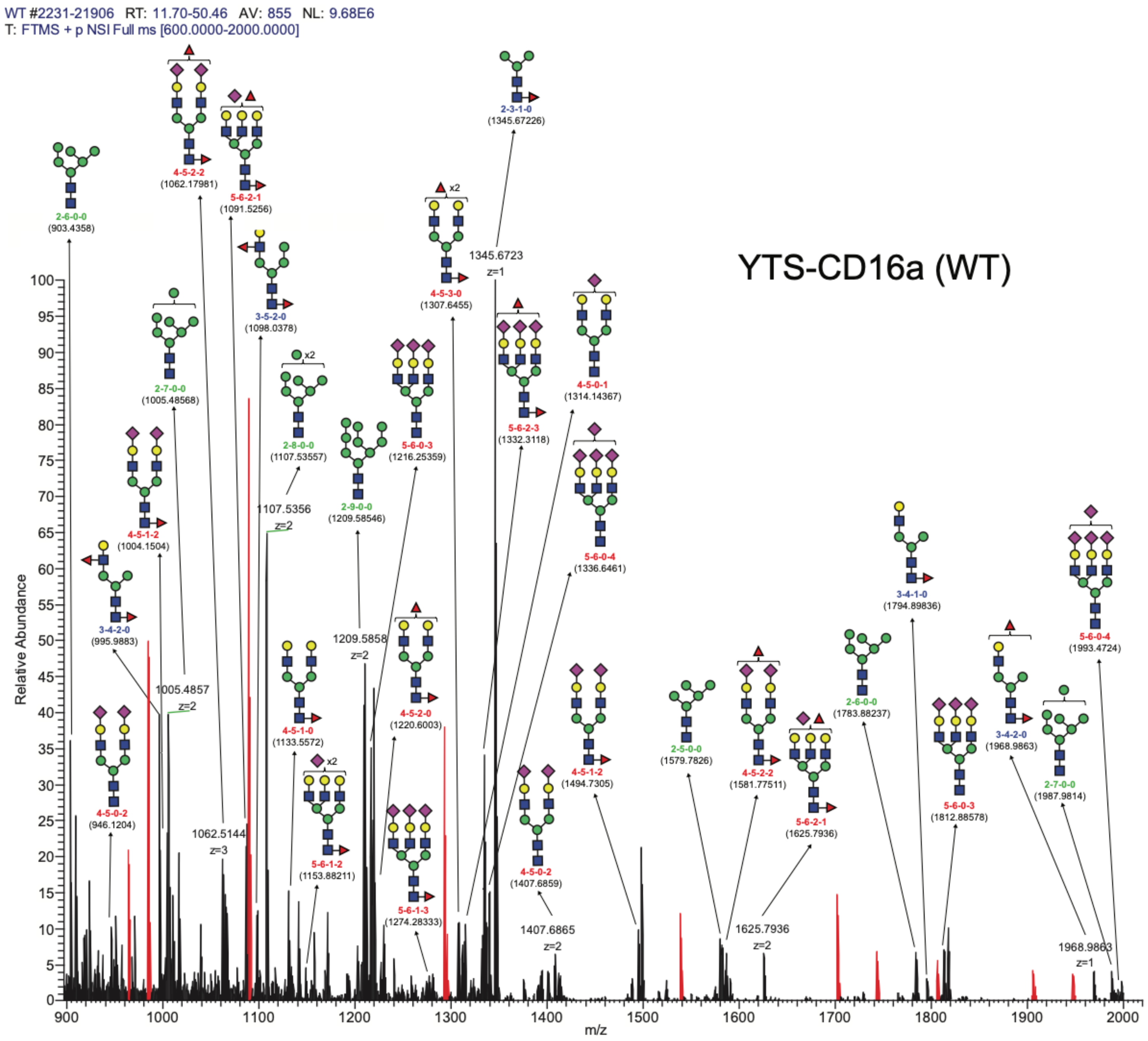

Supplemental Figure 2A. MS1 spectra of PNGaseF released permethylated N-glycans from YTS-CD16a cells.

Spectral average (11-50 minutes; m/z 900-2000) from LC ESI-MS/MS. Red peaks represent identified cellulose contamination. Masses are sodiated and reflect multiple charge states.

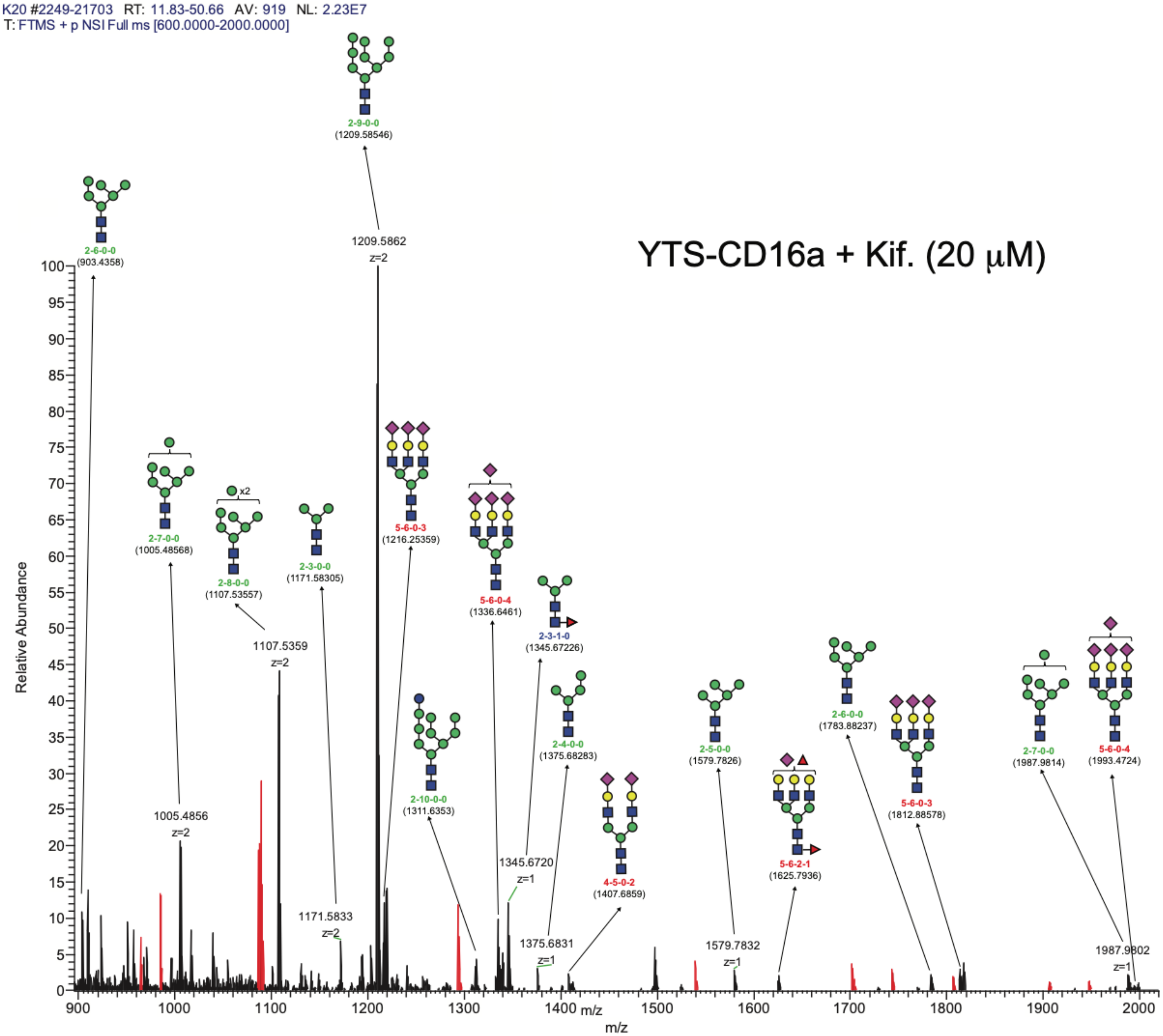

Supplemental Figure 2B. MS1 spectra of PNGaseF released permethylated N-glycans from YTS-CD16a cells treated with kifunensine (Kif. 20 μM).

Spectral average (11-50 minutes; m/z 900-2000) from LC ESI-MS/MS. Red peaks represent identified cellulose contamination. Masses are sodiated and reflect multiple charge states.

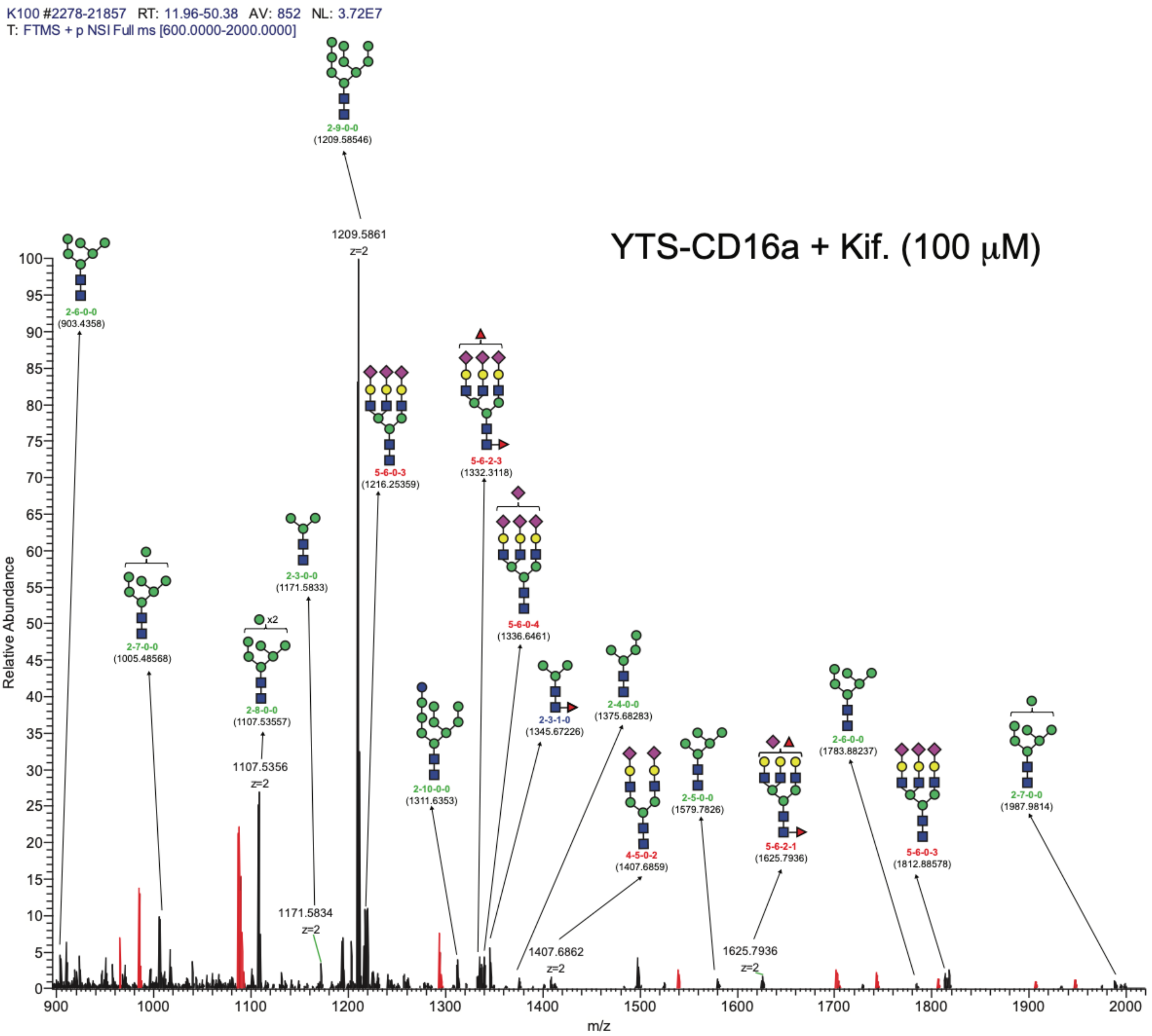

Supplemental Figure 2C. MS1 spectra of PNGaseF released permethylated N-glycans from YTS-CD16a cells treated with kifunensine (Kif. 20 μM).
Spectral average (11-50 minutes; m/z 900-2000) from LC ESI-MS/MS. Red peaks represent identified cellulose contamination. Masses are sodiated and reflect multiple charge states.

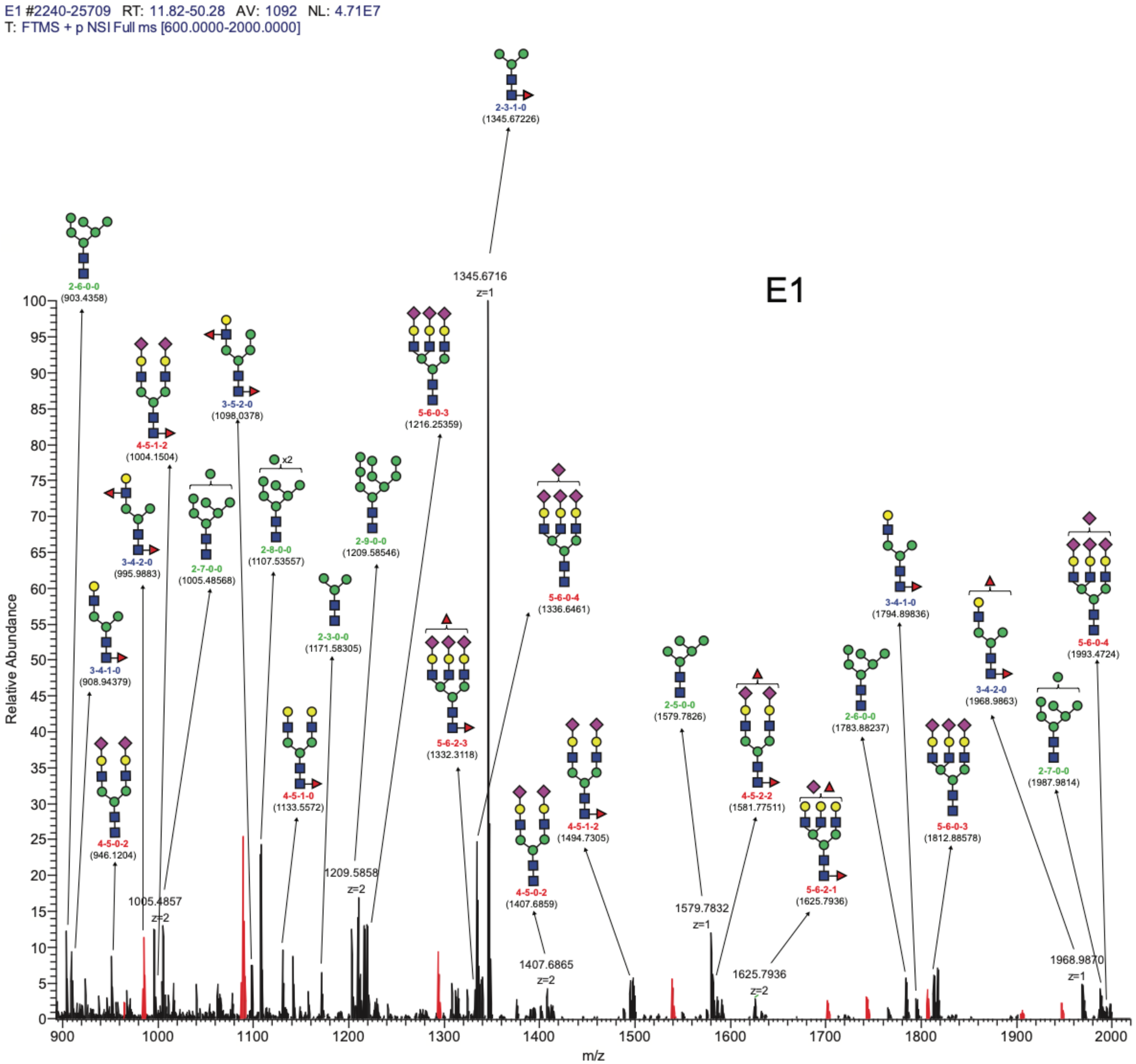

Supplemental Figure 2D. MS1 spectra of PNGaseF released permethylated N-glycans from E1 clone. Spectral average (11-50 minutes; m/z 900-2000) from LC ESI-MS/MS. Red peaks represent identified cellulose contamination. Masses are sodiated and reflect multiple charge states.

YTS-CD16a (WT)

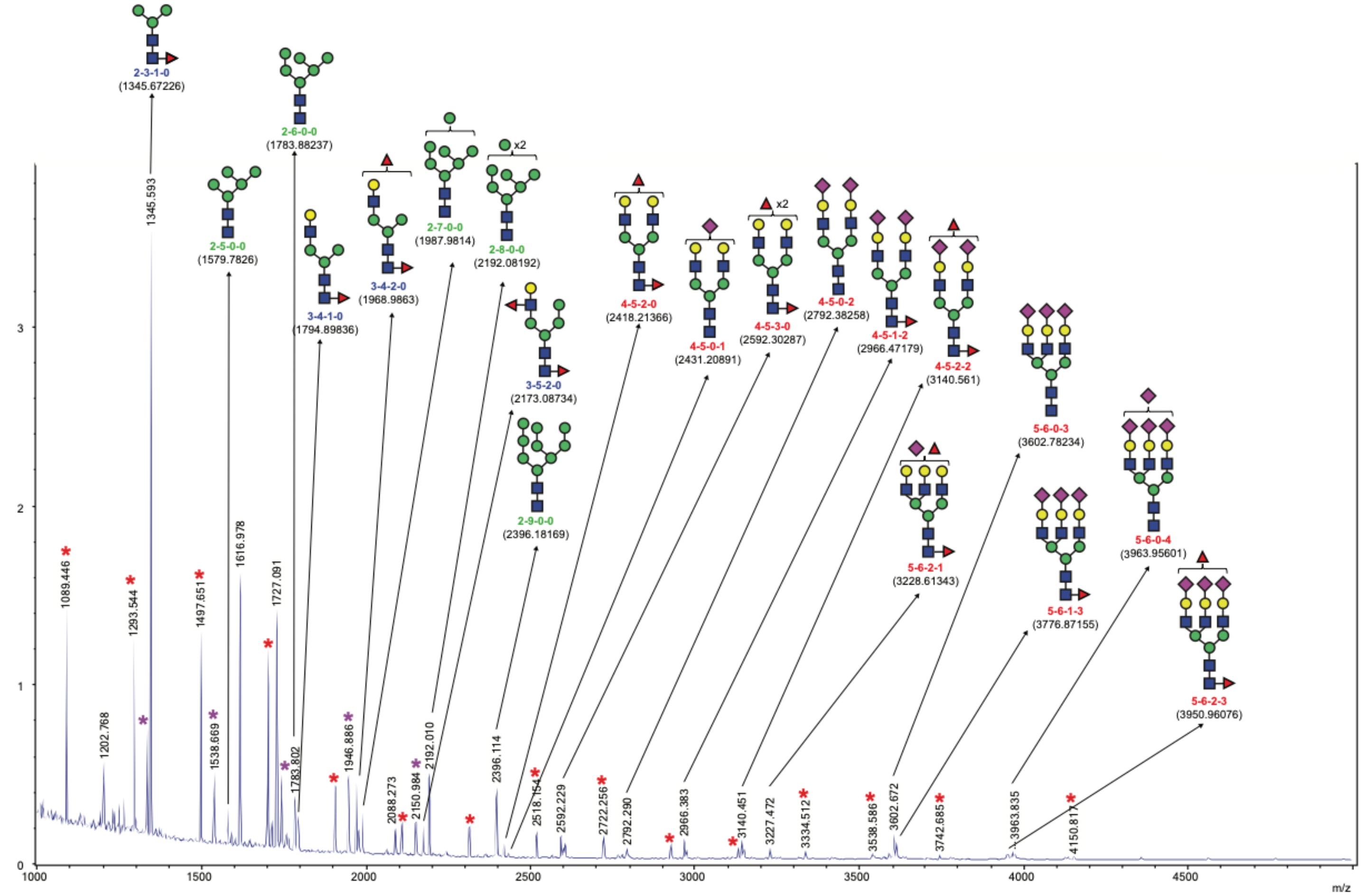

Supplemental Figure 2E. MALDI-MS (m/z 1000-5000) of PNGaseF released permethylated N-glycans from YTS-CD16a cells.

(*) represent identified cellulose contamination; (*) represent identified truncated N-glycan products. Masses represented as M+Na$^+$.

YTS-CD16a + Kif. (20 μM)

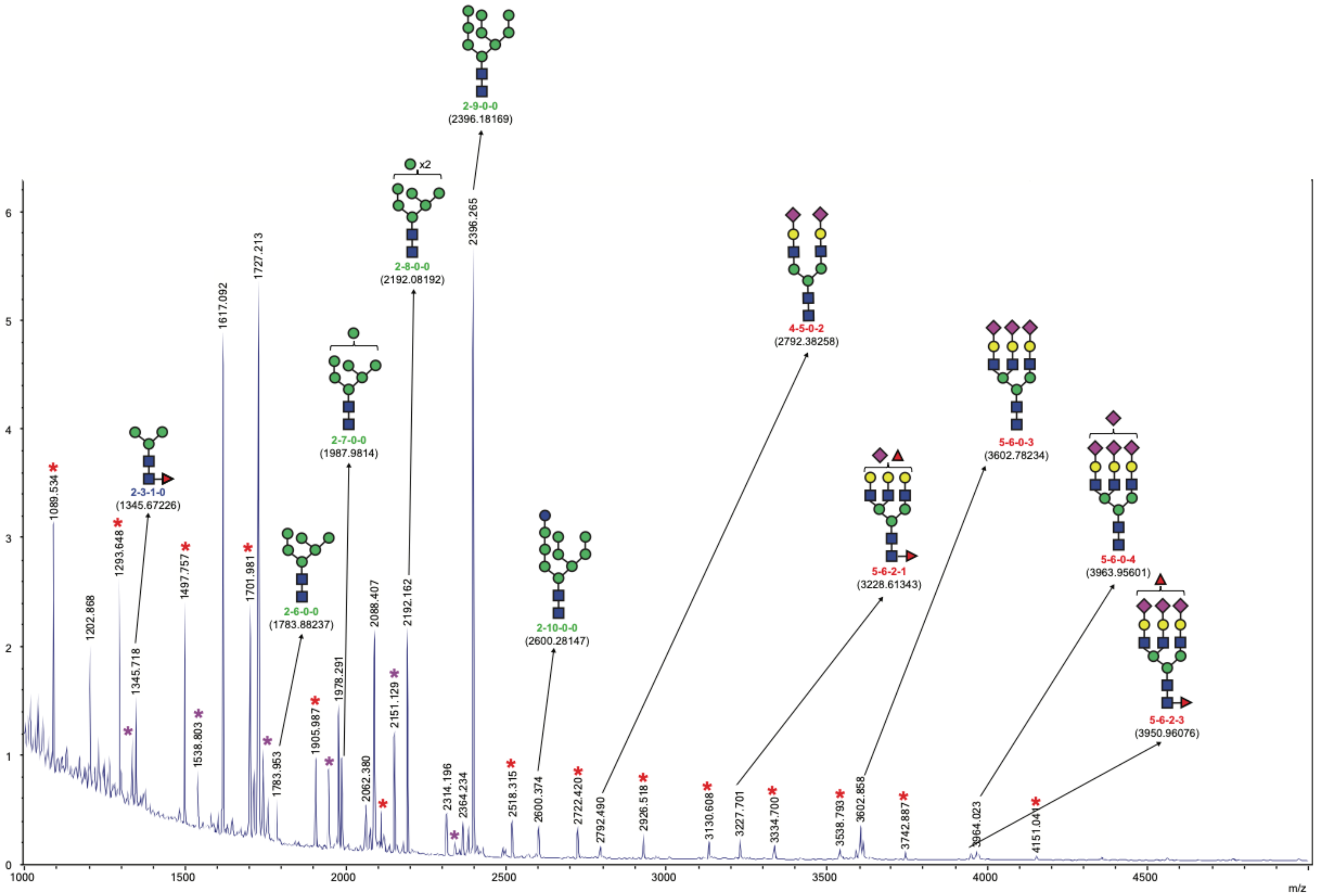

Supplemental Figure 2F. MALDI-MS (m/z 1000-5000) of PNGaseF released permethylated N-glycans from YTS-CD16a cells treated with kifunensine (Kif. 20 μM).

(*) represent identified cellulose contamination; (*) represent identified truncated N-glycan products. Masses represented as M+Na^{+}.

YTS-CD16a + Kif. (100 μM)

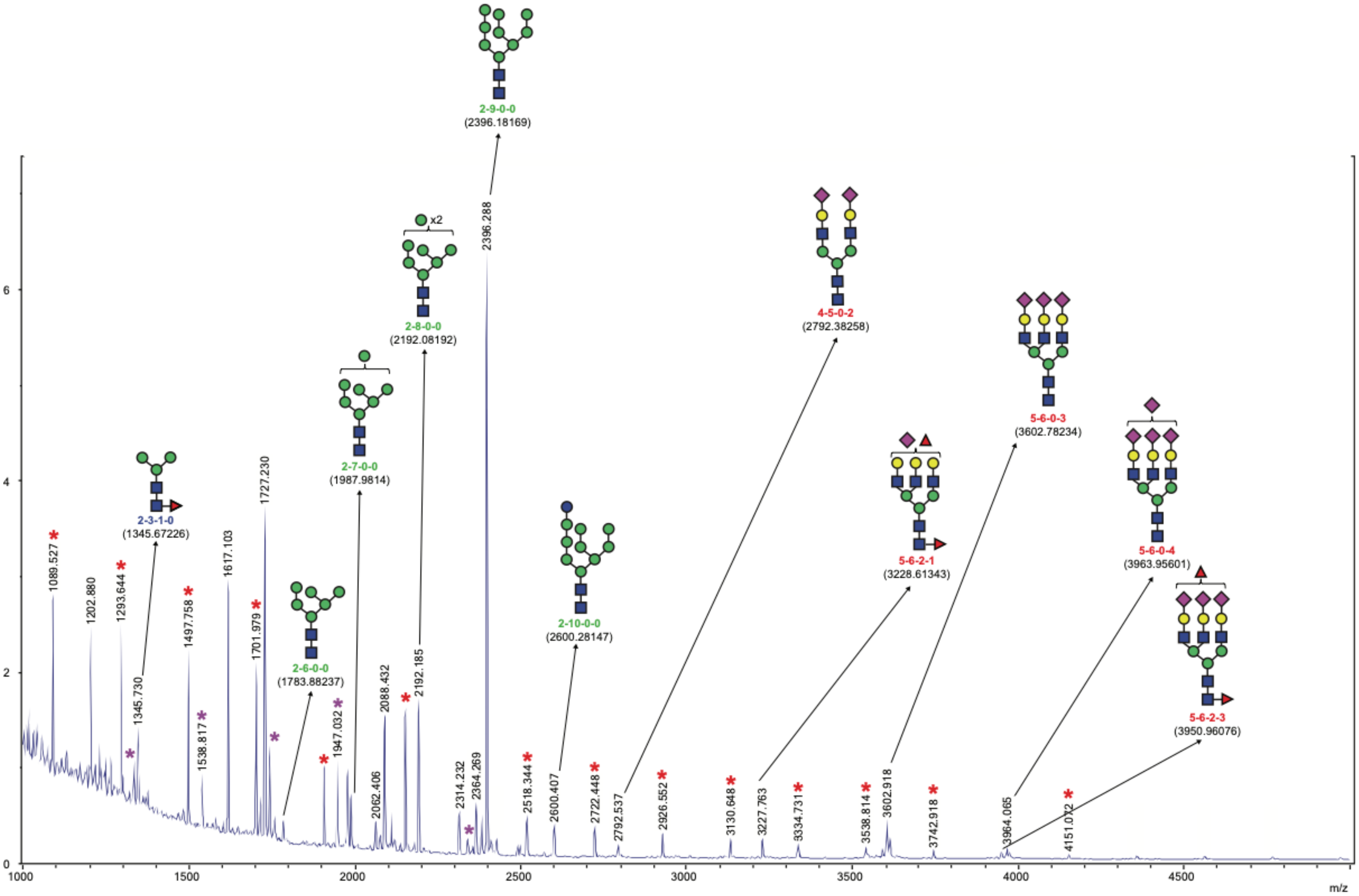

Supplemental Figure 2G. MALDI-MS (m/z 1000-5000) of PNGaseF released permethylated N-glycans from YTS-CD16a cells treated with kifunensine (Kif. 100 μM).
(*) represent identified cellulose contamination; (*) represent identified truncated N-glycan products. Masses represented as M+Na⁺.

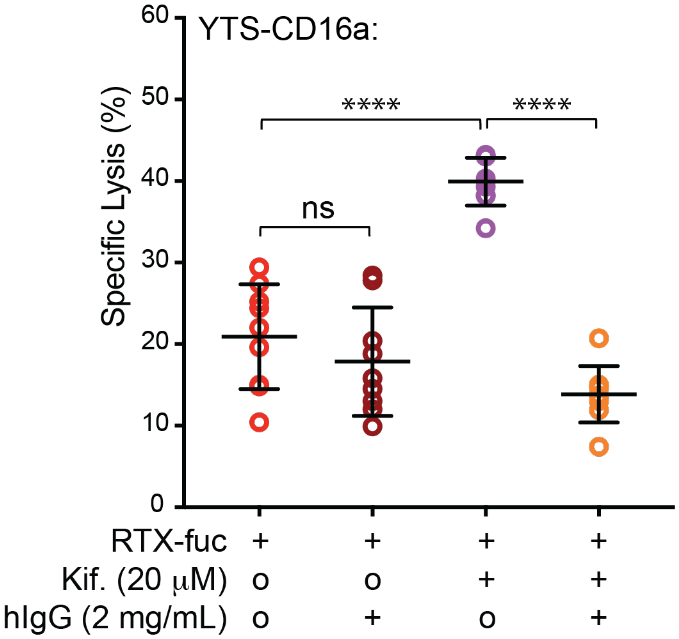

Supplemental Figure 3. ADCC elicited using afucosylated rituximab is likewise sensitive to IgG blocking.
YTS-CD16a cells show no significant decrease in ADCC with afucosylated-rituximab (RTX-fuc) blocking with hIgG (2 mg/mL) at a 1:20 target-to-effector cell ratio. Kifunensine-treated (Kif., 20 μM) YTS-CD16a cells show significant decrease in ADCC with hIgG blocking with afucosylated-rituximab (RTX-fuc). Data shown include three independent experiments collected on different days, each with three replicates. hIgG blocking (2 mg/mL) of ADCC following treatment with kifunensine (Kif., 20 μM) for primary NK cells isolated from three donors at 20:1 (effector:target) ratio. RTX - rituximab. **** $p < 0.0001$.

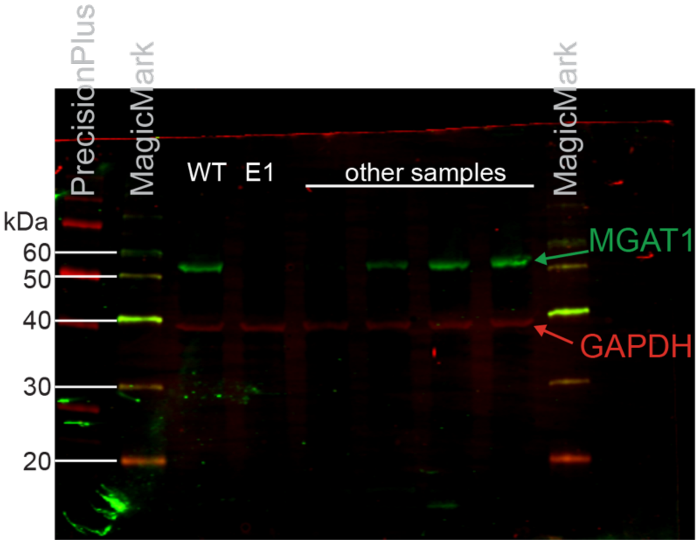

Supplemental Figure 4. The complete blot image from Figure 4C to show the ladder and other features.

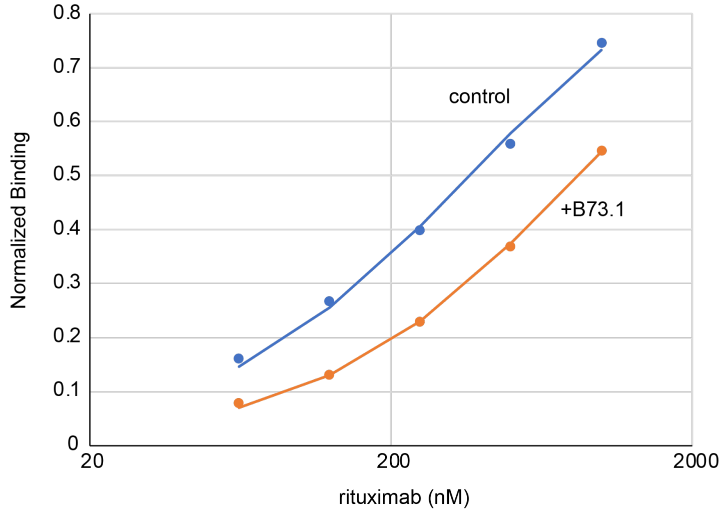

Supplemental Figure 5. Rituximab binding a CD16a-coated surface as measured by surface plasmon resonance.

Points (circles) and a fitted line (solid line) are shown. Rituximab bound CD16a with an affinity of 360 ±40 nM. Following preincubation of 100 nM B73.1 with the CD16a-coated surface for 500s, rituximab bound with an affinity of 840 ±60 nM.

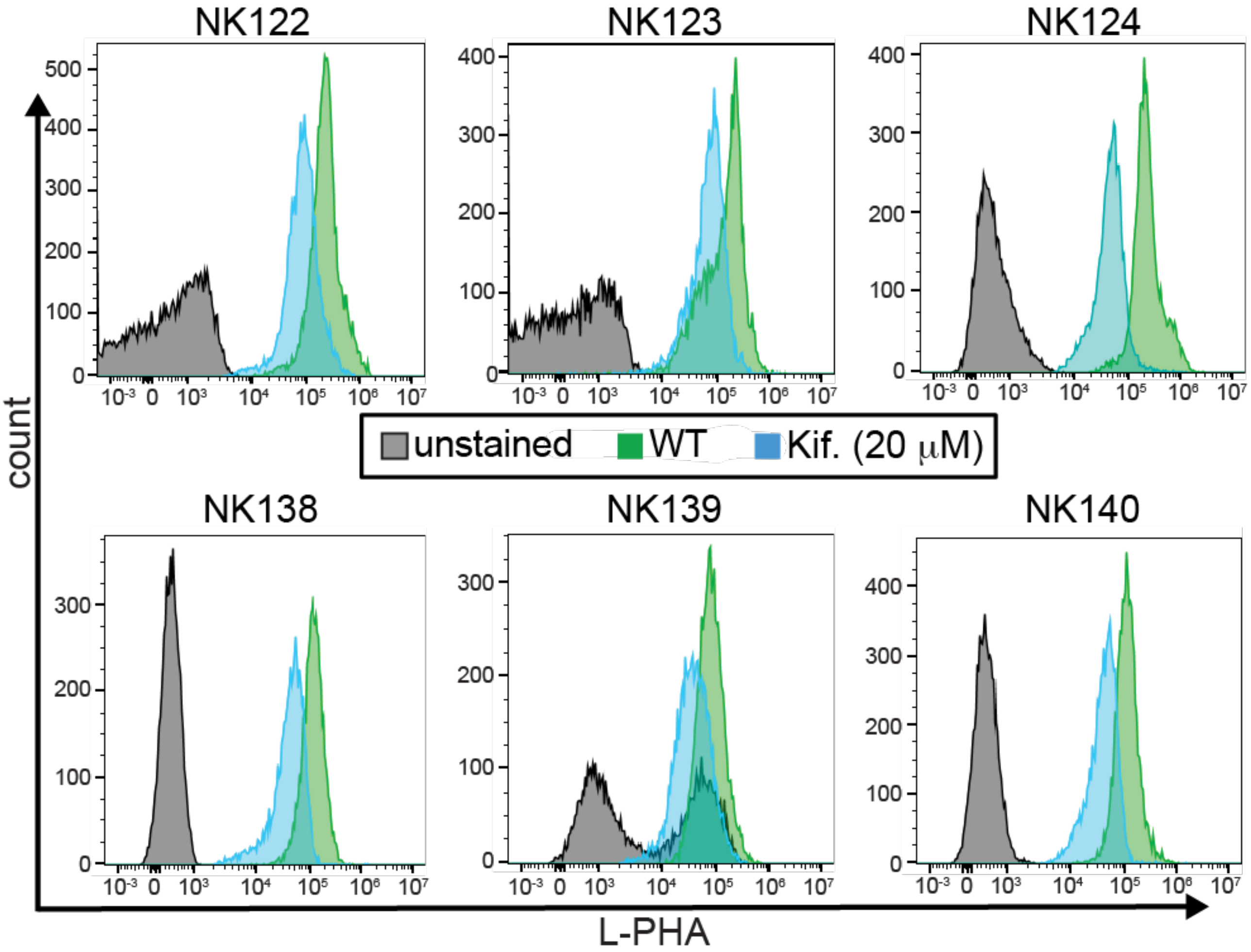

Supplemental Figure 6. Representative flow cytometry plots.
Primary NK cell donors (NK122, NK123, NK124, NK138, NK139, NK140) showing decreased L-PHA staining for kifunensine (“Kif.”, 20 μM) cells.

Supplemental Figure 7. The following panels show MS1 and MS2 spectra for the permethylated N-glycans identified by ESI-MS/MS. Each panel shows an individual species, with the cell source denoted in the upper left of the panel, a cartoon diagram showing the composition of each glycan using the SNFG nomenclature, the MS spectrum in the upper right inset, and the main panel showing the MS2 spectrum with annotations of each ion. There are 116 individual assigned MS spectra.

MS1 and MS2 for YTS-CD16 (WT) N-glycoforms.

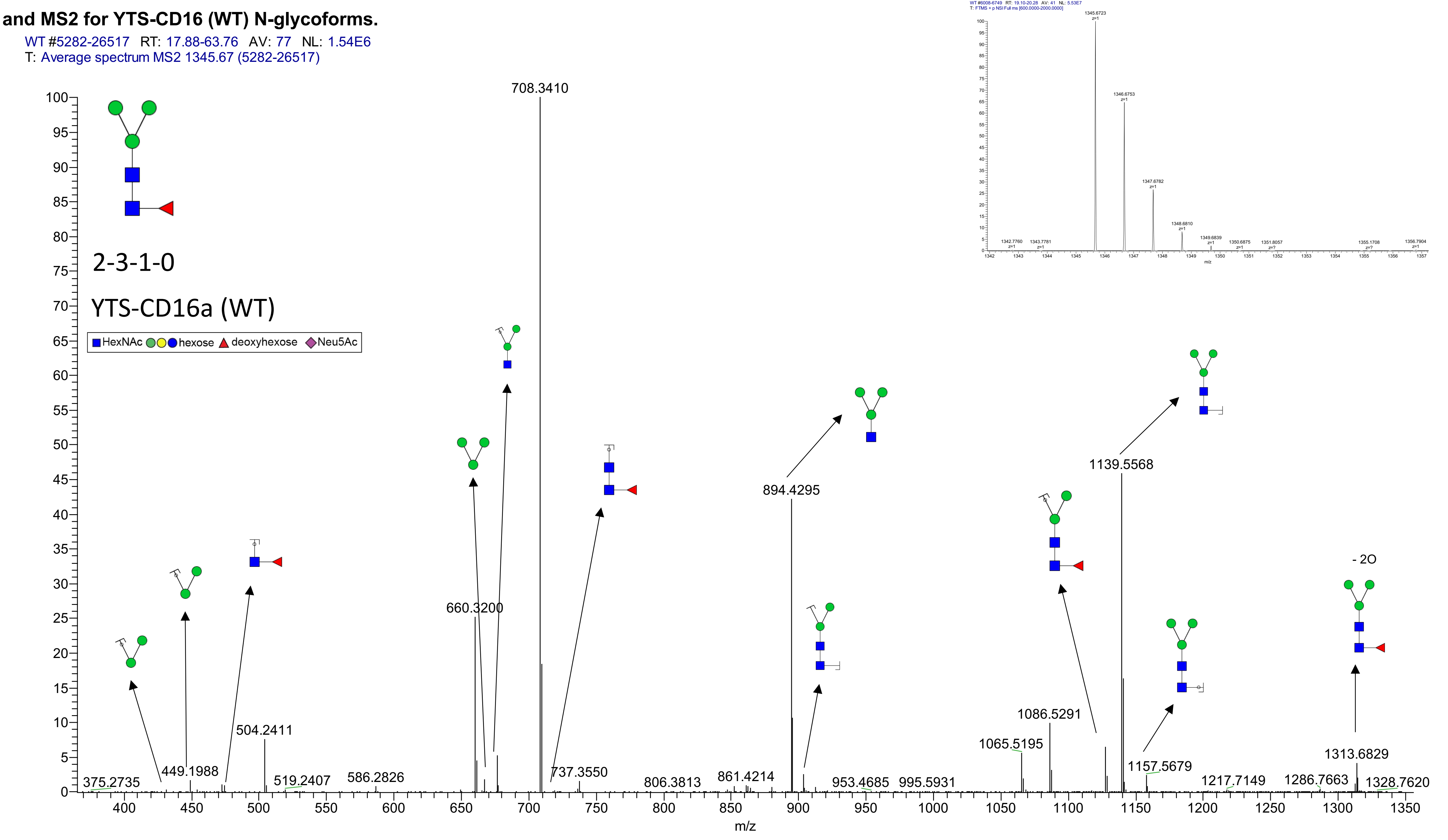

MS1 and MS2 for YTS-CD16 (WT) N-glycoforms.

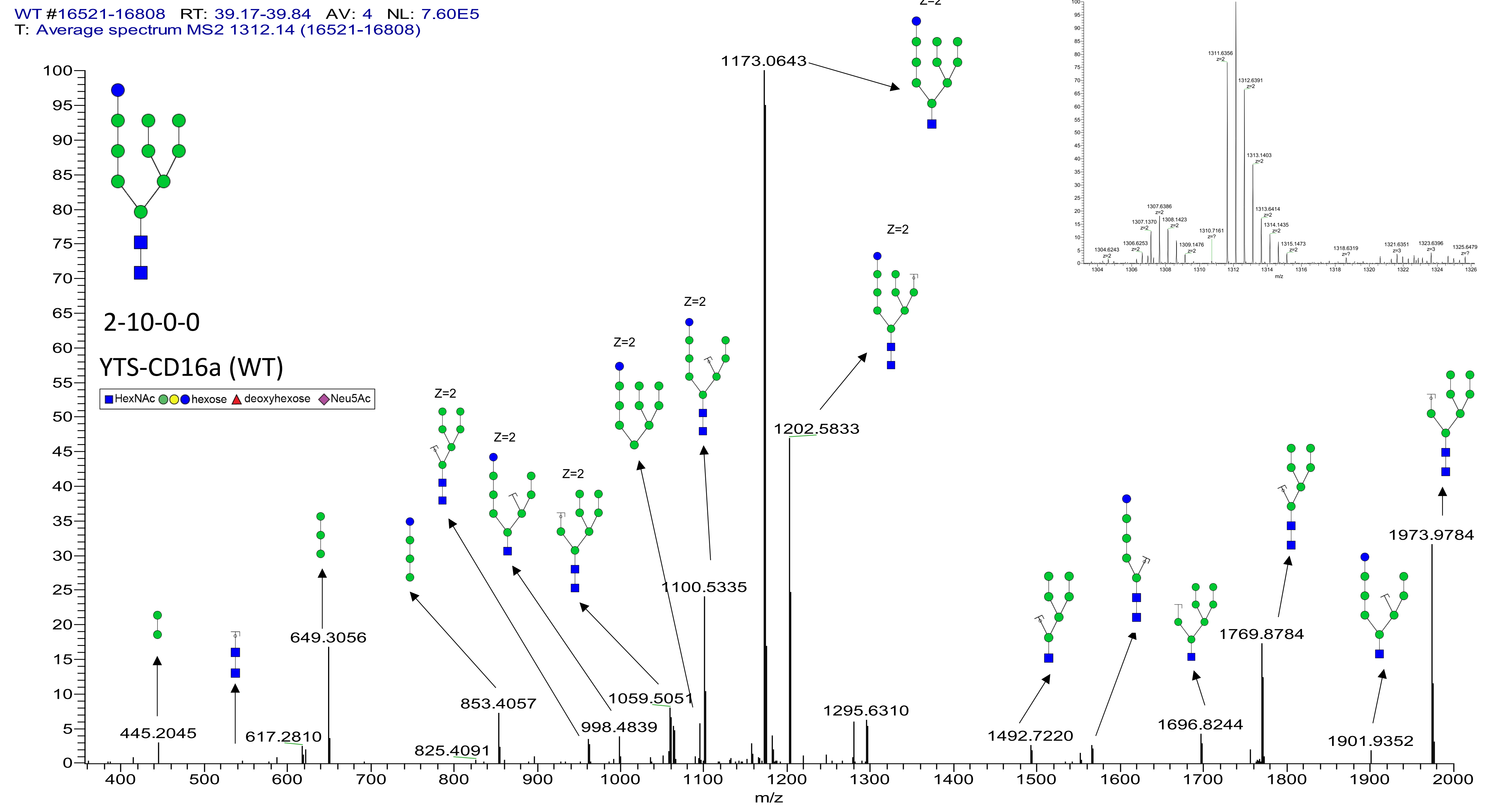

MS1 and MS2 for YTS-CD16 (WT) N-glycoforms.

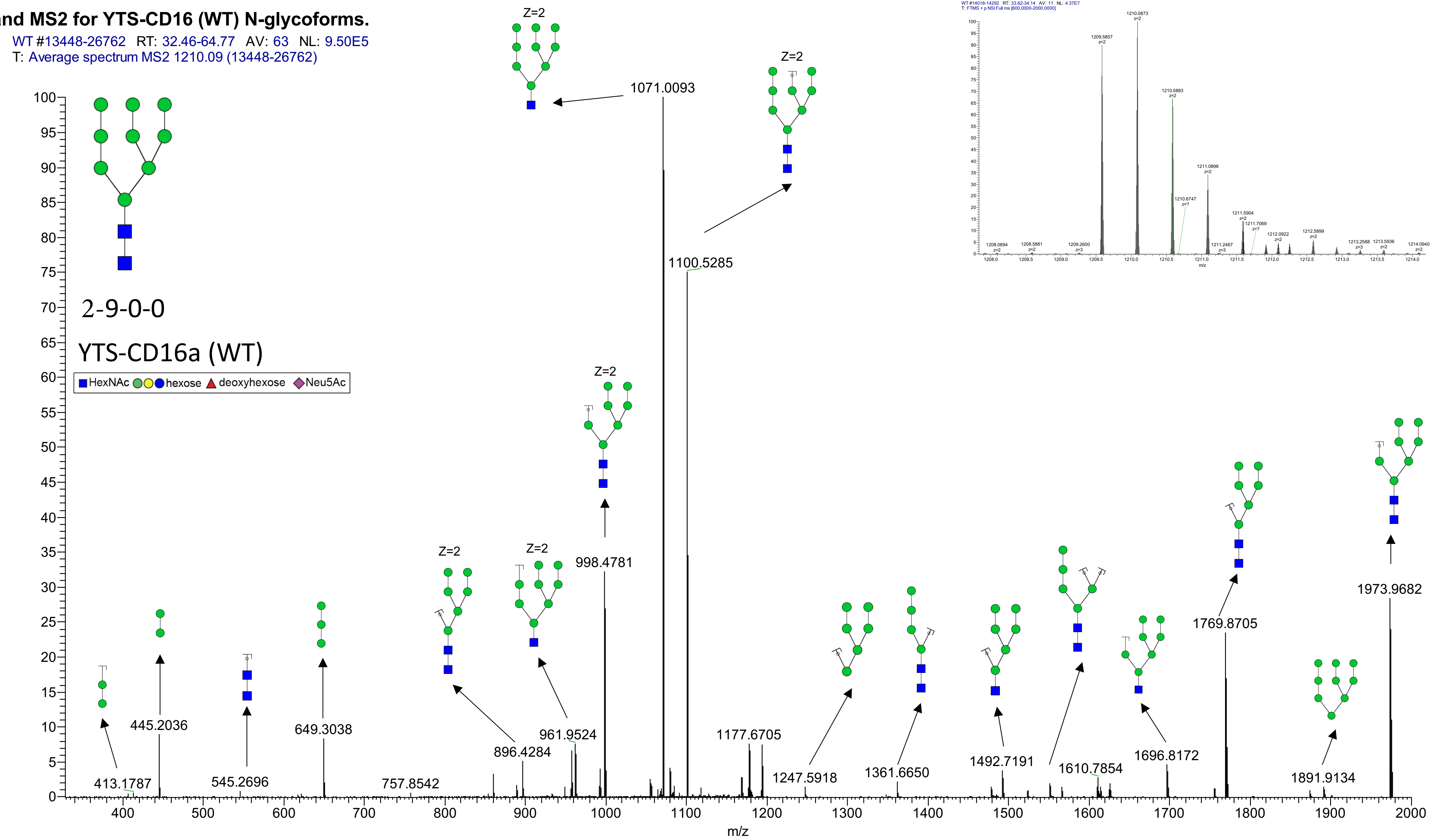

MS1 and MS2 for YTS-CD16 (WT) N-glycoforms.

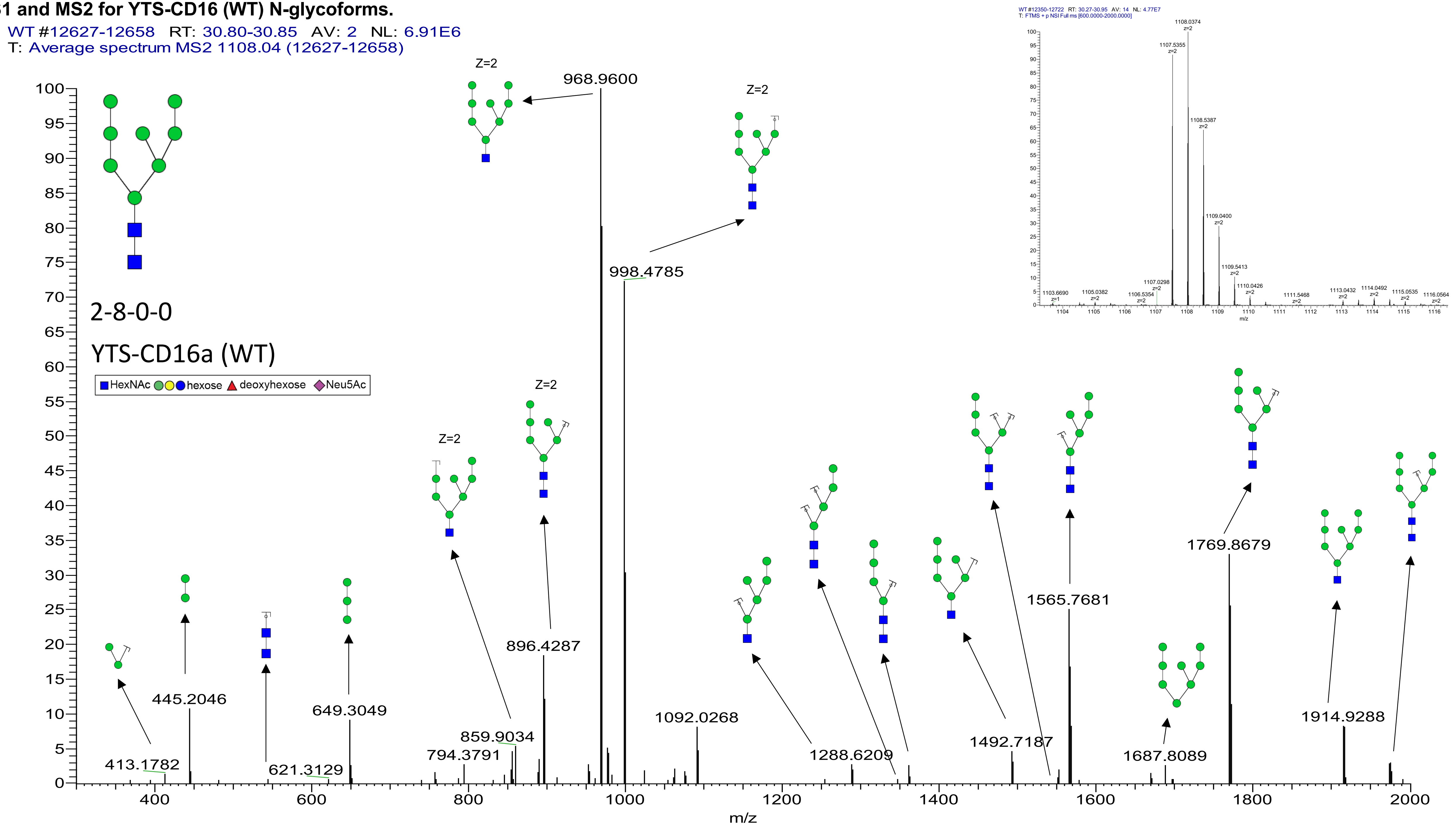

MS1 and MS2 for YTS-CD16 (WT) N-glycoforms.

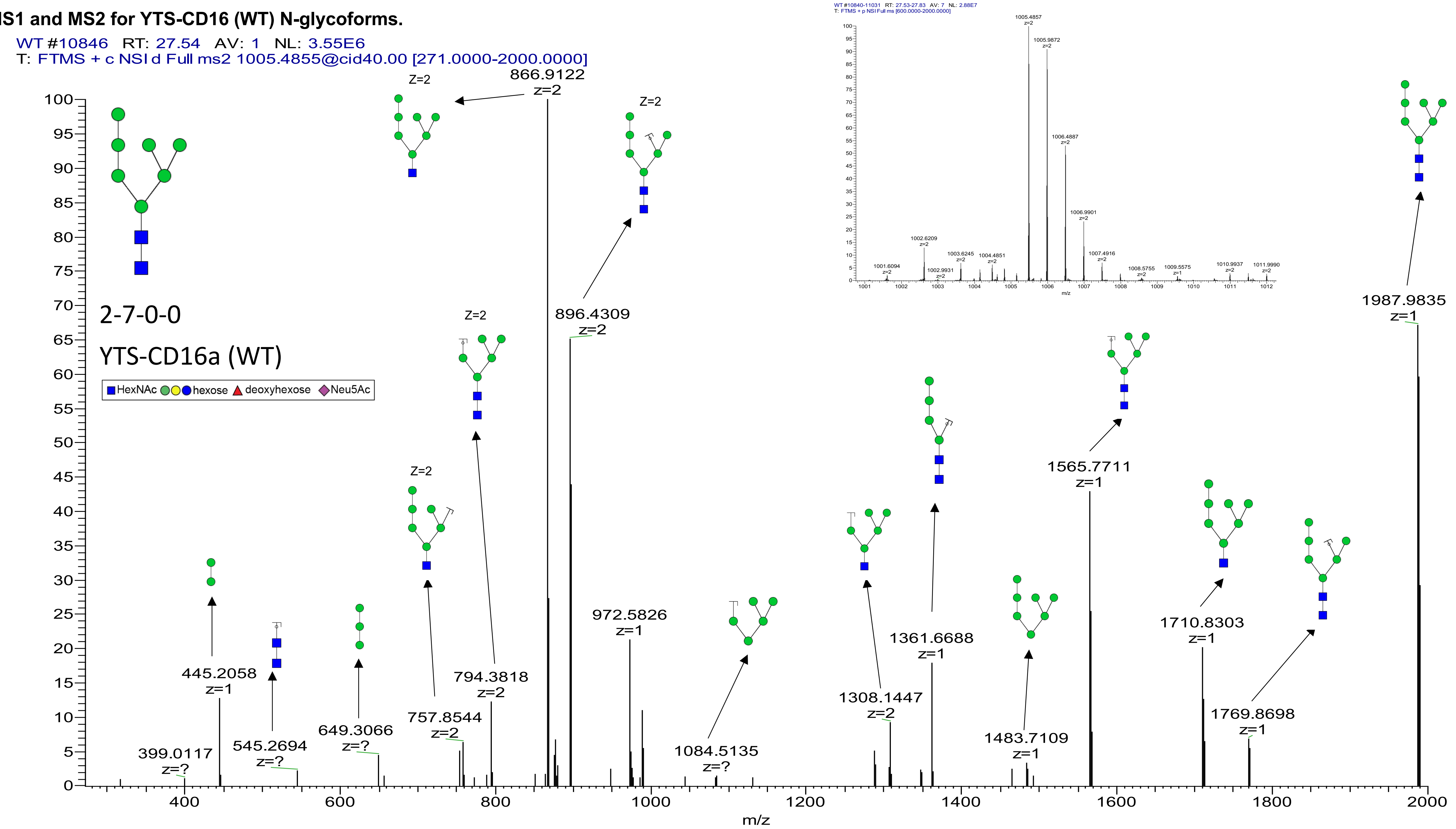

MS1 and MS2 for YTS-CD16 (WT) N-glycoforms.

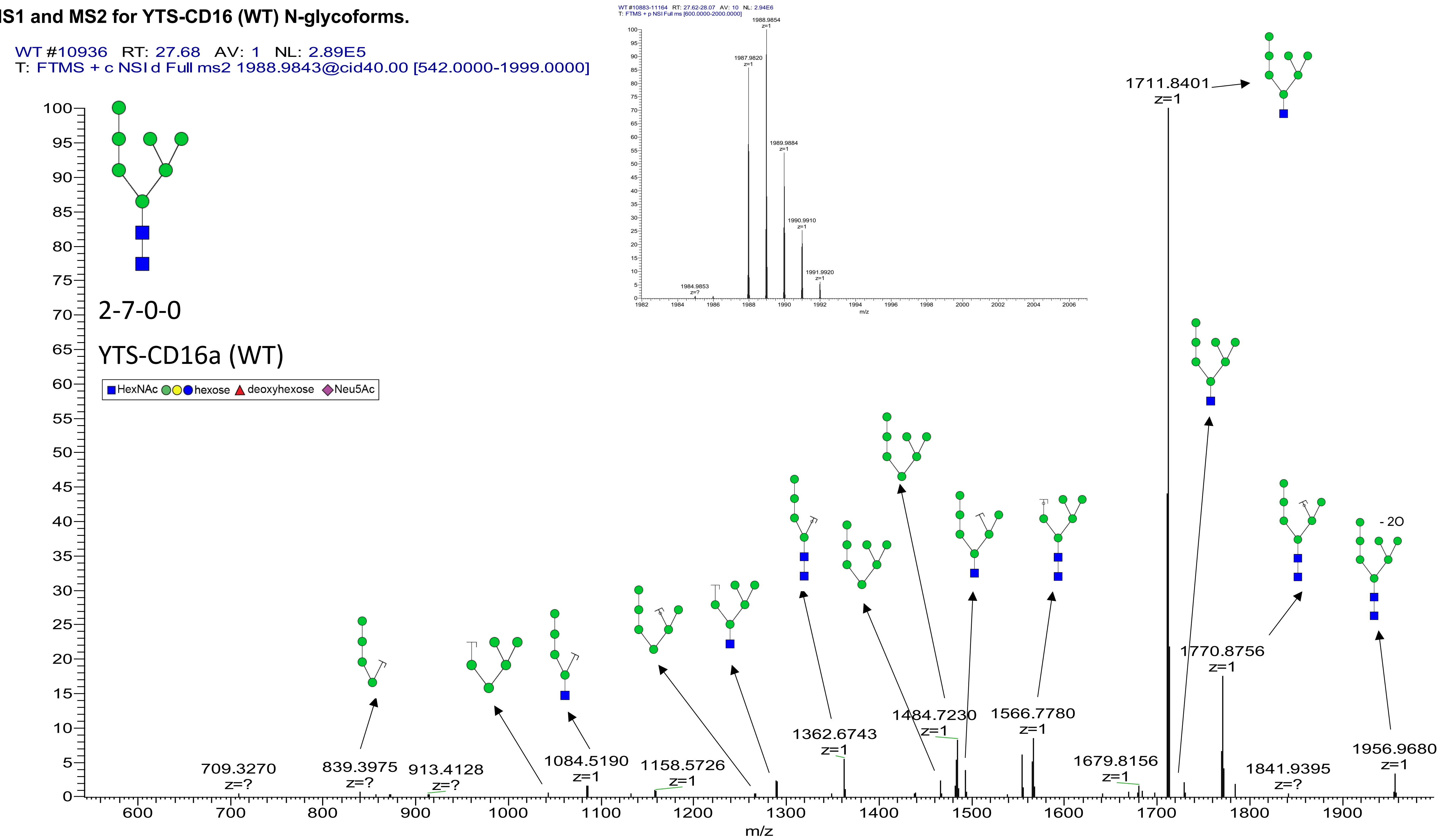

MS1 and MS2 for YTS-CD16 (WT) N-glycoforms.

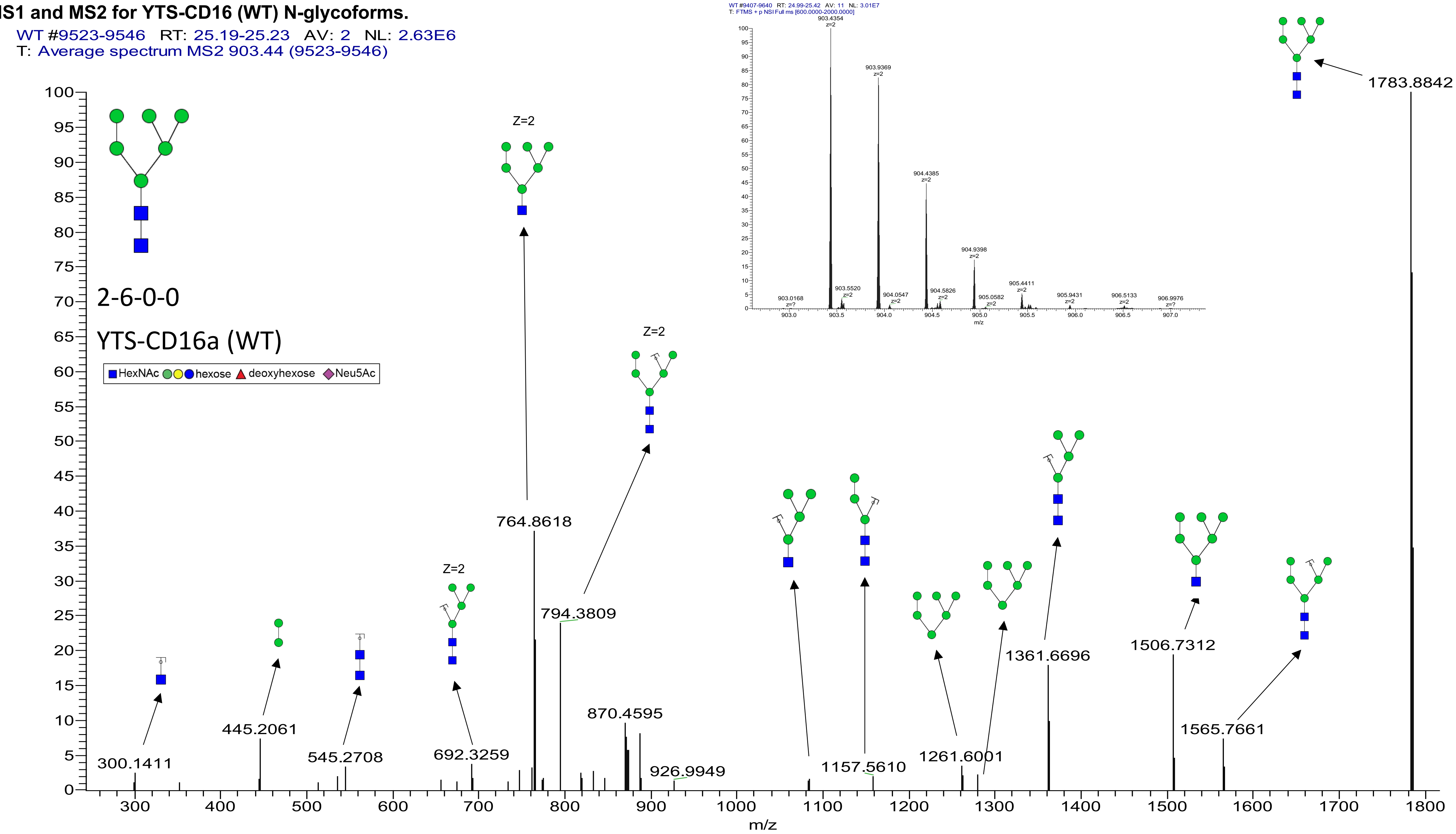

MS1 and MS2 for YTS-CD16 (WT) N-glycoforms.

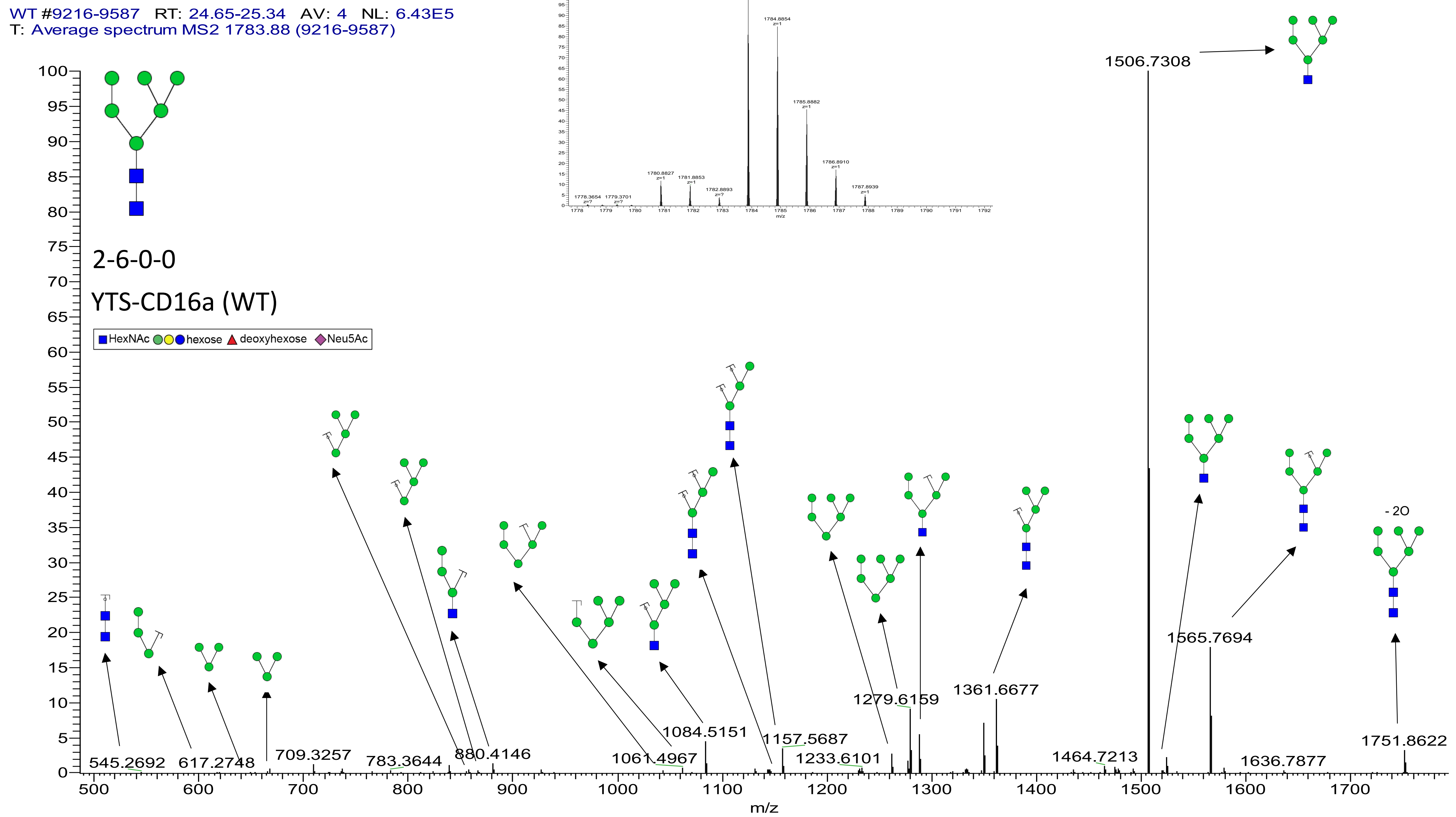

MS1 and MS2 for YTS-CD16 (WT) N-glycoforms.

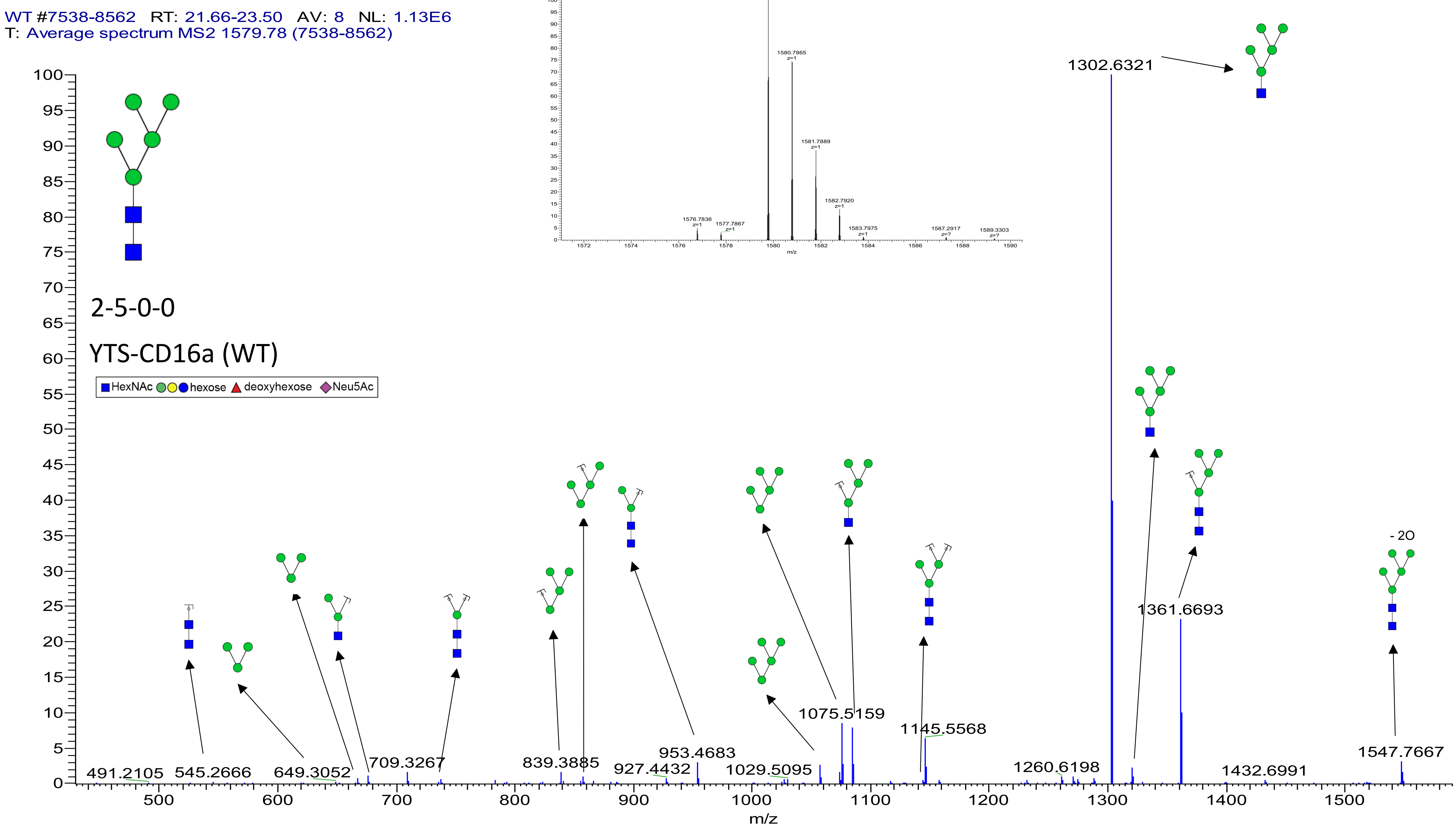

MS1 and MS2 for YTS-CD16 (WT) N-glycoforms.

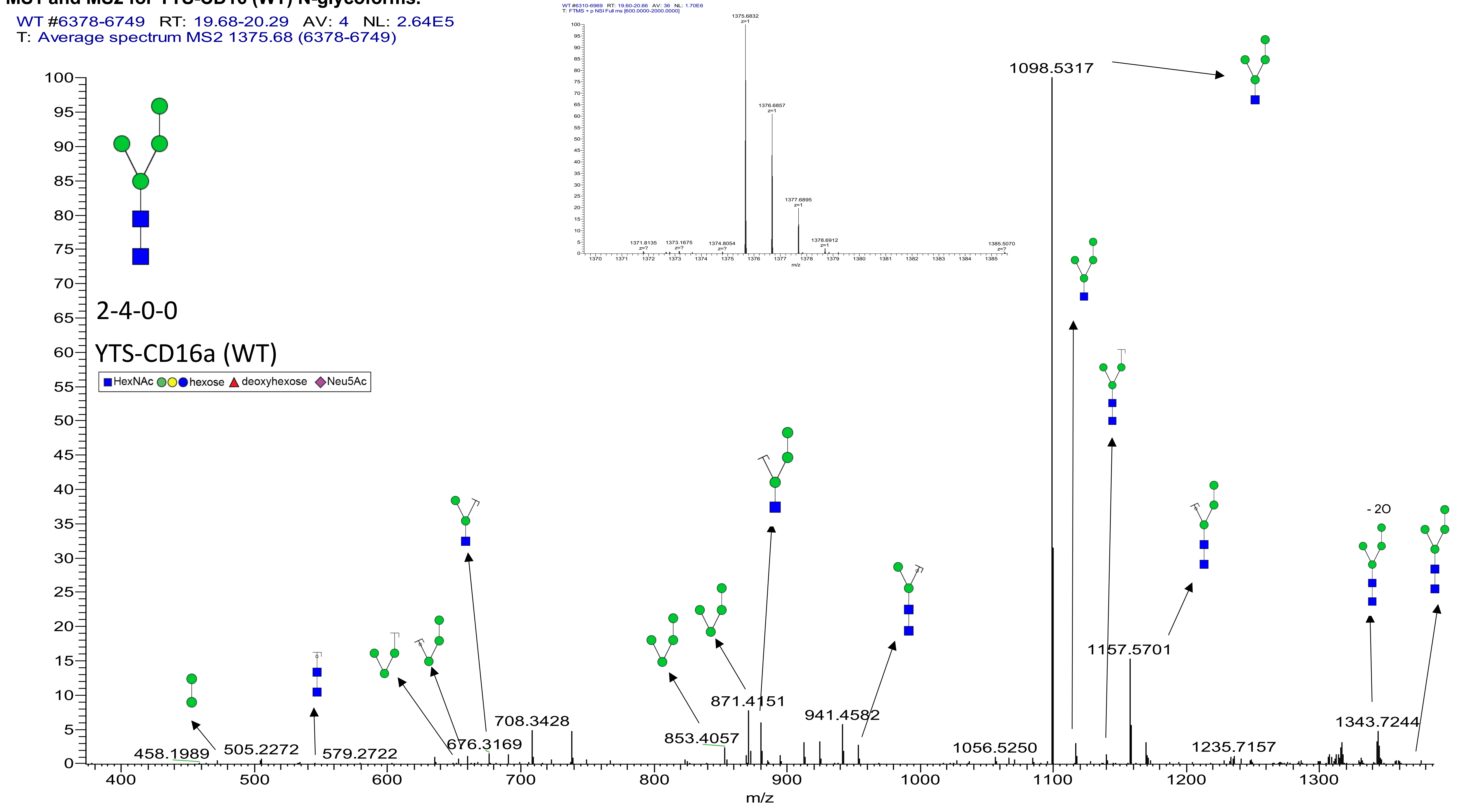

MS1 and MS2 for YTS-CD16 (WT) N-glycoforms.

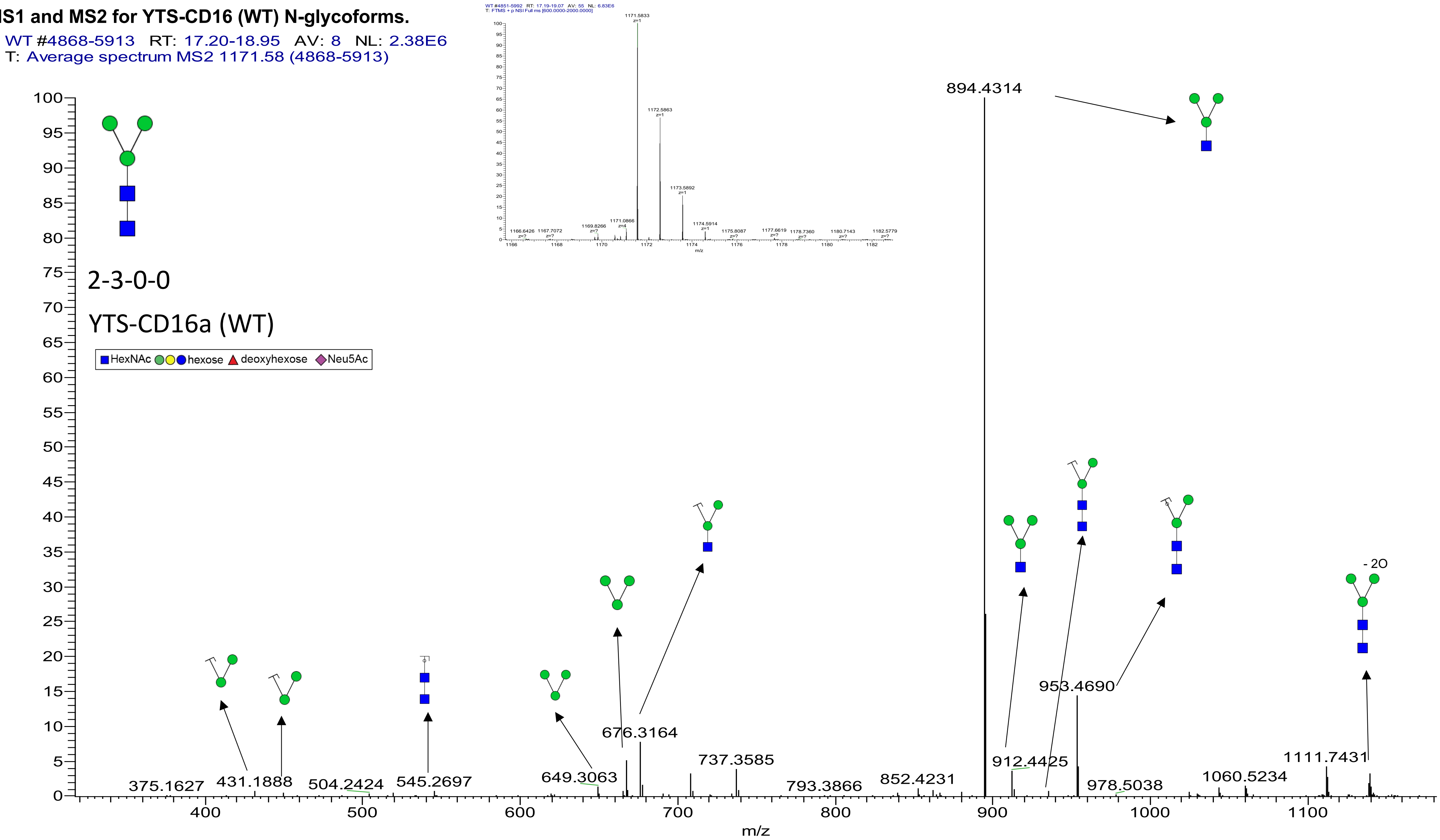

MS1 and MS2 for YTS-CD16 (WT) N-glycoforms.

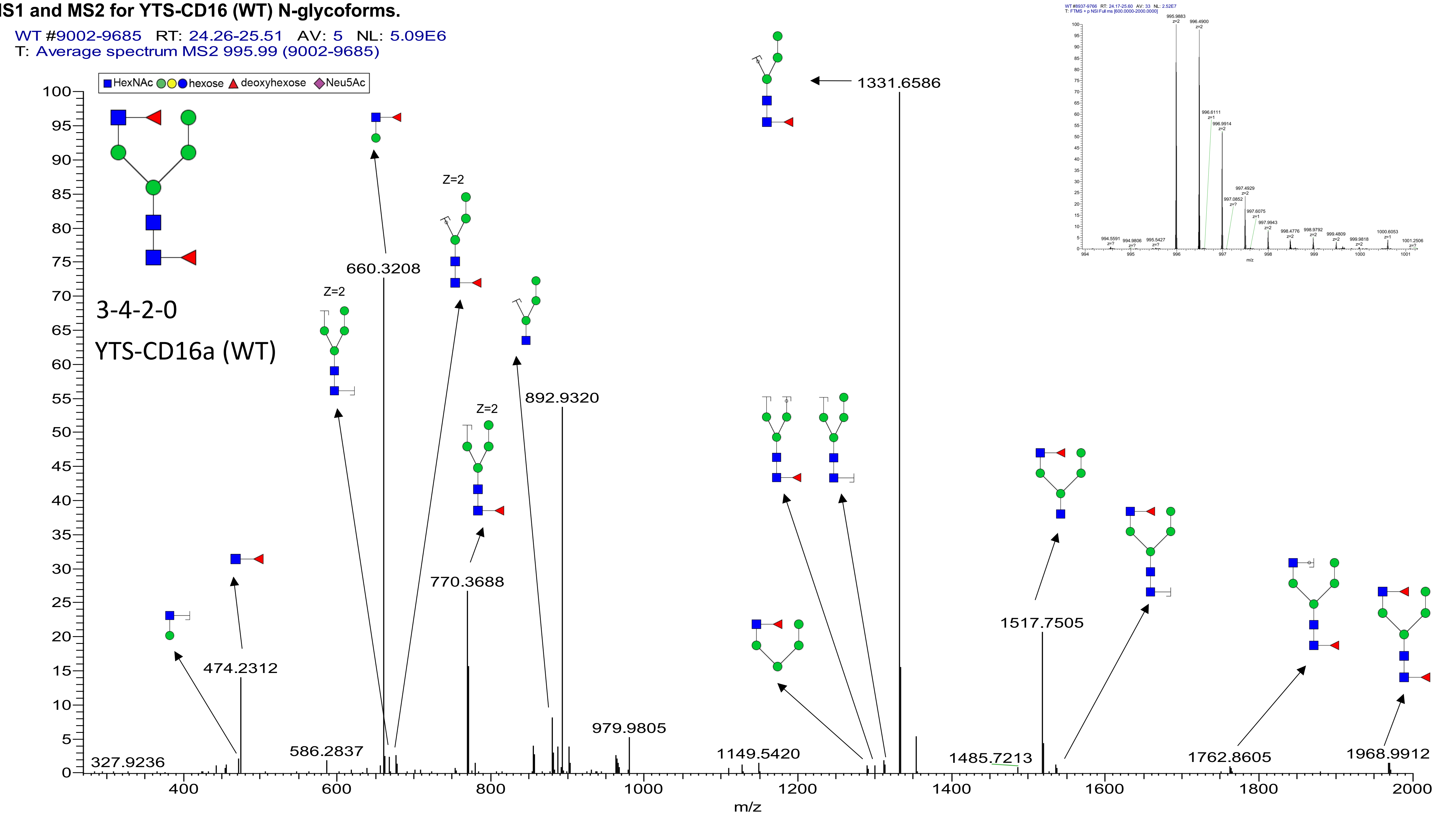

MS1 and MS2 for YTS-CD16 (WT) N-glycoforms.

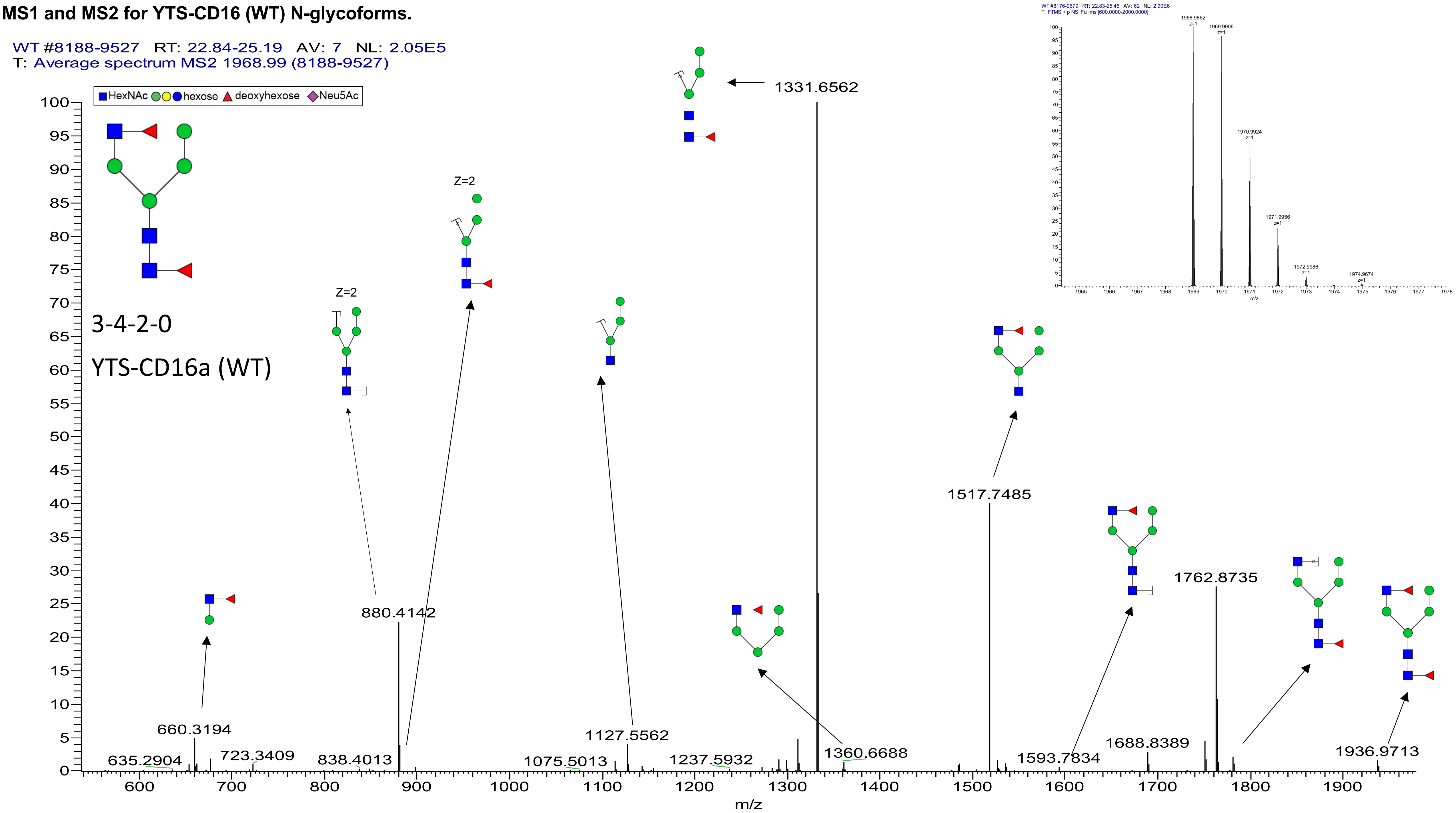

MS1 and MS2 for YTS-CD16 (WT) N-glycoforms.

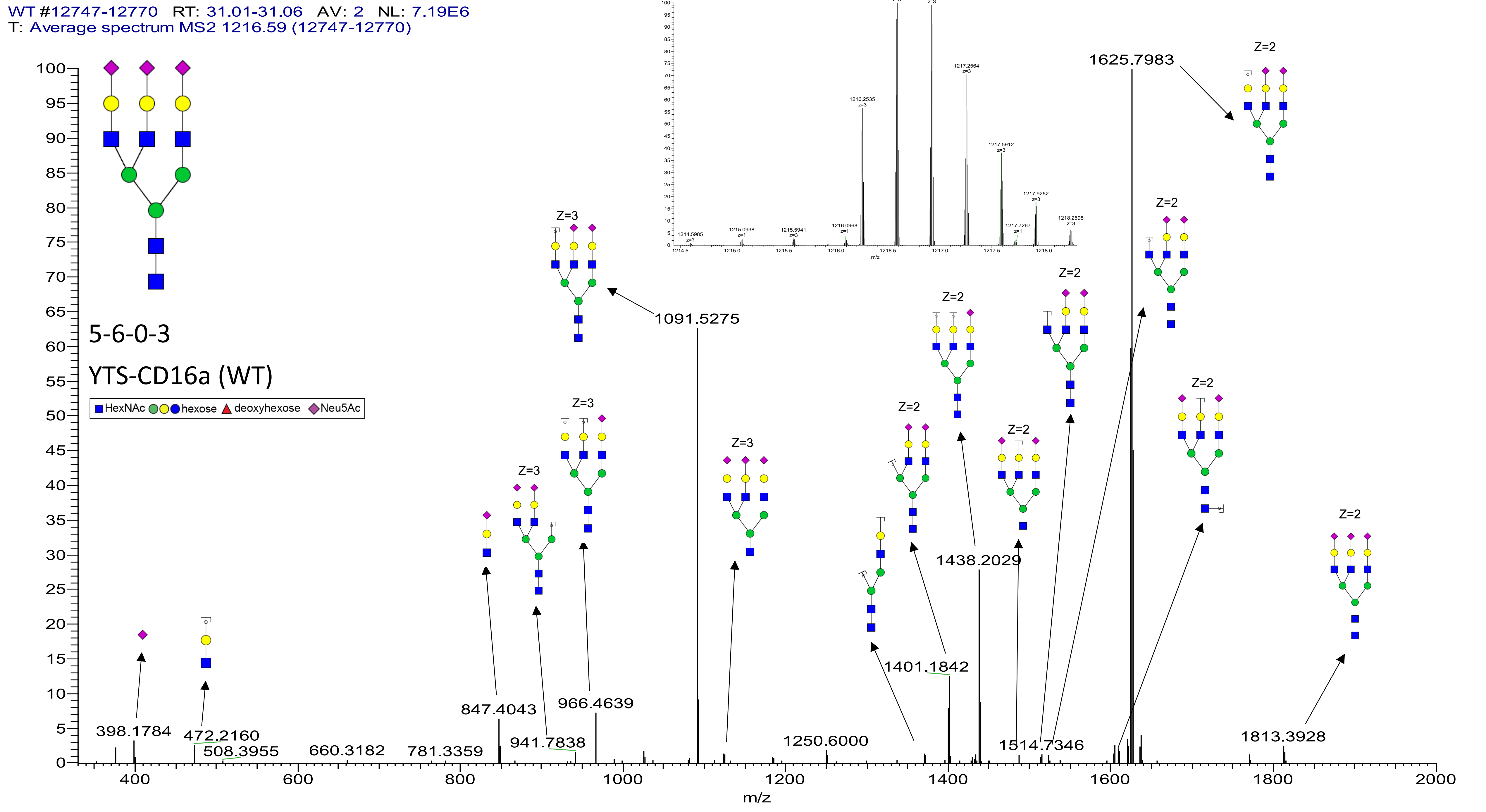

MS1 and MS2 for YTS-CD16 (WT) N-glycoforms.

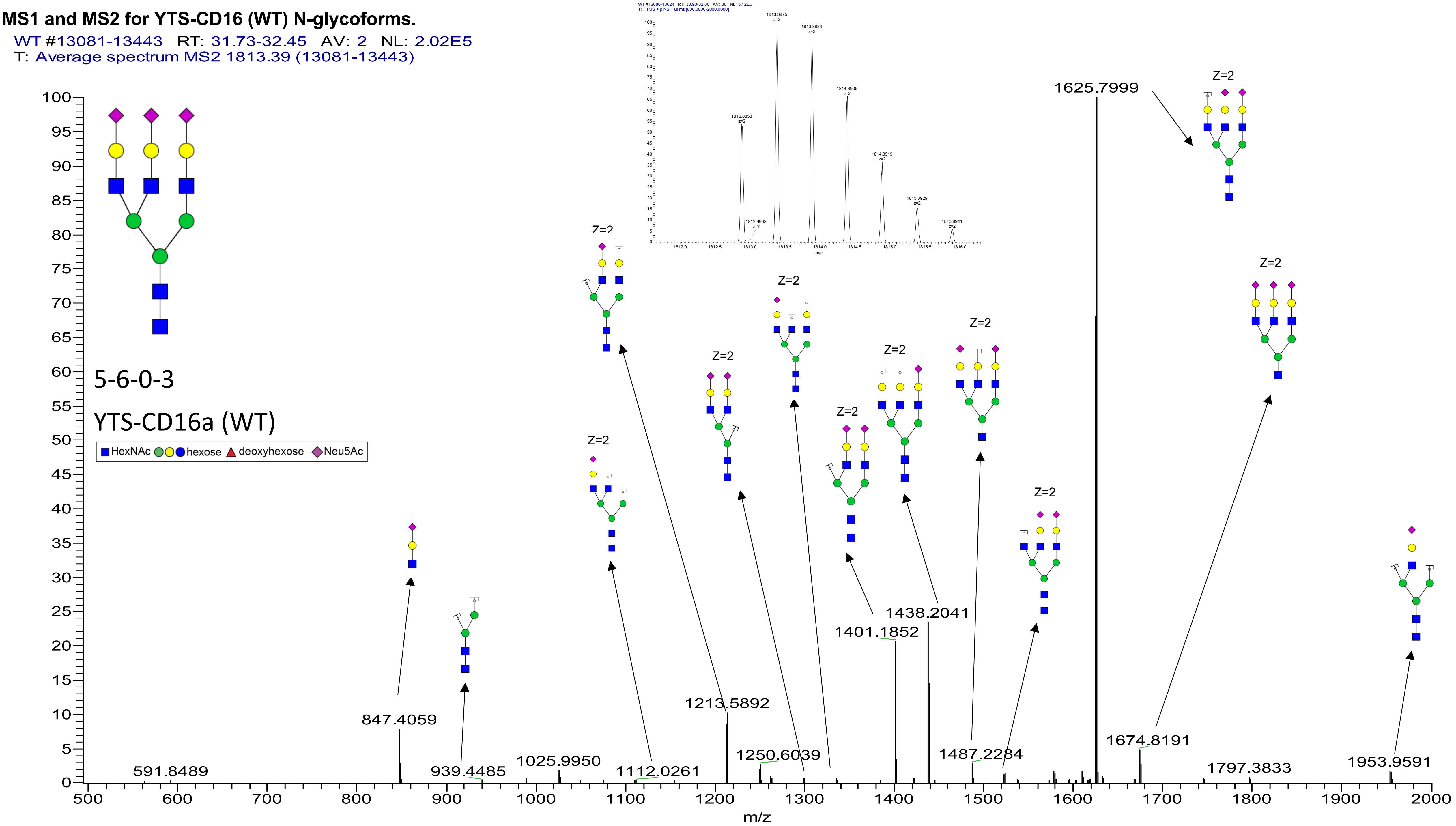

MS1 and MS2 for YTS-CD16 (WT) N-glycoforms.

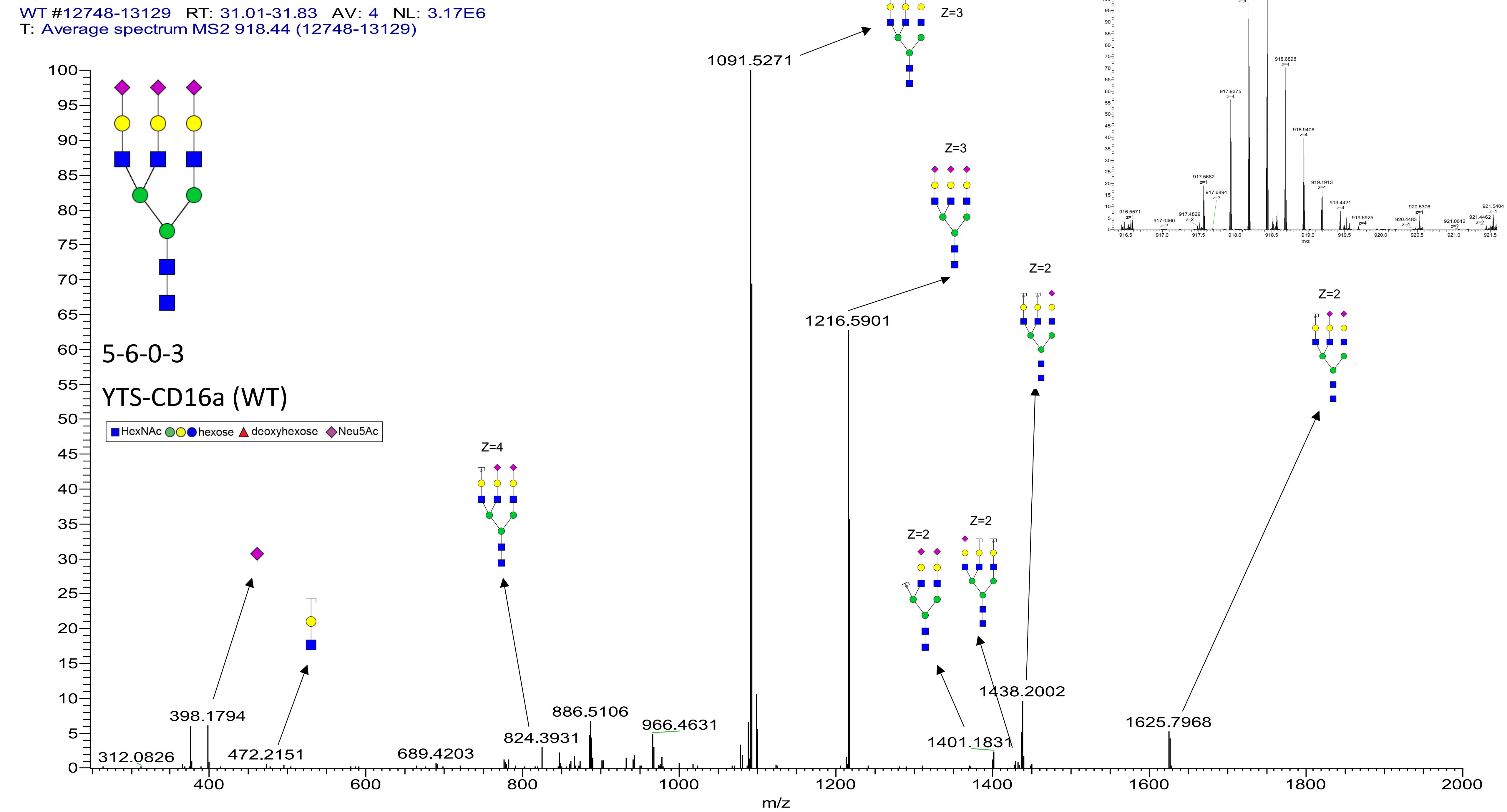

MS1 and MS2 for YTS-CD16 (WT) N-glycoforms.

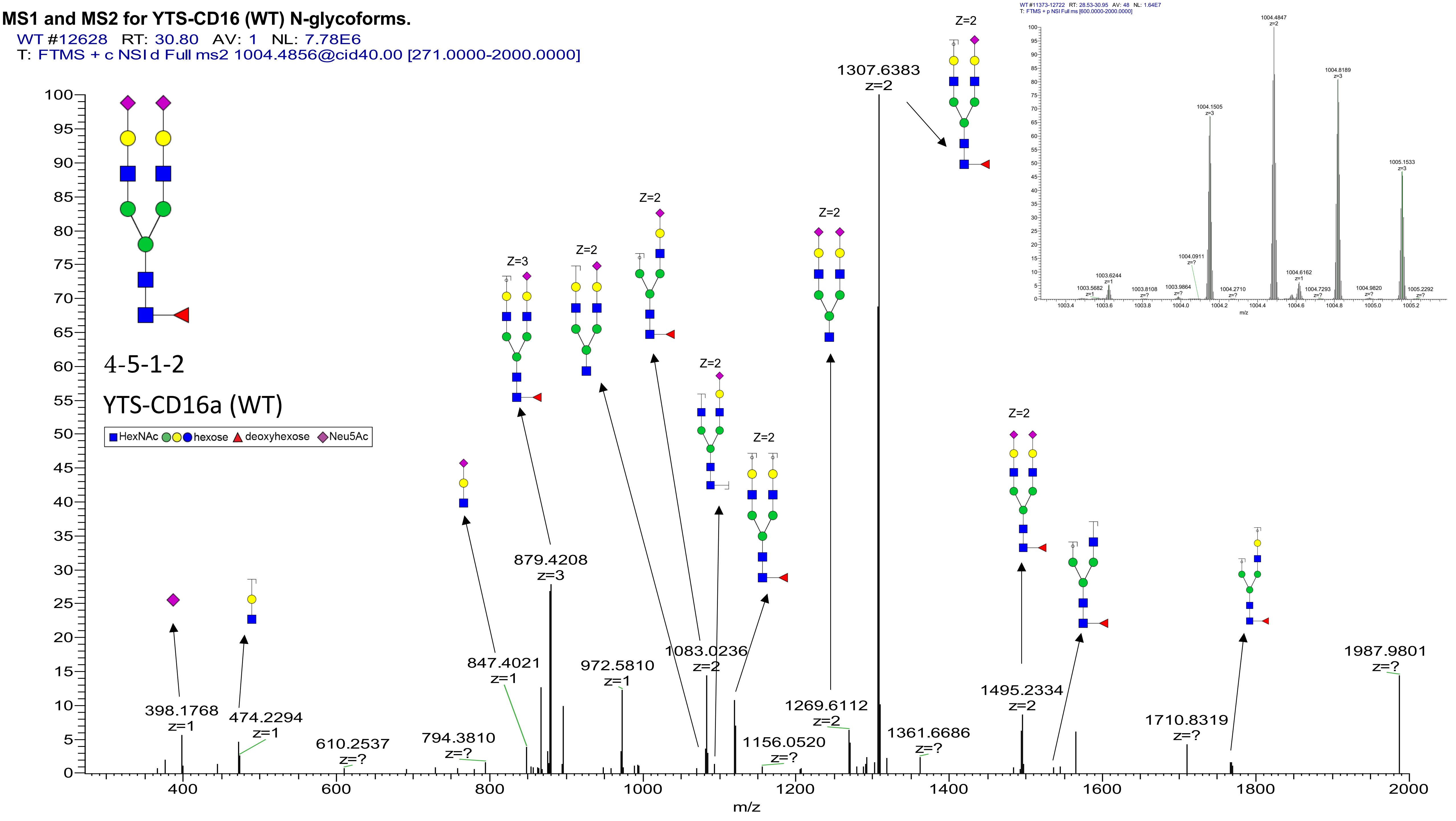

MS1 and MS2 for YTS-CD16 (WT) N-glycoforms.

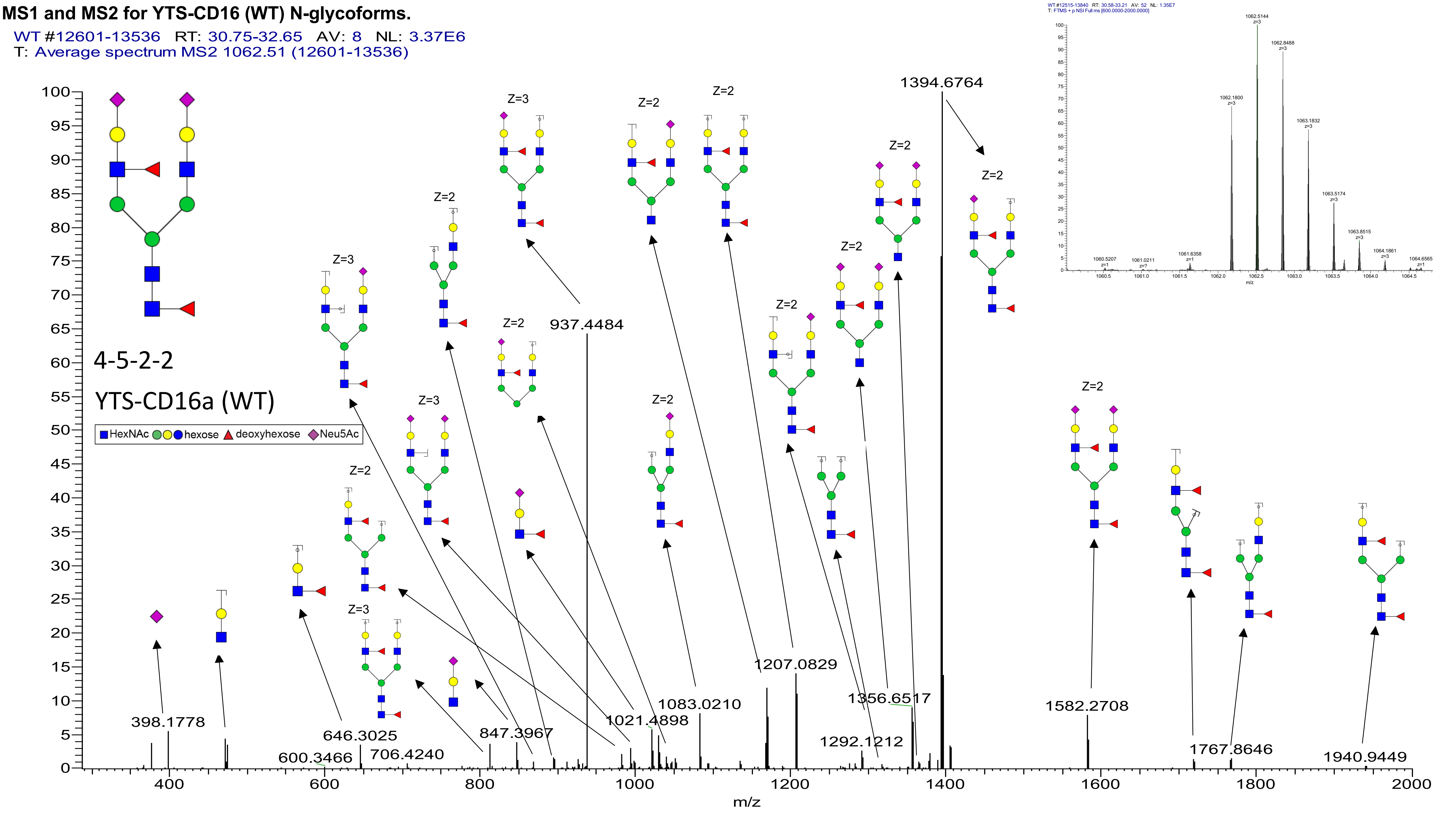

MS1 and MS2 for YTS-CD16 (WT) N-glycoforms.

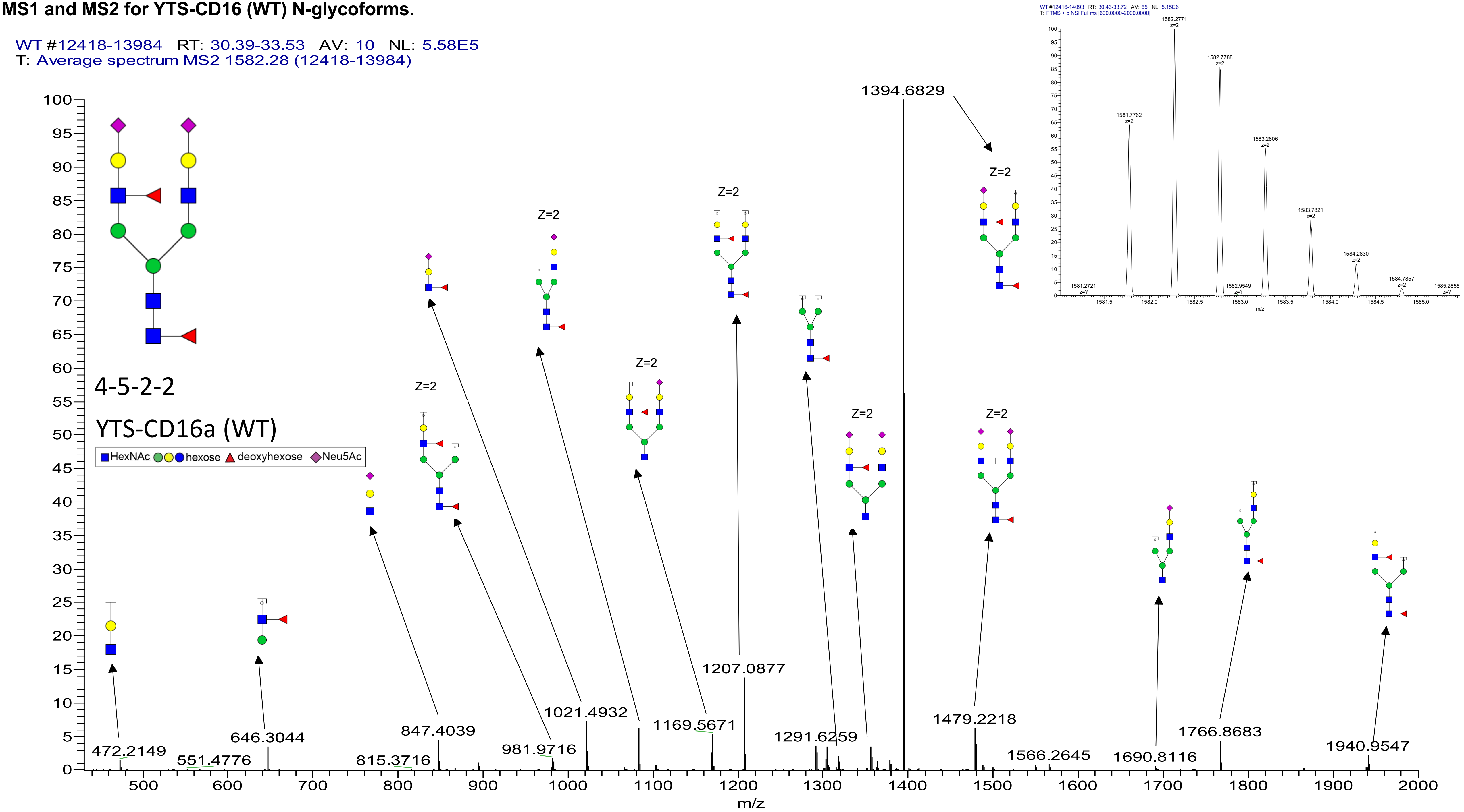

MS1 and MS2 for YTS-CD16 (WT) N-glycoforms.

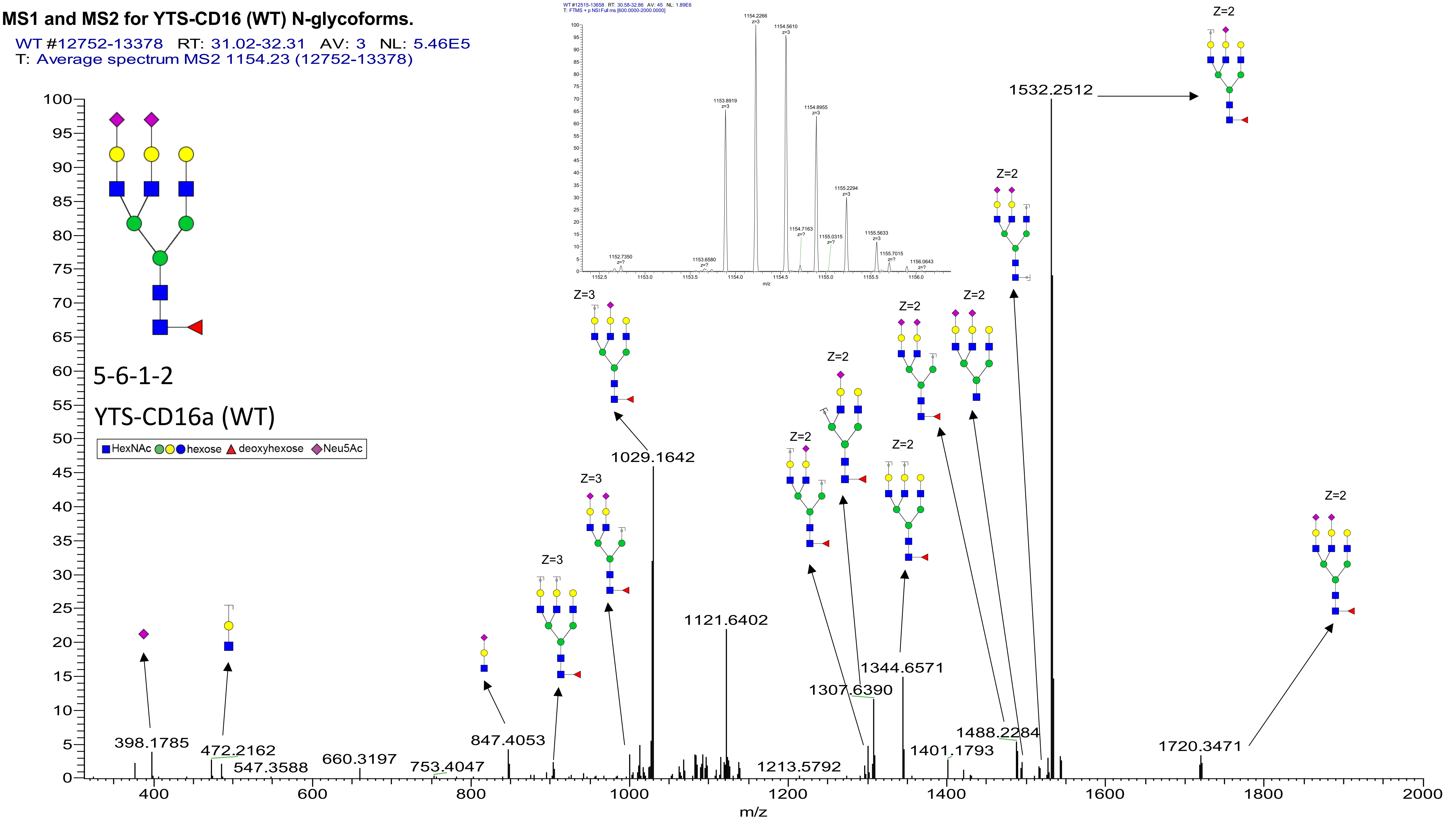

MS1 and MS2 for YTS-CD16 (WT) N-glycoforms.

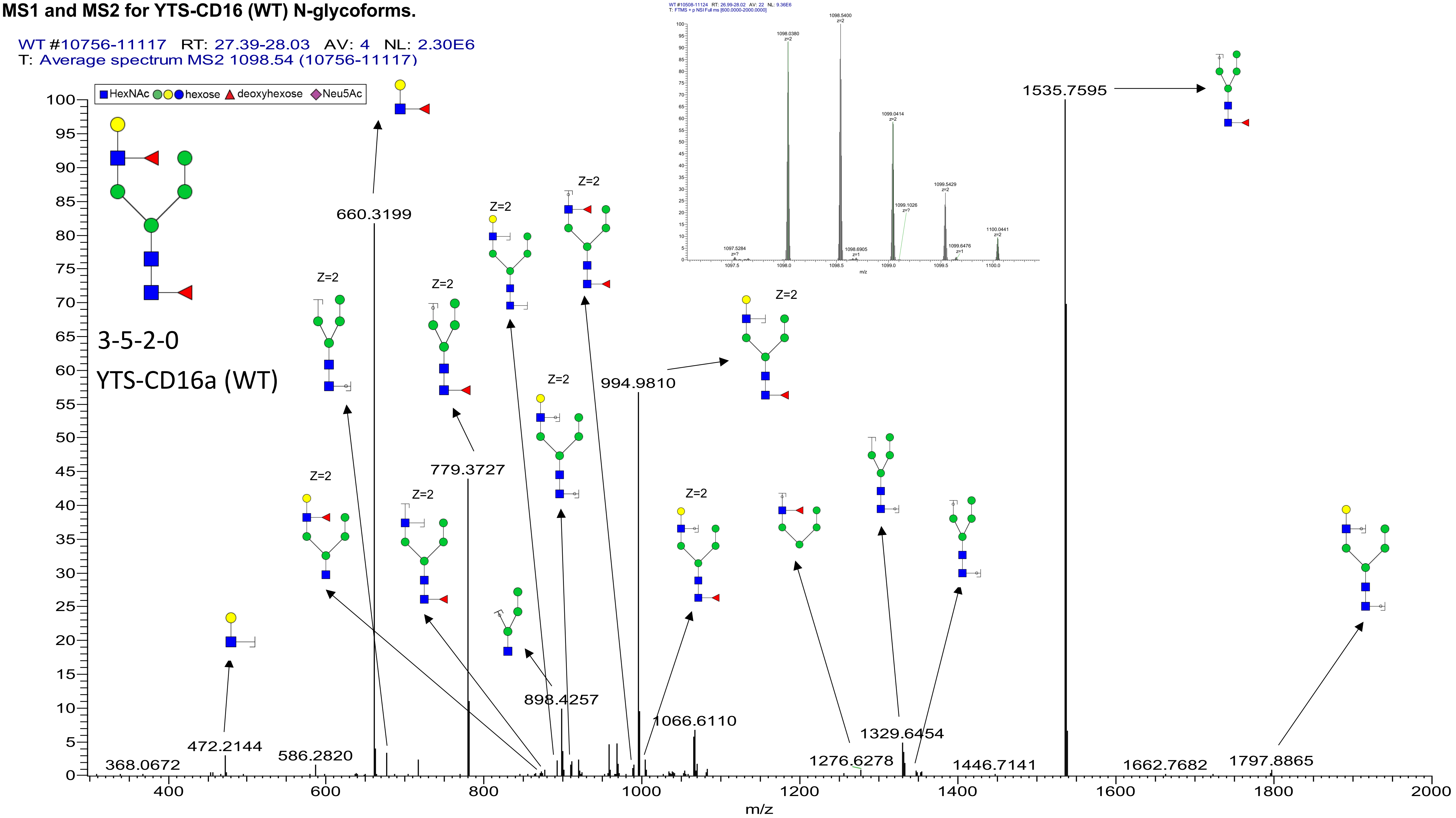

MS1 and MS2 for YTS-CD16 (WT) N-glycoforms.

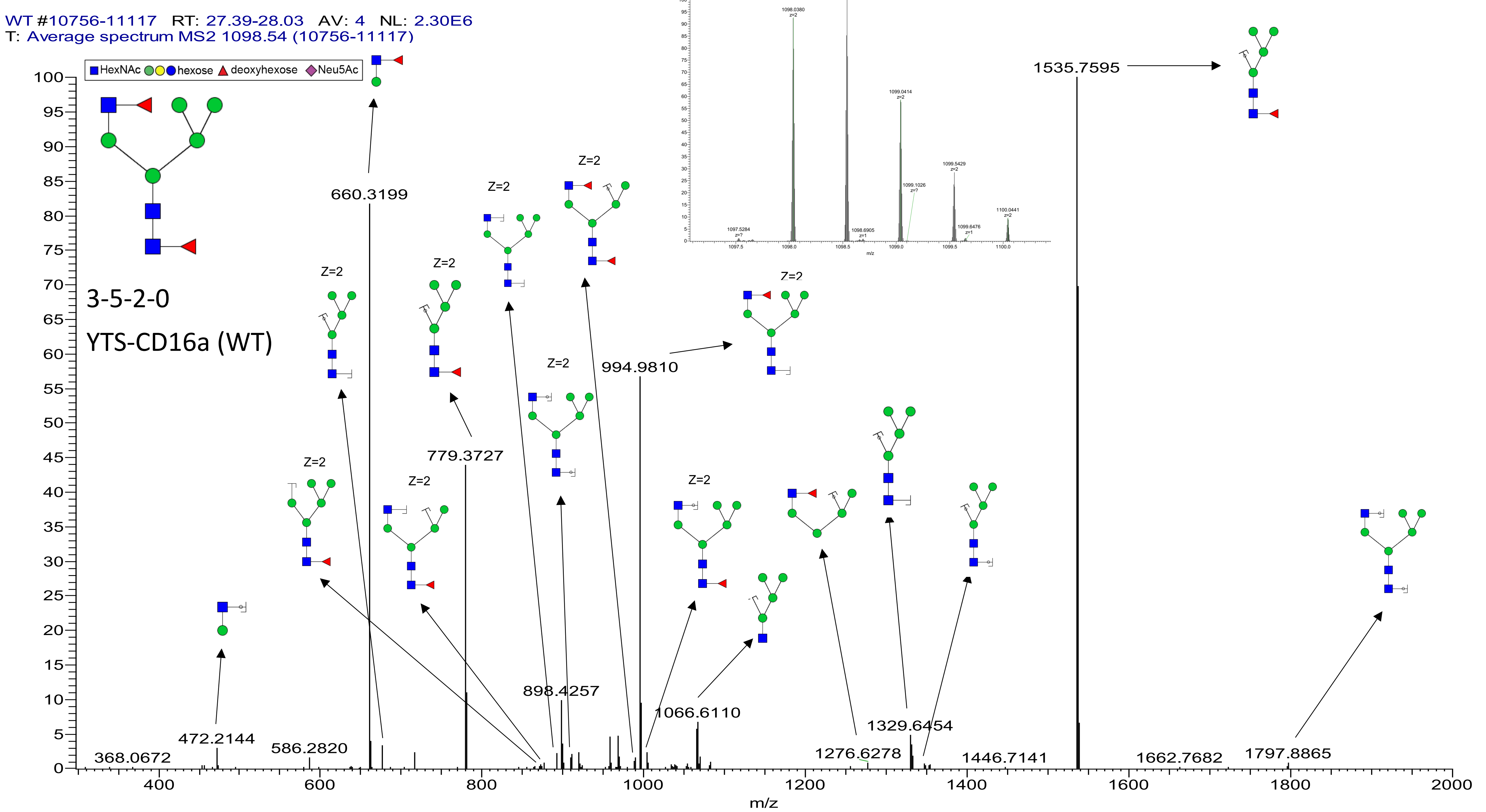

MS1 and MS2 for YTS-CD16 (WT) N-glycoforms.

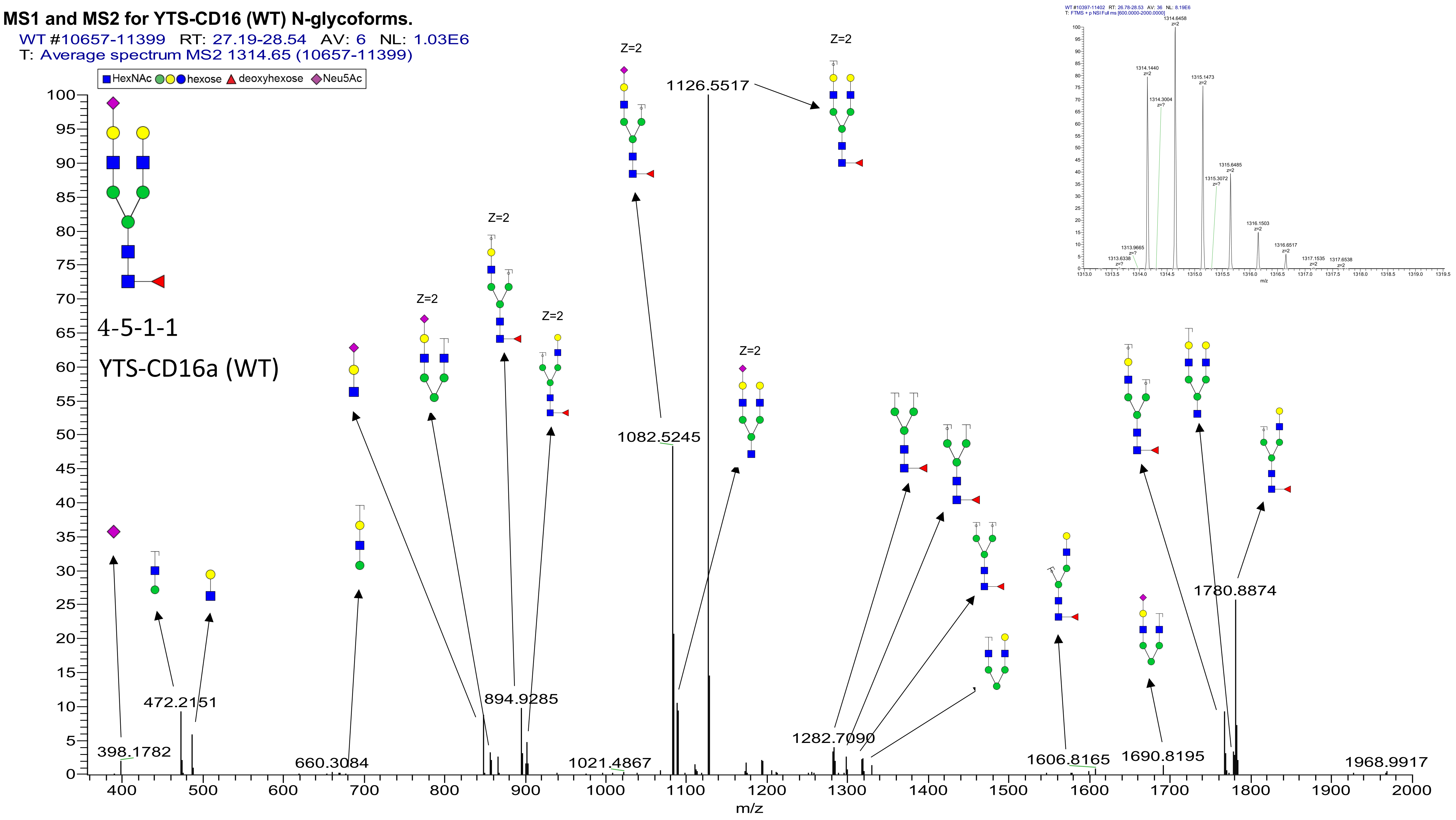

MS1 and MS2 for YTS-CD16 (WT) N-glycoforms.

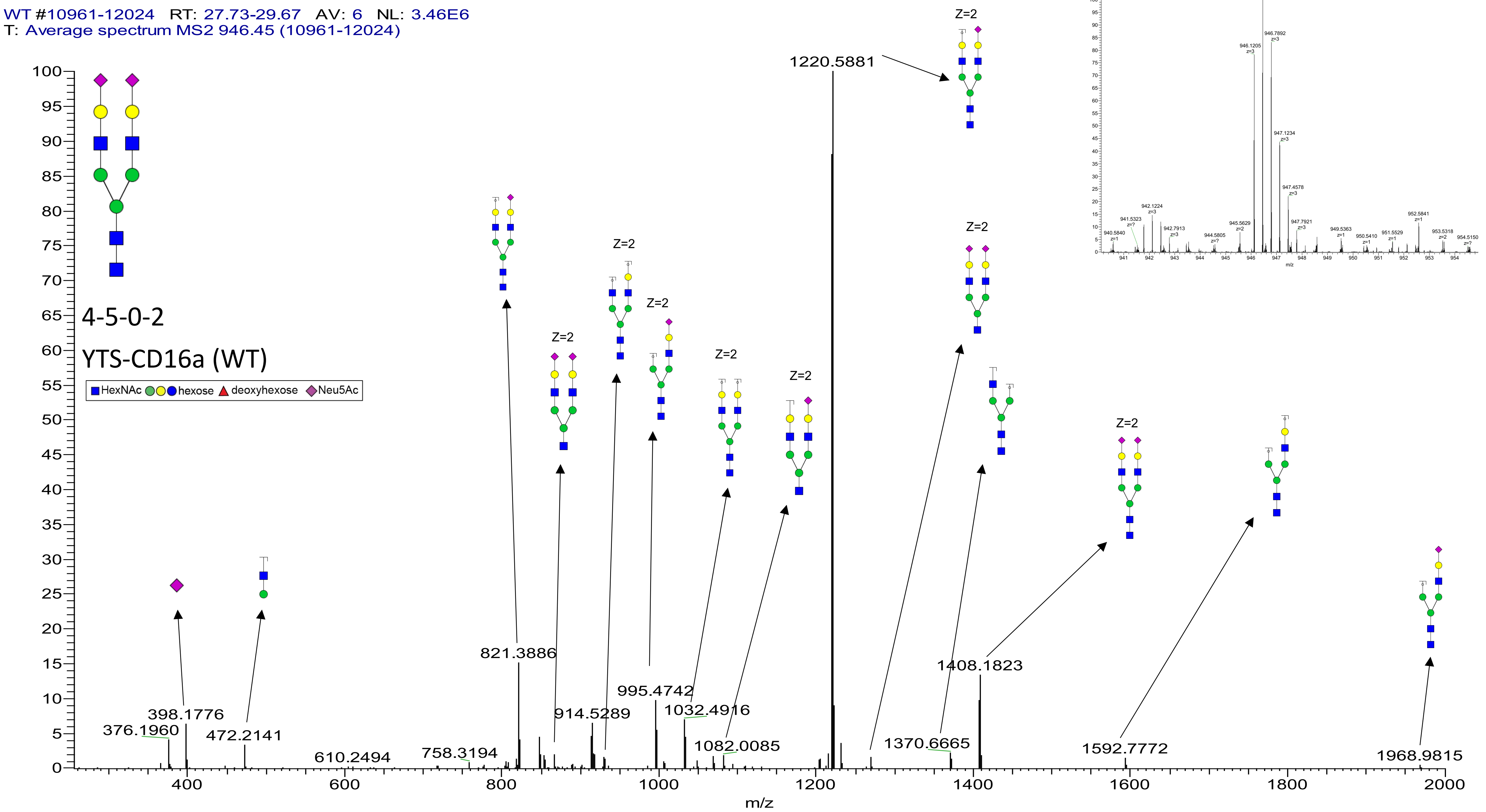

MS1 and MS2 for YTS-CD16 (WT) N-glycoforms.

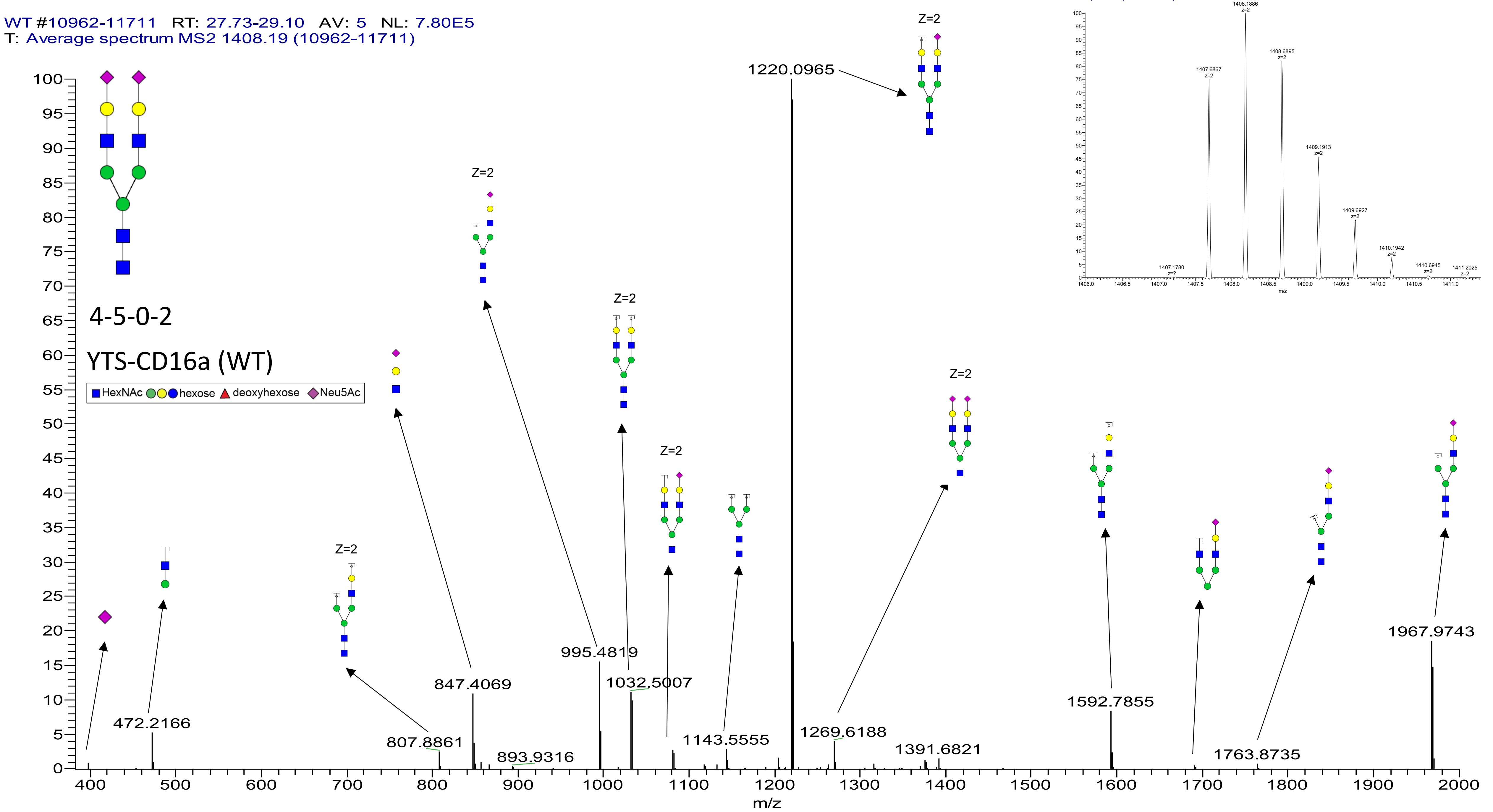

MS1 and MS2 for YTS-CD16 (WT) N-glycoforms.

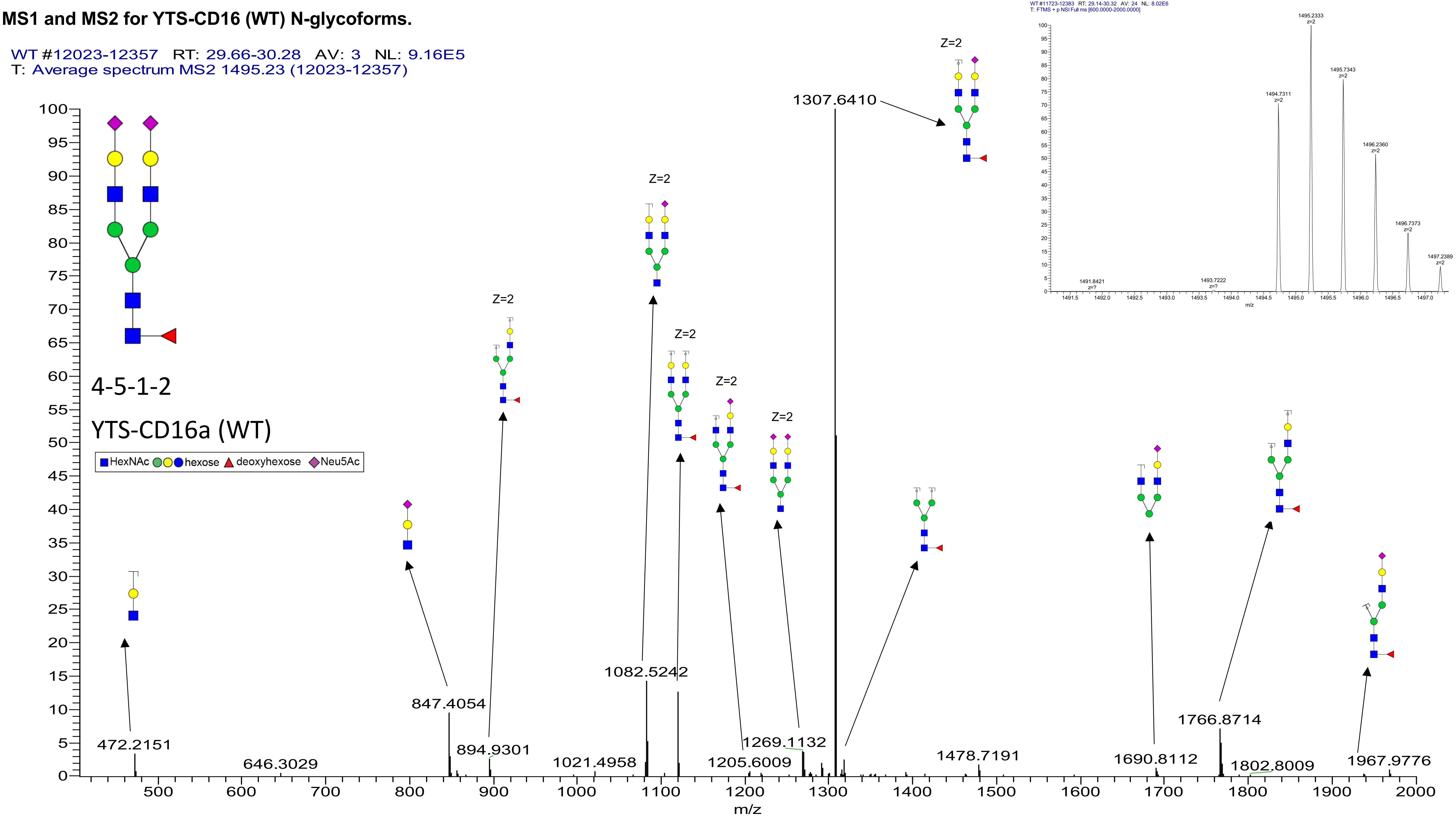

MS1 and MS2 for YTS-CD16 (WT) N-glycoforms.

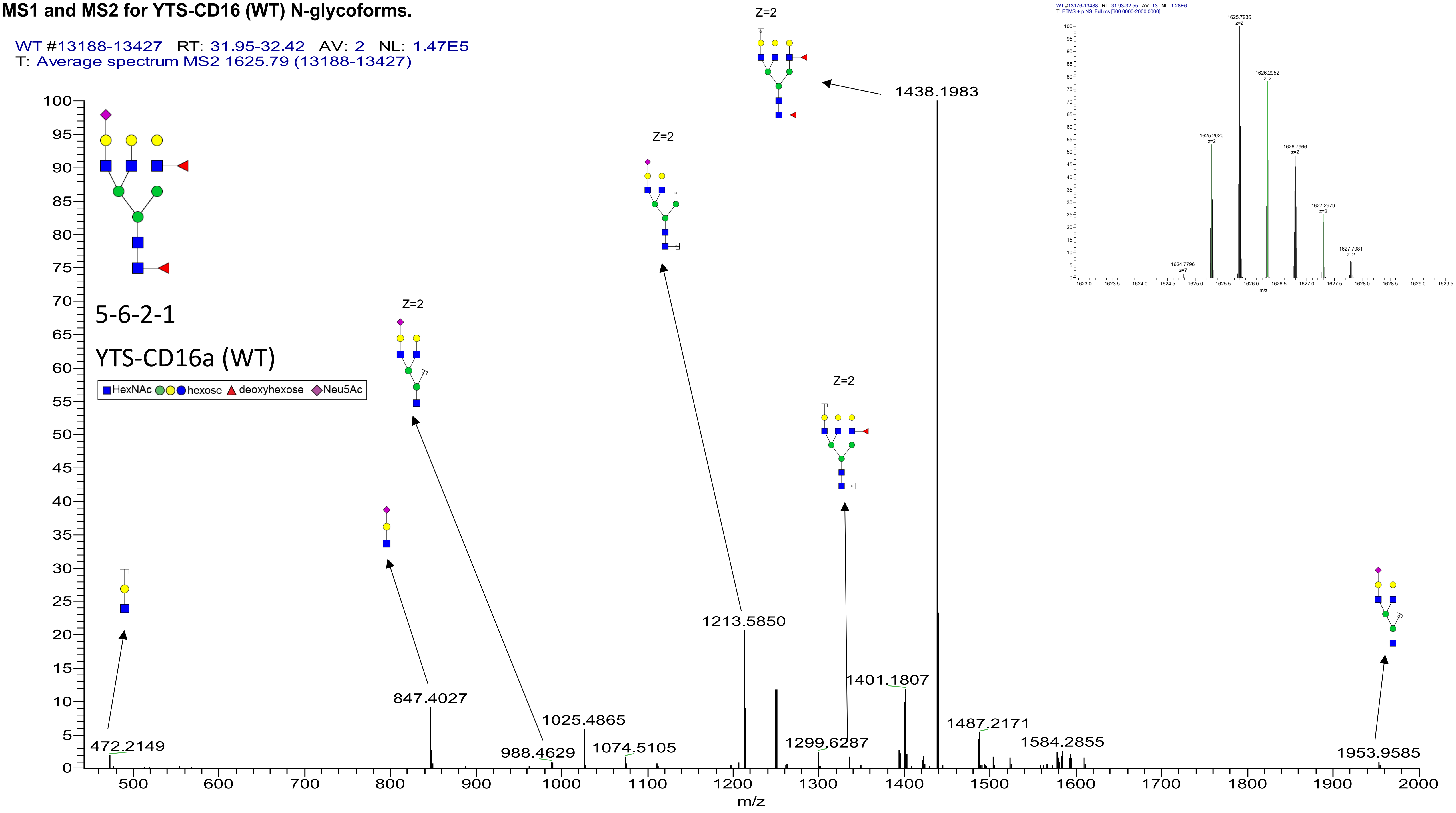

MS1 and MS2 for YTS-CD16 (WT) N-glycoforms.

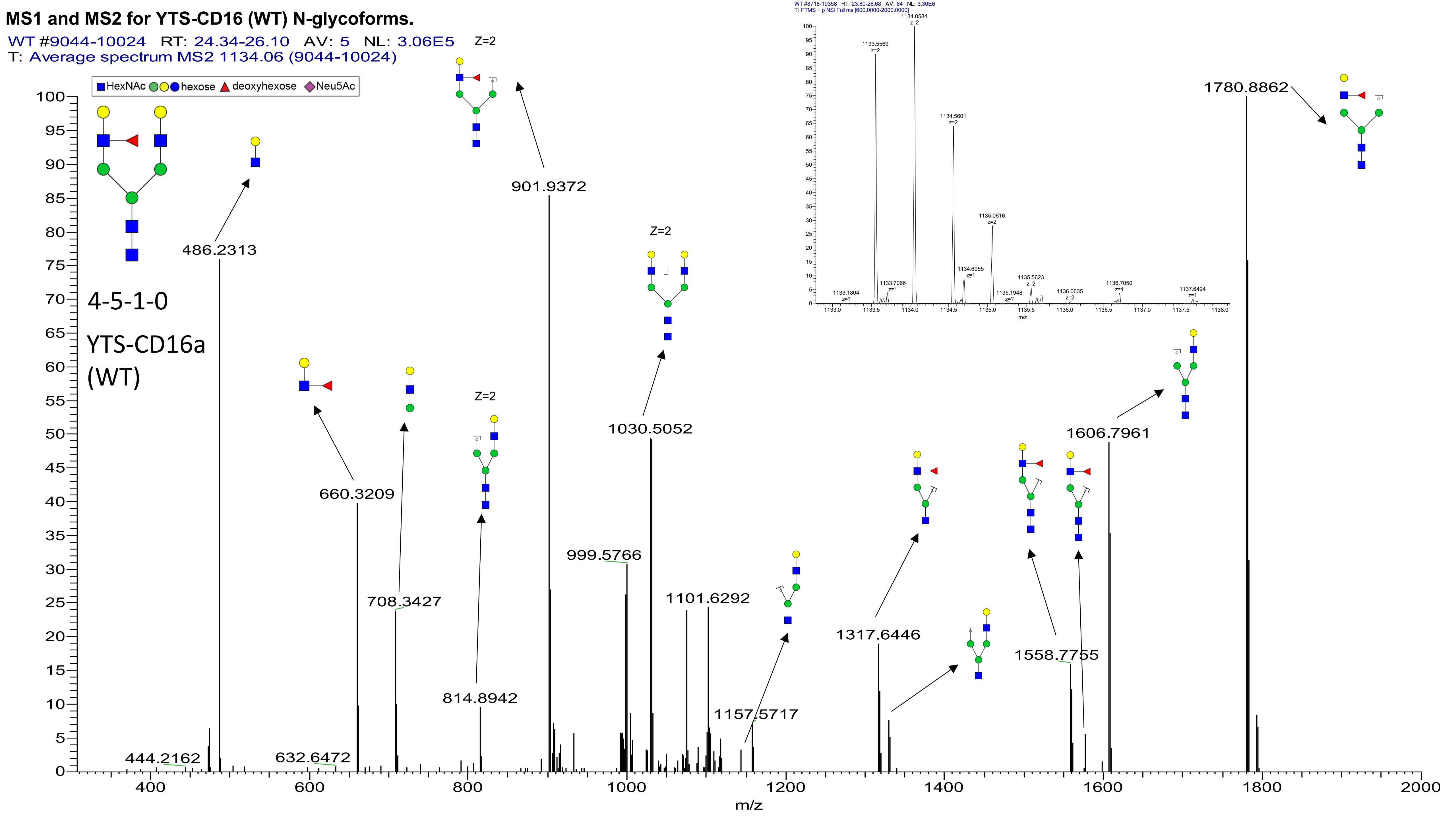

MS1 and MS2 for YTS-CD16 (WT) N-glycoforms.

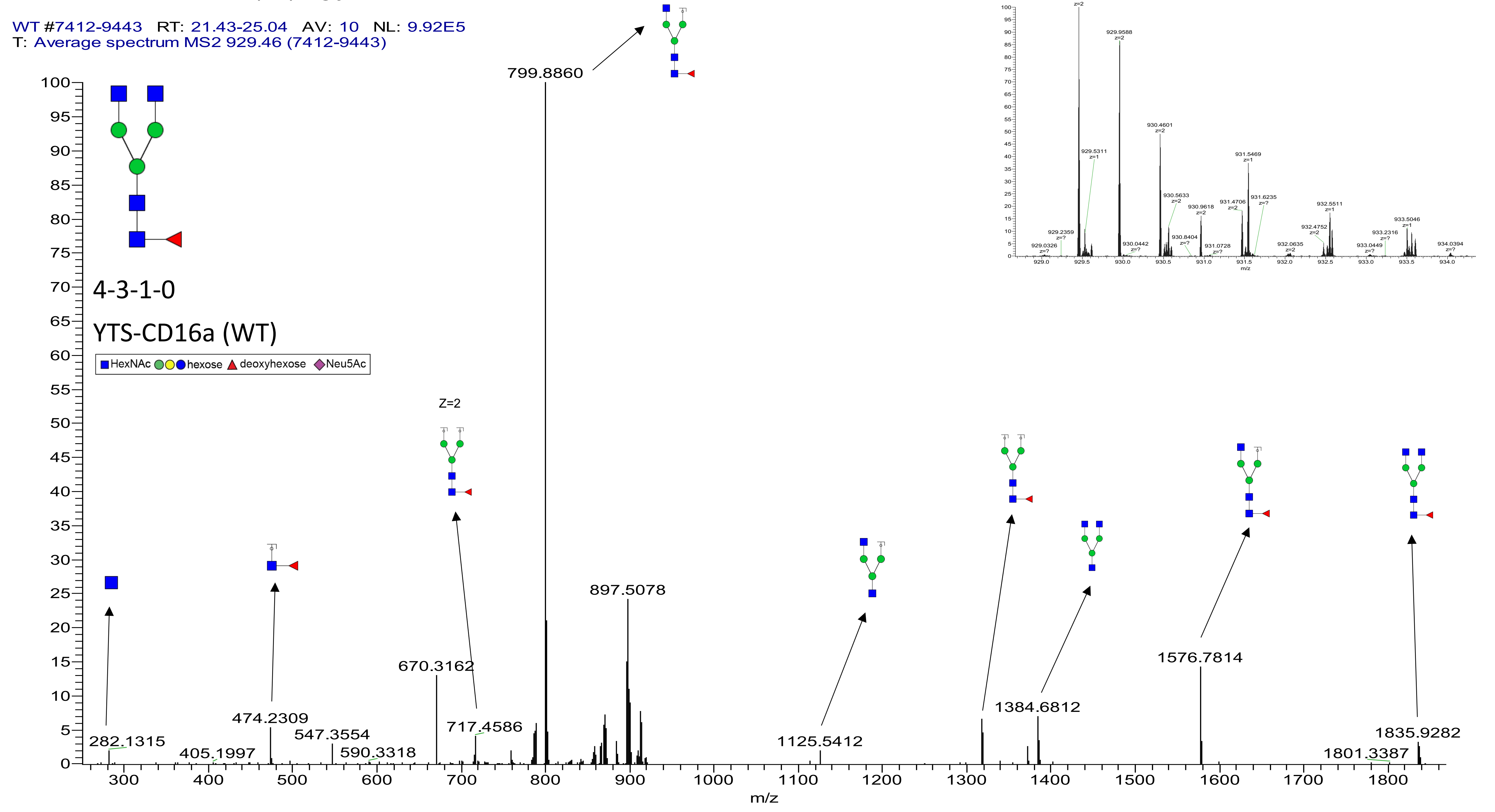

MS1 and MS2 for YTS-CD16 (WT) N-glycoforms.

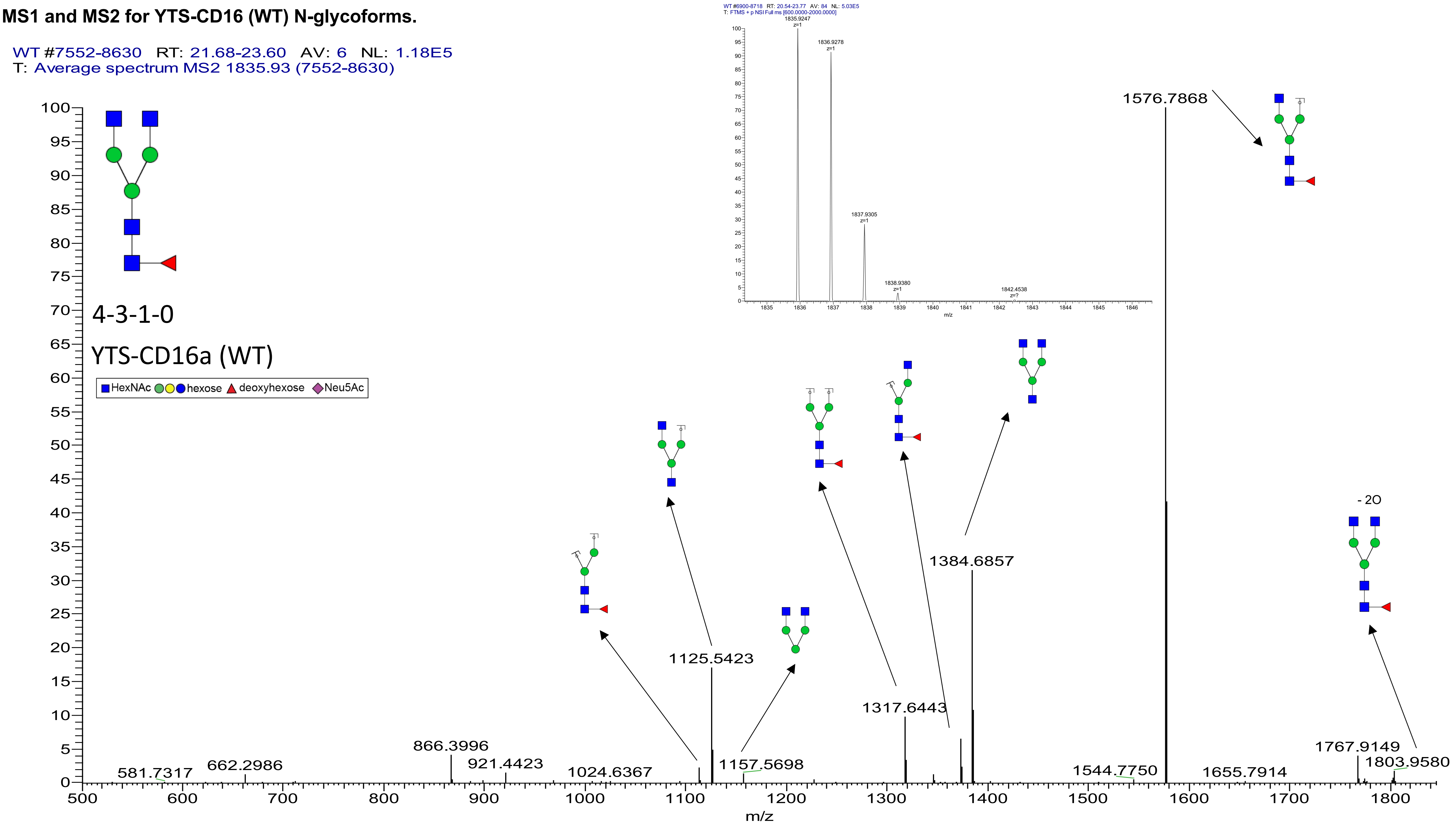

MS1 and MS2 for YTS-CD16 (WT) N-glycoforms.

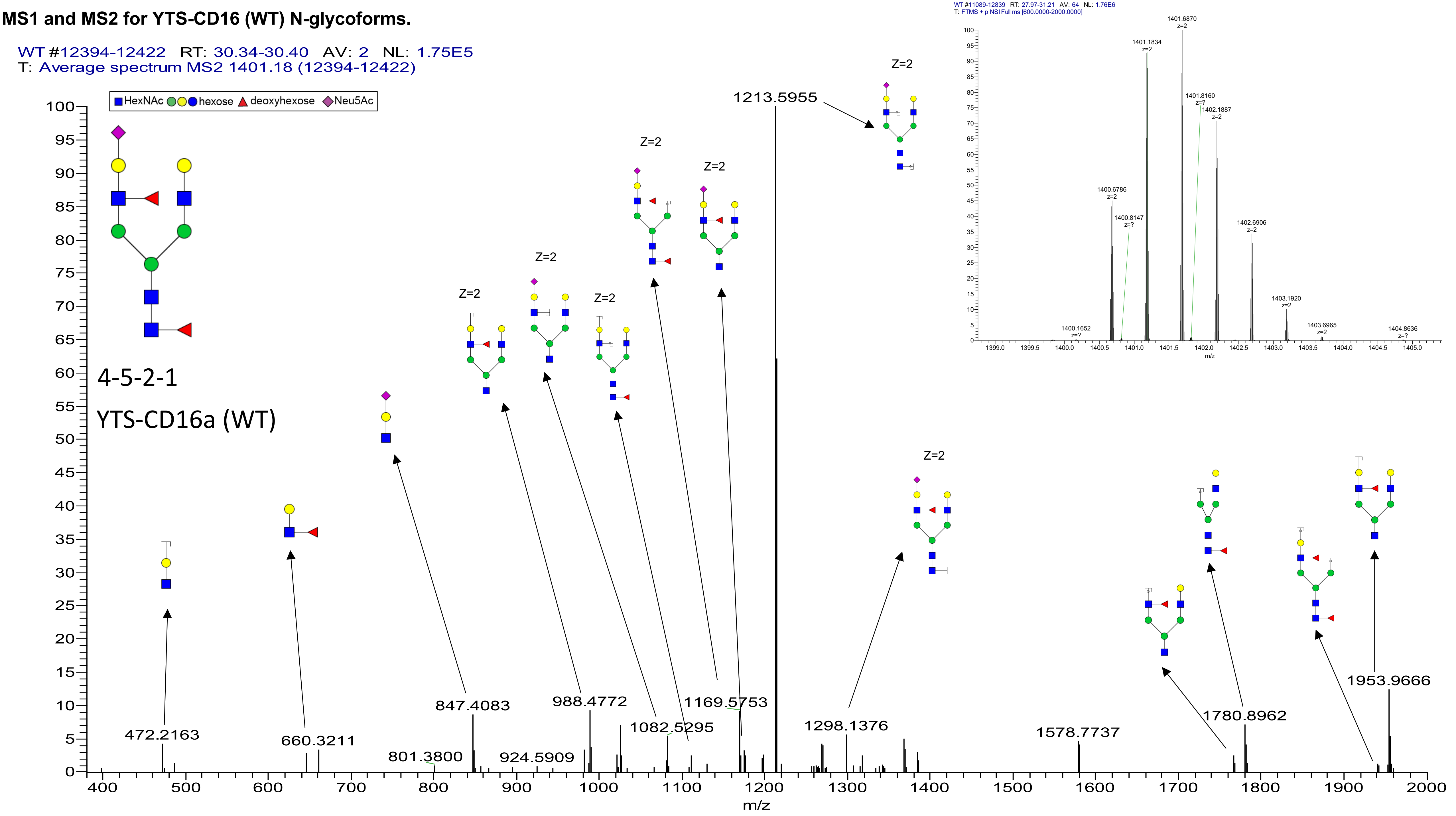

MS1 and MS2 for YTS-CD16 (WT) N-glycoforms.

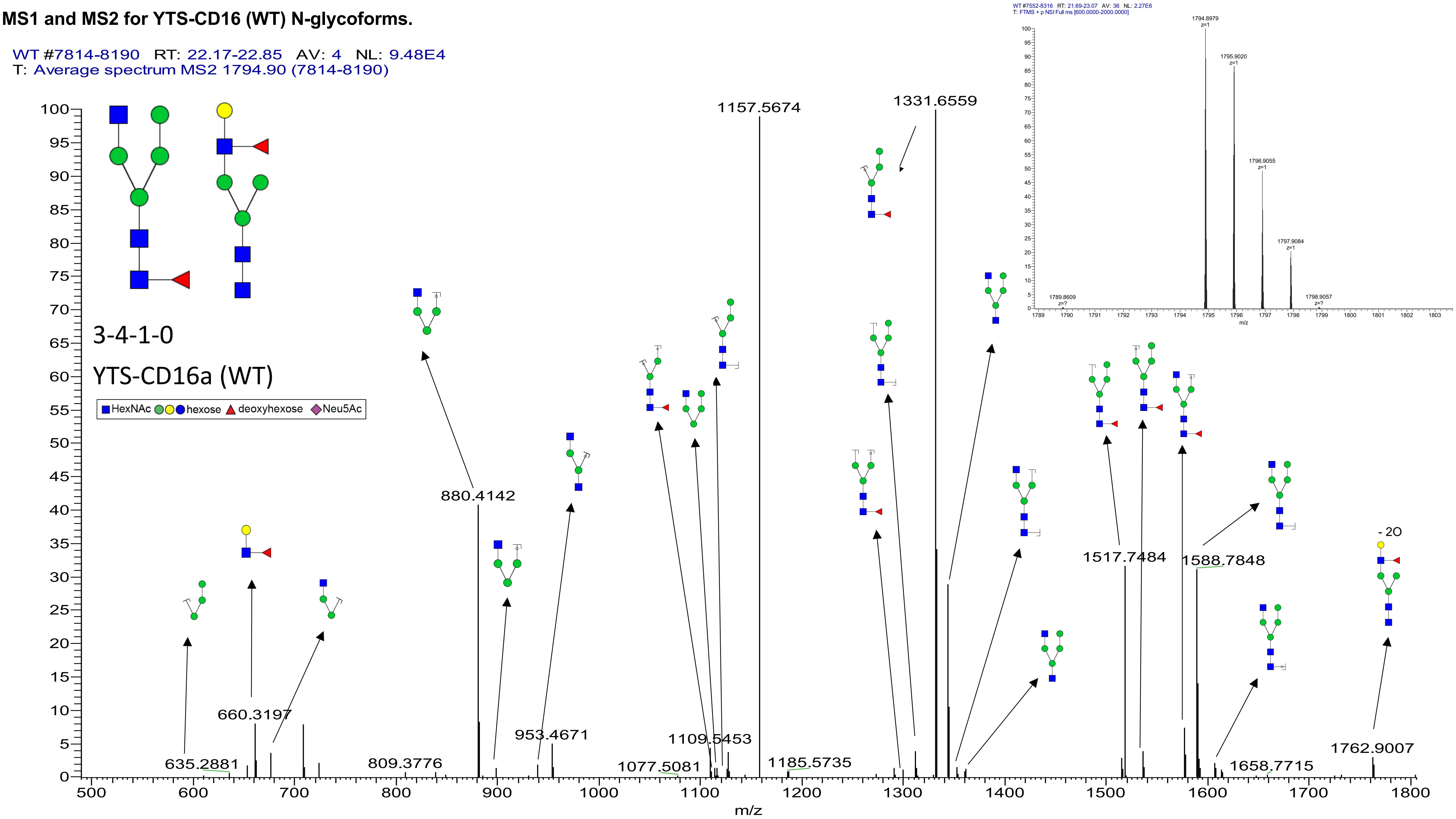

MS1 and MS2 for YTS-CD16 (WT) N-glycoforms.

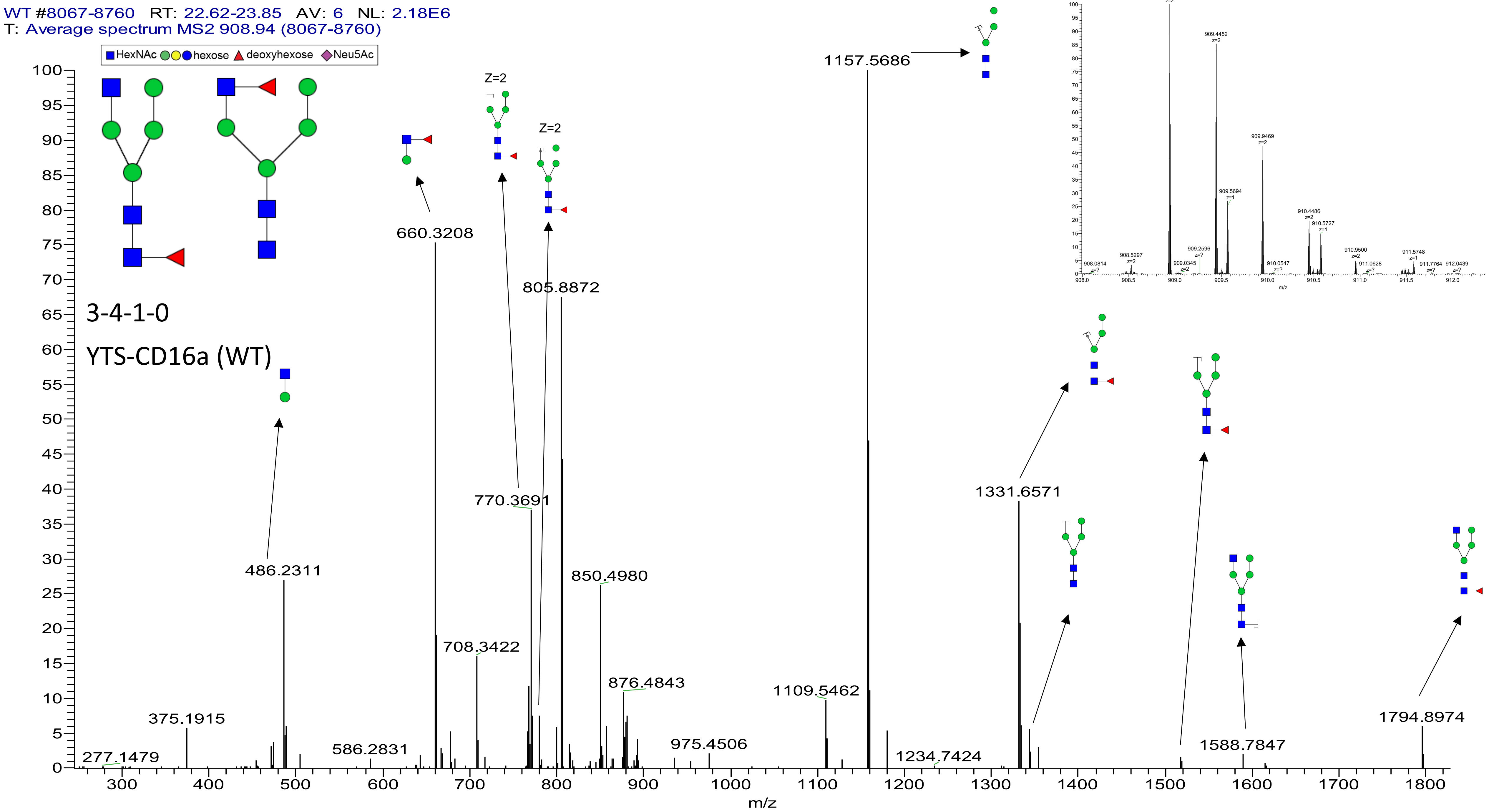

MS1 and MS2 for YTS-CD16 (WT) N-glycoforms.

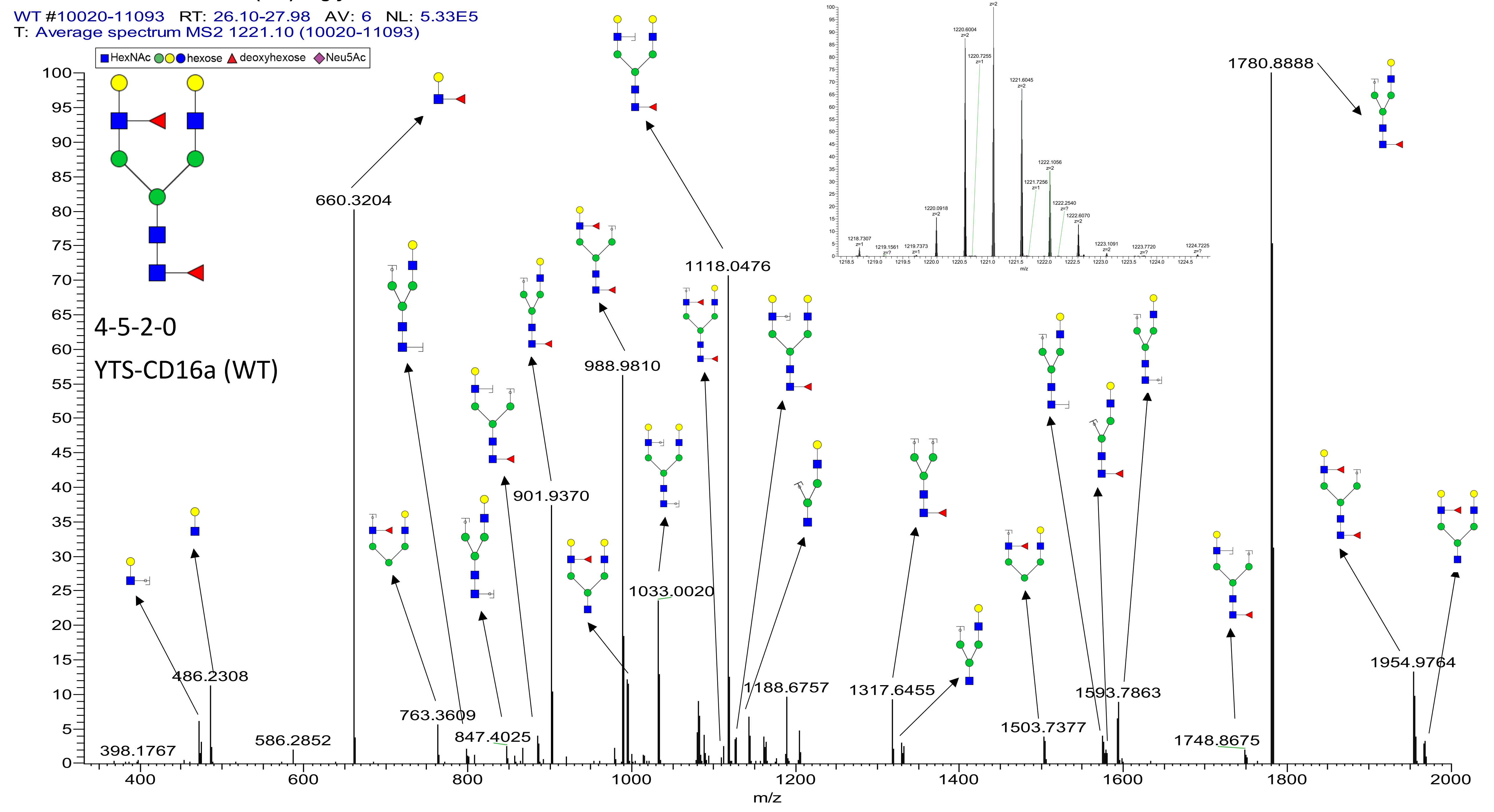

MS1 and MS2 for YTS-CD16 (WT) N-glycoforms.

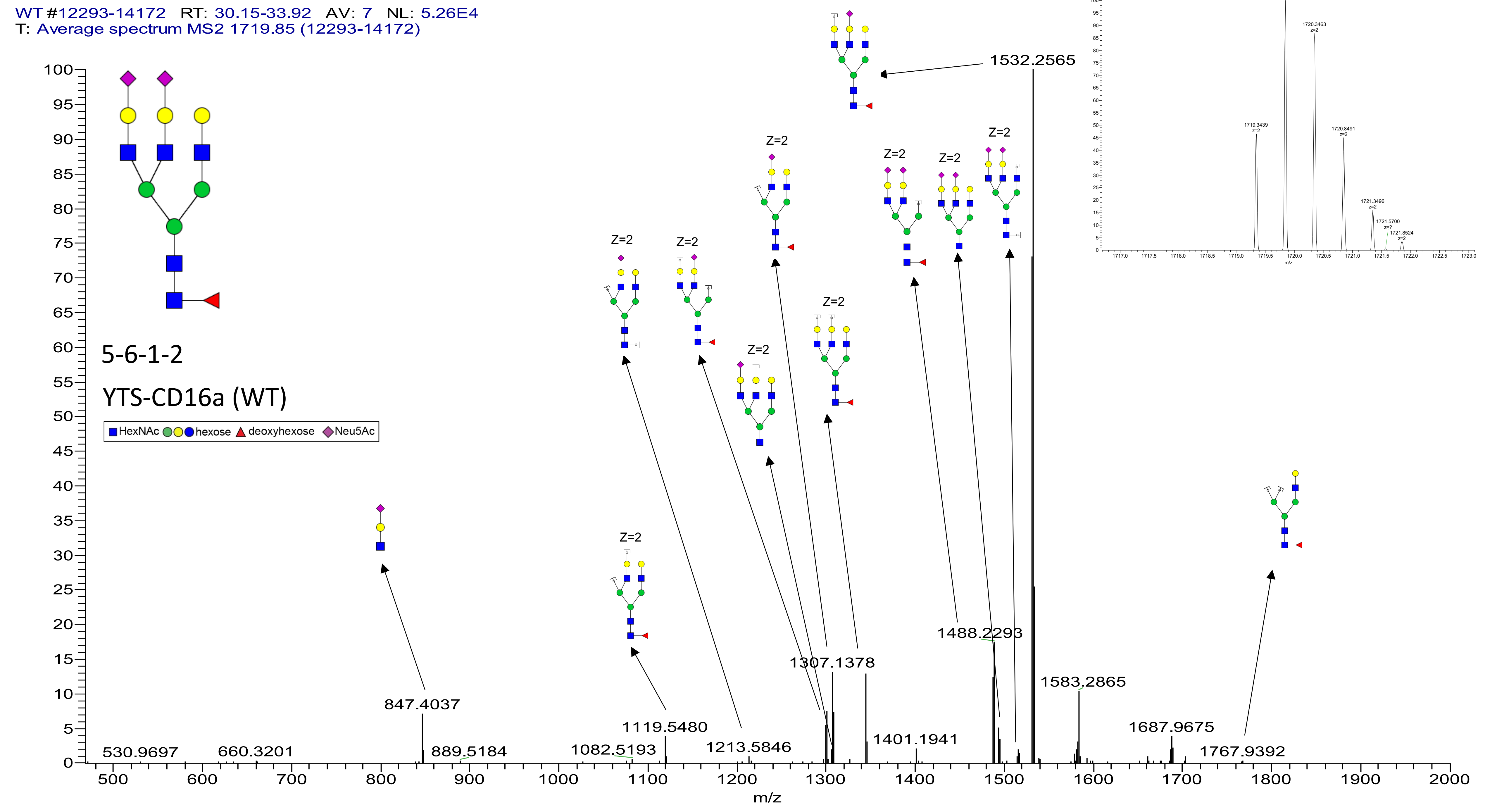

MS1 and MS2 for YTS-CD16 (WT) N-glycoforms.

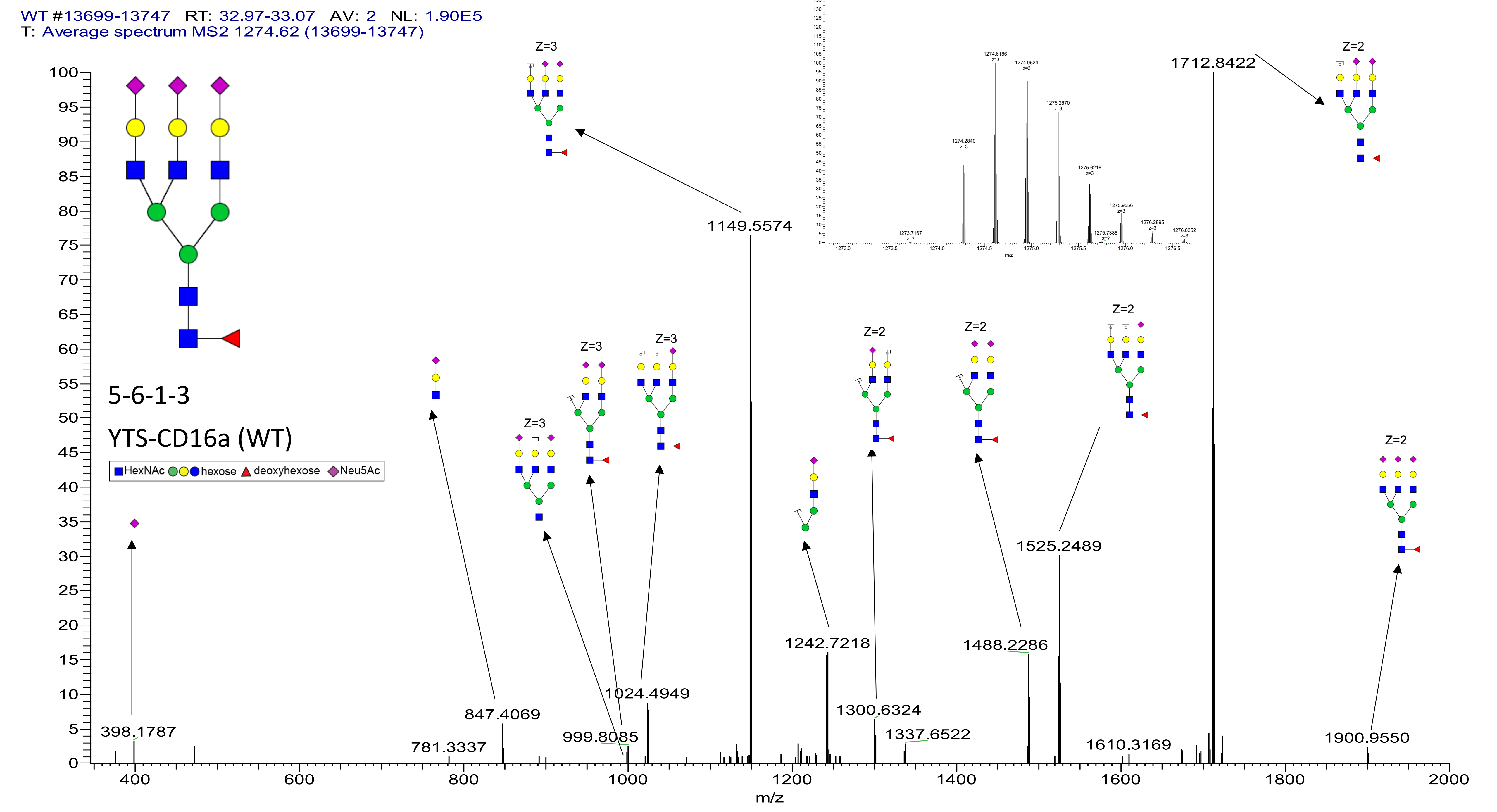

MS1 and MS2 for YTS-CD16 (WT) N-glycoforms.

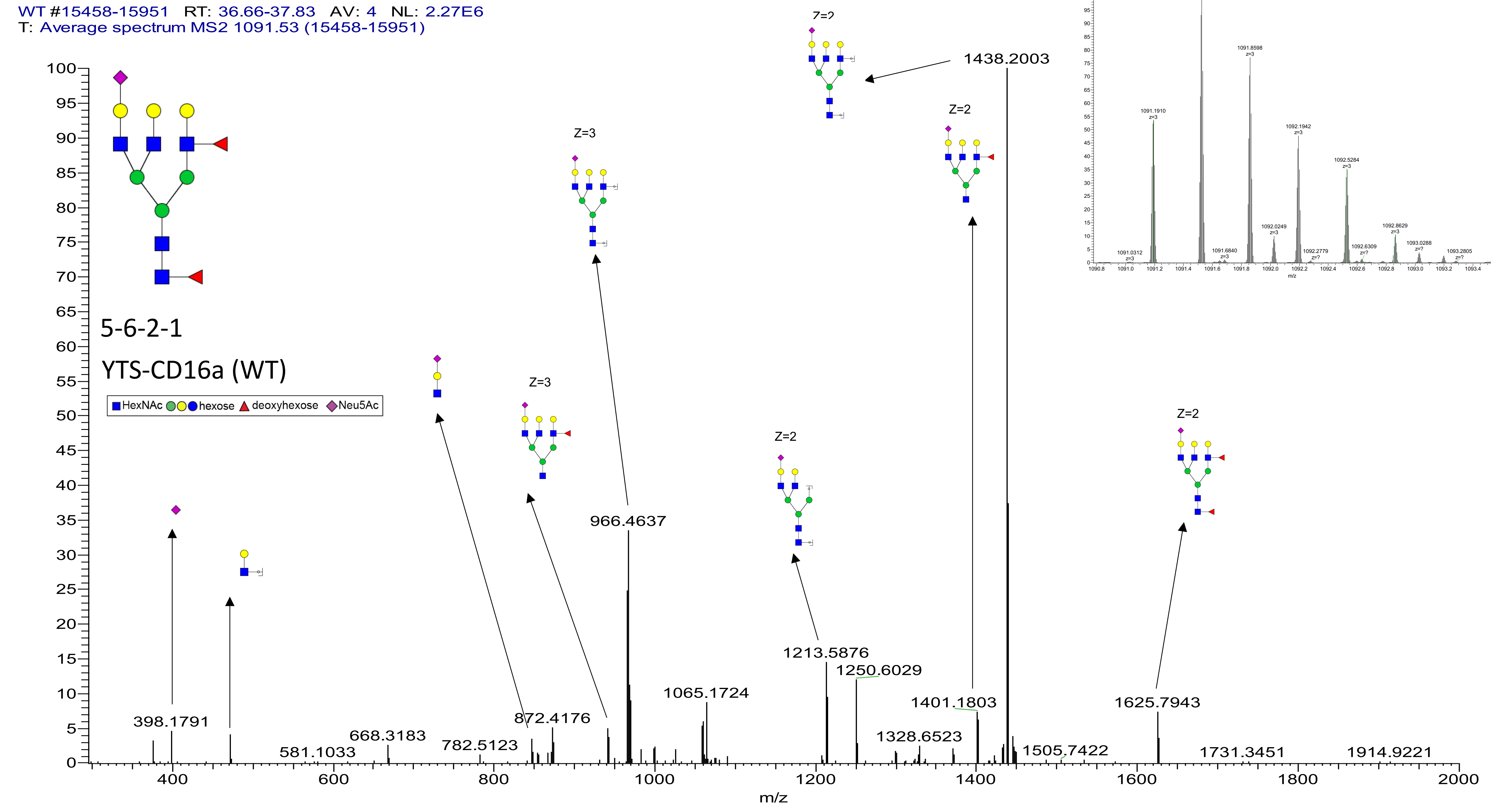

MS1 and MS2 for YTS-CD16 (WT) N-glycoforms.

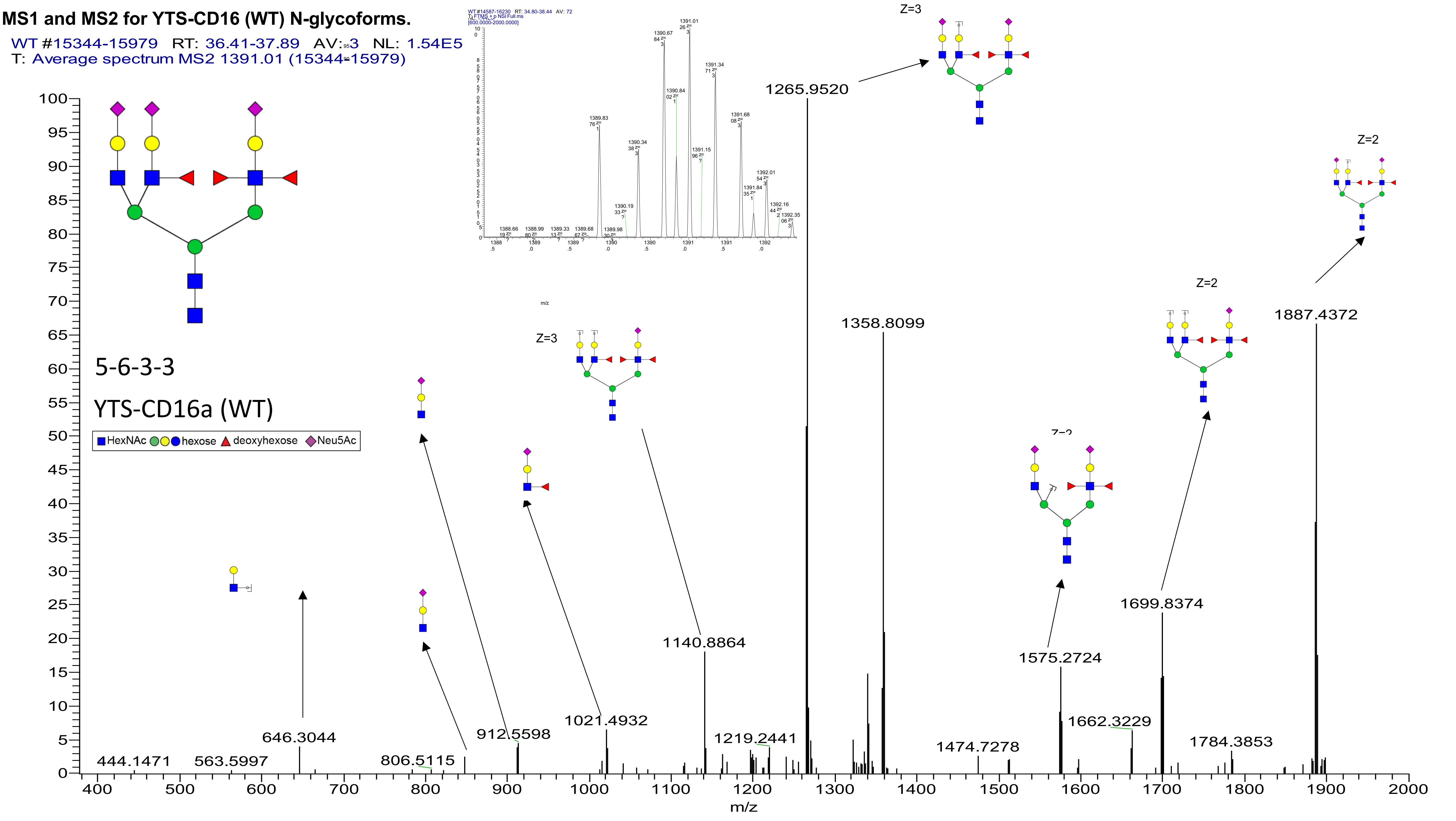

MS1 and MS2 for YTS-CD16 (WT) N-glycoforms.

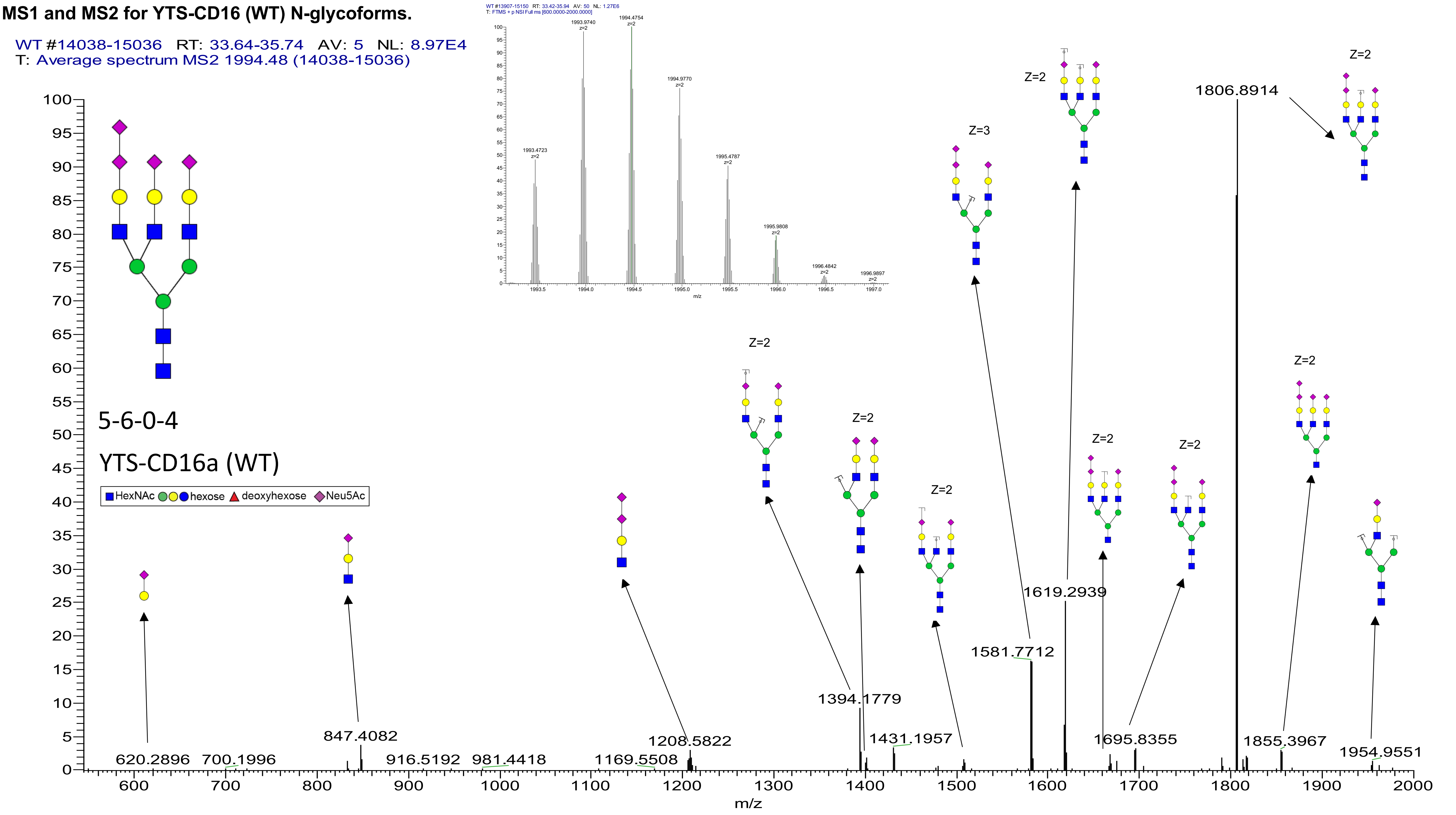

MS1 and MS2 for YTS-CD16 (WT) N-glycoforms.

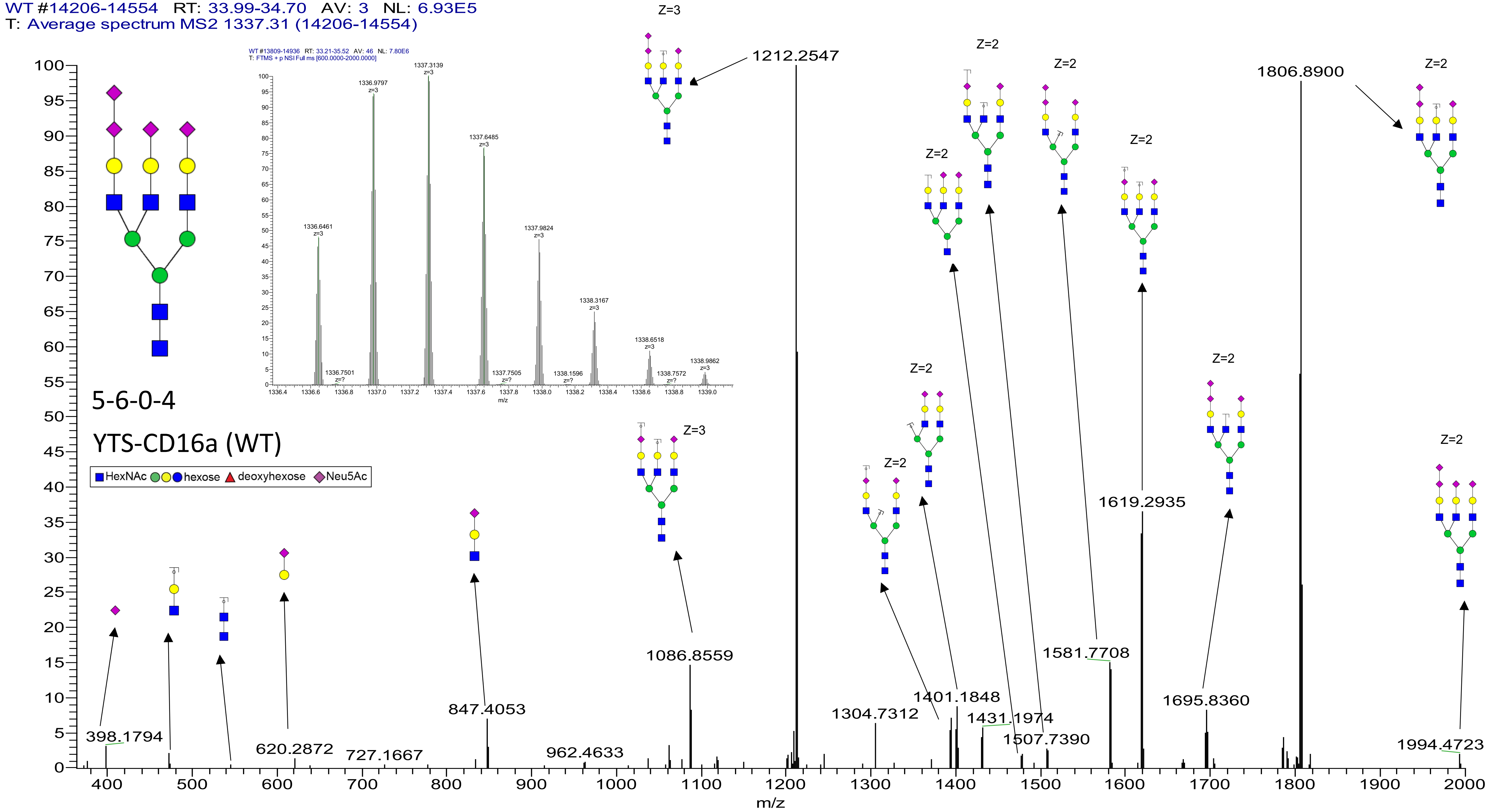

MS1 and MS2 for YTS-CD16 treated with Kifunensine (20uM) N-glycoforms.

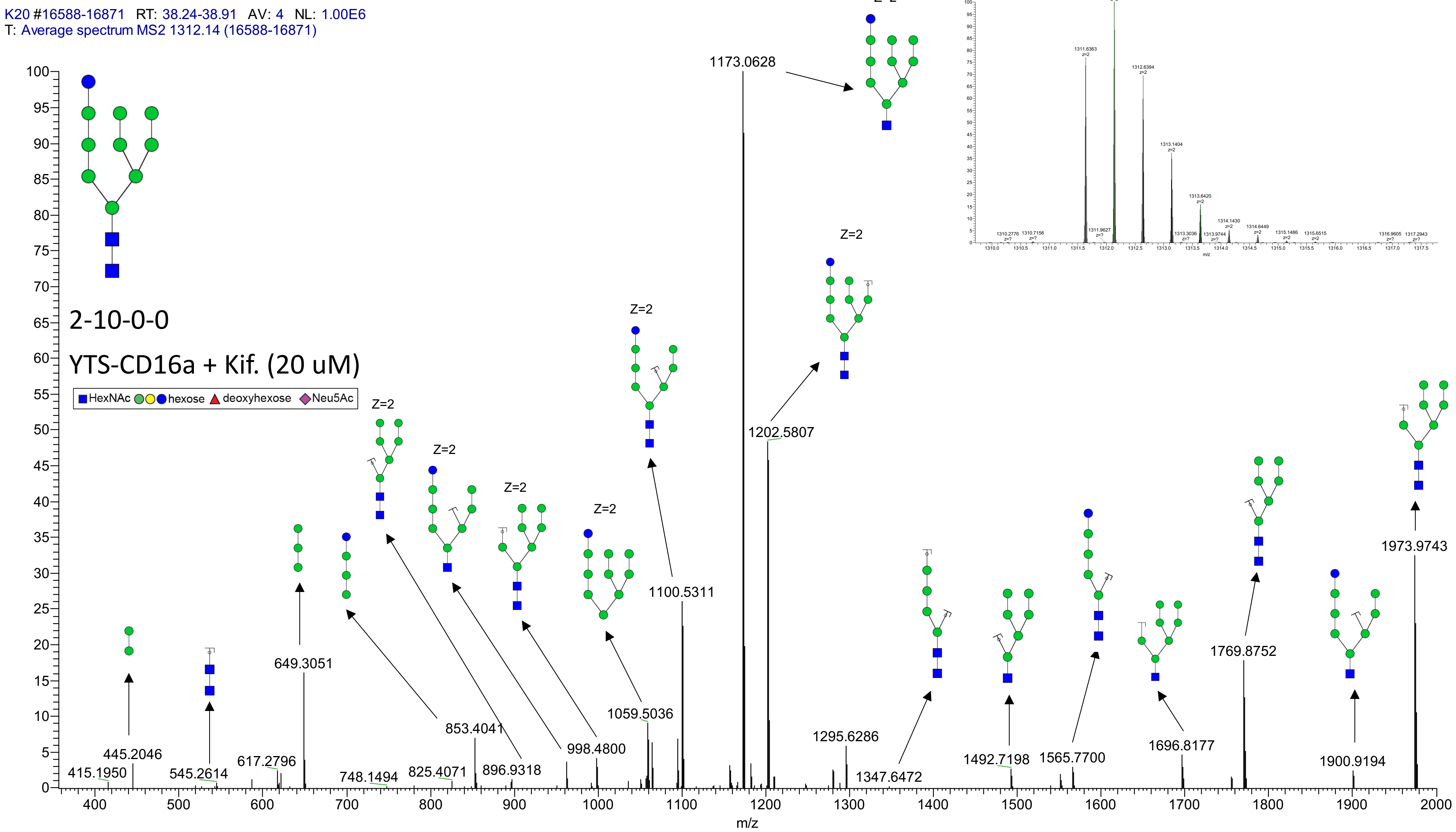

MS1 and MS2 for YTS-CD16 treated with Kifunensine (20uM) N-glycoforms.

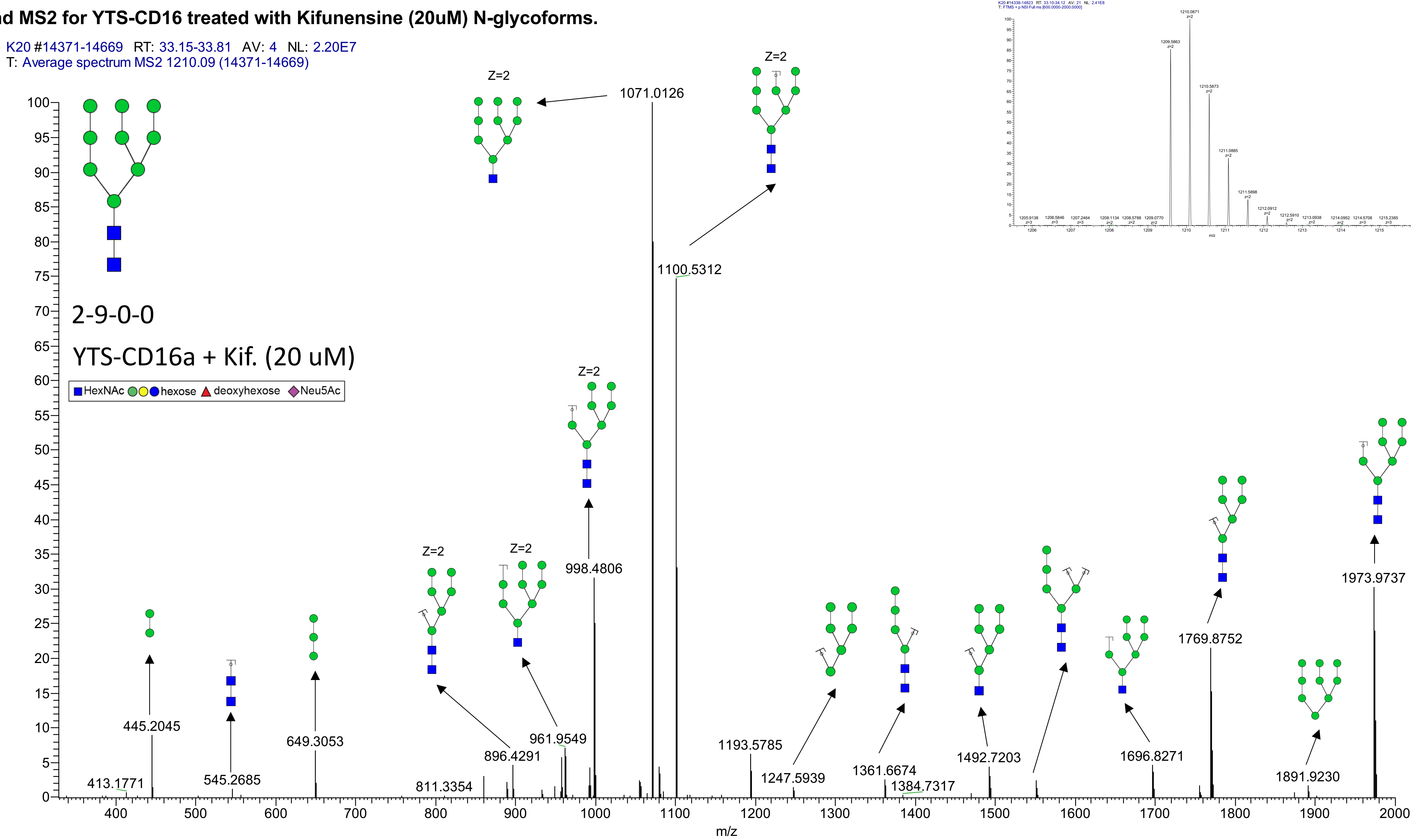

MS1 and MS2 for YTS-CD16 treated with Kifunensine (20uM) N-glycoforms.

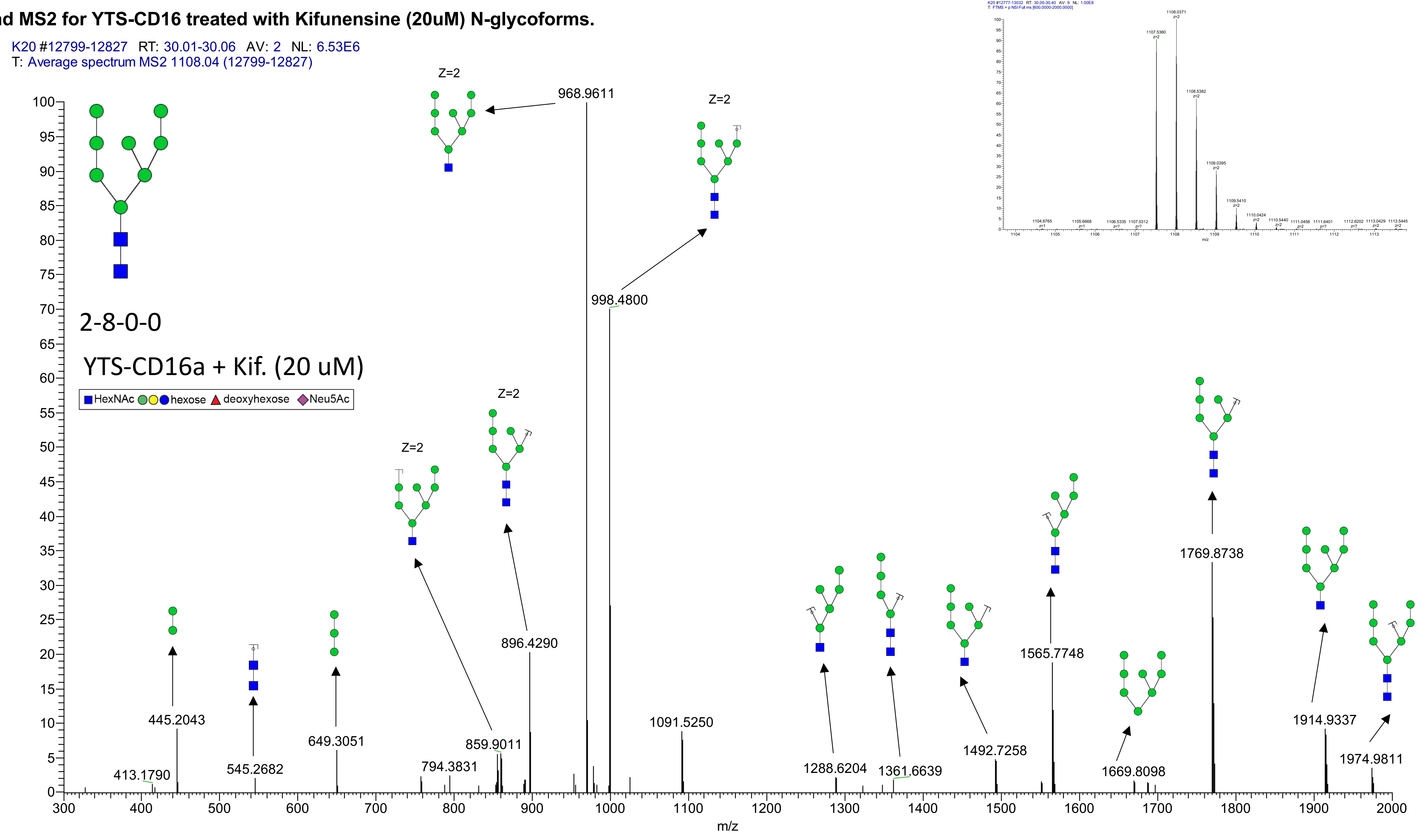

MS1 and MS2 for YTS-CD16 treated with Kifunensine (20uM) N-glycoforms.

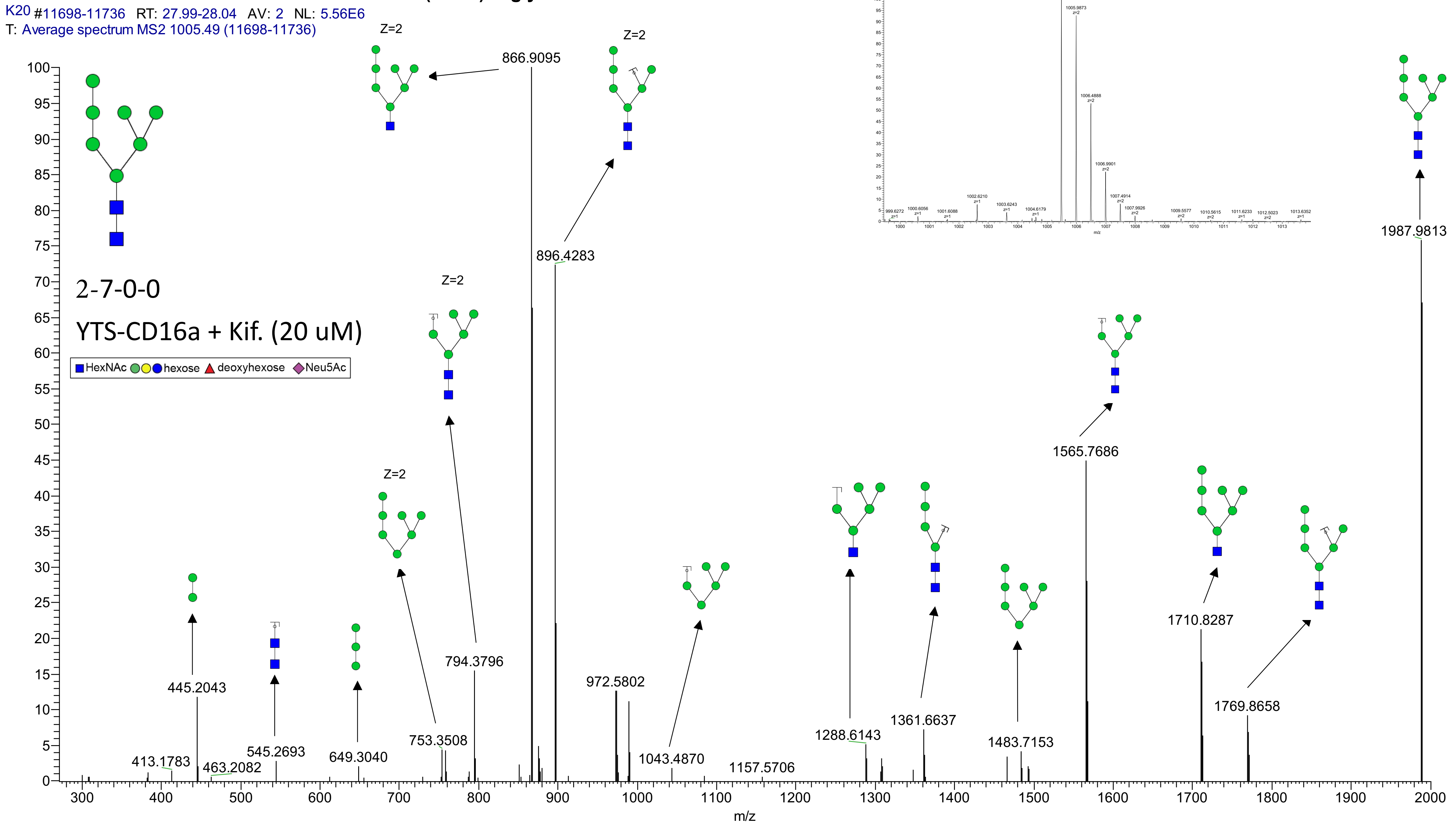

MS1 and MS2 for YTS-CD16 treated with Kifunensine (20uM) N-glycoforms.

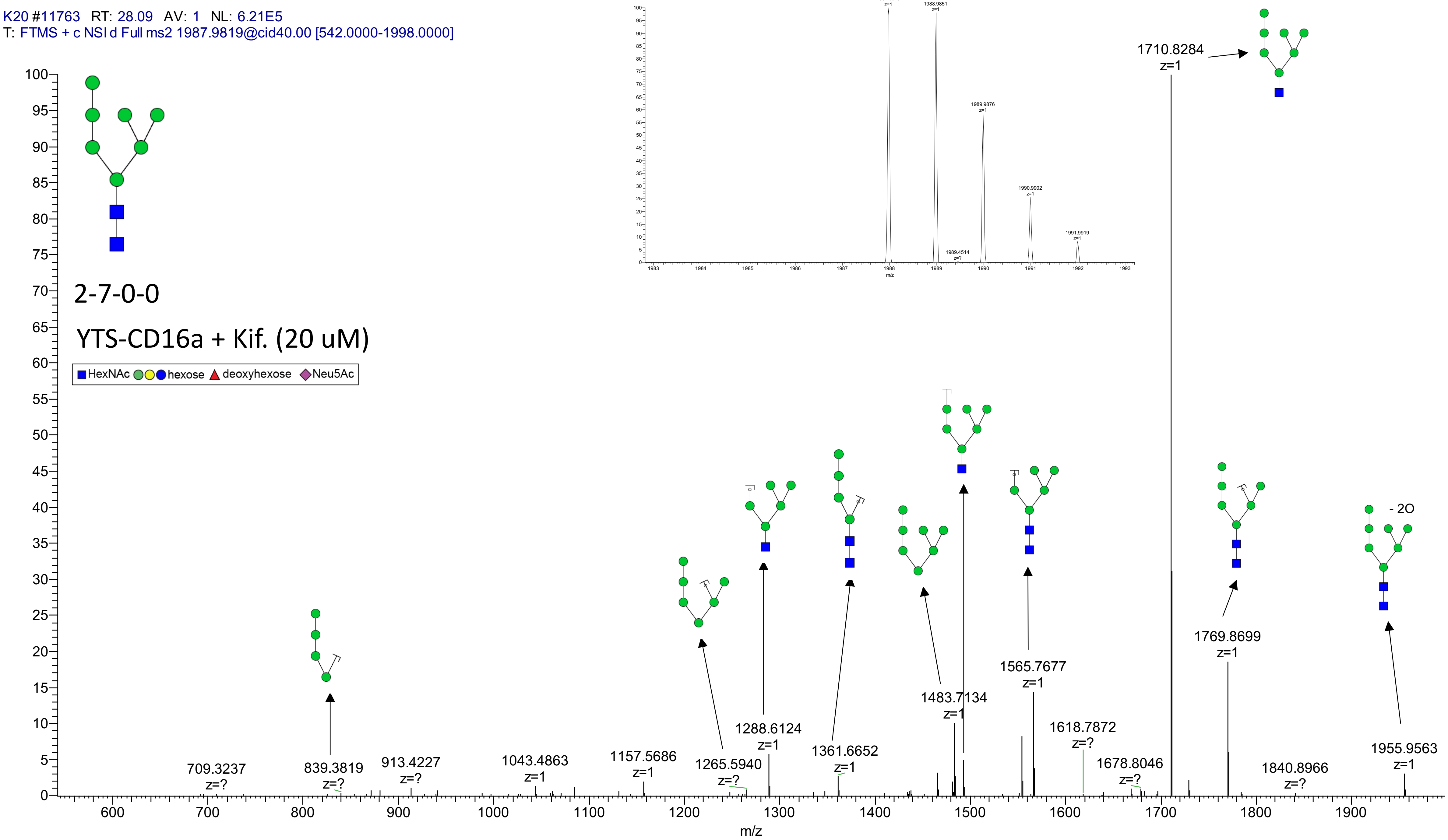

MS1 and MS2 for YTS-CD16 treated with Kifunensine (20uM) N-glycoforms.

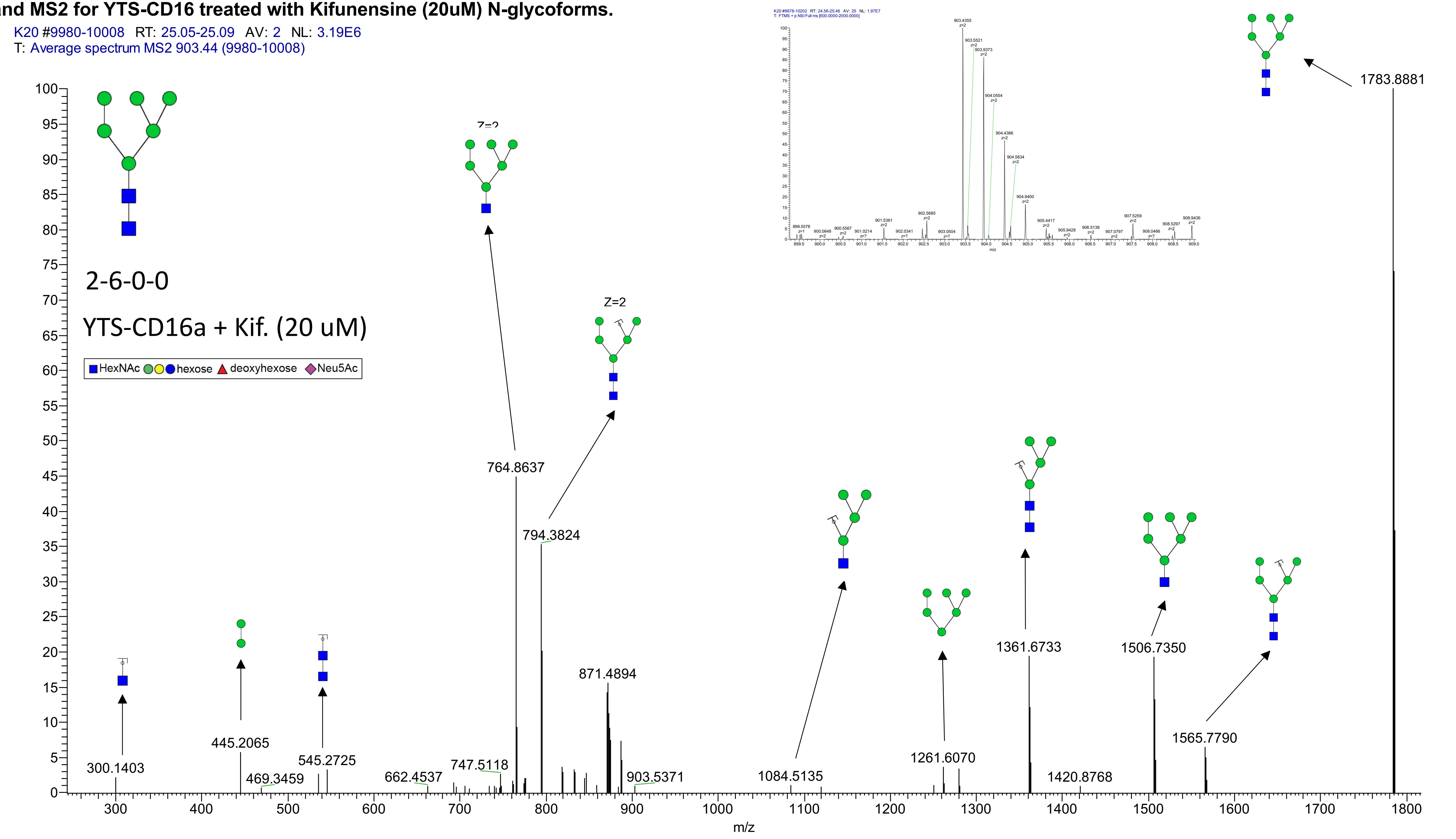

MS1 and MS2 for YTS-CD16 treated with Kifunensine (20uM) N-glycoforms.

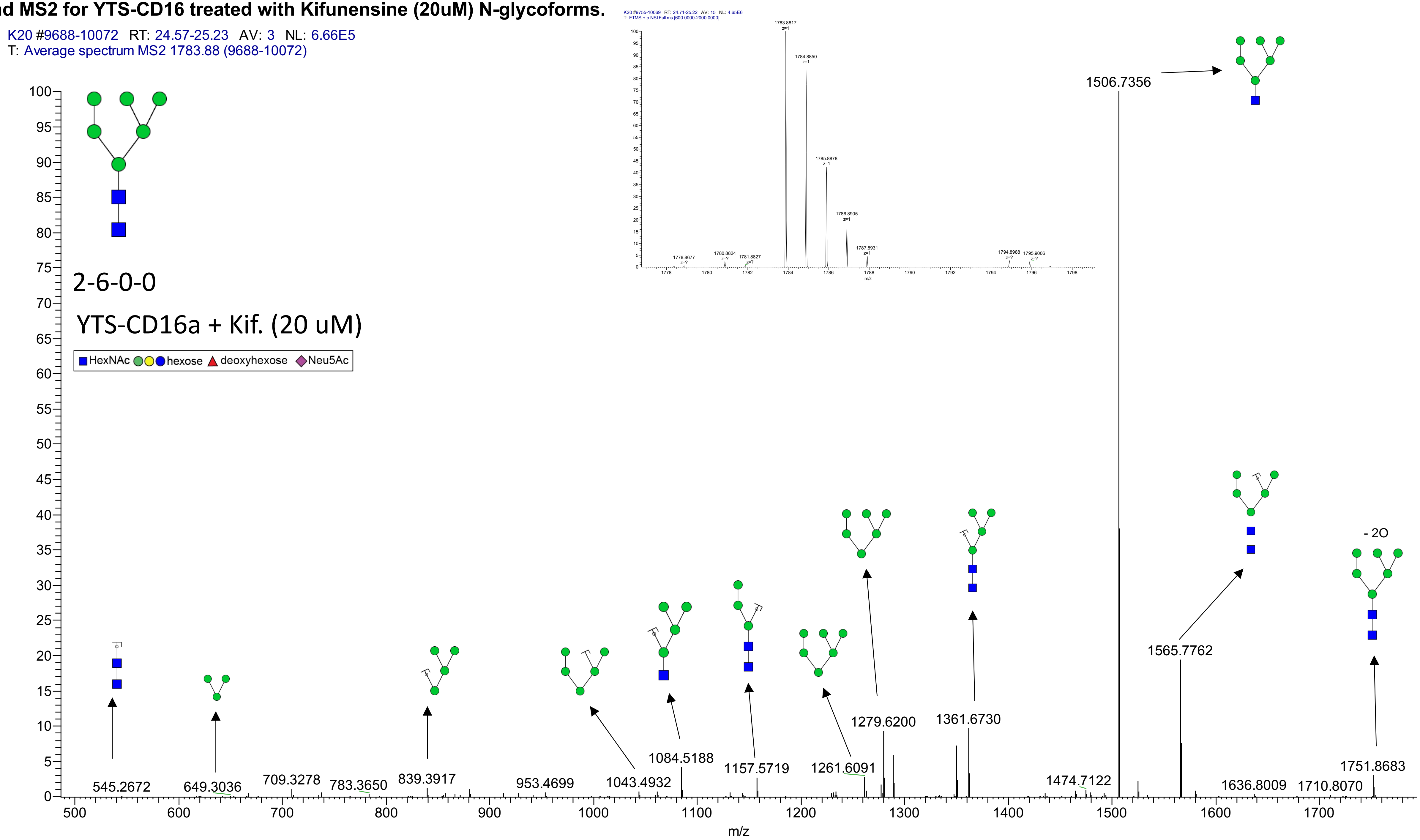

MS1 and MS2 for YTS-CD16 treated with Kifunensine (20uM) N-glycoforms.

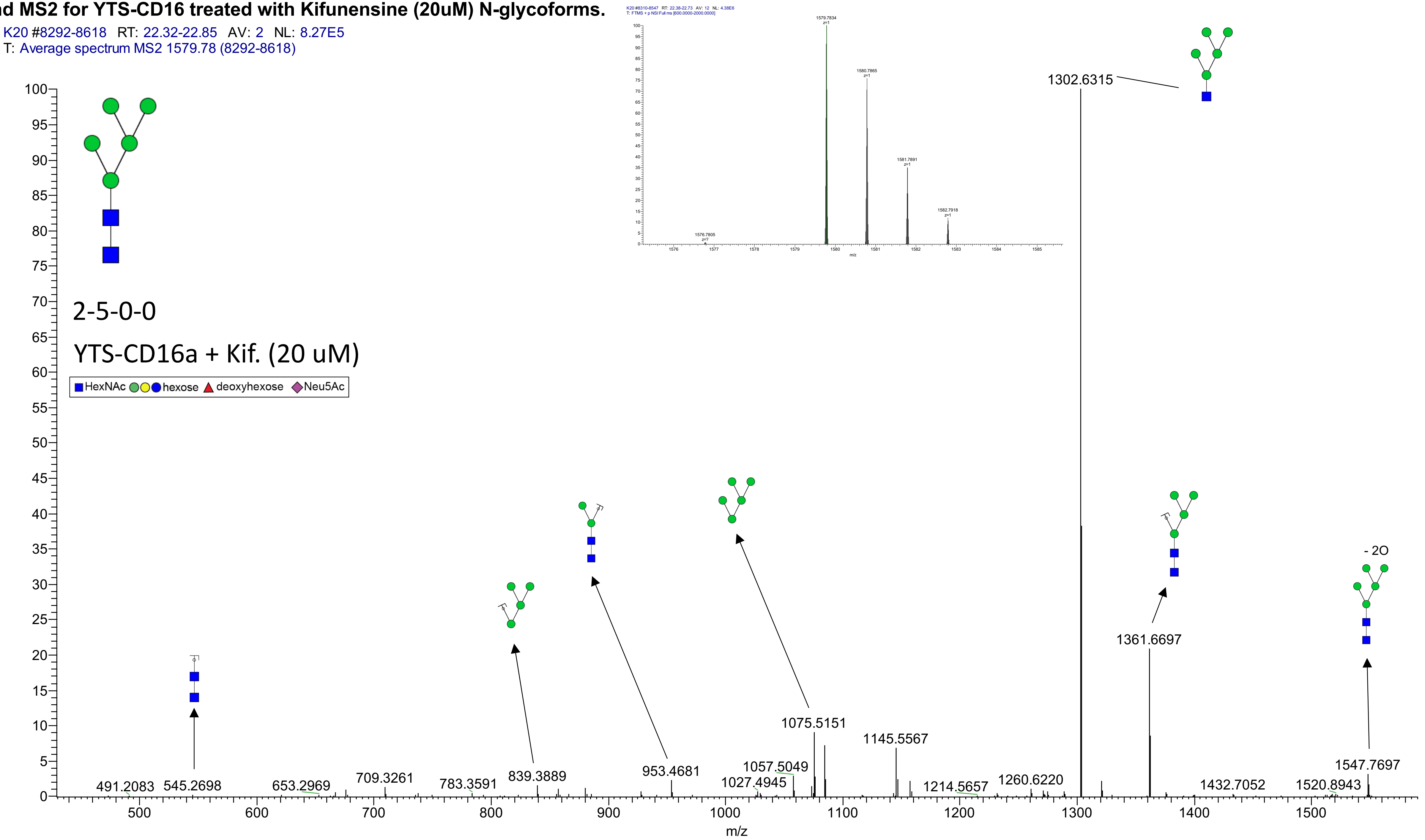

MS1 and MS2 for YTS-CD16 treated with Kifunensine (20uM) N-glycoforms.

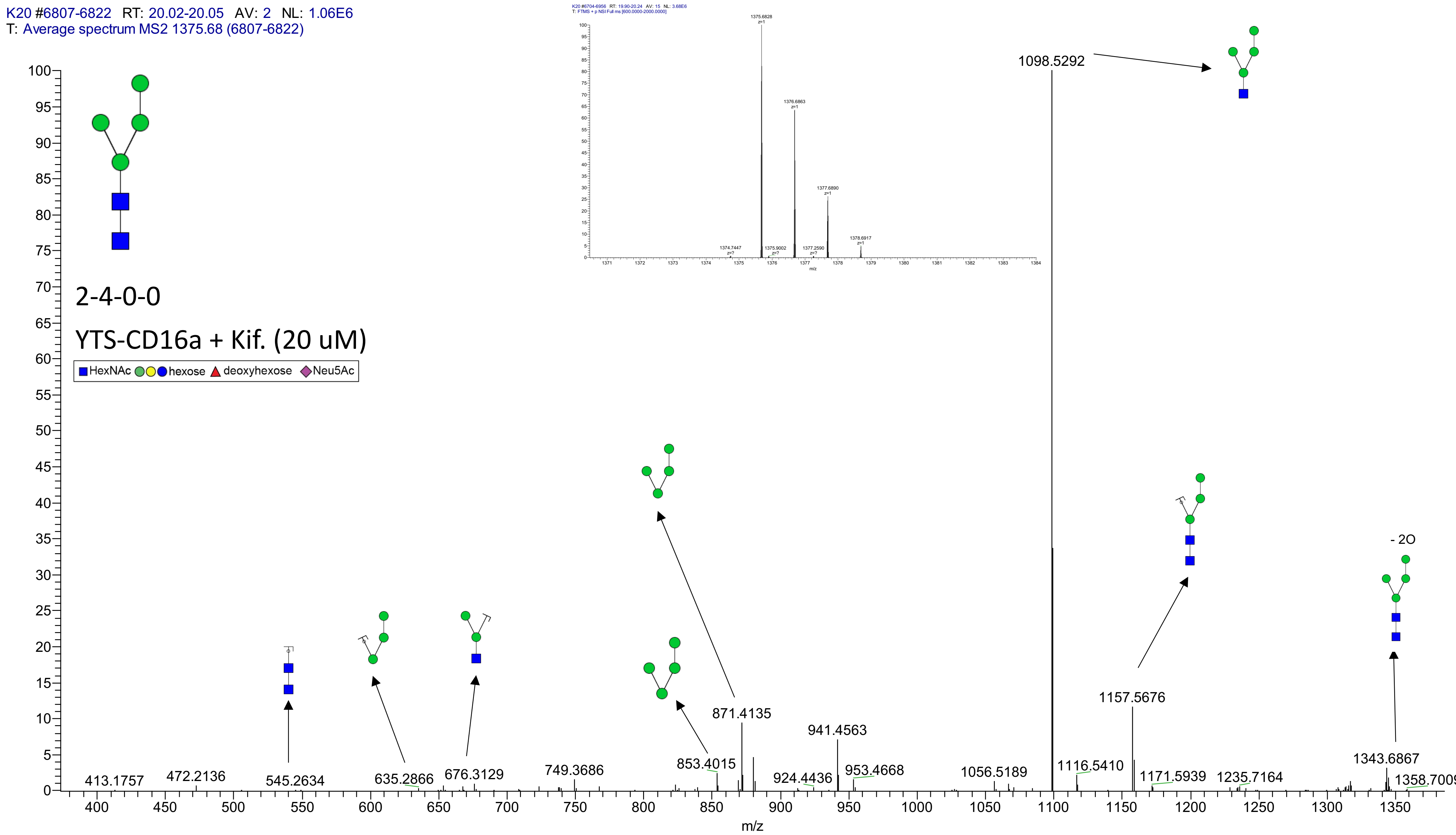

MS1 and MS2 for YTS-CD16 treated with Kifunensine (20uM) N-glycoforms.

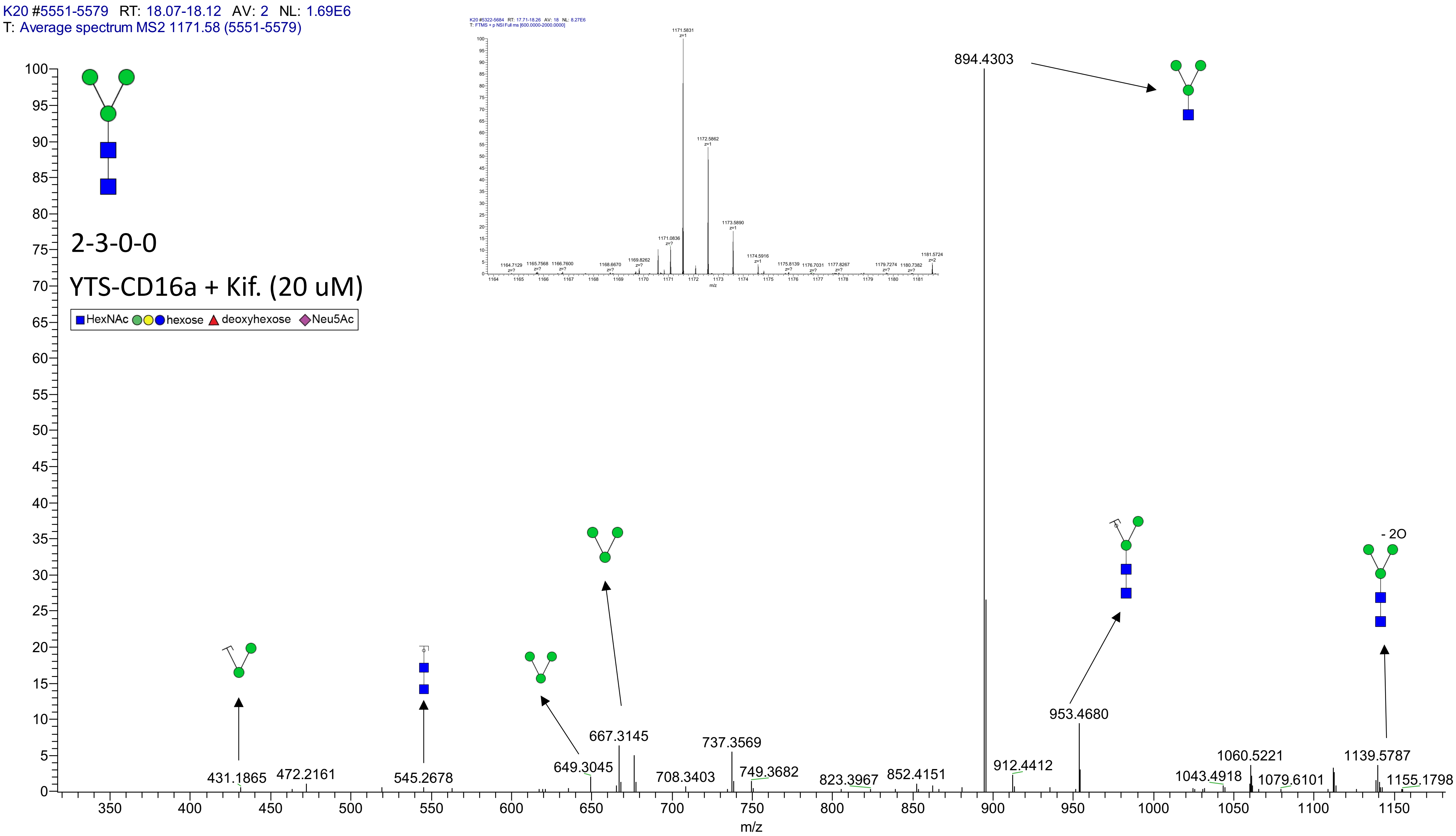

MS1 and MS2 for YTS-CD16 treated with Kifunensine (20uM) N-glycoforms.

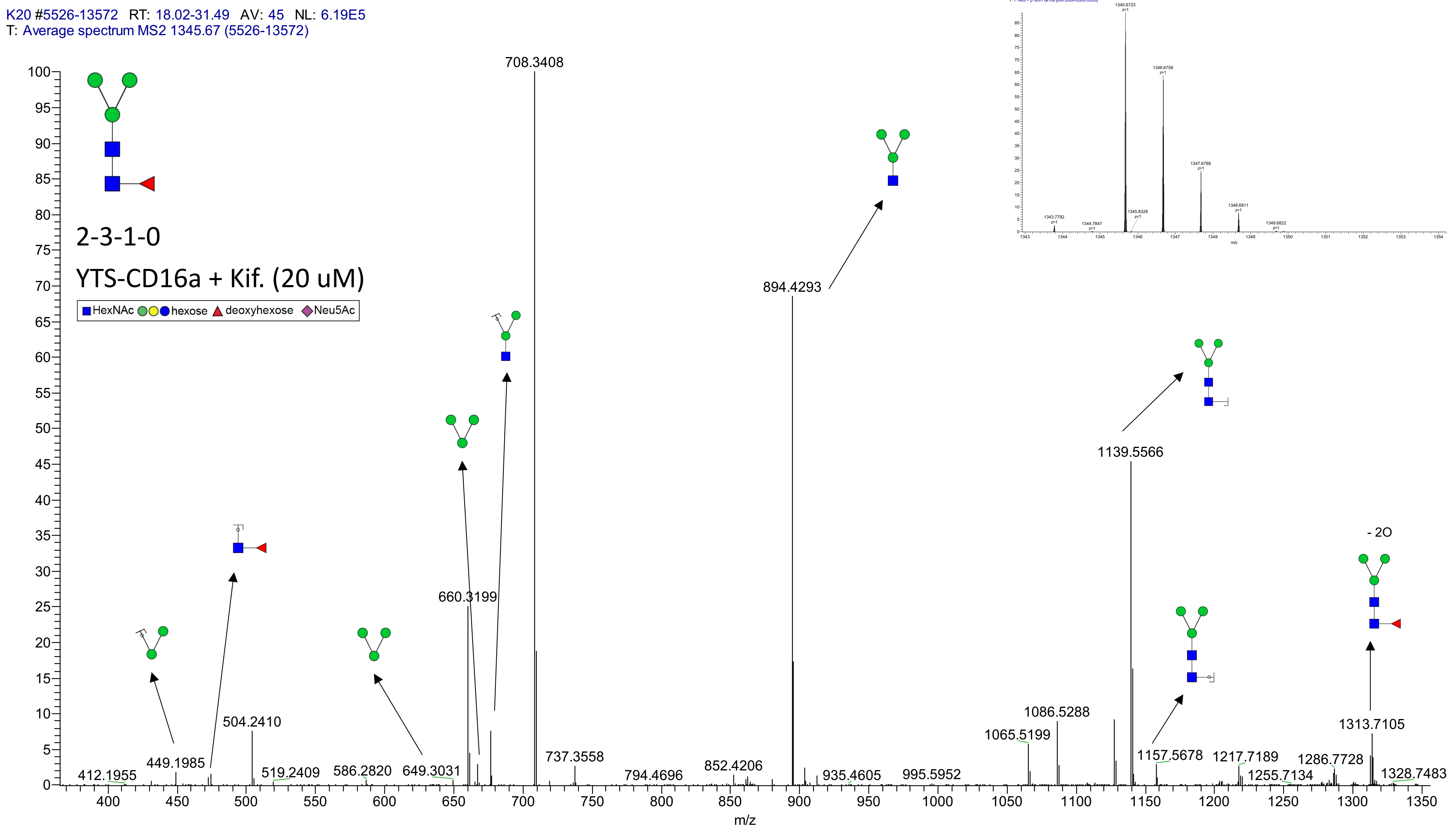

MS1 and MS2 for YTS-CD16 treated with Kifunensine (20uM) N-glycoforms.

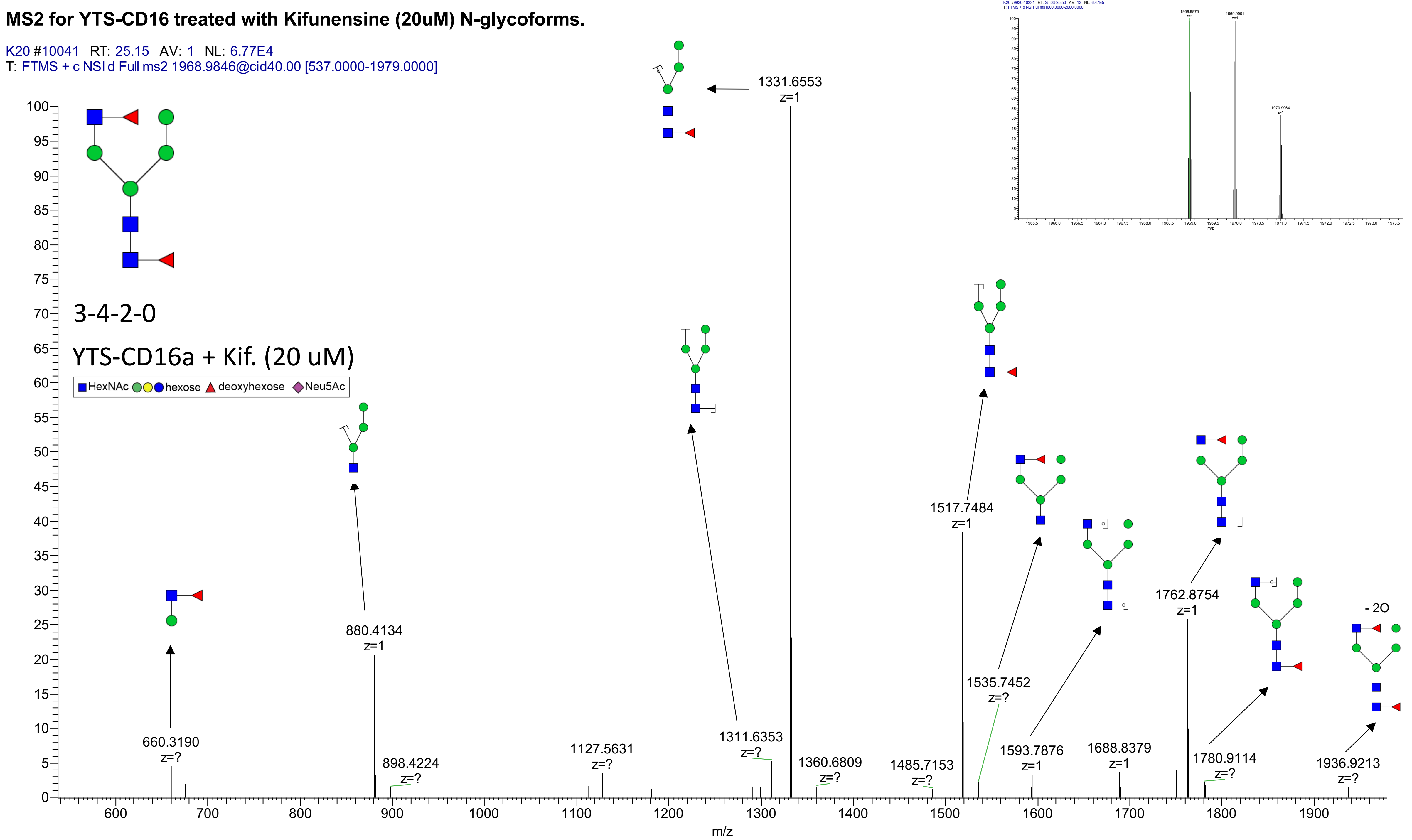

MS1 and MS2 for YTS-CD16 treated with Kifunensine (20uM) N-glycoforms.

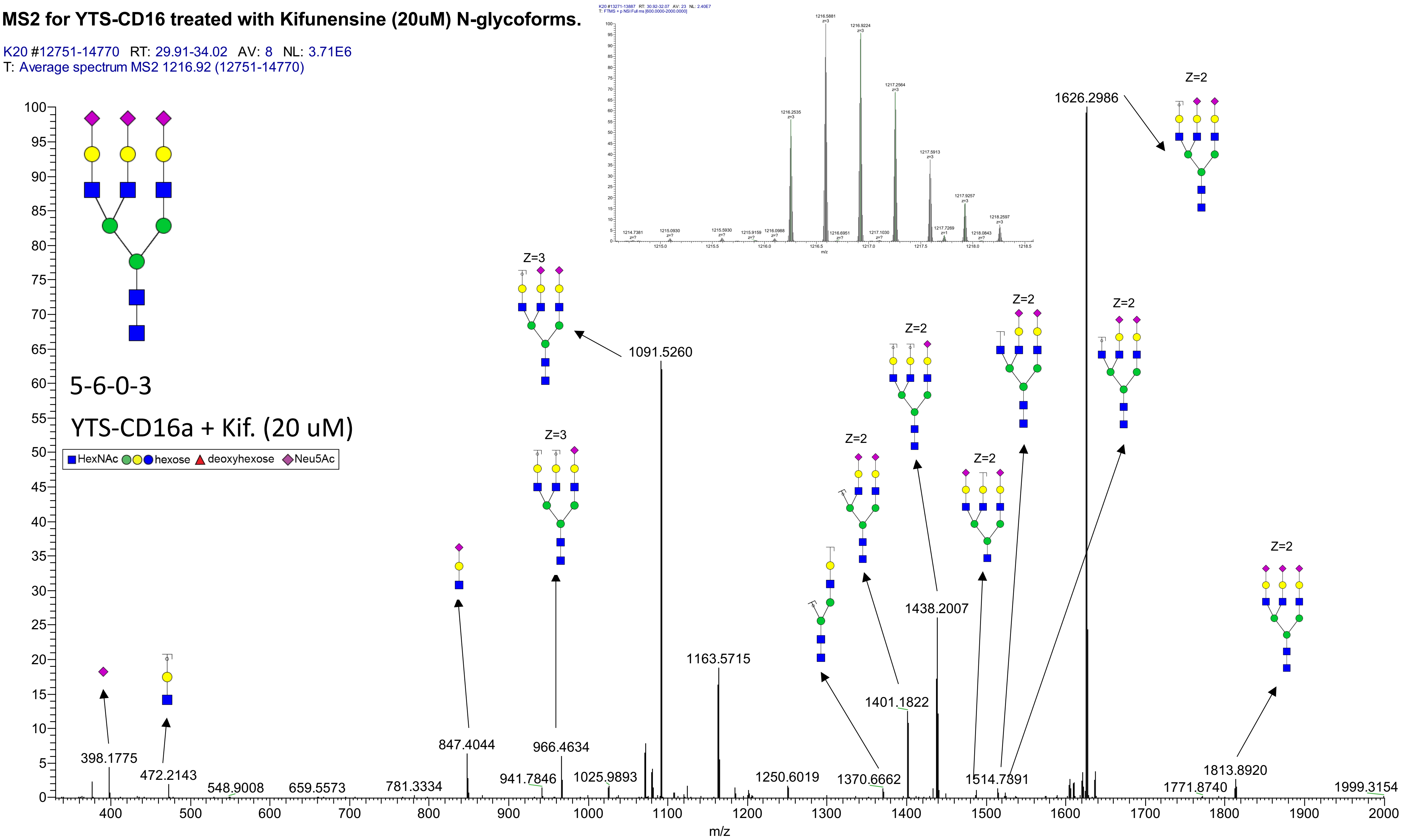

MS1 and MS2 for YTS-CD16 treated with Kifunensine (20uM) N-glycoforms.

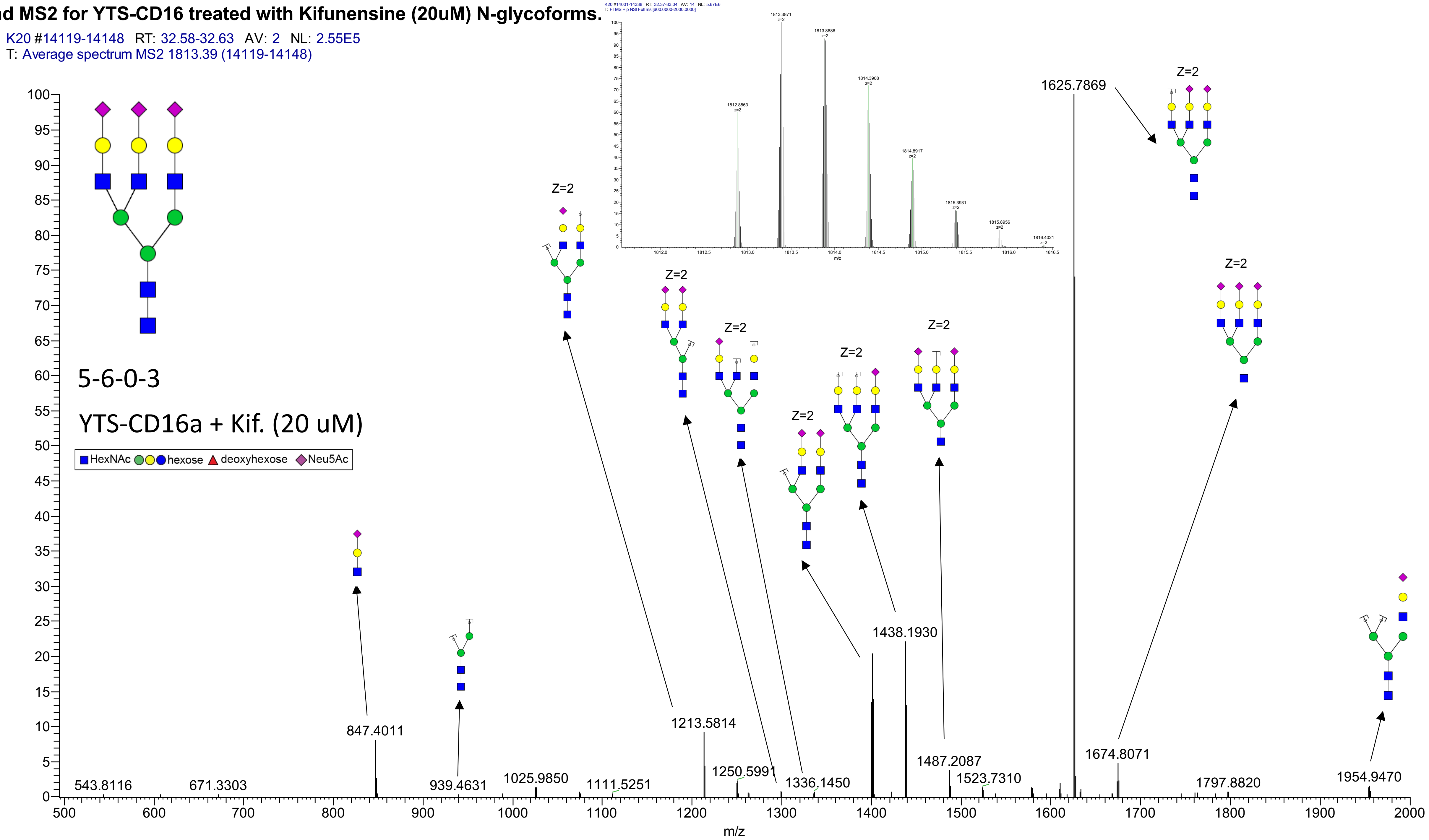

MS1 and MS2 for YTS-CD16 treated with Kifunensine (20uM) N-glycoforms.

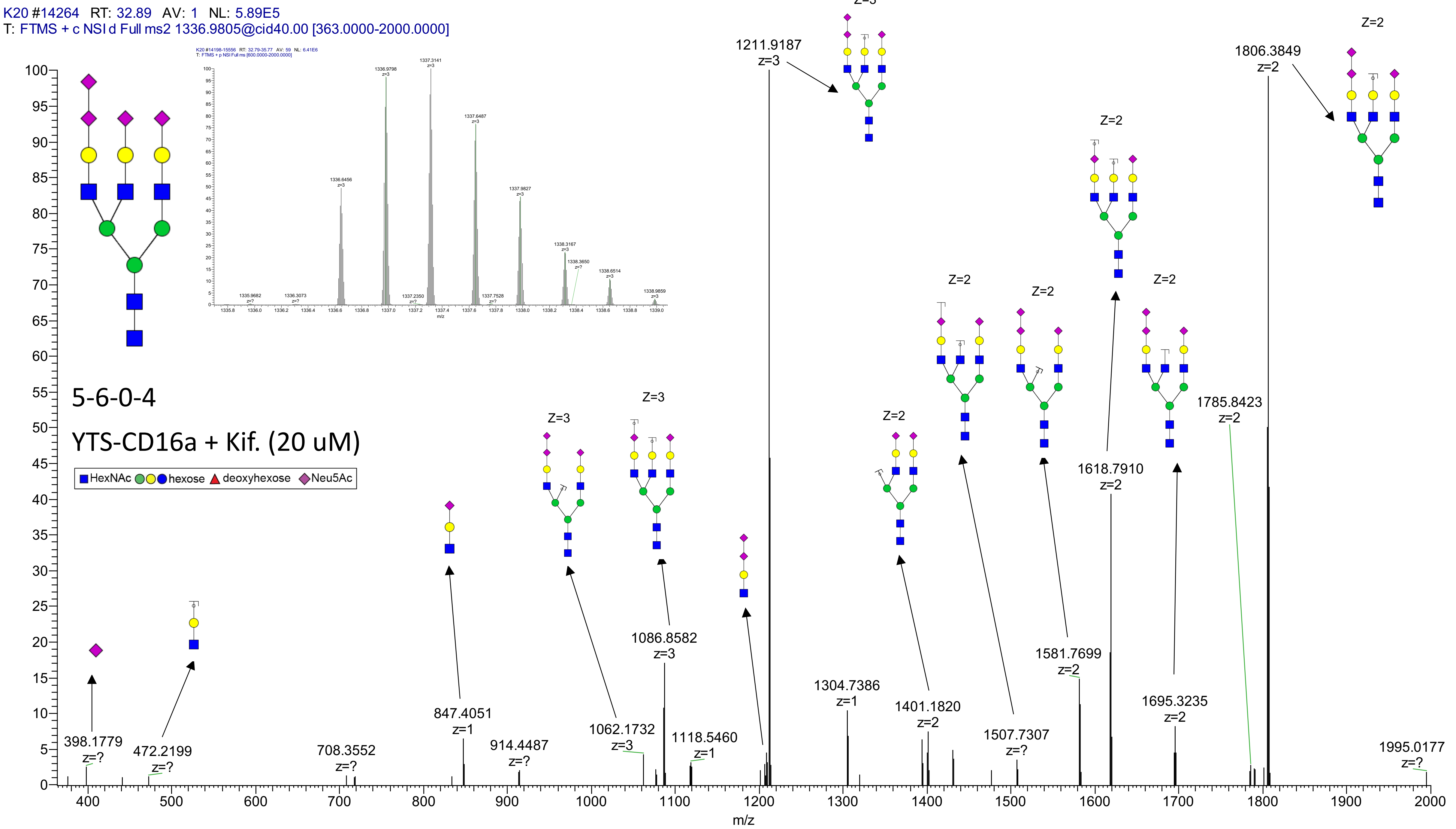

MS1 and MS2 for YTS-CD16 treated with Kifunensine (20uM) N-glycoforms.

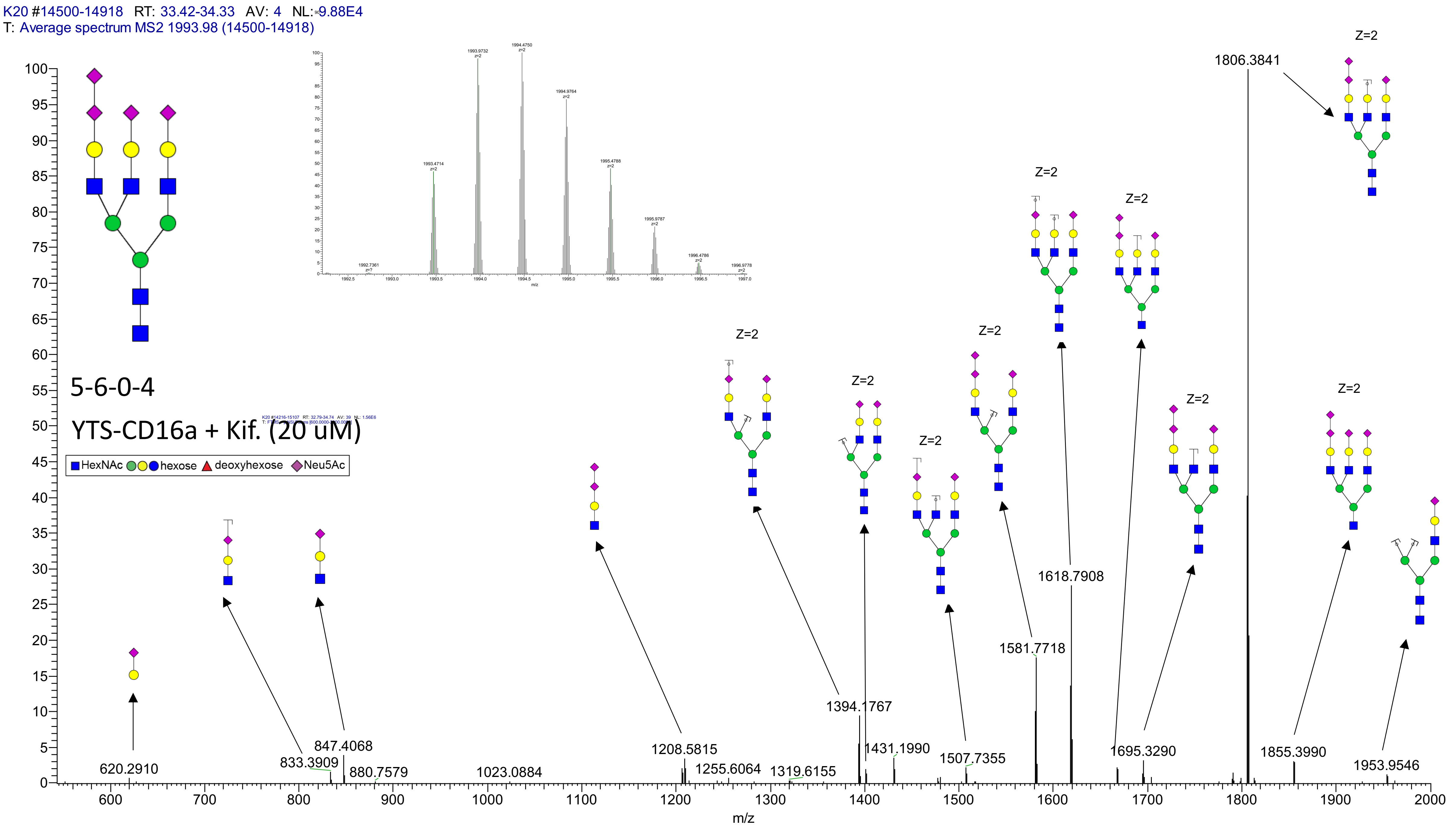

MS1 and MS2 for YTS-CD16 treated with Kifunensine (20uM) N-glycoforms.

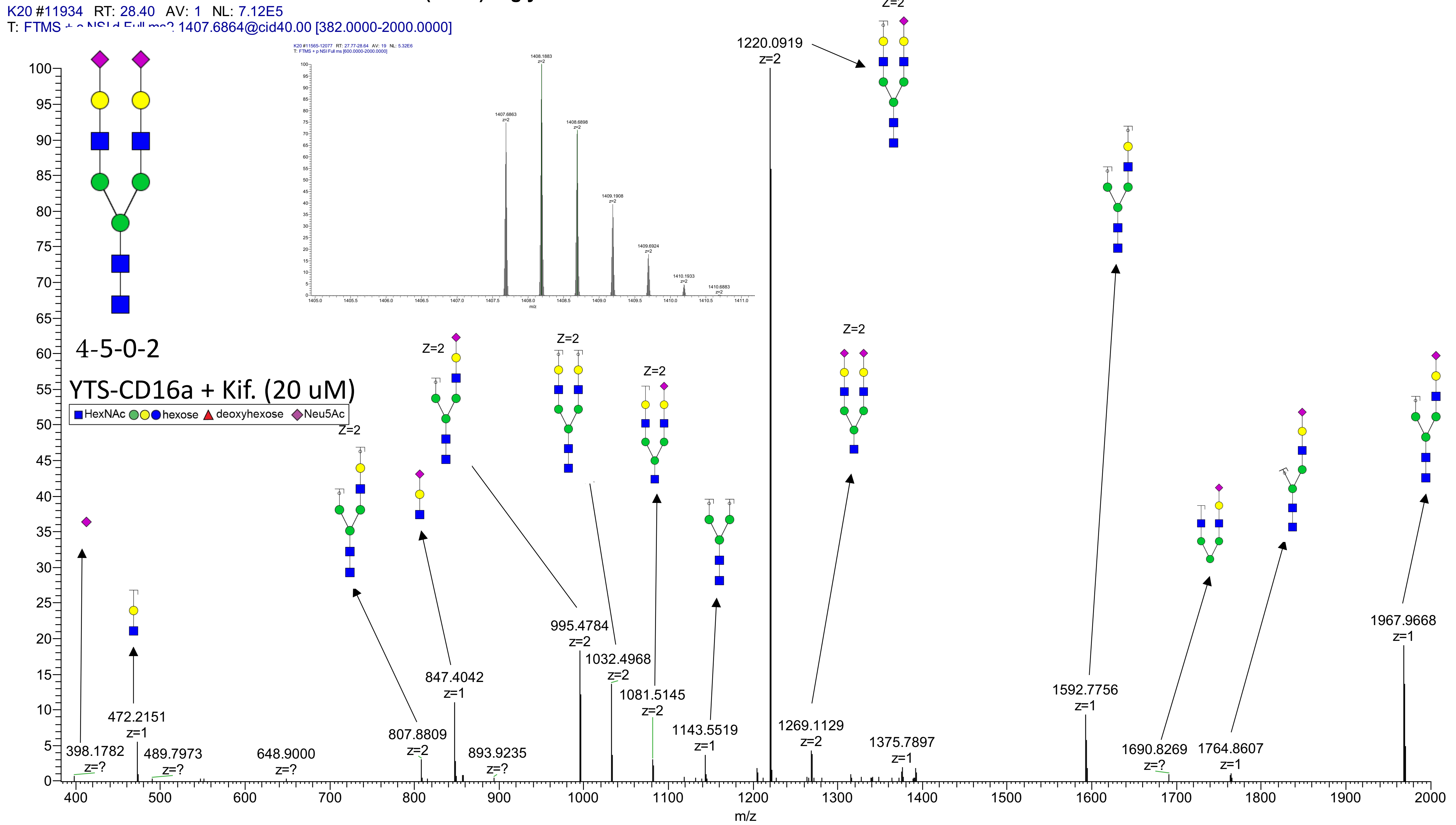

MS1 and MS2 for YTS-CD16 treated with Kifunensine (20uM) N-glycoforms.

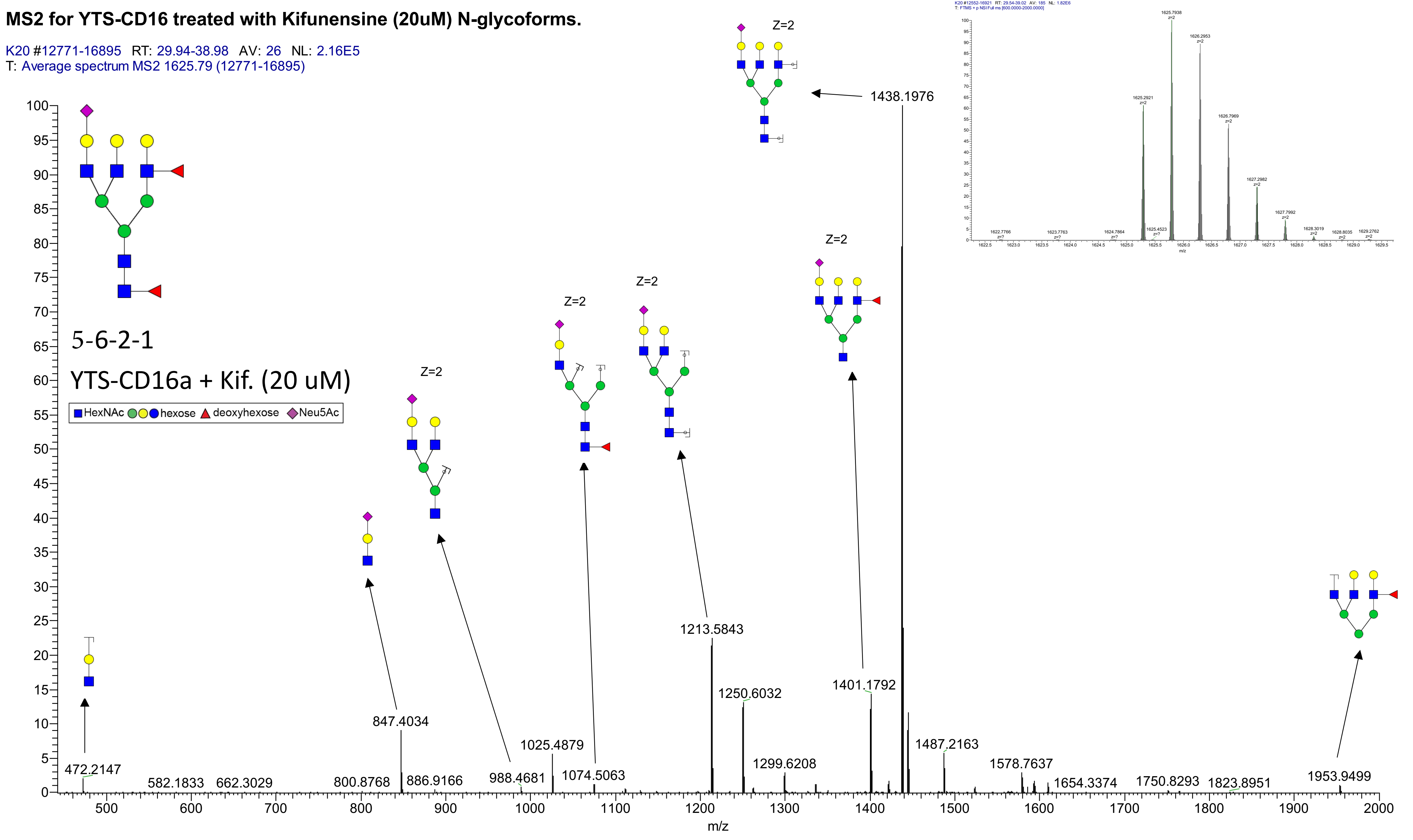

MS1 and MS2 for YTS-CD16 treated with Kifunensine (20uM) N-glycoforms.

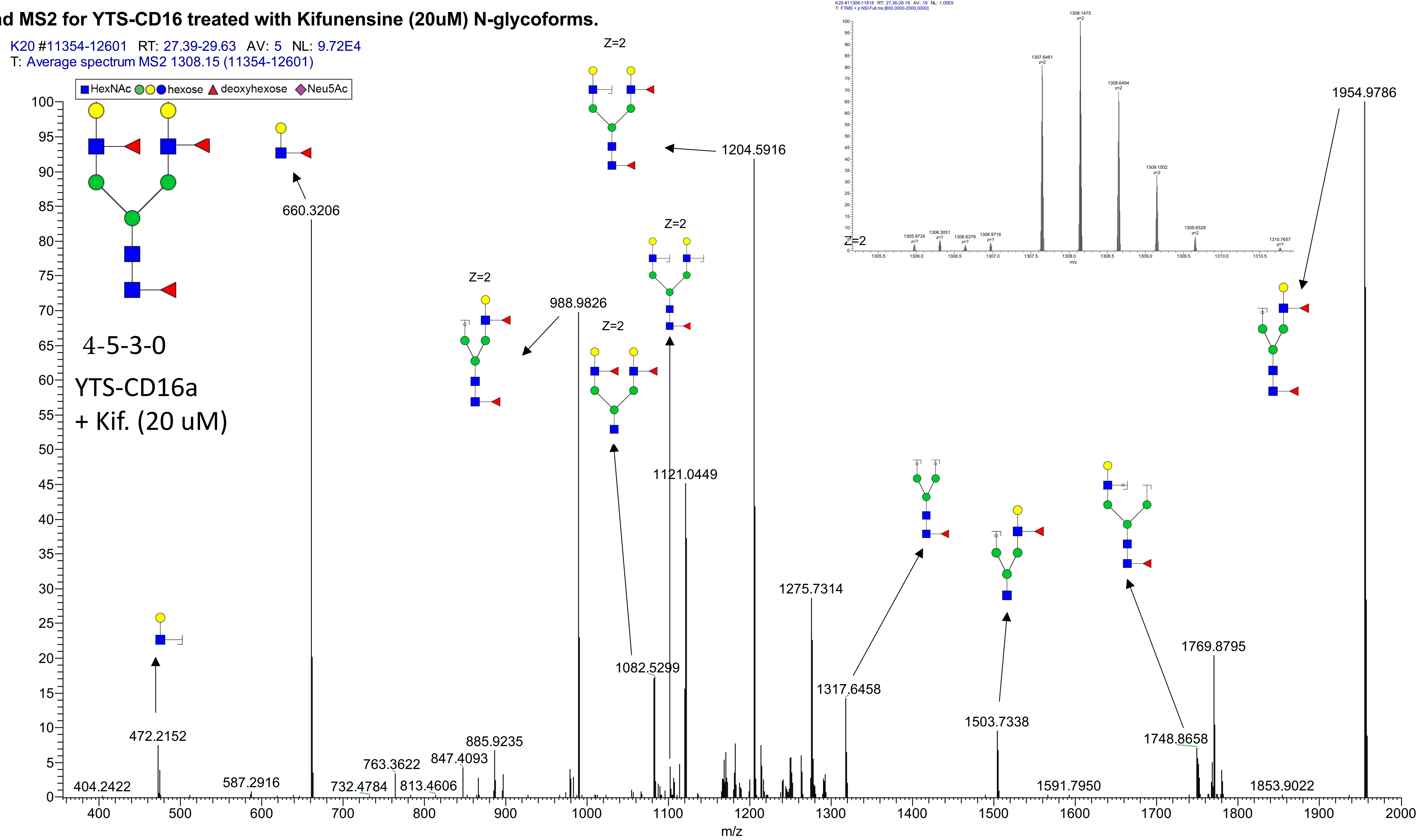

MS1 and MS2 for YTS-CD16 treated with Kifunensine (20uM) N-glycoforms.

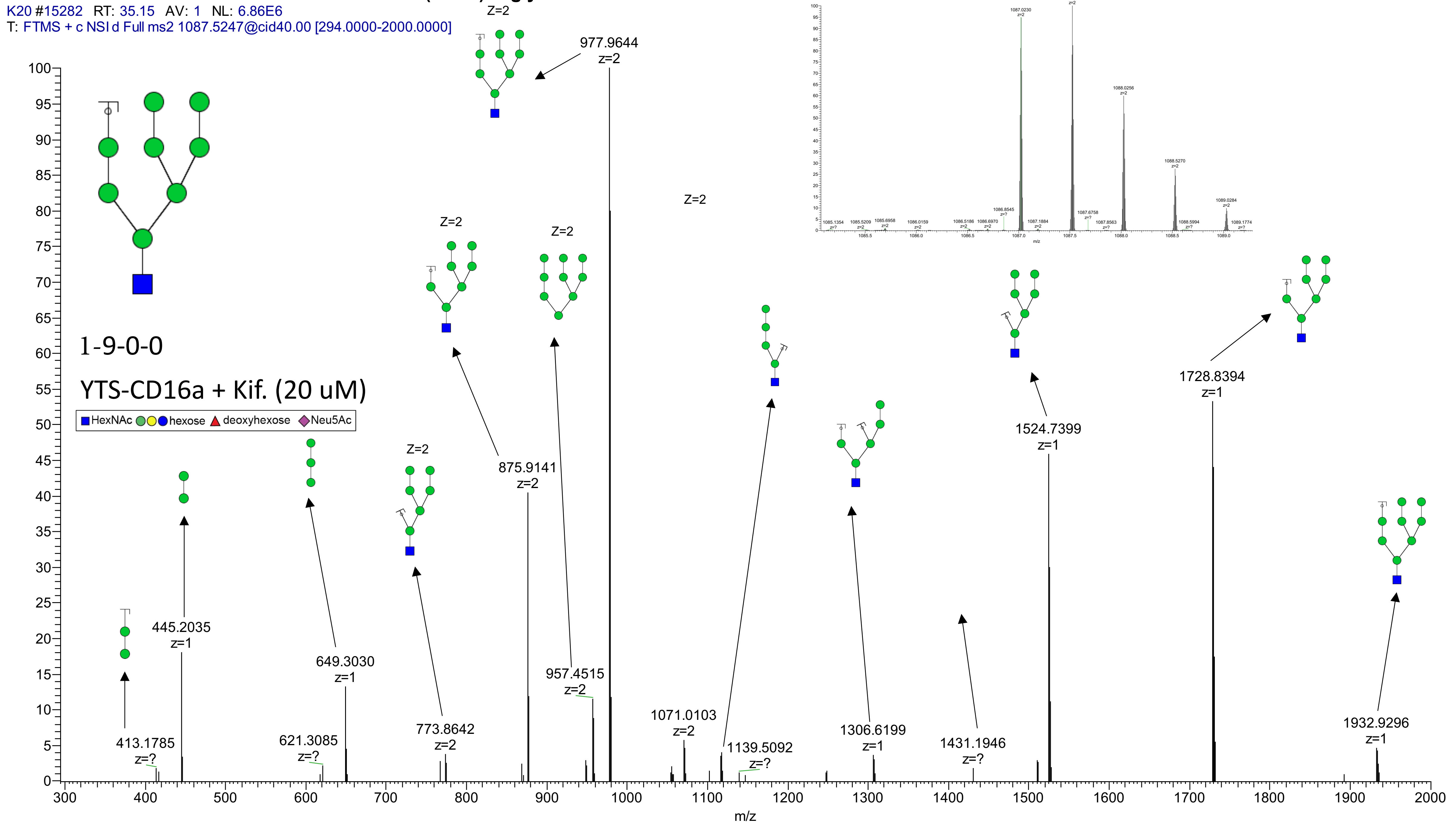

MS1 and MS2 for YTS-CD16 treated with Kifunensine (100uM) N-glycoforms.

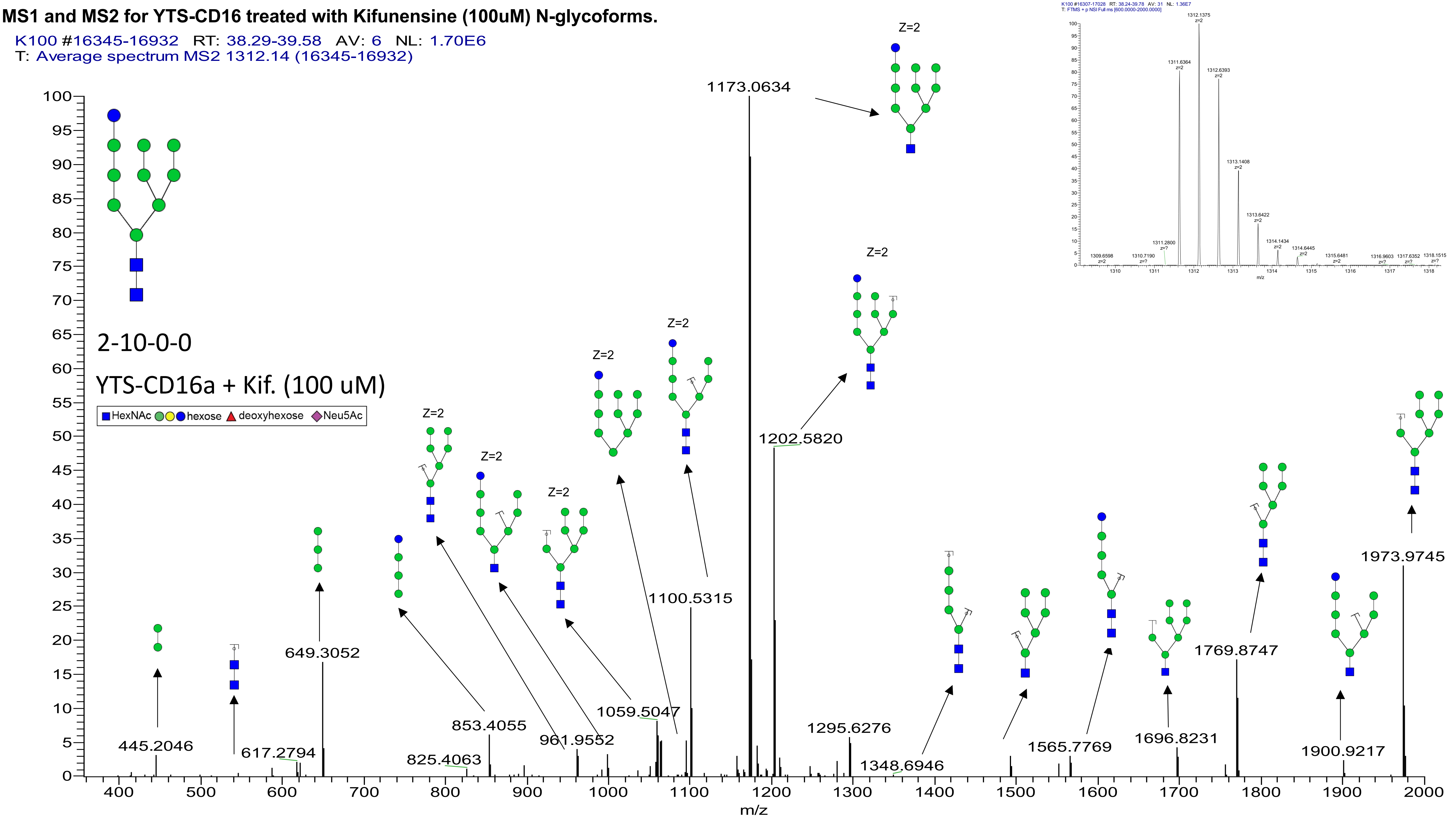

MS1 and MS2 for YTS-CD16 treated with Kifunensine (100uM) N-glycoforms.

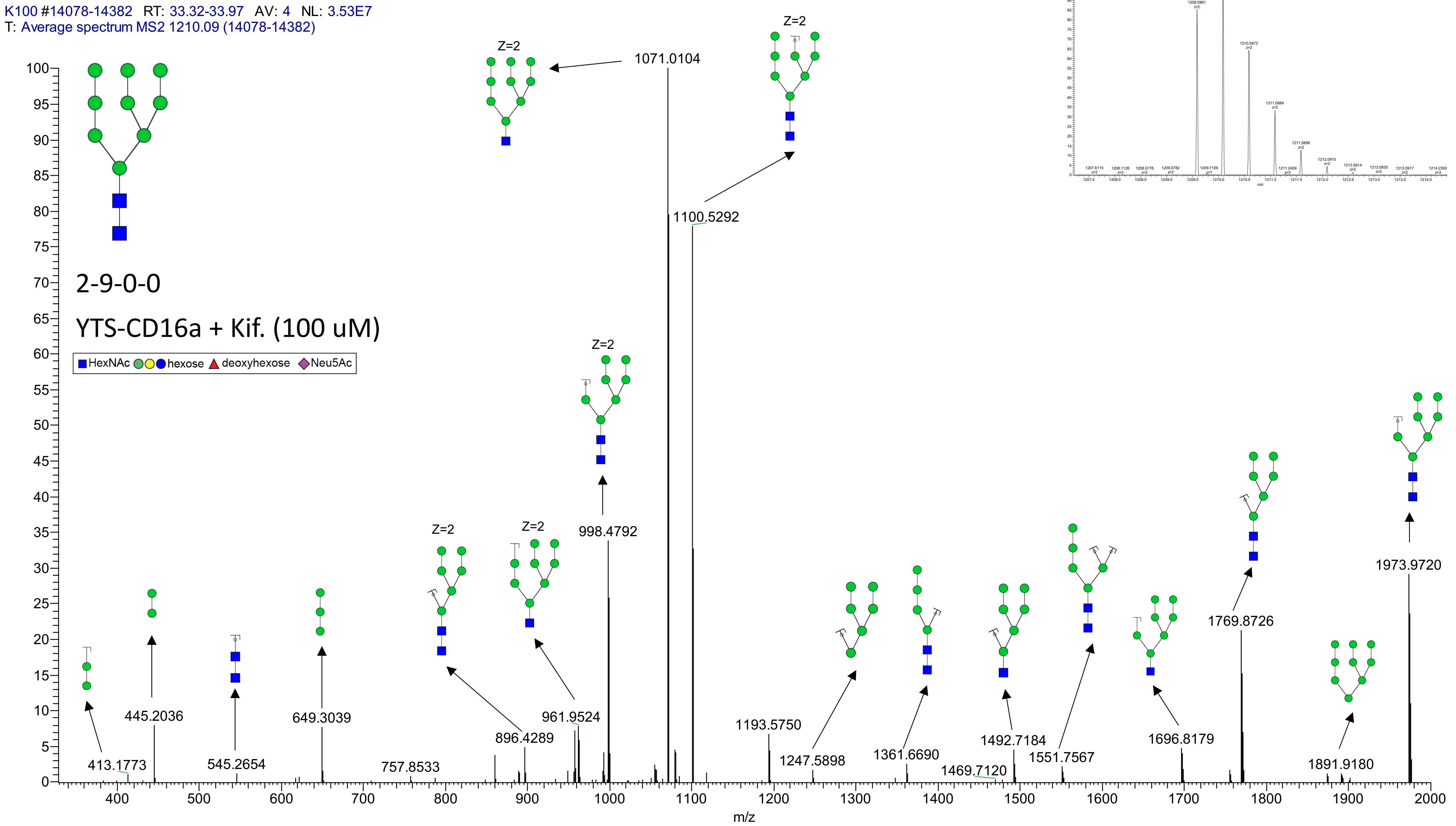

MS1 and MS2 for YTS-CD16 treated with Kifunensine (100uM) N-glycoforms.

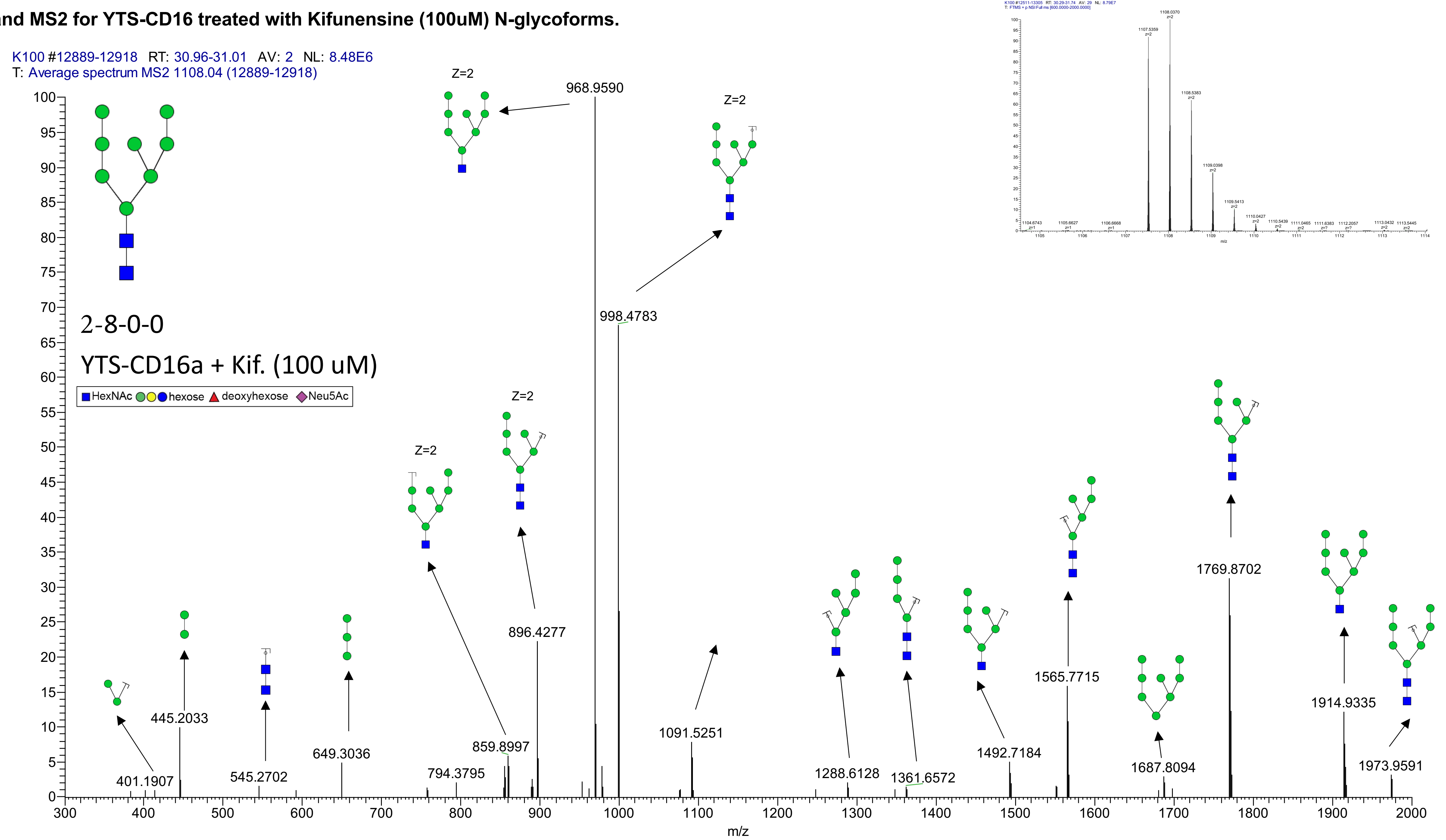

MS1 and MS2 for YTS-CD16 treated with Kifunensine (100uM) N-glycoforms.

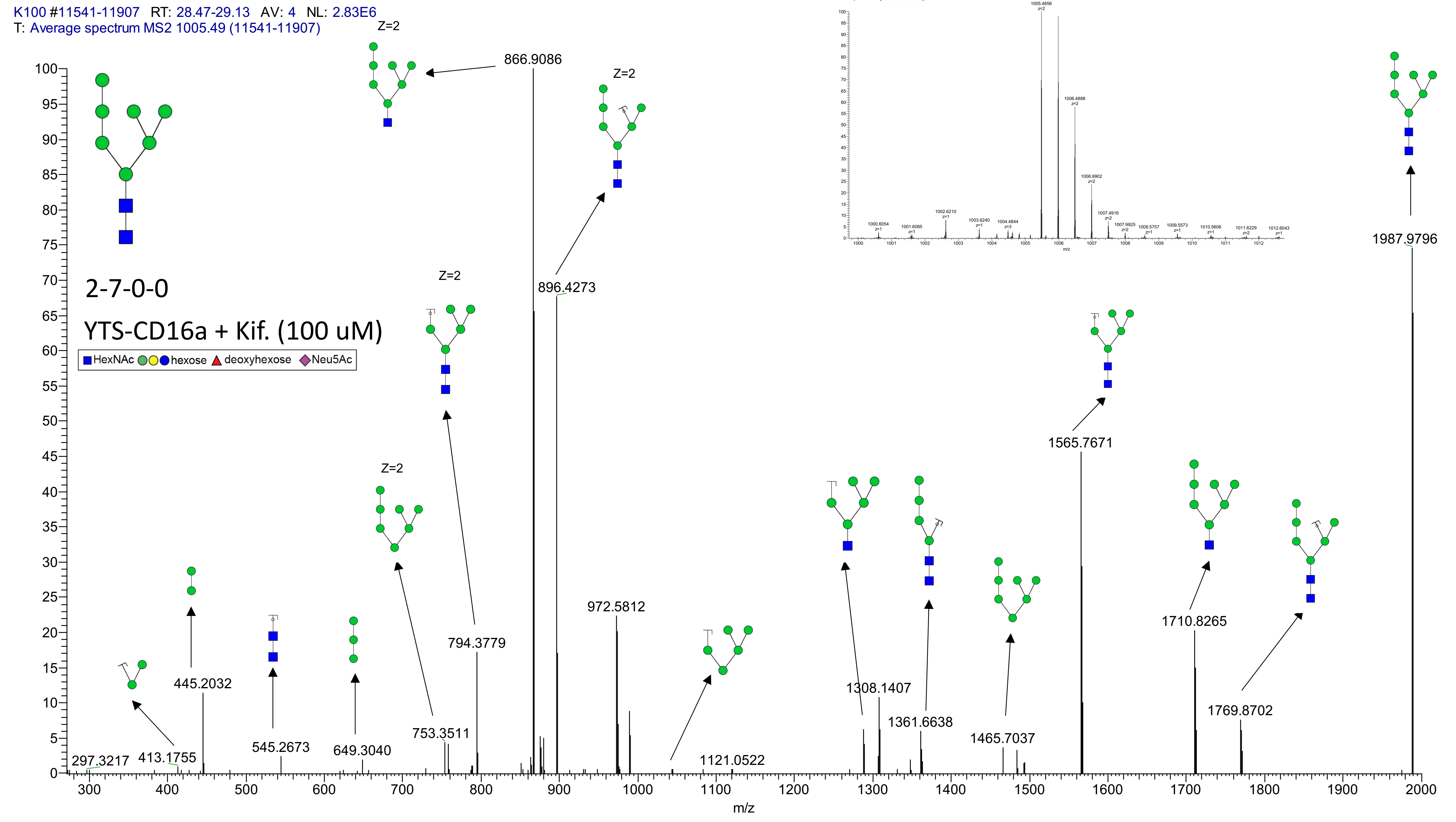

MS1 and MS2 for YTS-CD16 treated with Kifunensine (100uM) N-glycoforms.

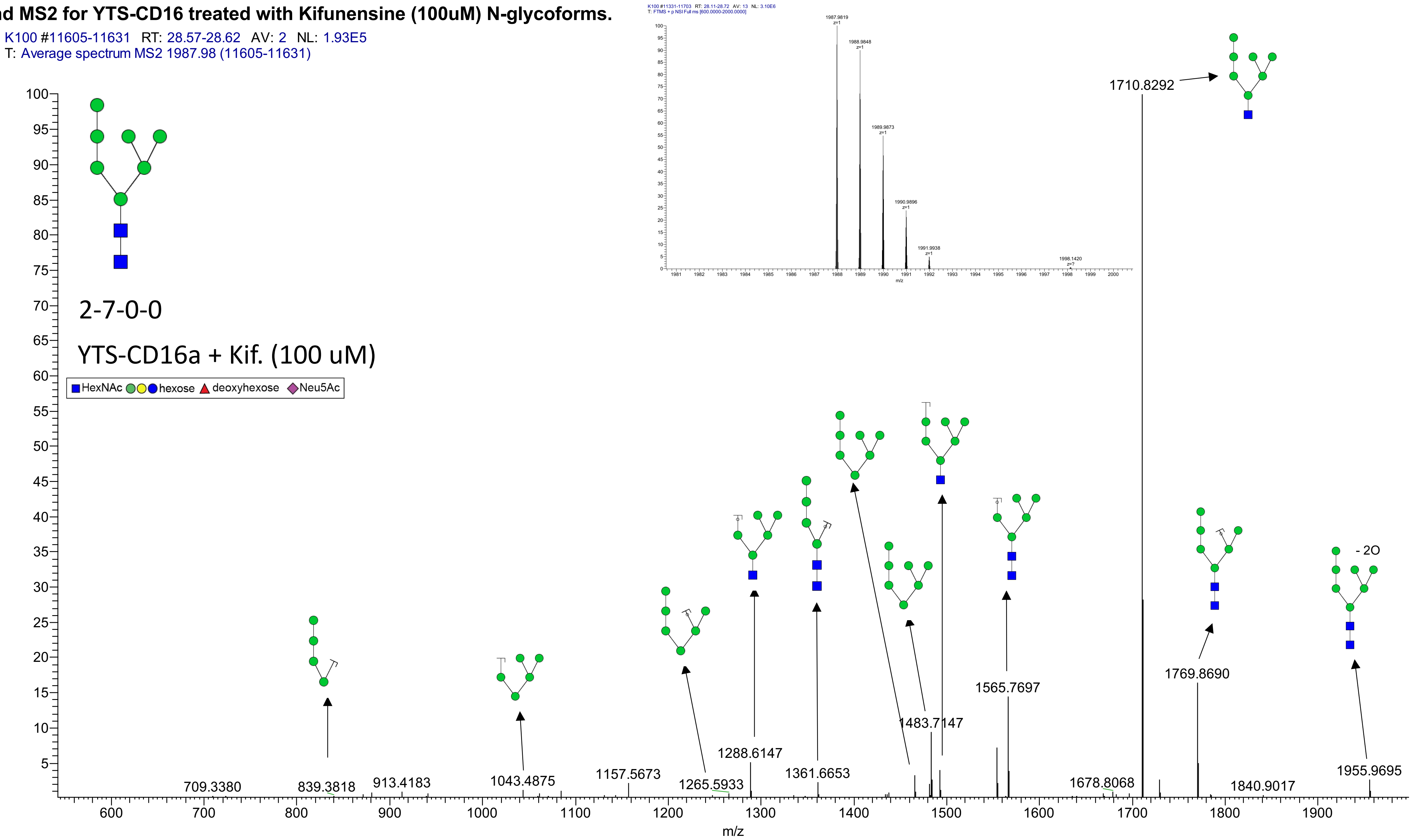

MS1 and MS2 for YTS-CD16 treated with Kifunensine (100uM) N-glycoforms.

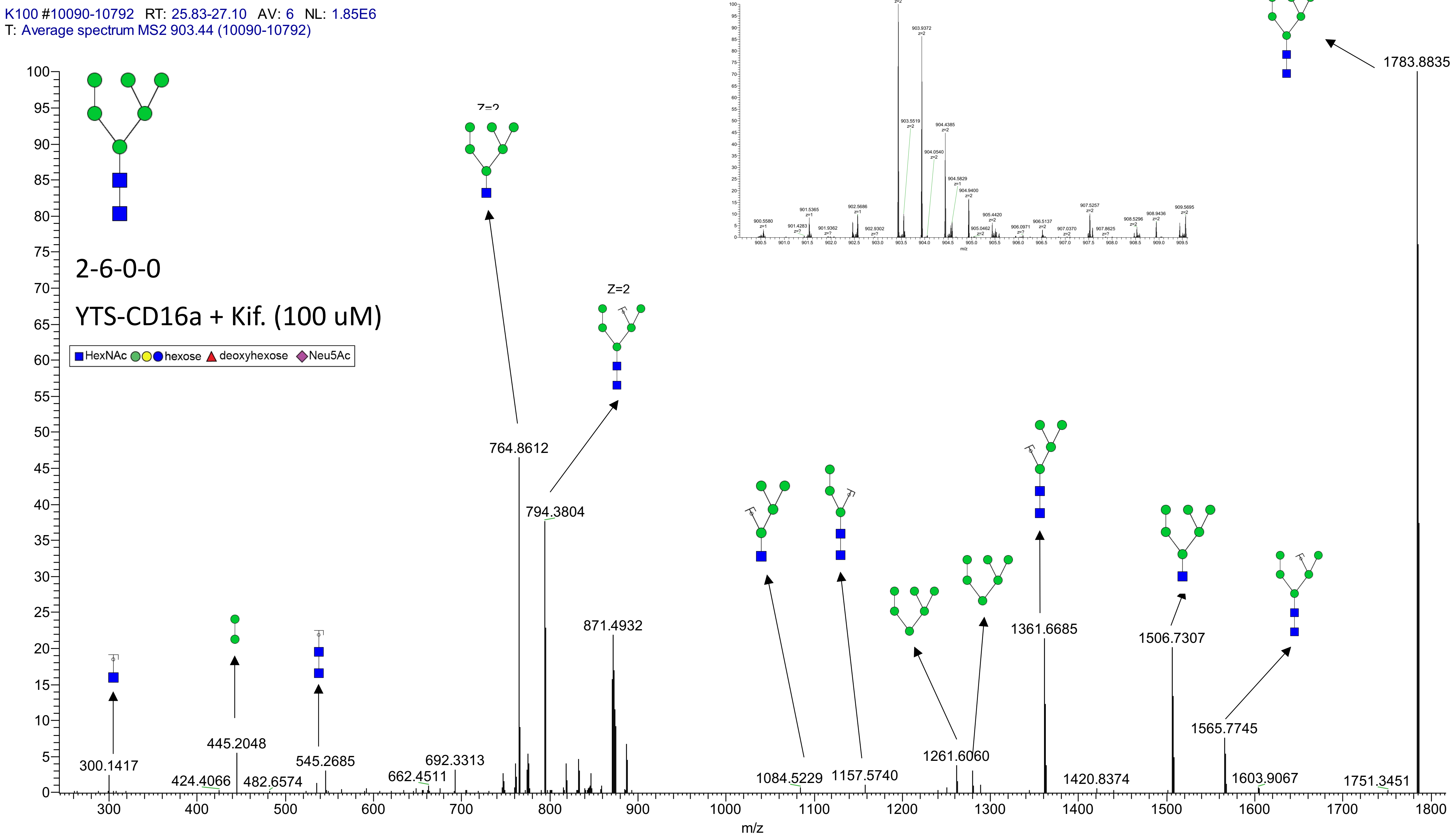

MS1 and MS2 for YTS-CD16 treated with Kifunensine (100uM) N-glycoforms.

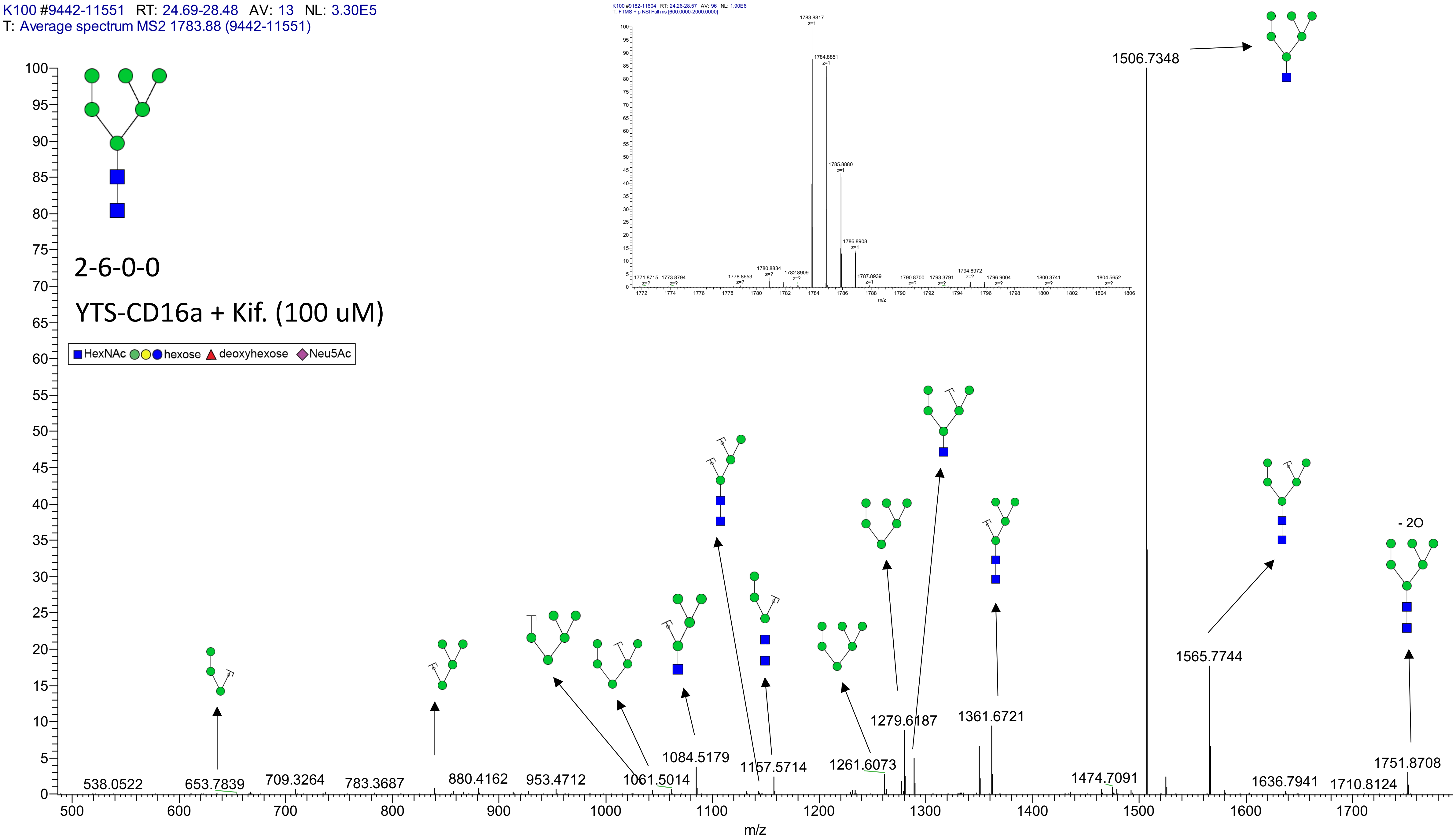

MS1 and MS2 for YTS-CD16 treated with Kifunensine (100uM) N-glycoforms.

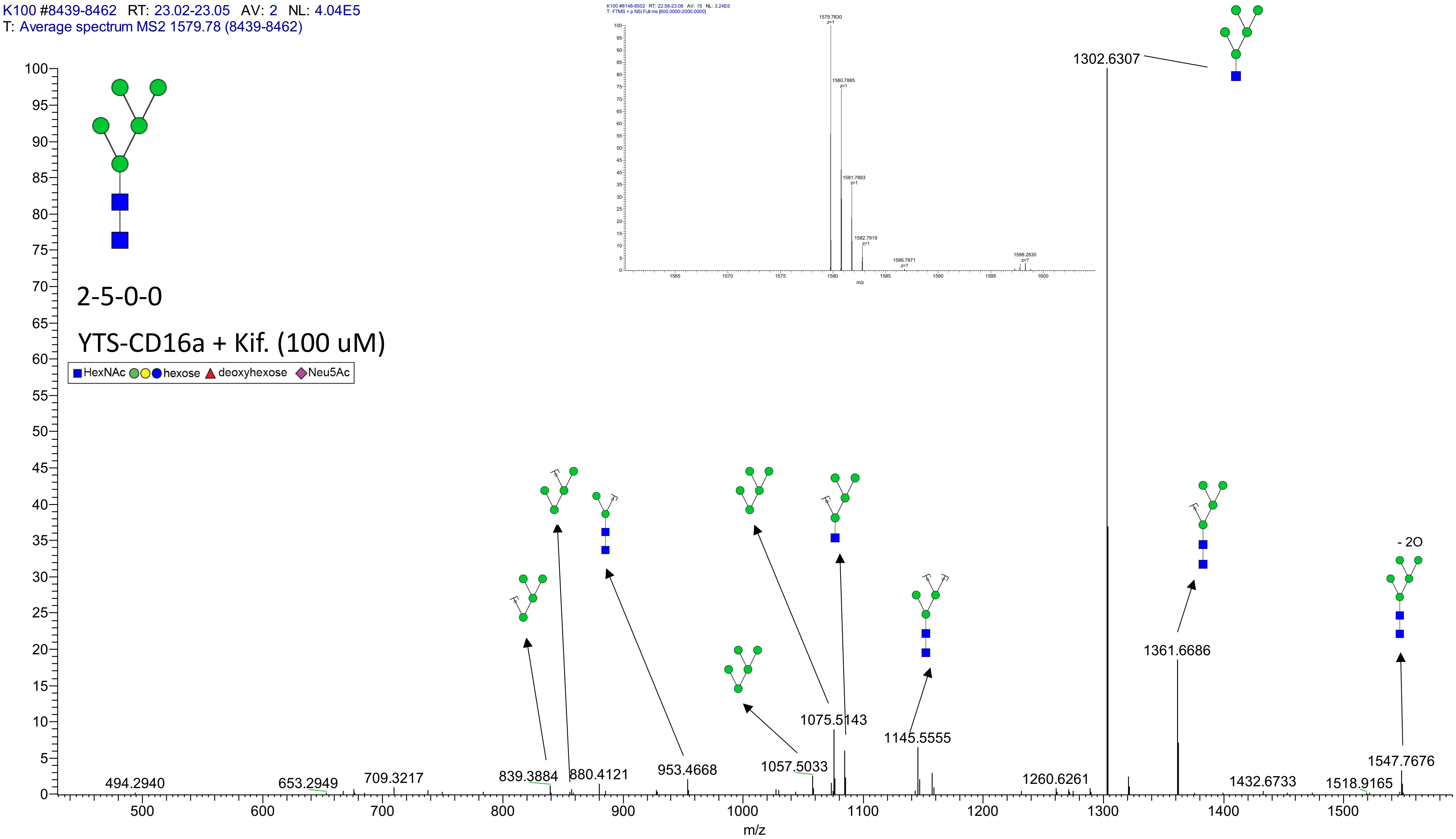

MS1 and MS2 for YTS-CD16 treated with Kifunensine (100uM) N-glycoforms.

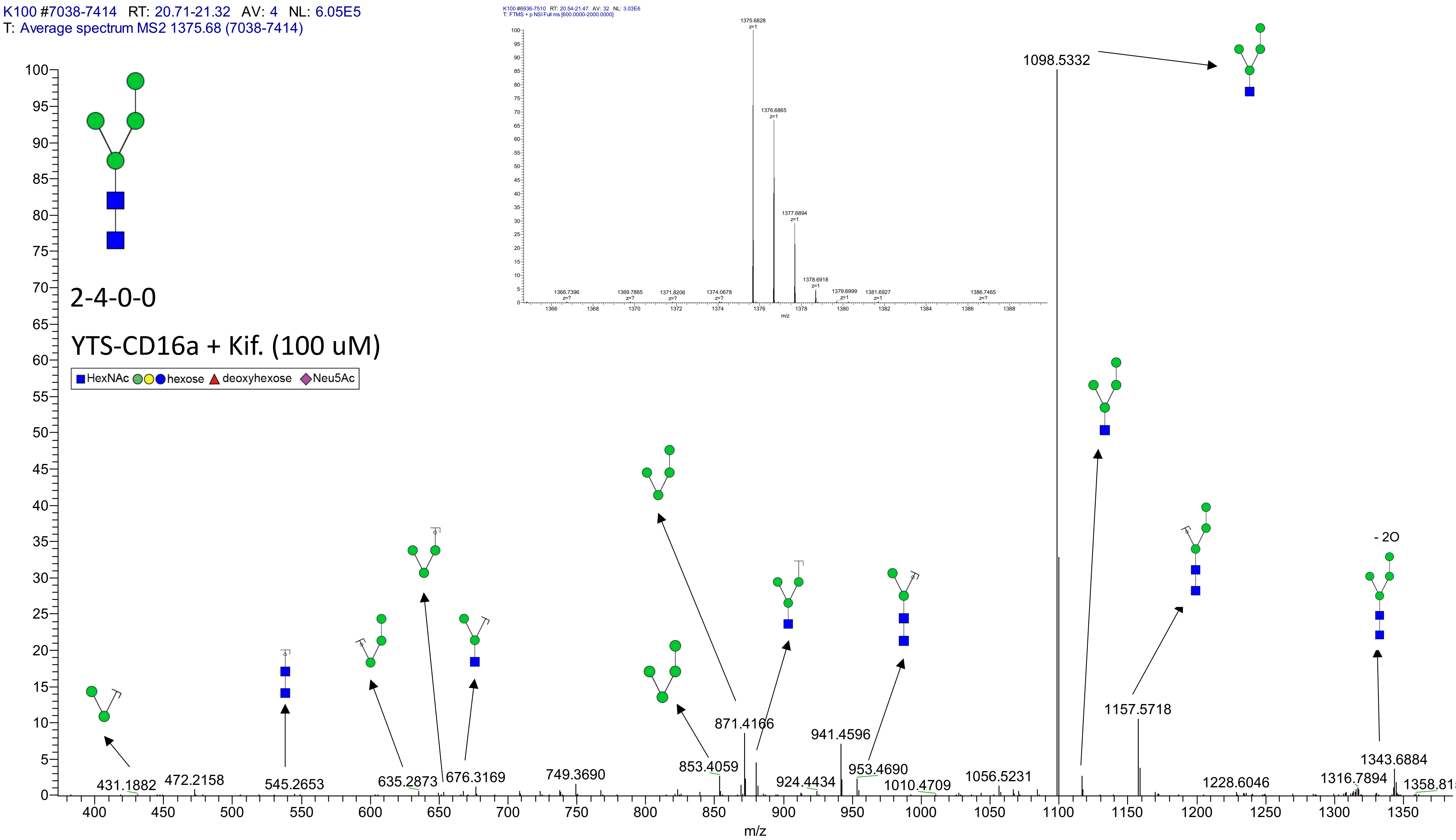

MS1 and MS2 for YTS-CD16 treated with Kifunensine (100uM) N-glycoforms.

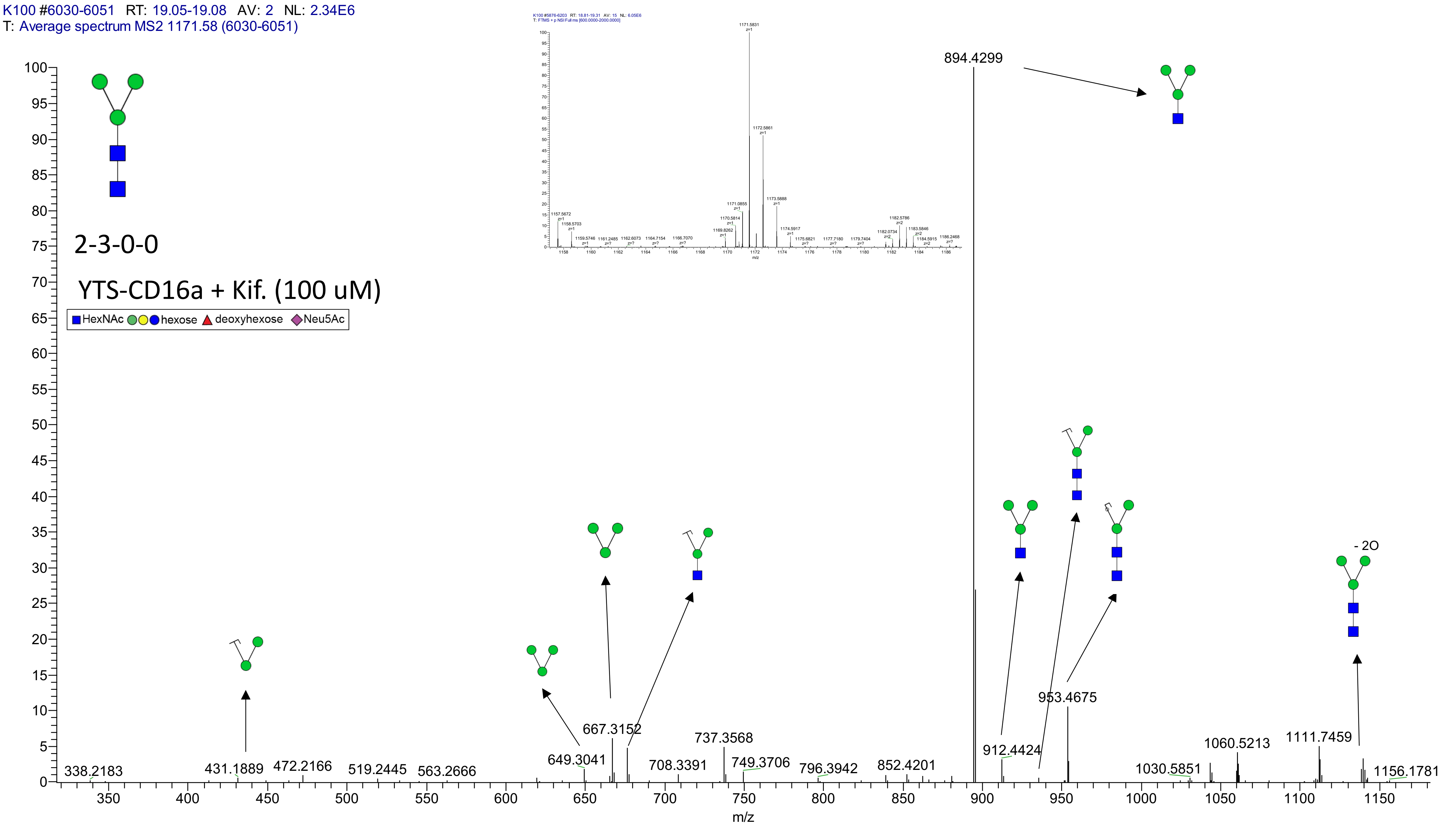

MS1 and MS2 for YTS-CD16 treated with Kifunensine (100uM) N-glycoforms.

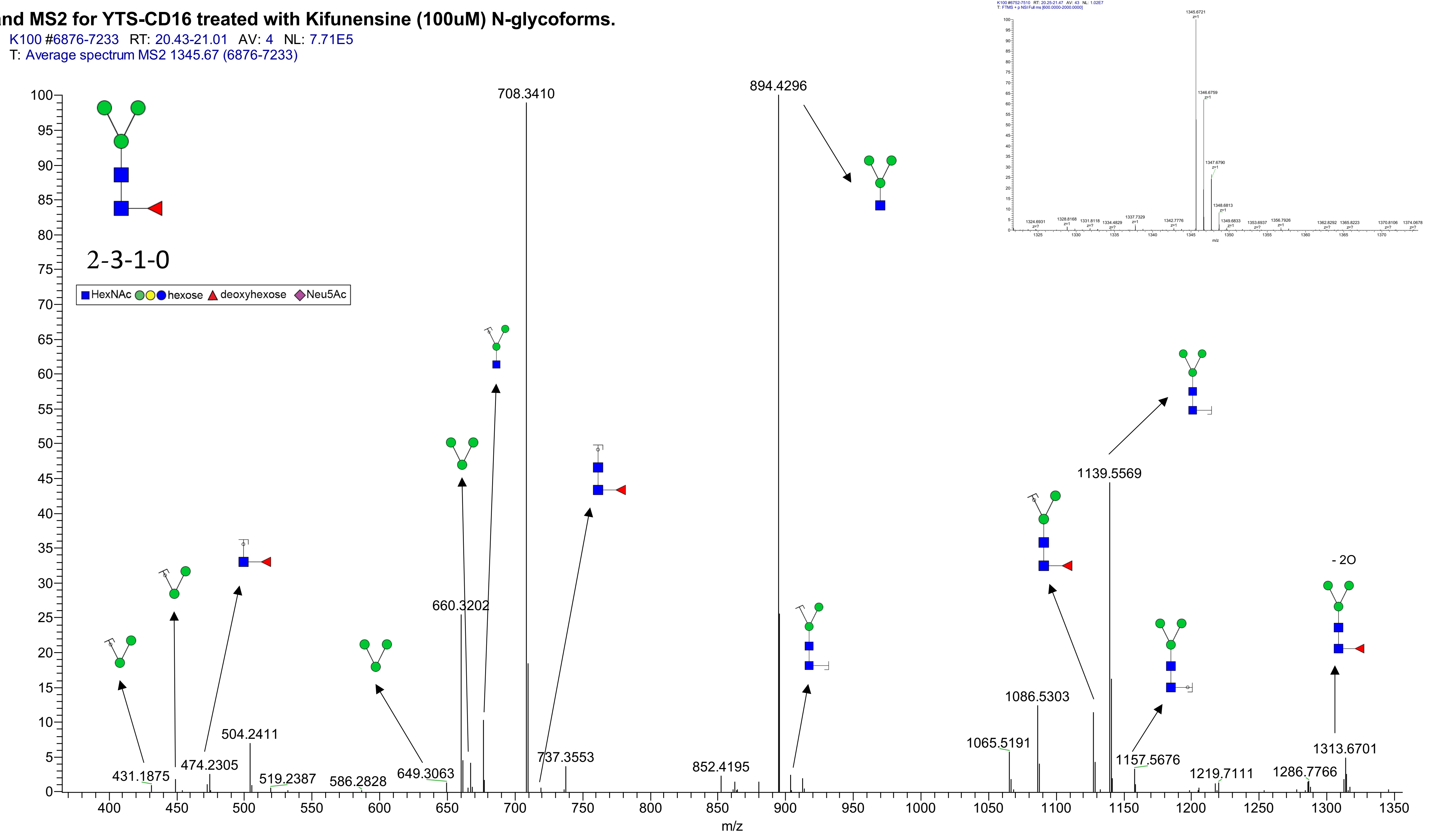

MS1 and MS2 for YTS-CD16 treated with Kifunensine (100uM) N-glycoforms.

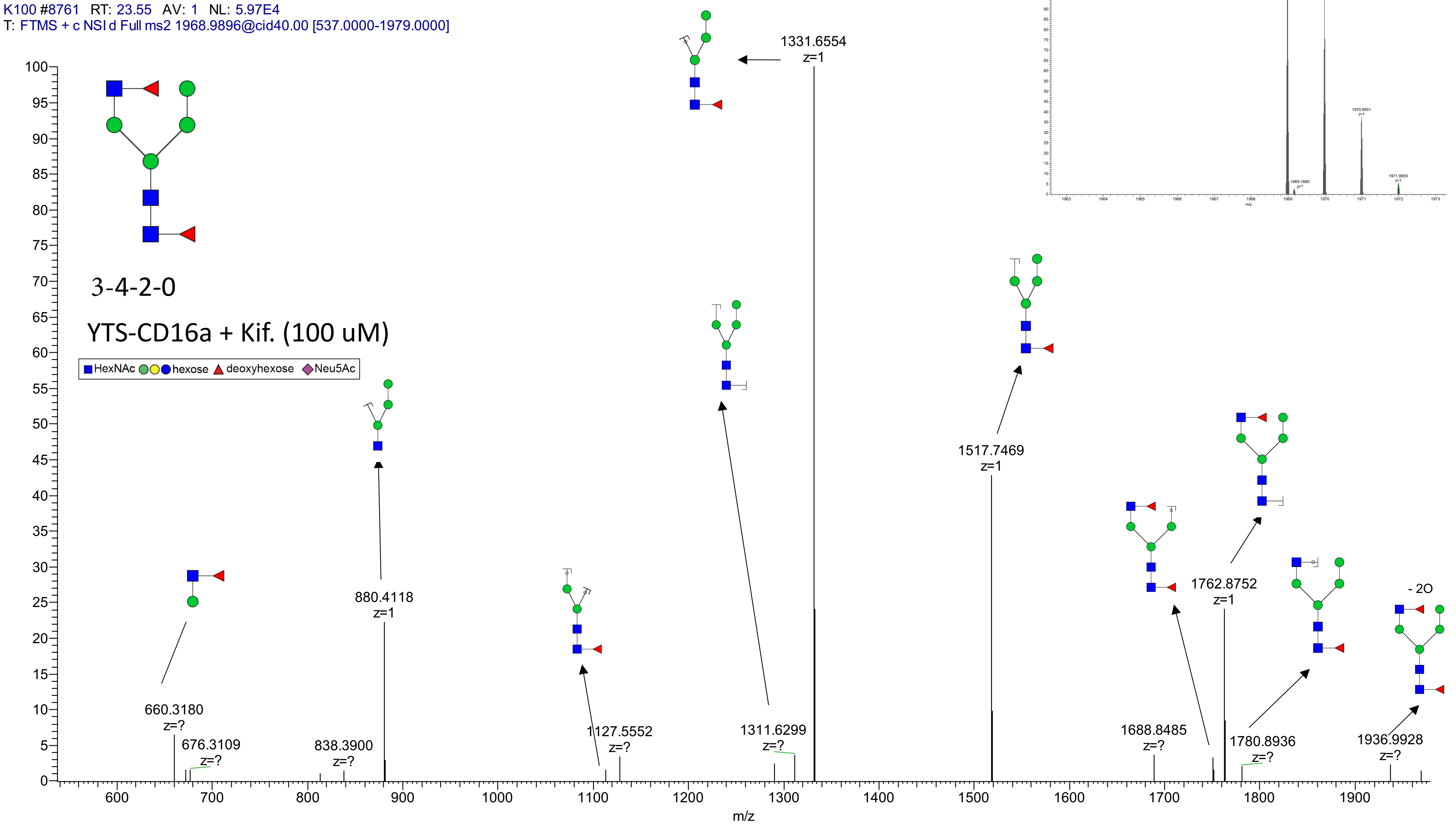

MS1 and MS2 for YTS-CD16 treated with Kifunensine (100uM) N-glycoforms.

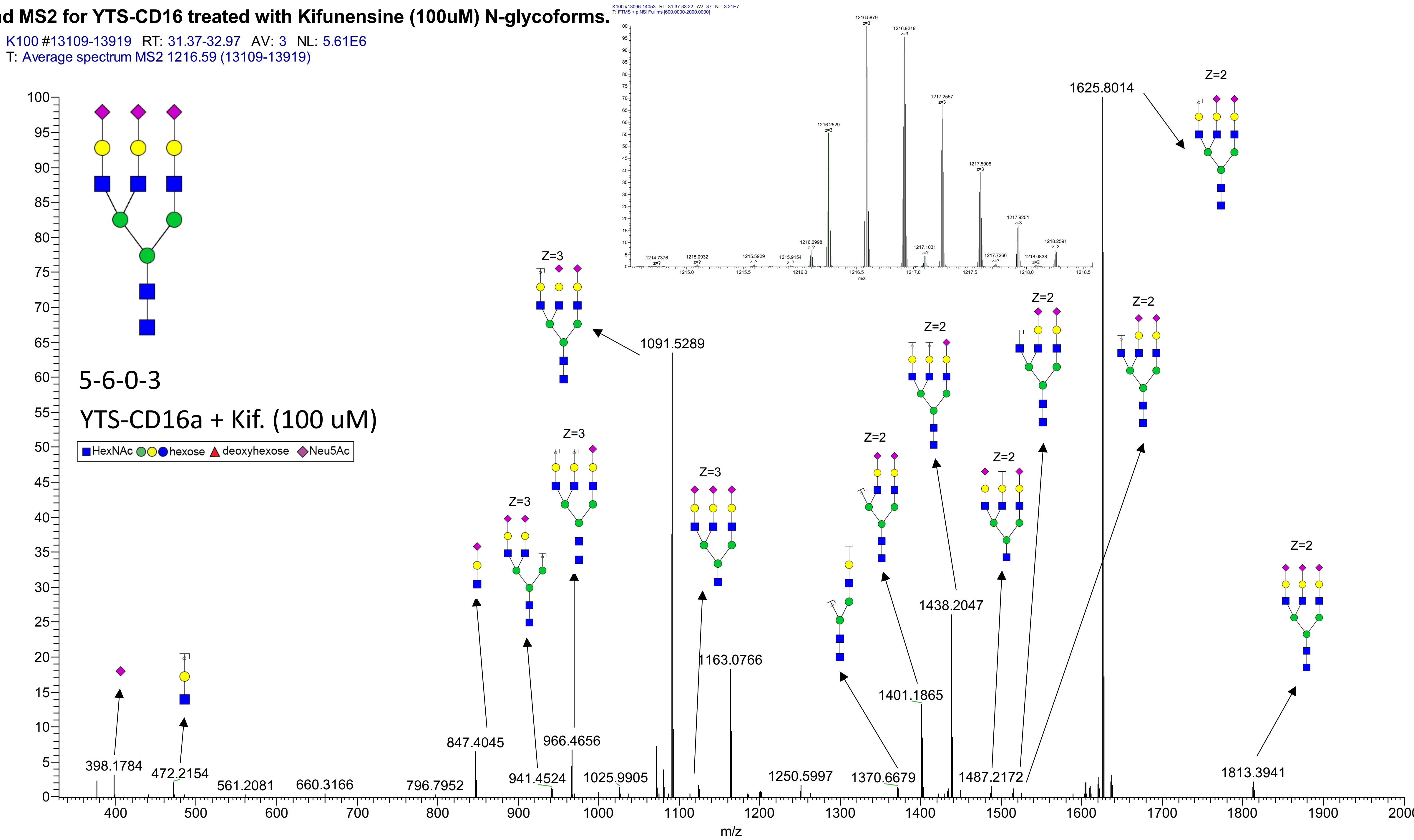

MS1 and MS2 for YTS-CD16 treated with Kifunensine (100uM) N-glycoforms.

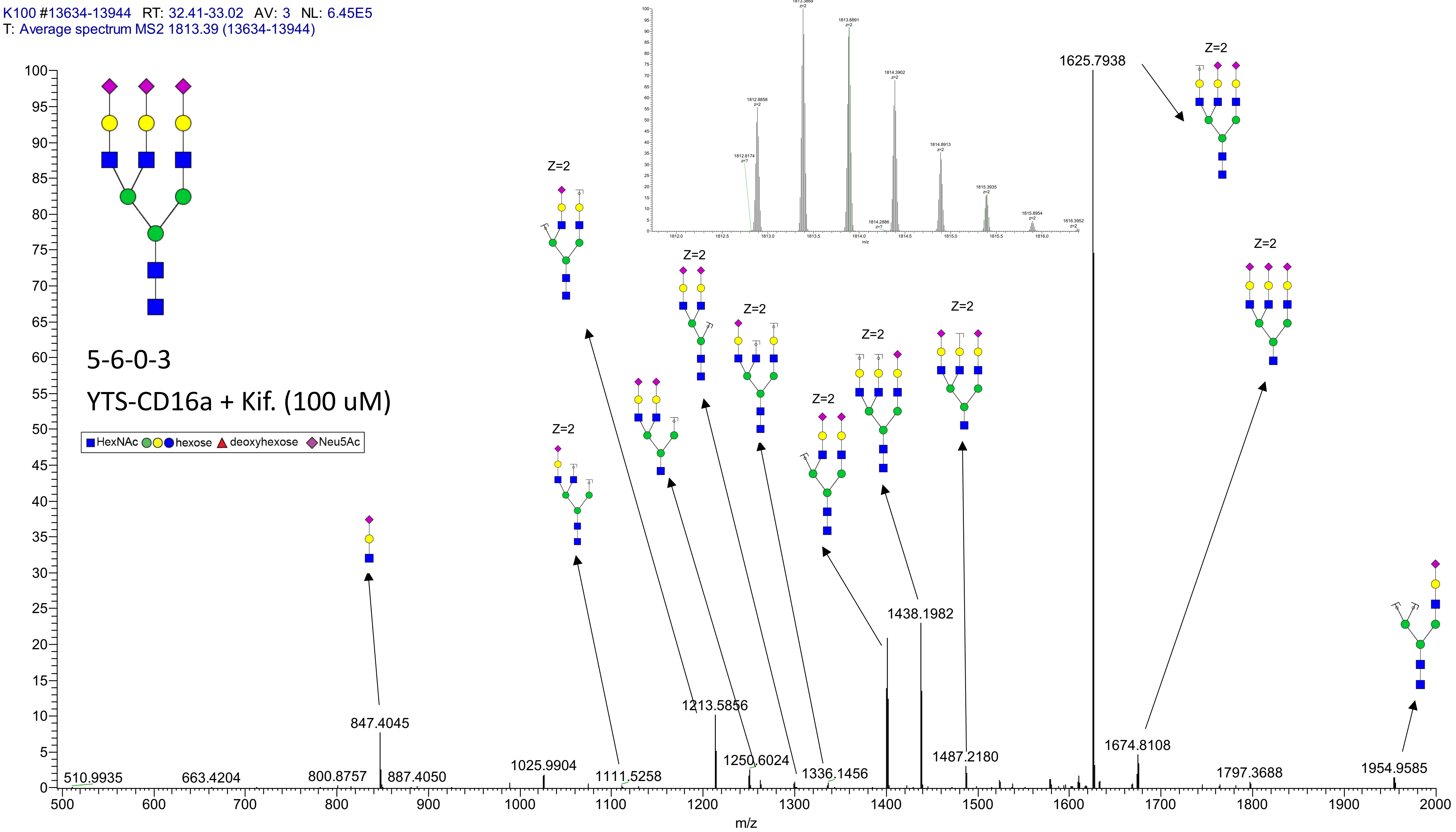

MS1 and MS2 for YTS-CD16 treated with Kifunensine (100uM) N-glycoforms.

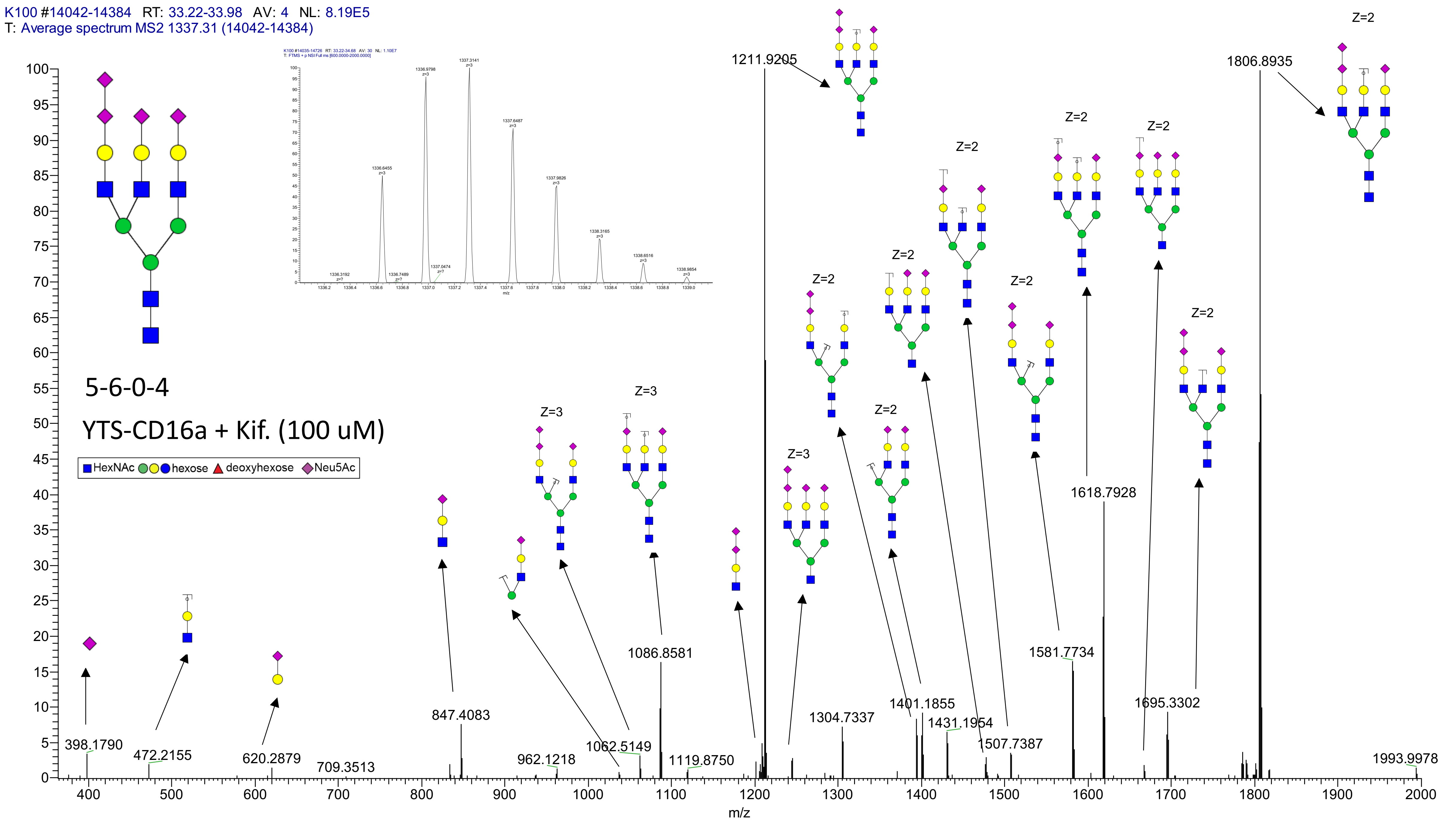

MS1 and MS2 for YTS-CD16 treated with Kifunensine (100uM) N-glycoforms.

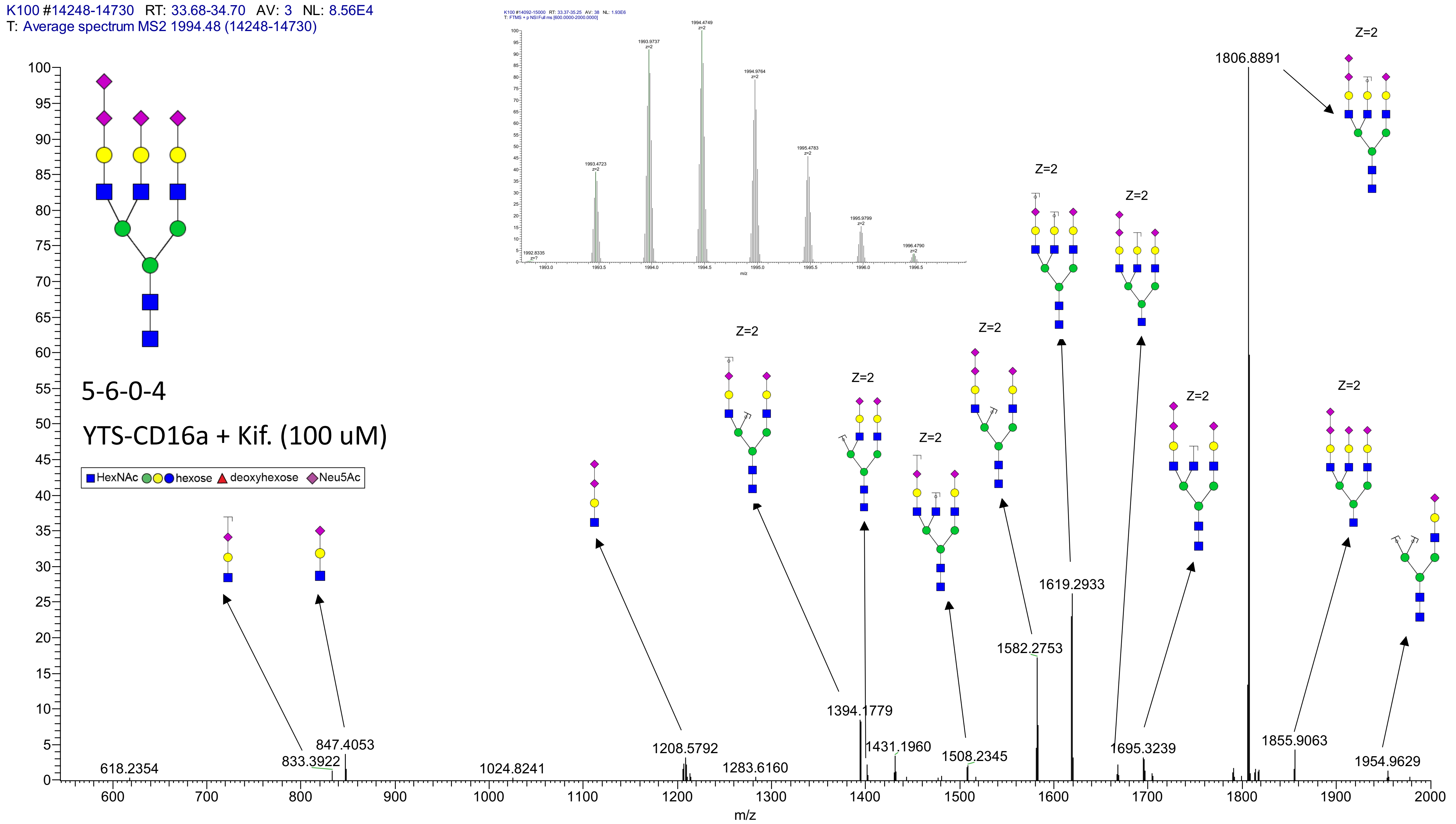

MS1 and MS2 for YTS-CD16 treated with Kifunensine (100uM) N-glycoforms.

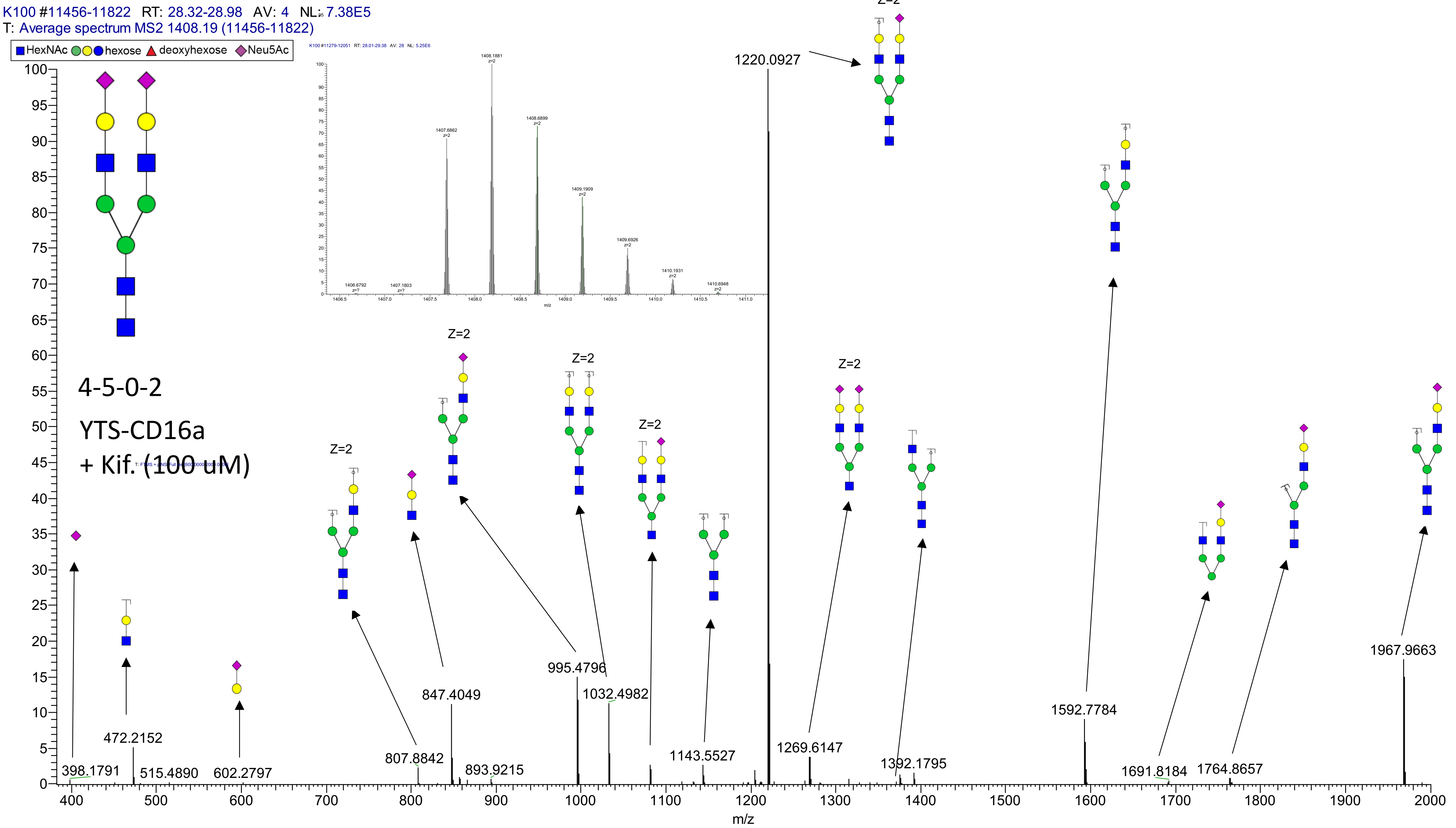

MS1 and MS2 for YTS-CD16 treated with Kifunensine (100uM) N-glycoforms.

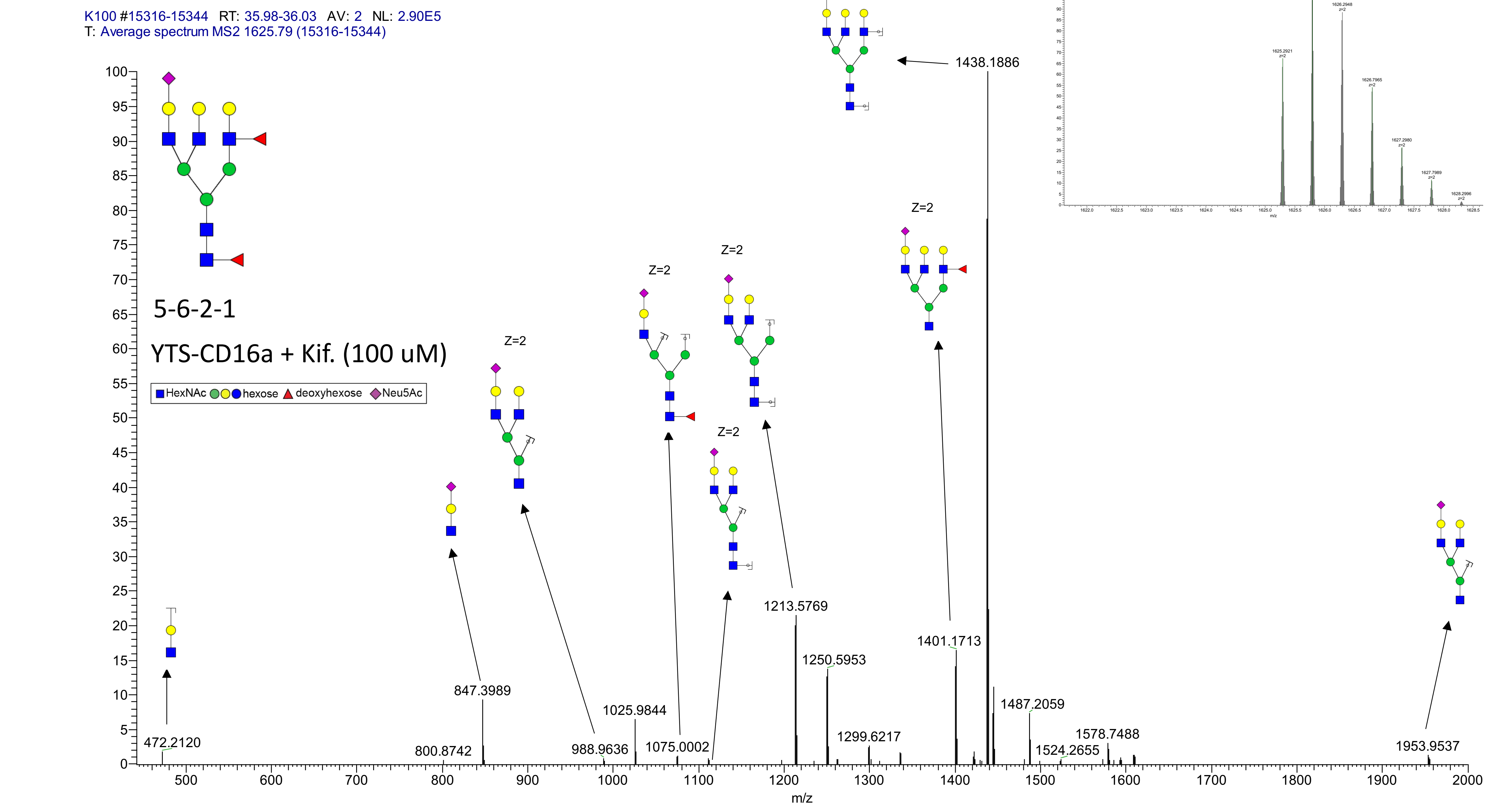

MS1 and MS2 for YTS-CD16 treated with Kifunensine (100uM) N-glycoforms.

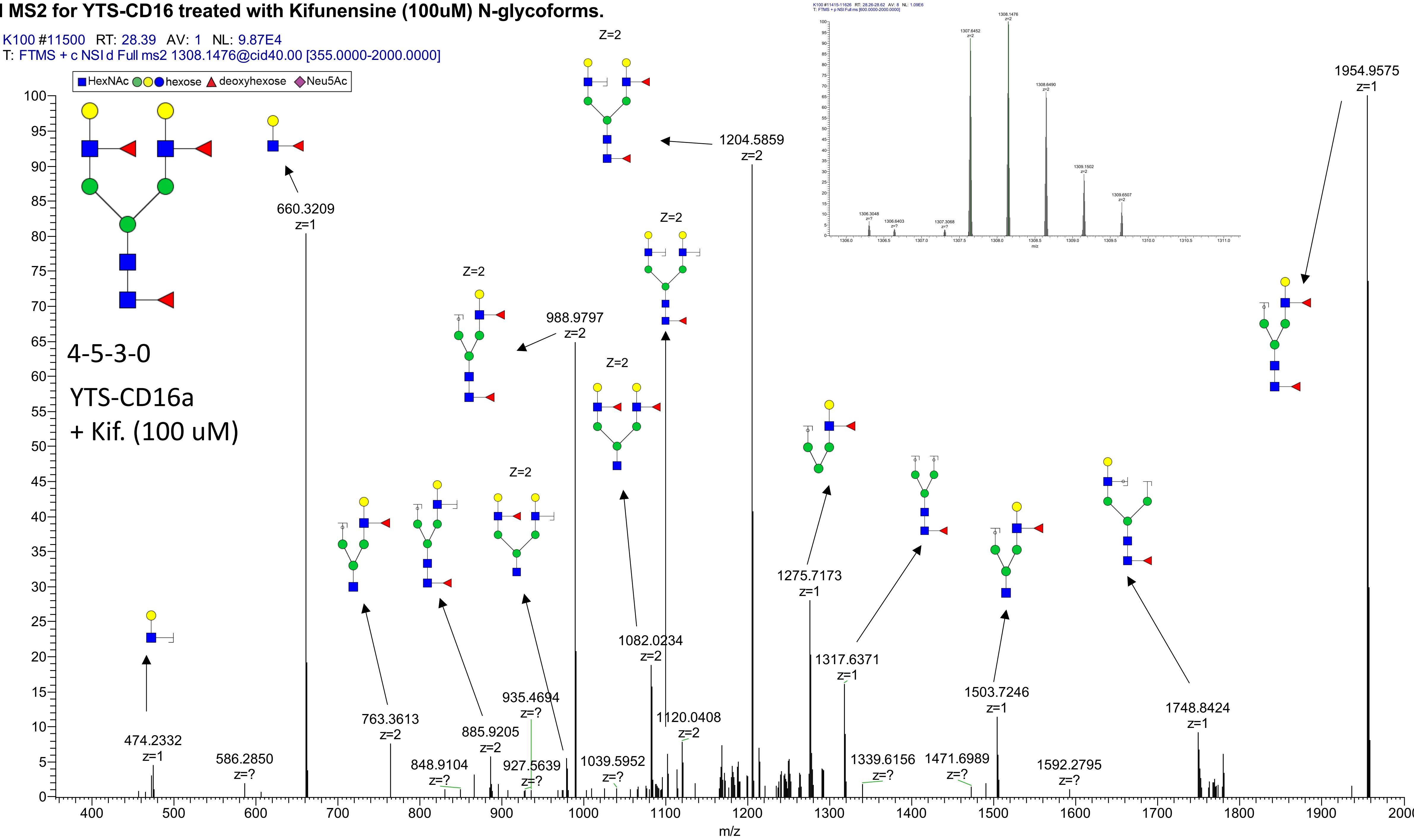

MS1 and MS2 for E1 clone N-glycoforms.

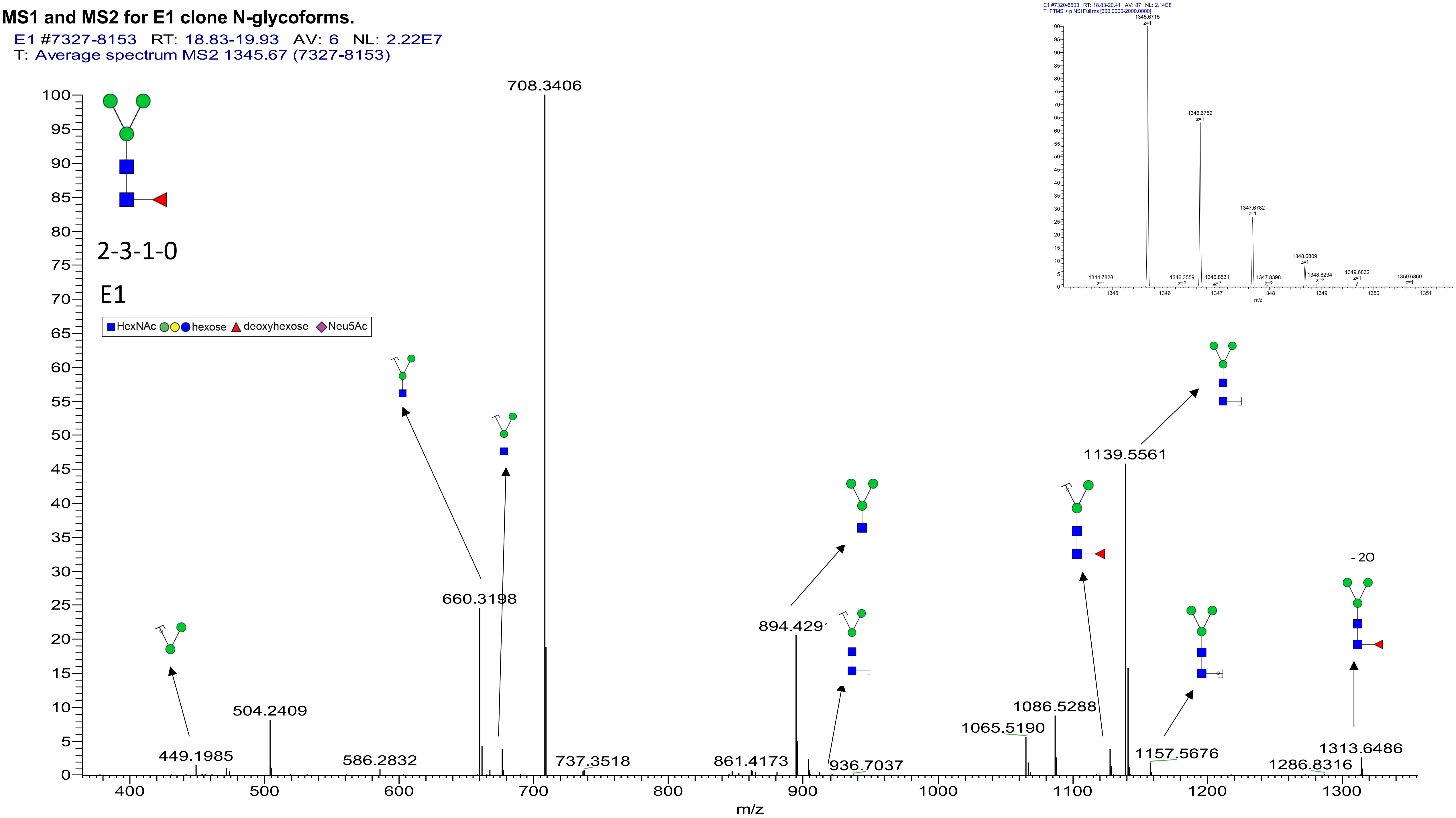

MS1 and MS2 for E1 clone N-glycoforms.

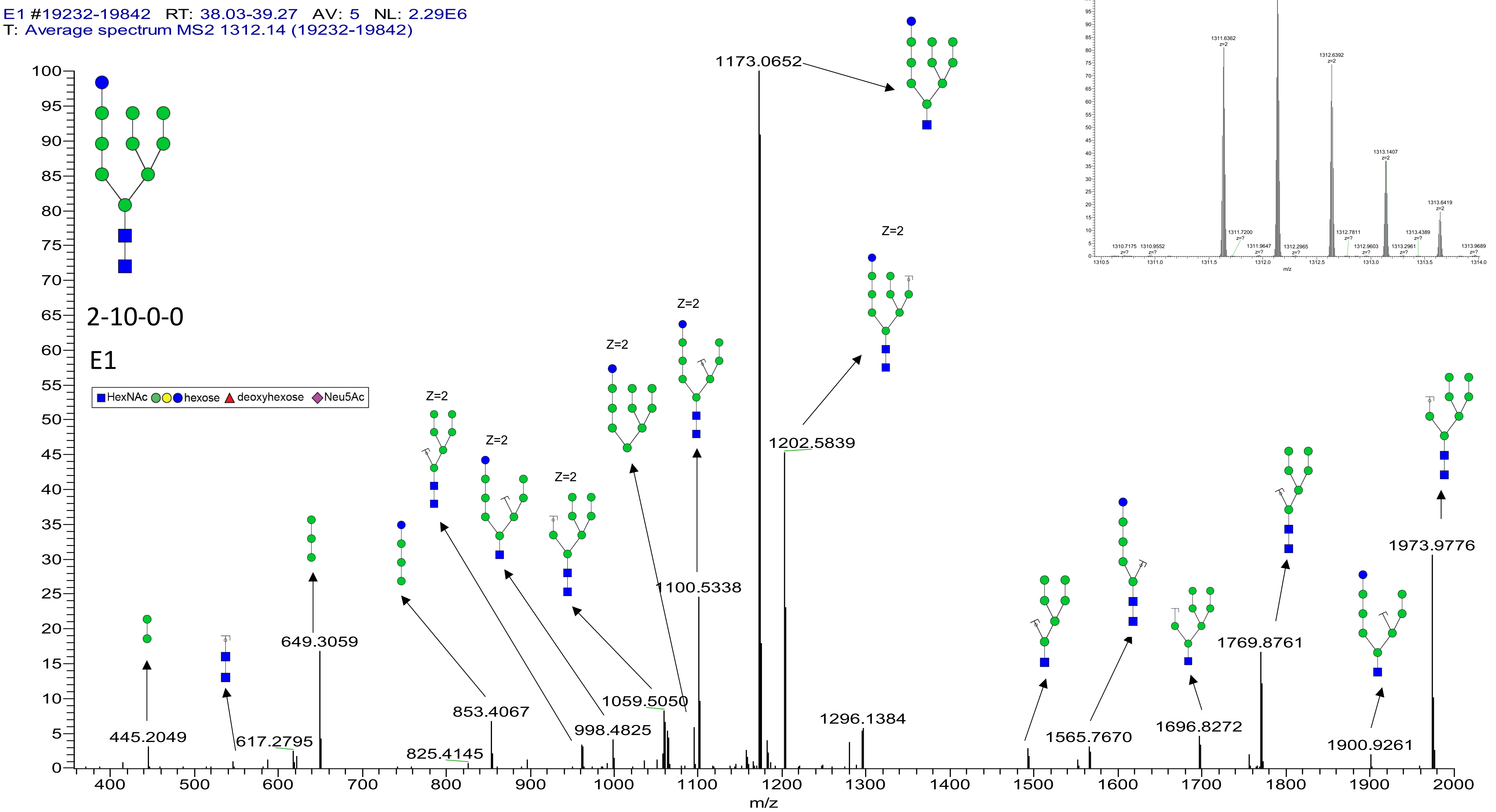

MS1 and MS2 for E1 clone N-glycoforms.

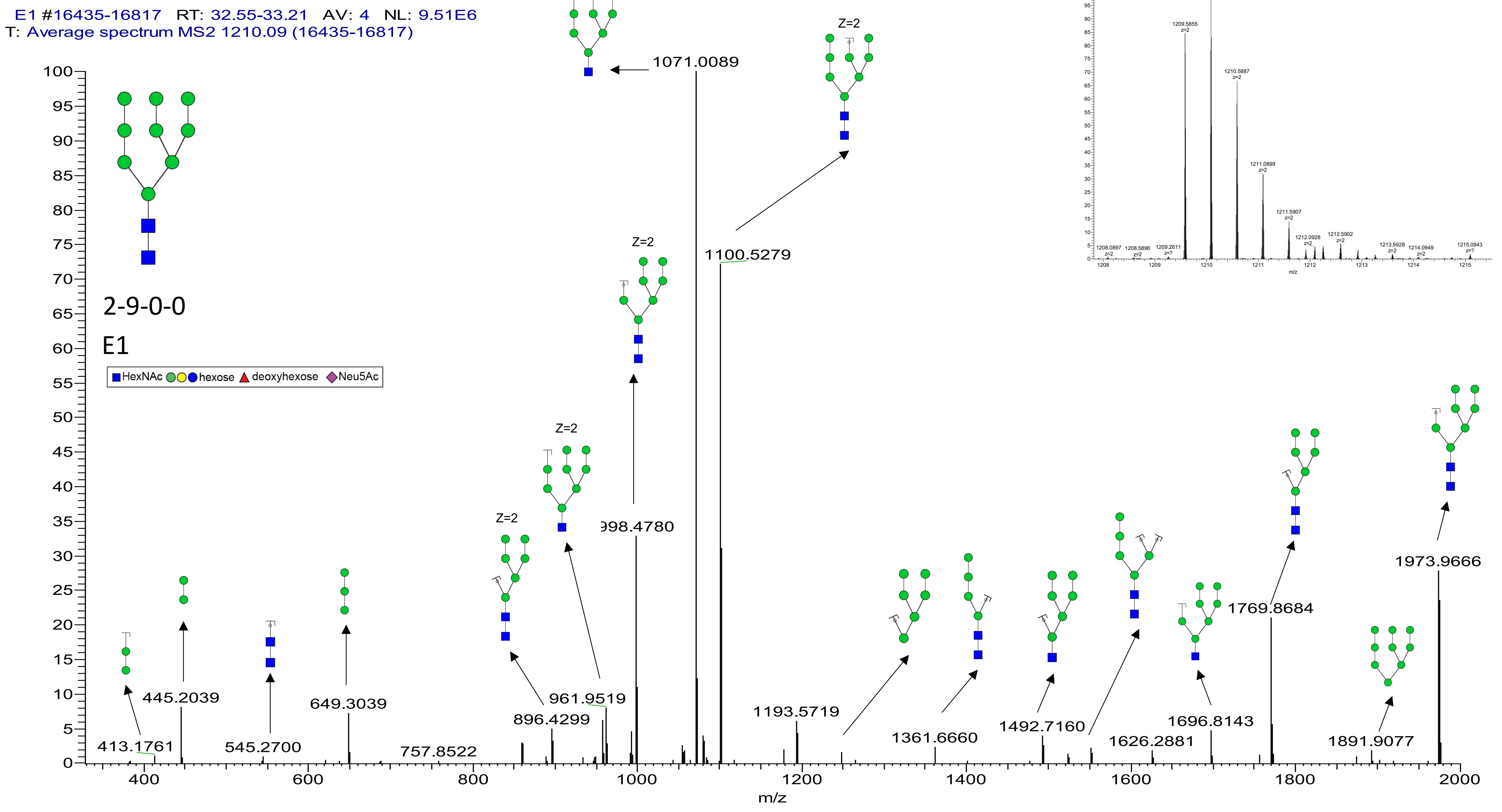

MS1 and MS2 for E1 clone N-glycoforms.

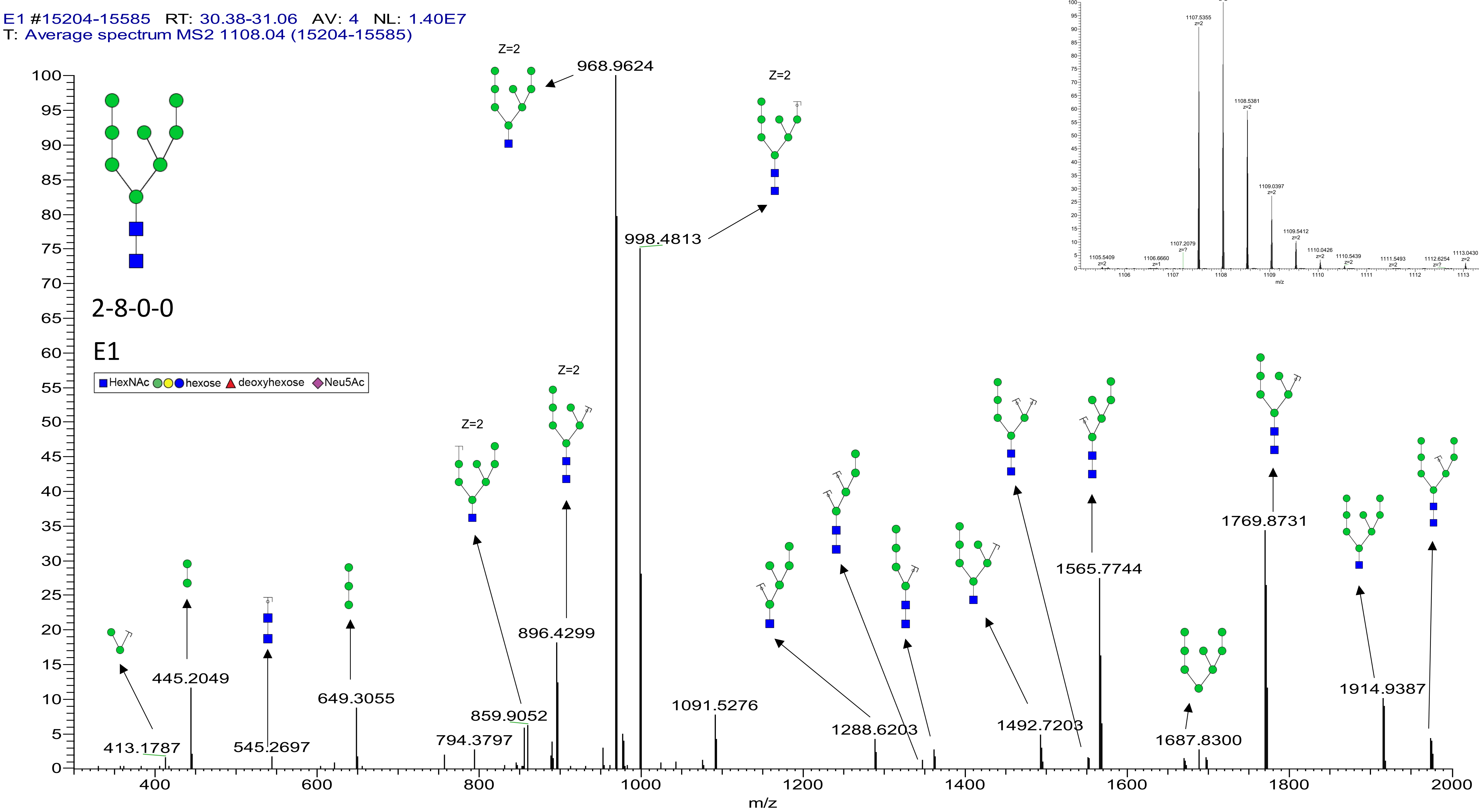

MS1 and MS2 for E1 clone N-glycoforms.

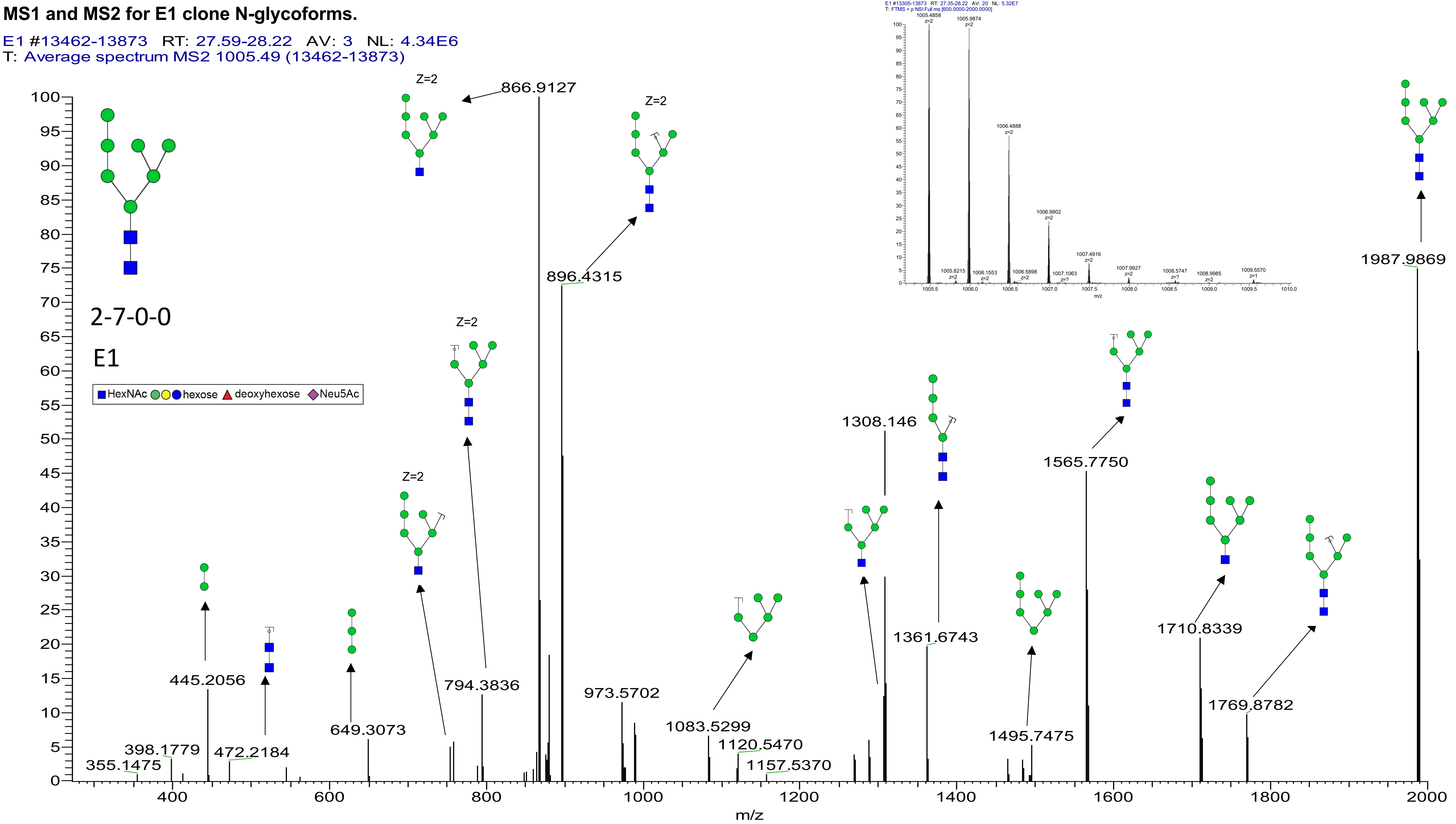

MS1 and MS2 for E1 clone N-glycoforms.

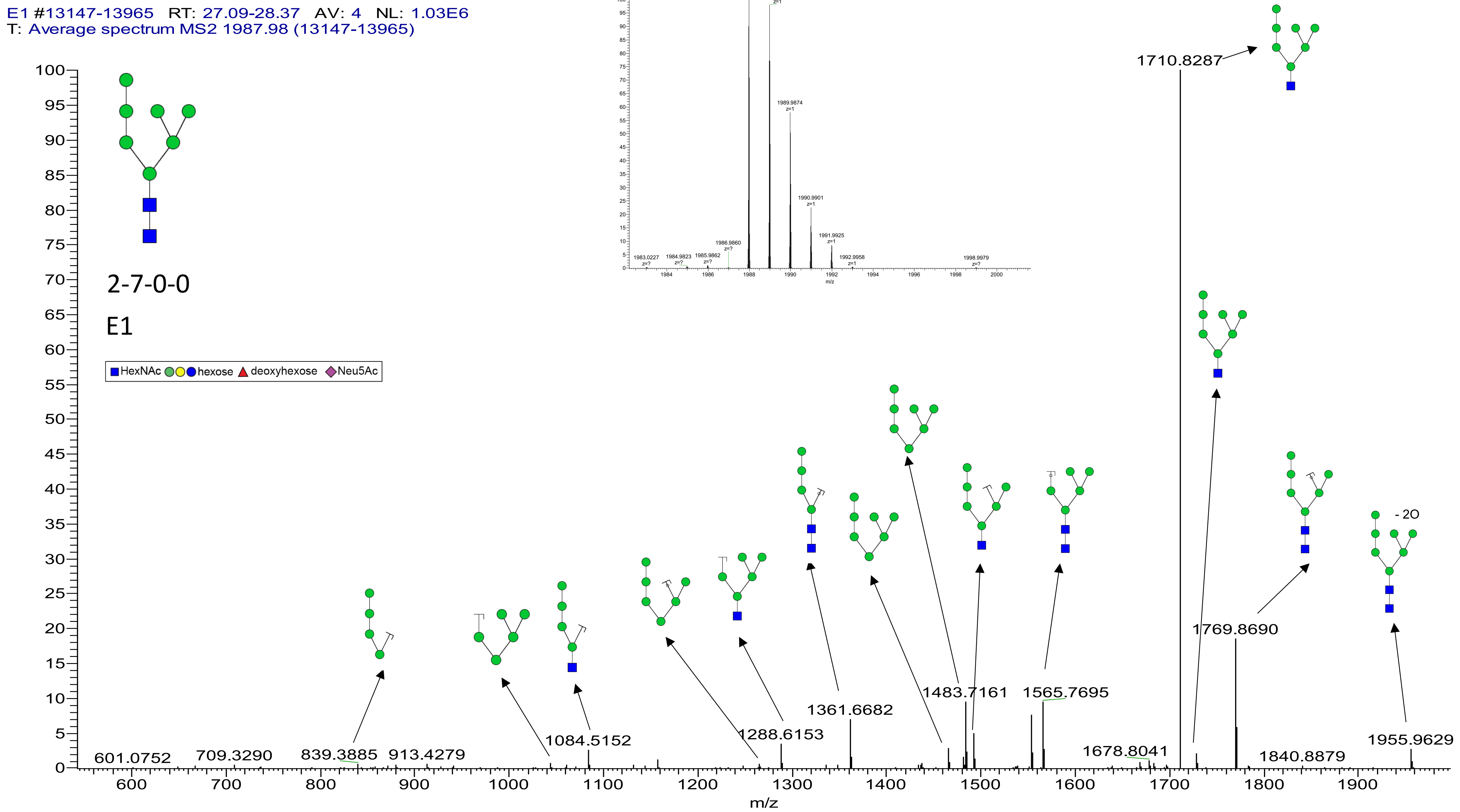

MS1 and MS2 for E1 clone N-glycoforms.

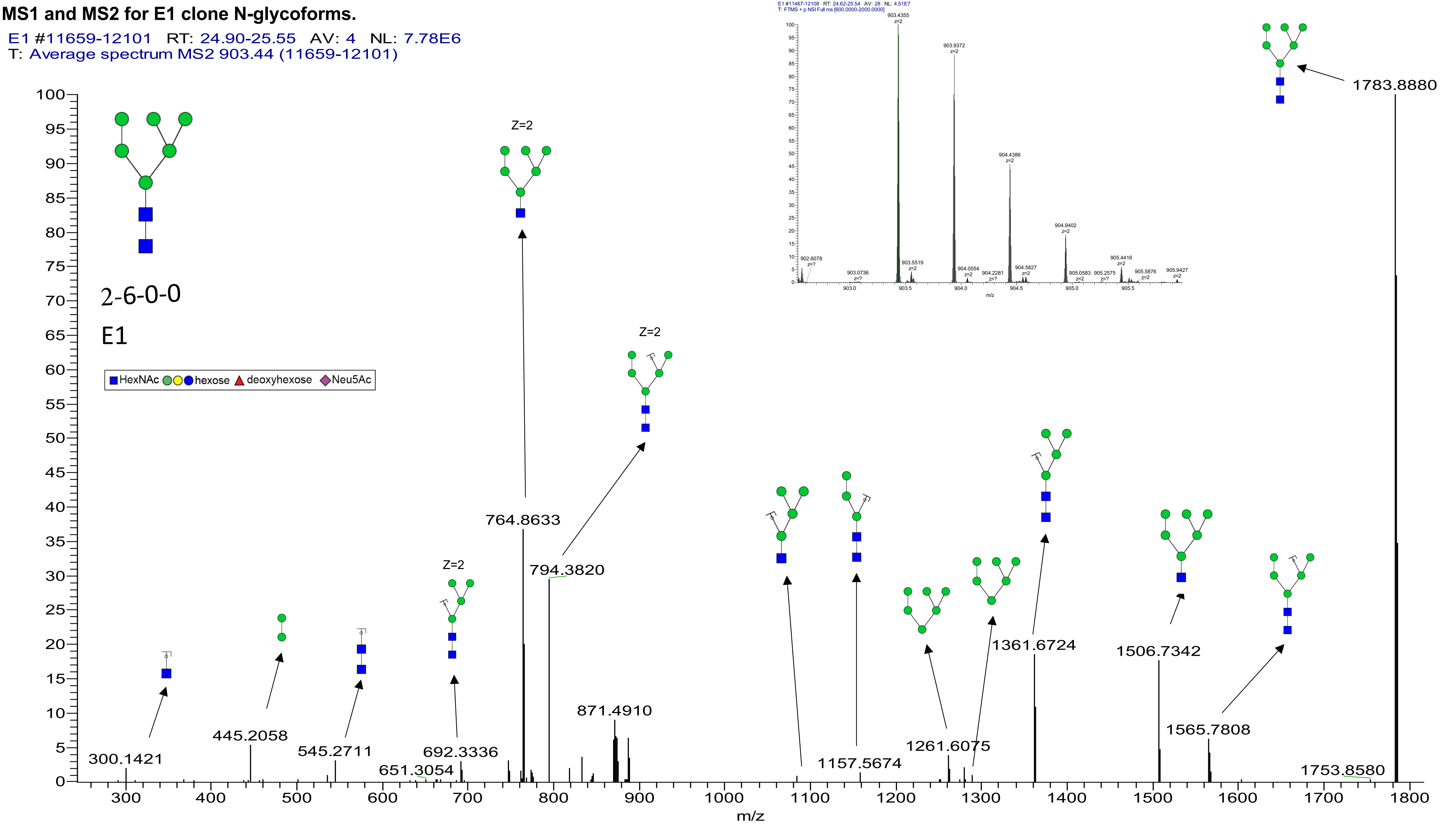

MS1 and MS2 for E1 clone N-glycoforms.

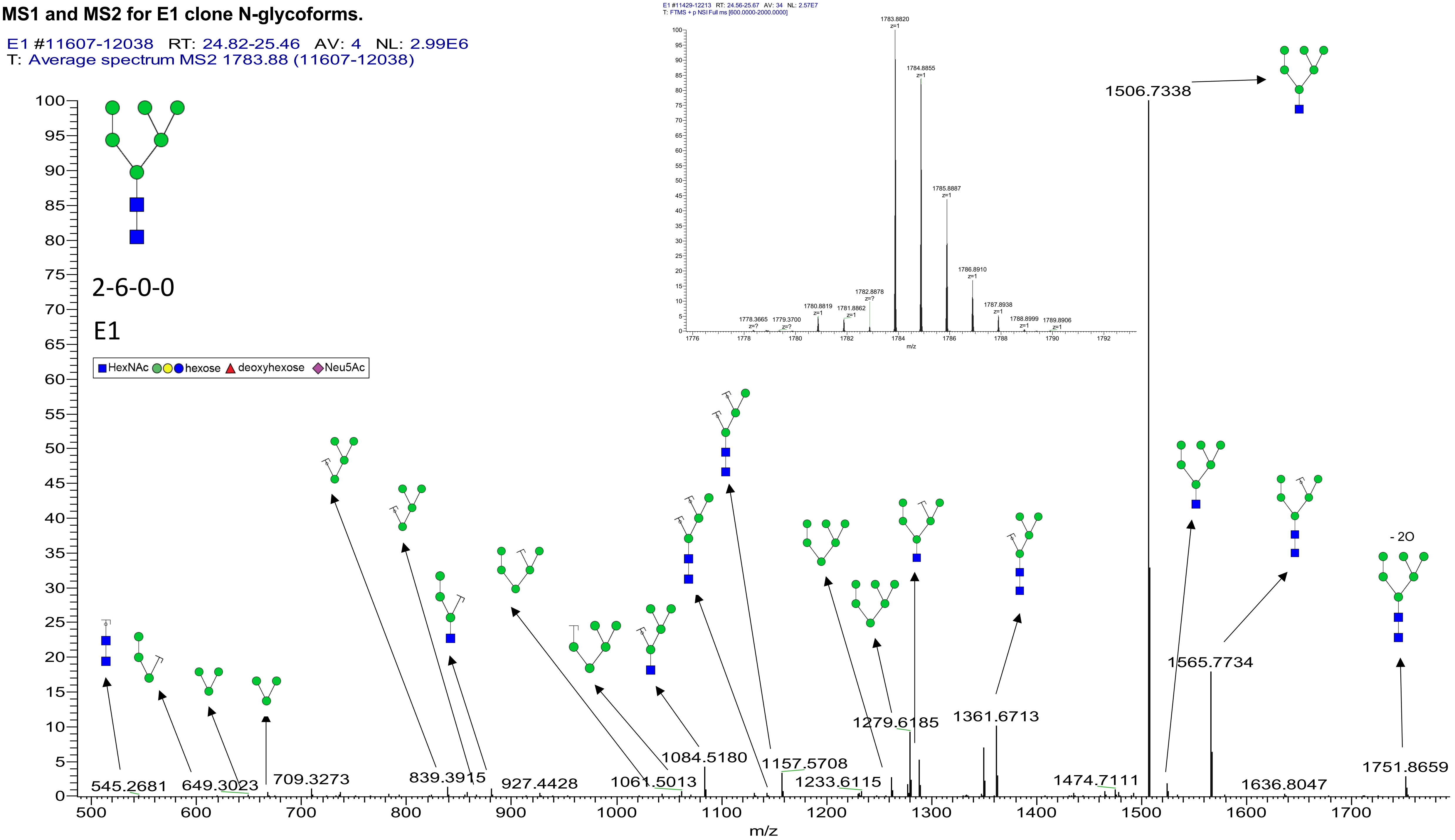

MS1 and MS2 for E1 clone N-glycoforms.

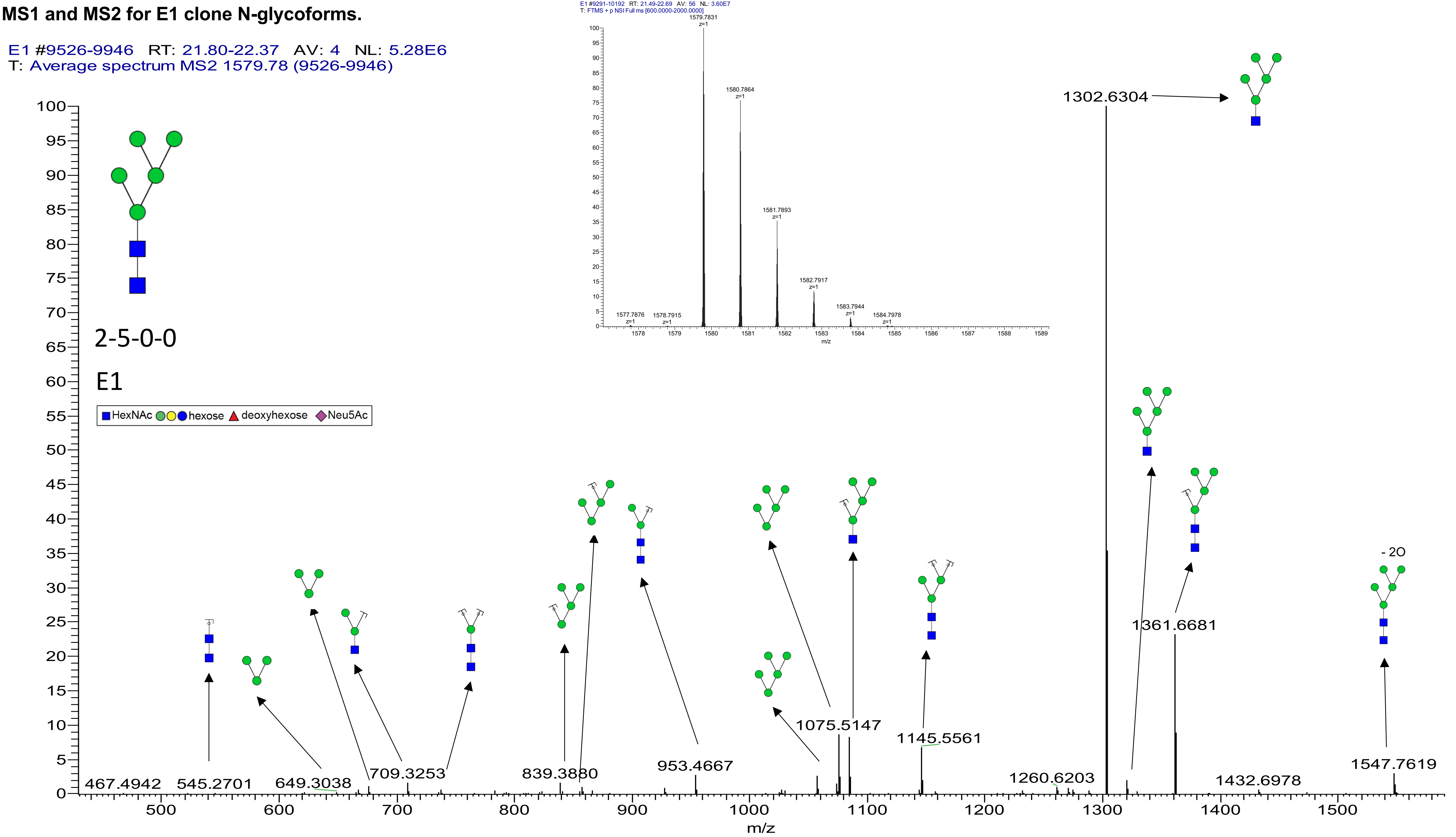

MS1 and MS2 for E1 clone N-glycoforms.

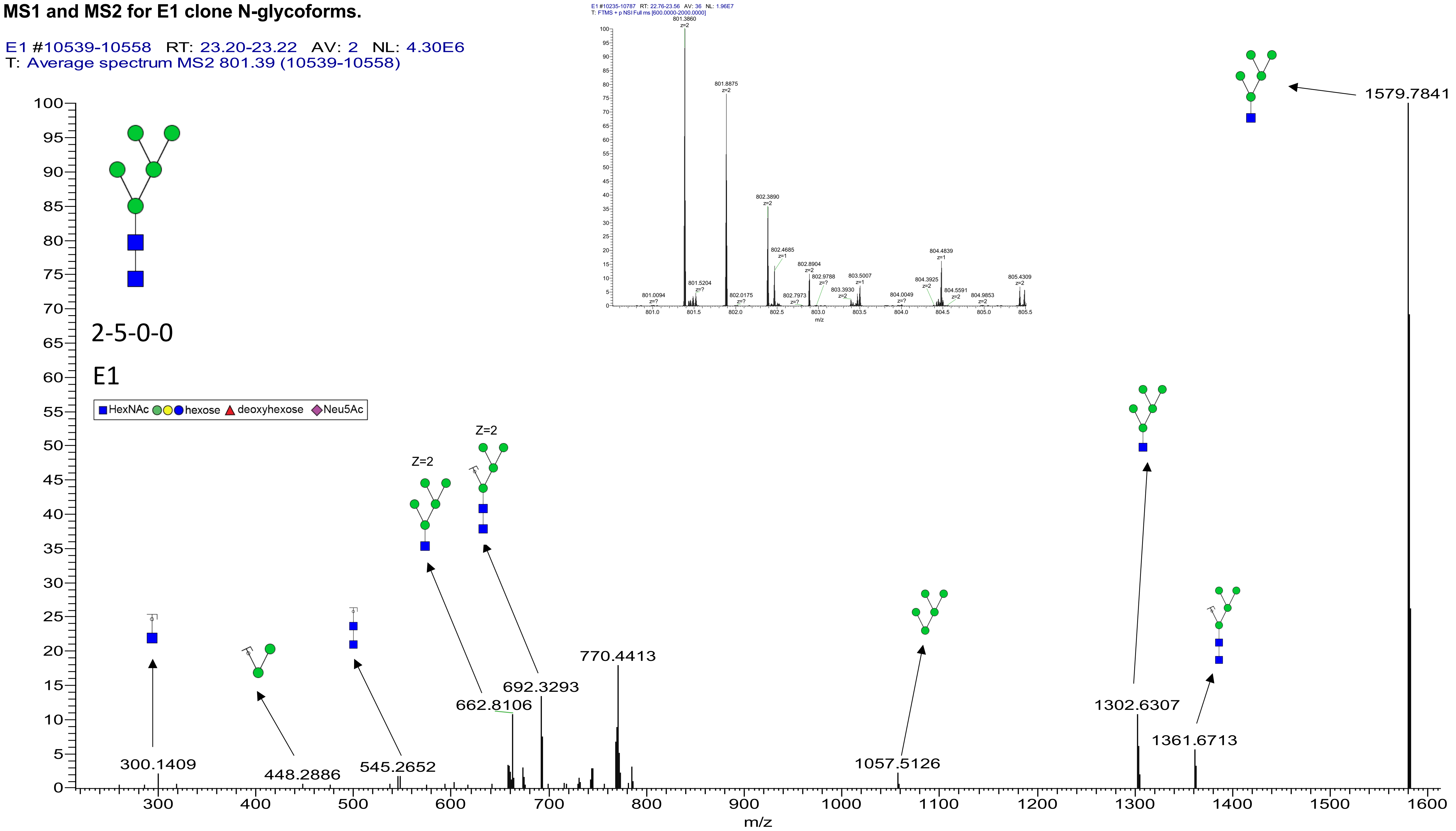

MS1 and MS2 for E1 clone N-glycoforms.

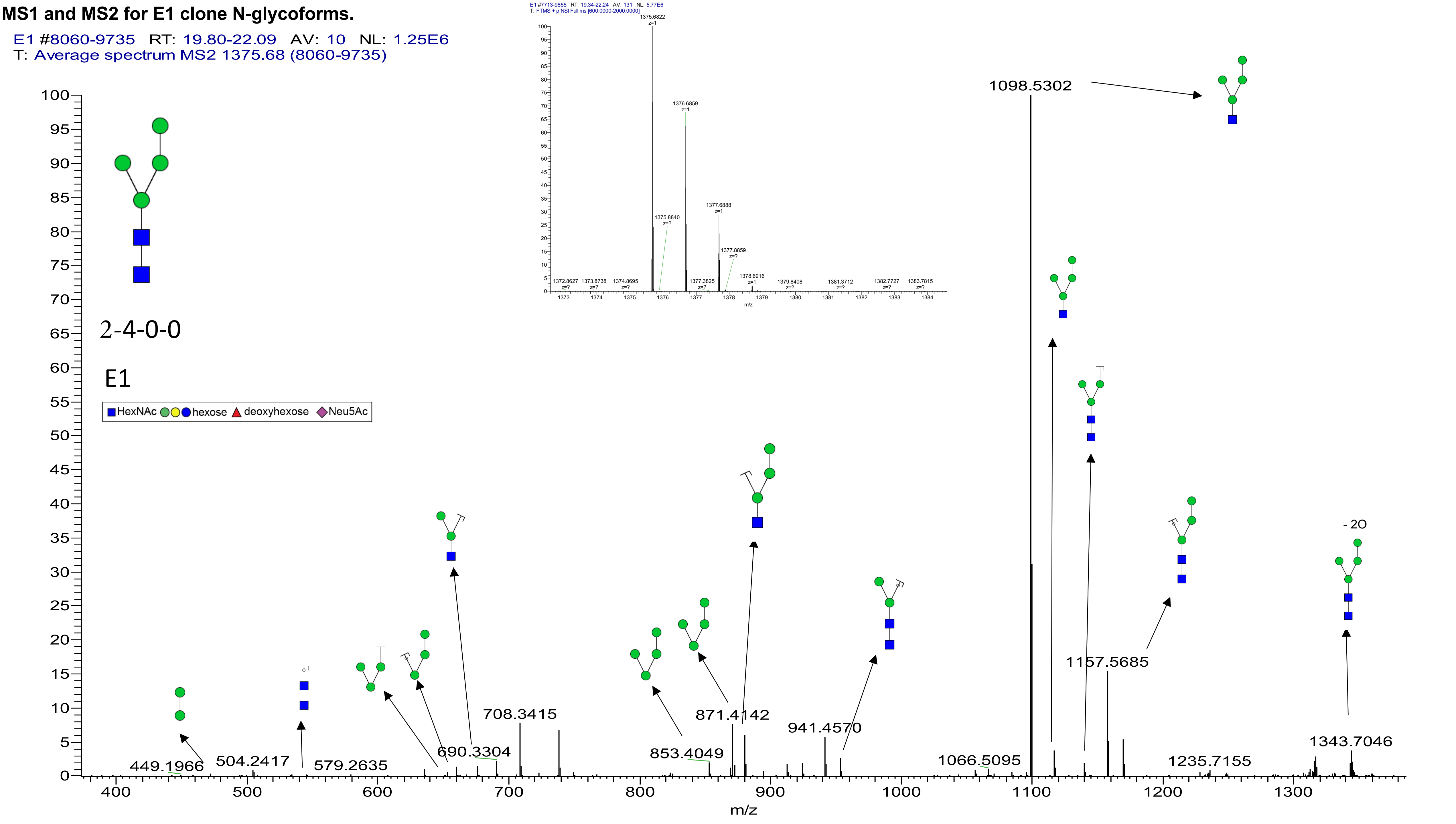

MS1 and MS2 for E1 clone N-glycoforms.

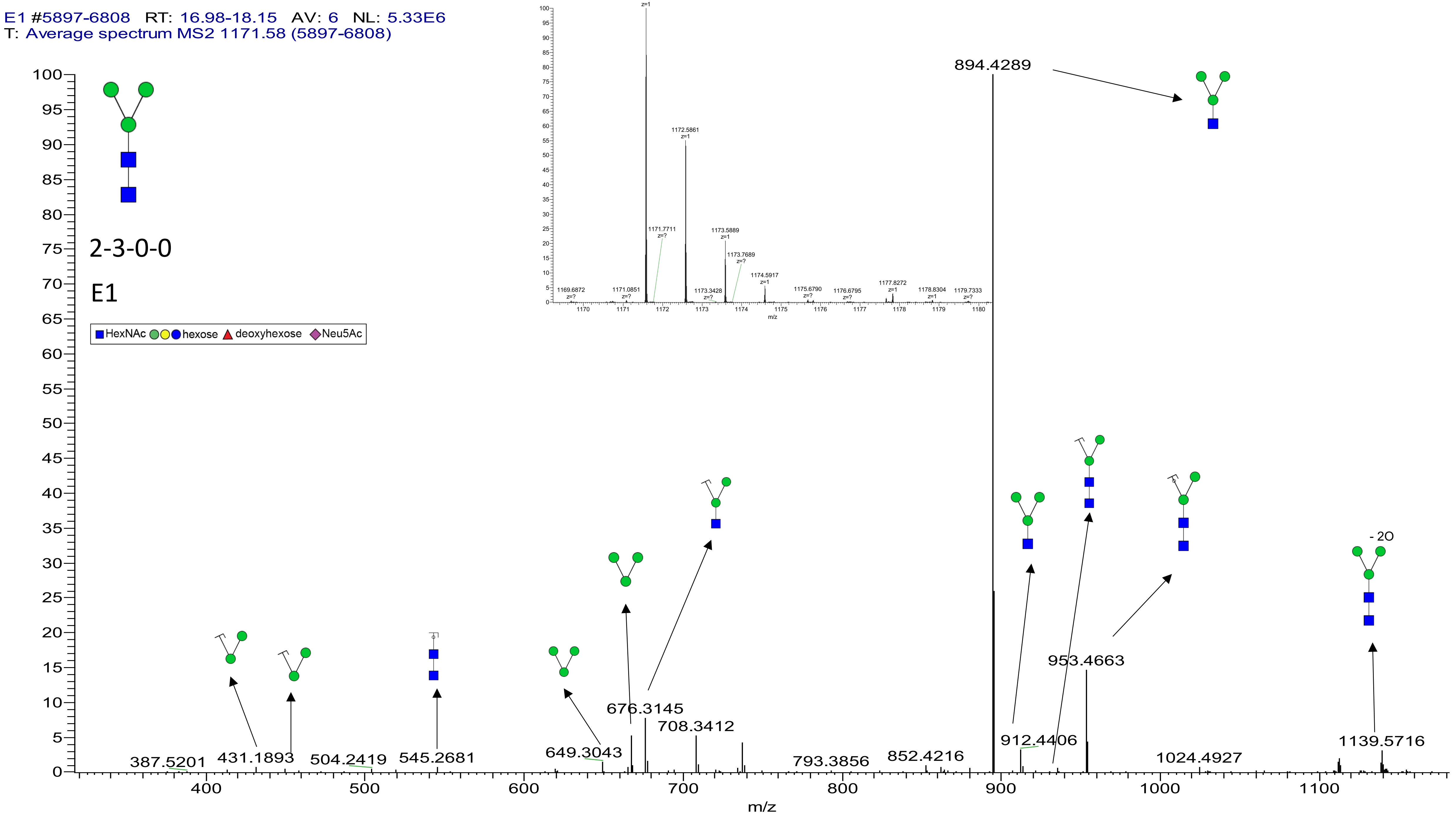

MS1 and MS2 for E1 clone N-glycoforms.

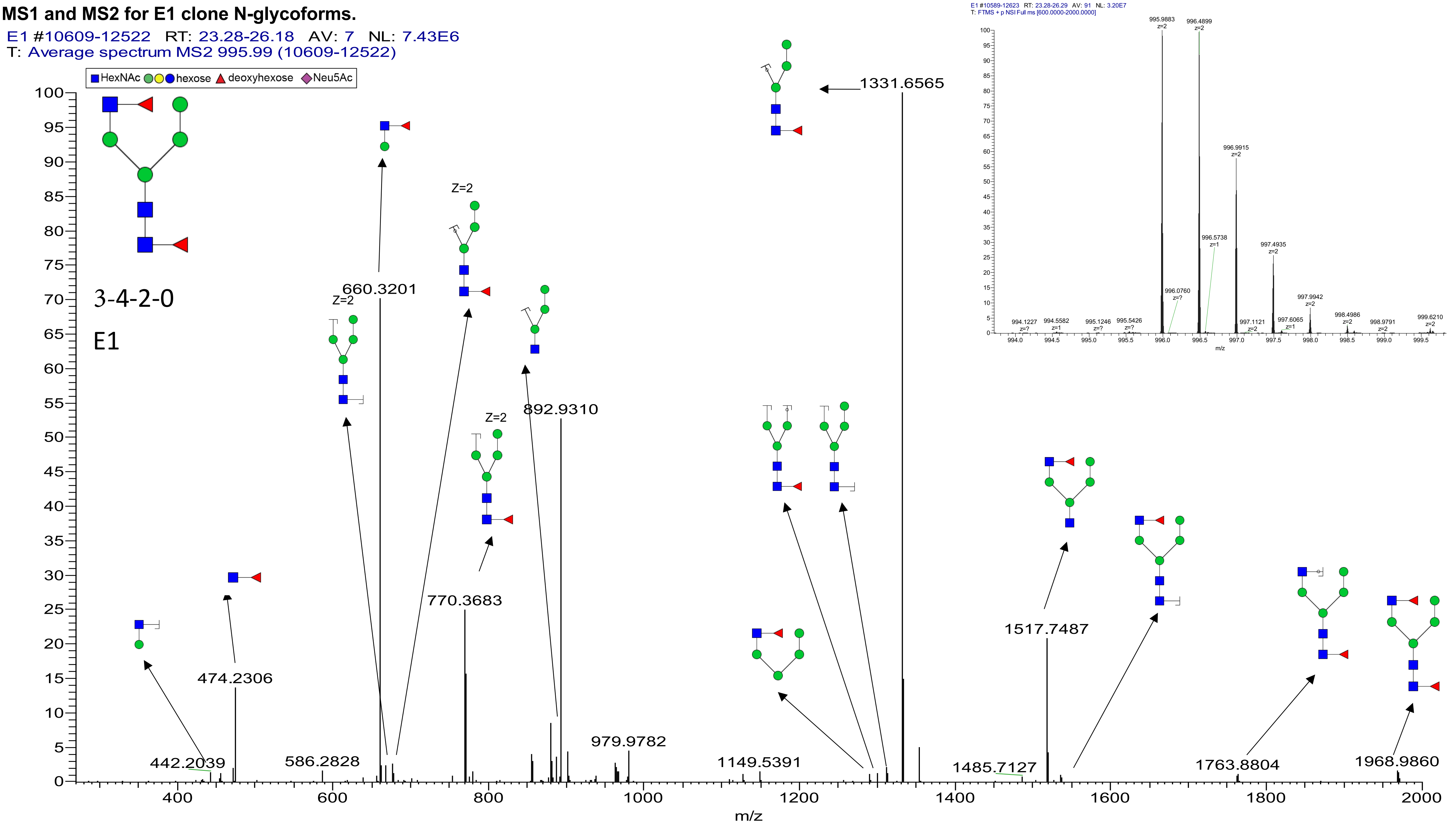

MS1 and MS2 for E1 clone N-glycoforms.

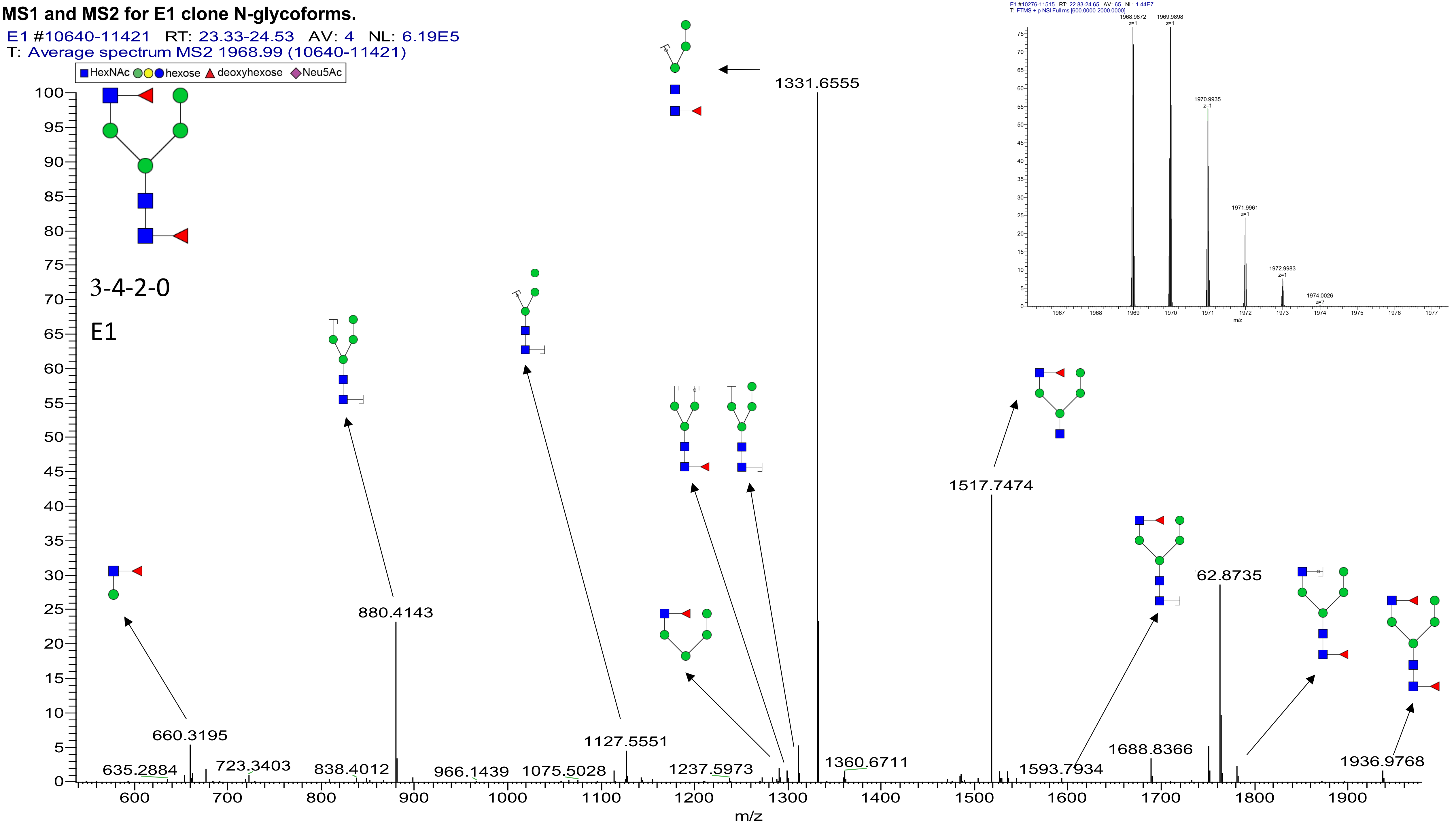

MS1 and MS2 for E1 clone N-glycoforms.

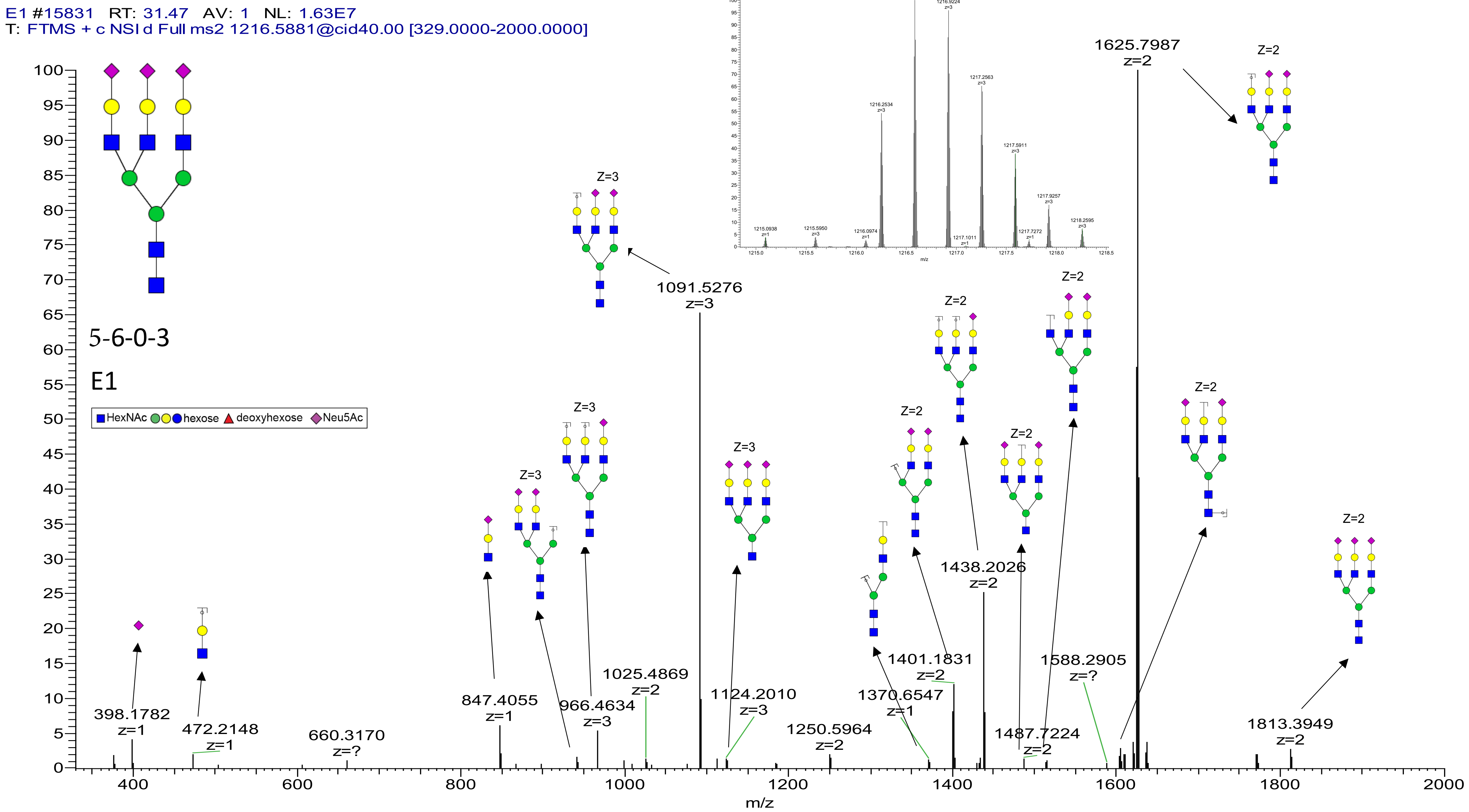

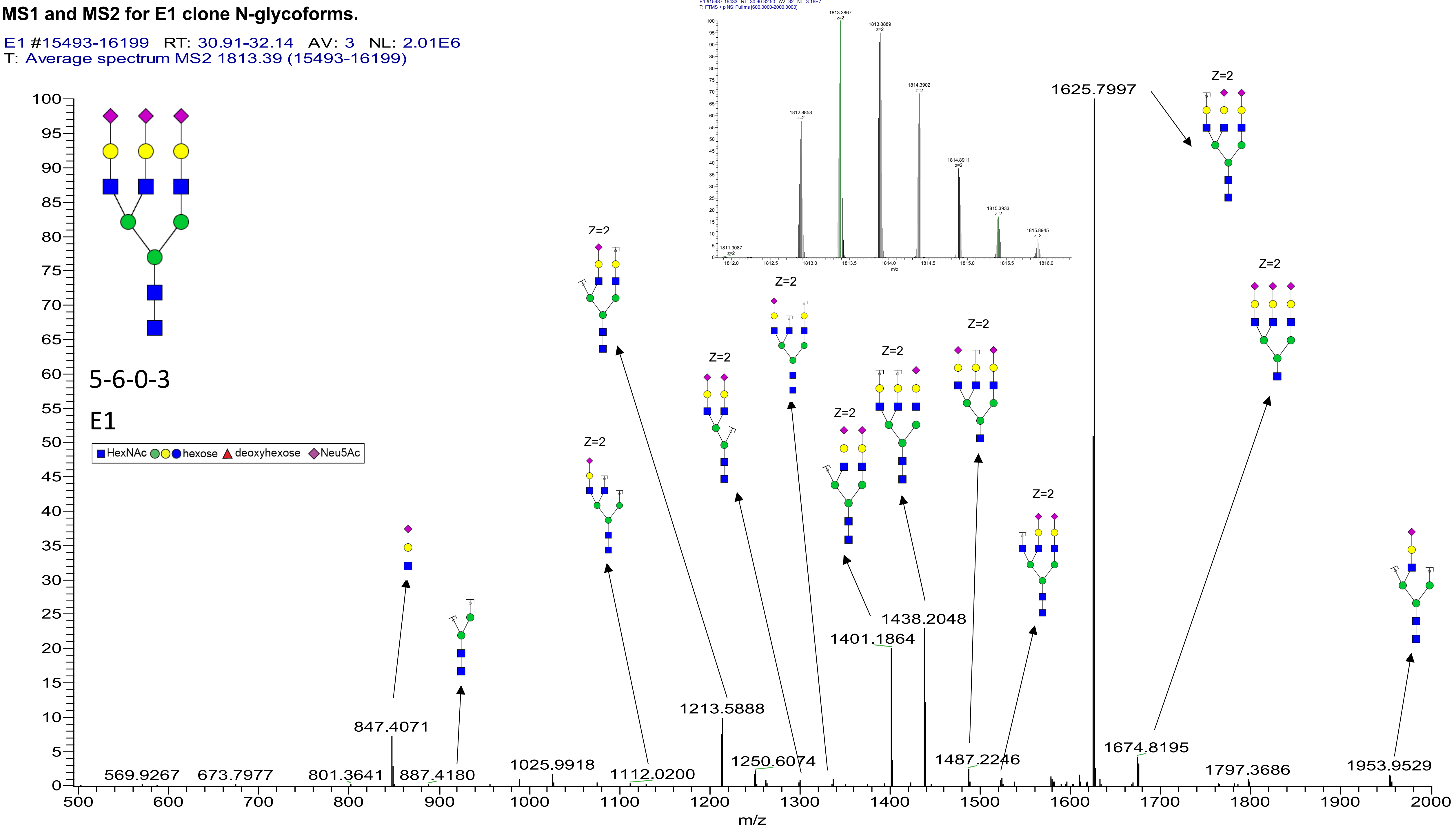

MS1 and MS2 for E1 clone N-glycoforms.

MS1 and MS2 for E1 clone N-glycoforms.

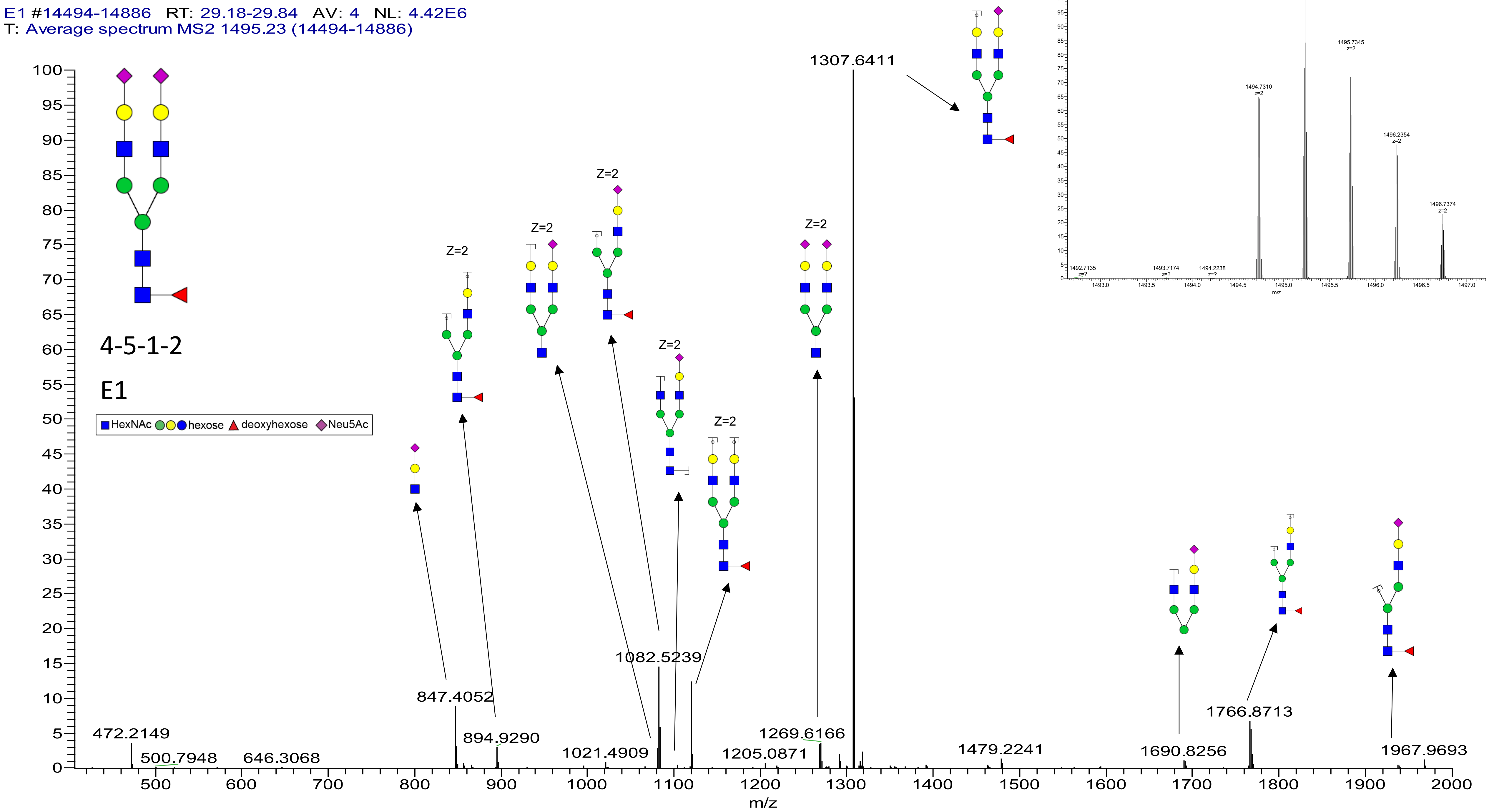

MS1 for E1 clone N-glycoforms.

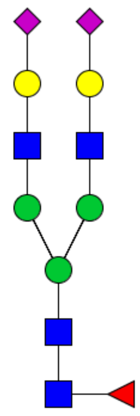

4-5-1-2

E1

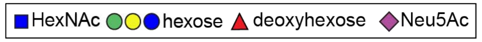

NO MS2 AVAILABLE

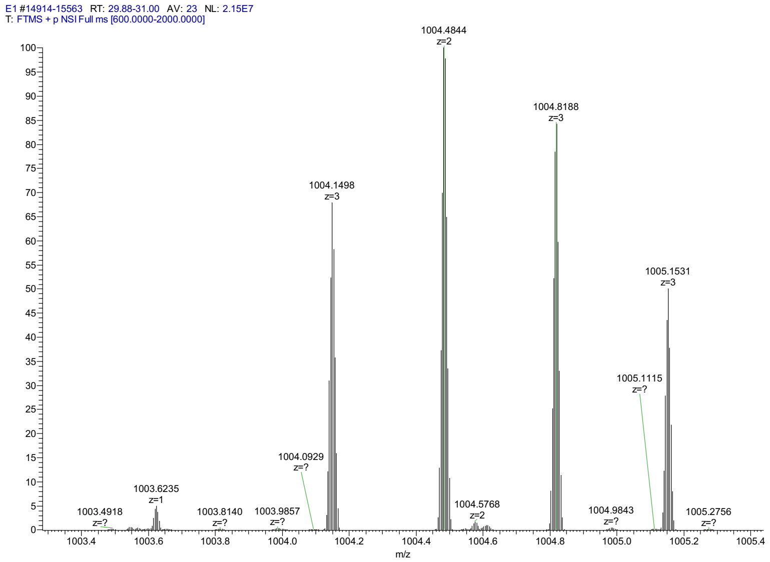

MS1 and MS2 for E1 clone N-glycoforms.

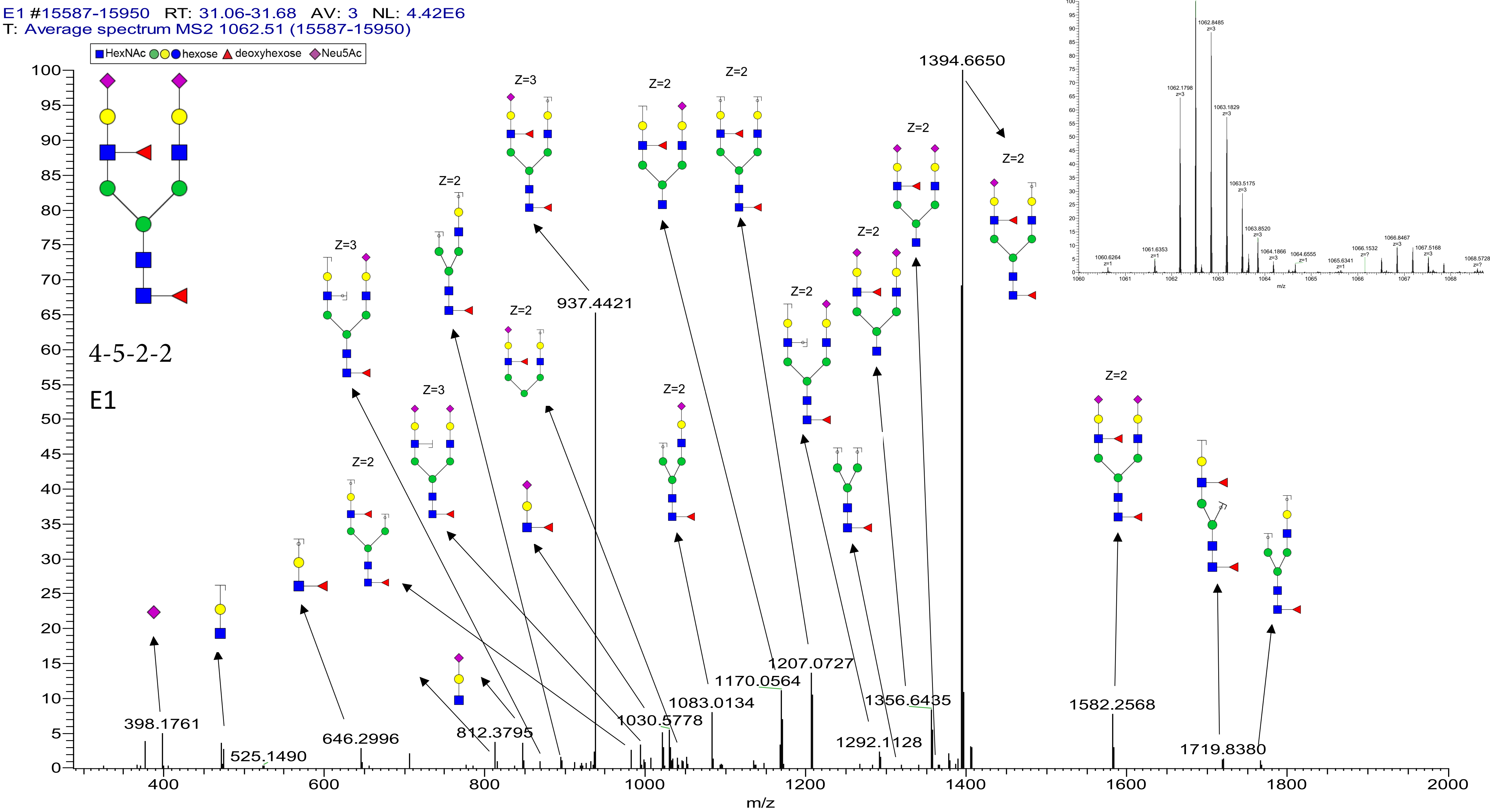

MS1 and MS2 for E1 clone N-glycoforms.

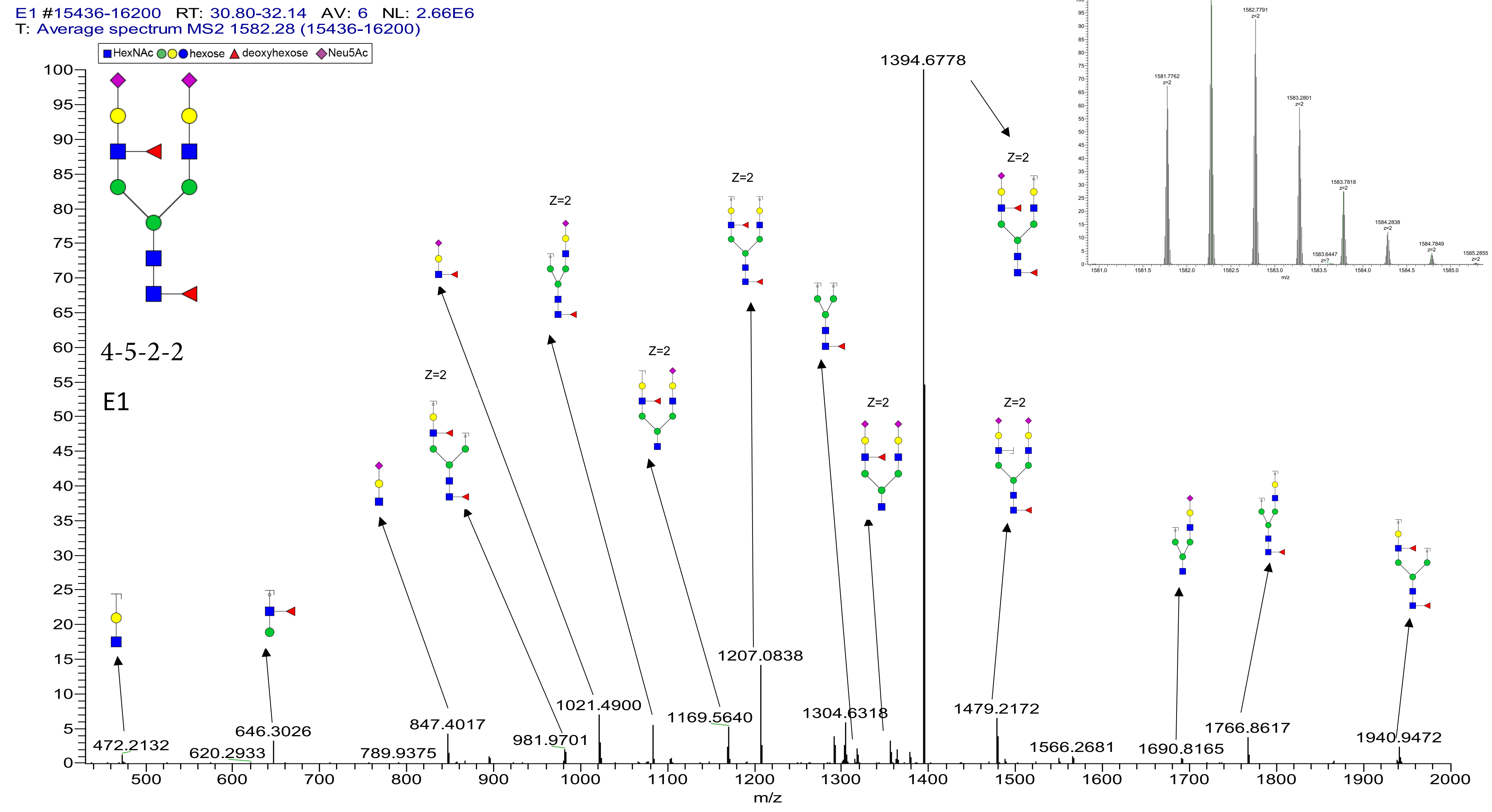

MS1 and MS2 for E1 clone N-glycoforms.

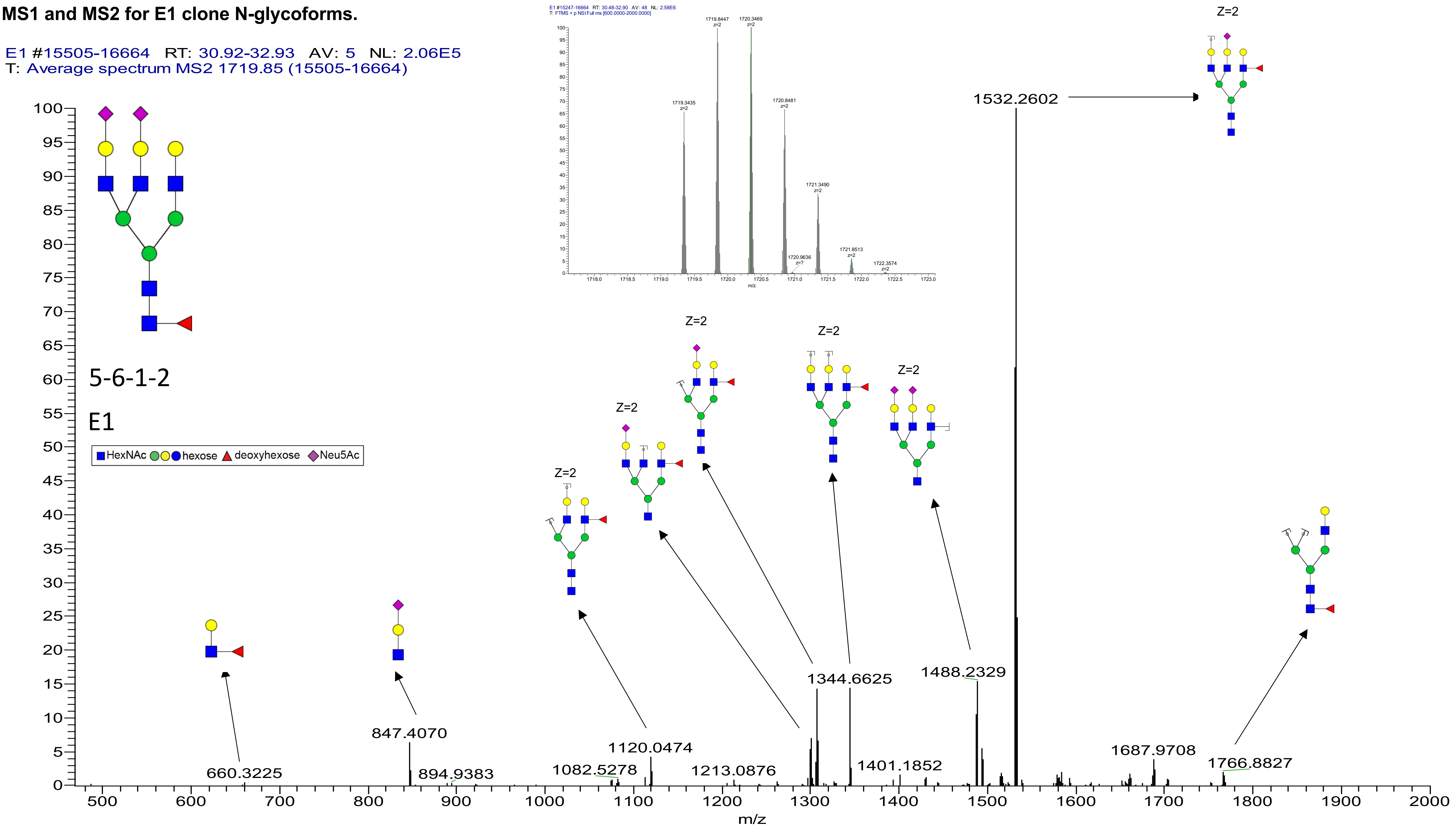

MS1 and MS2 for E1 clone N-glycoforms.

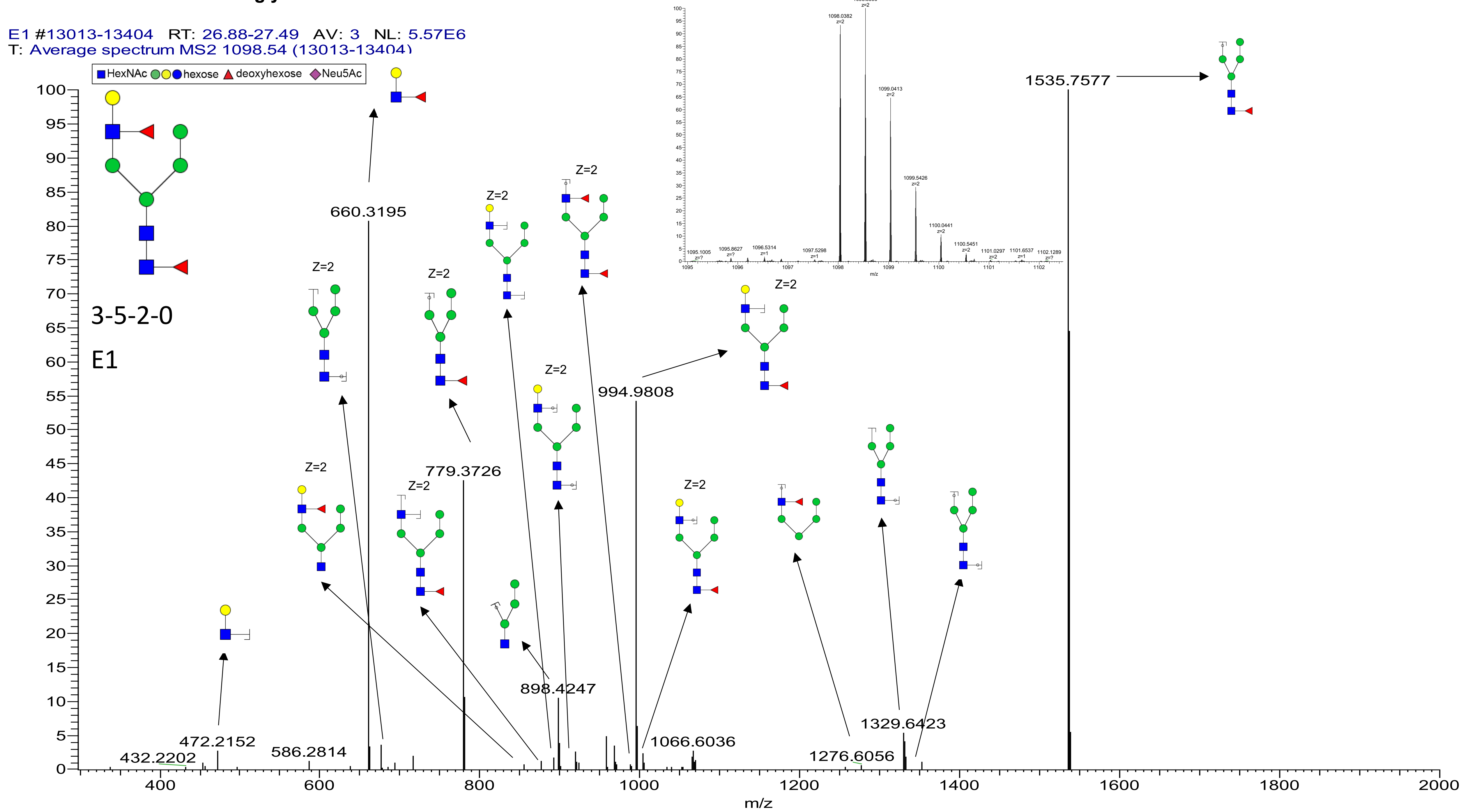

MS1 and MS2 for E1 clone N-glycoforms.

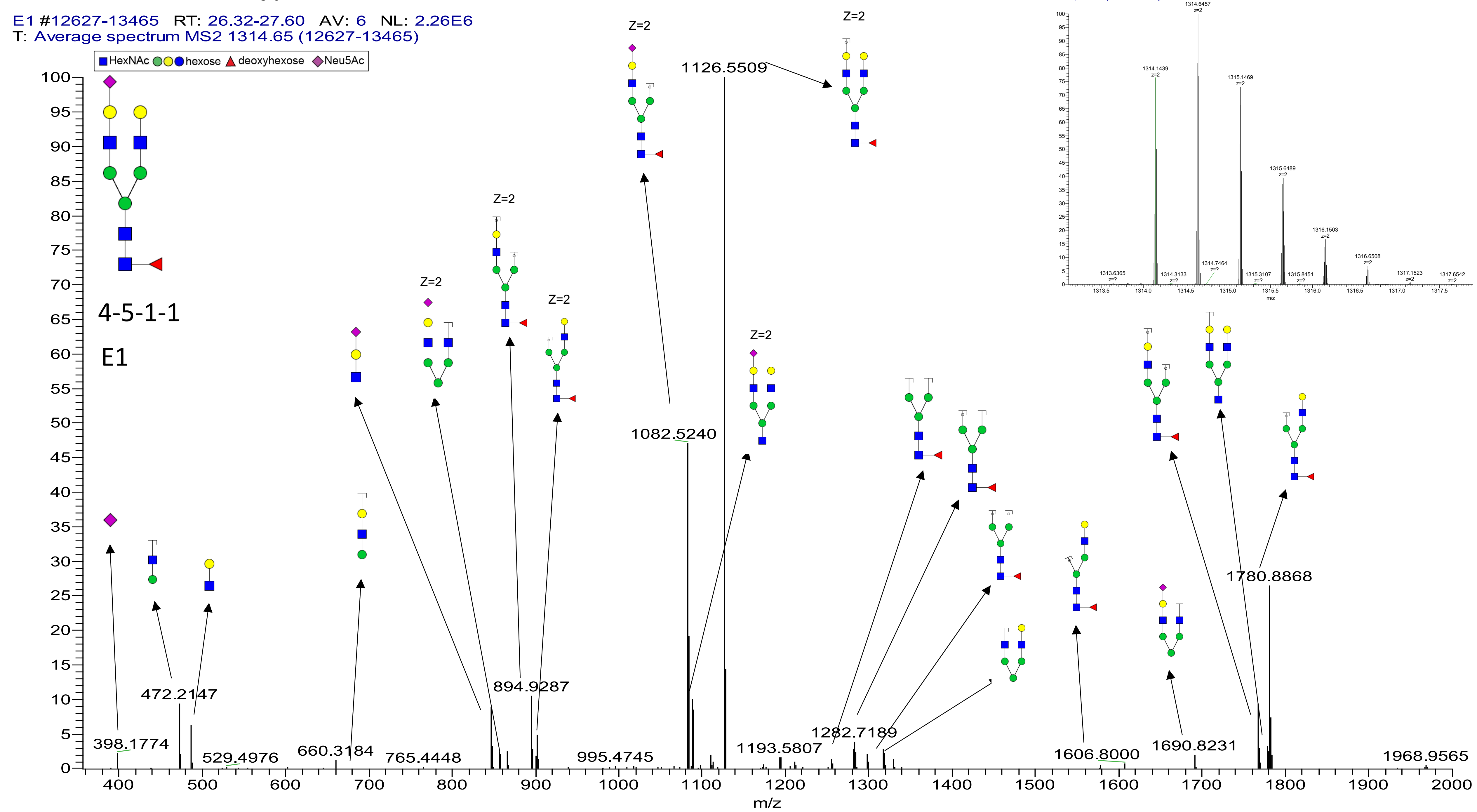

MS1 and MS2 for E1 clone N-glycoforms.

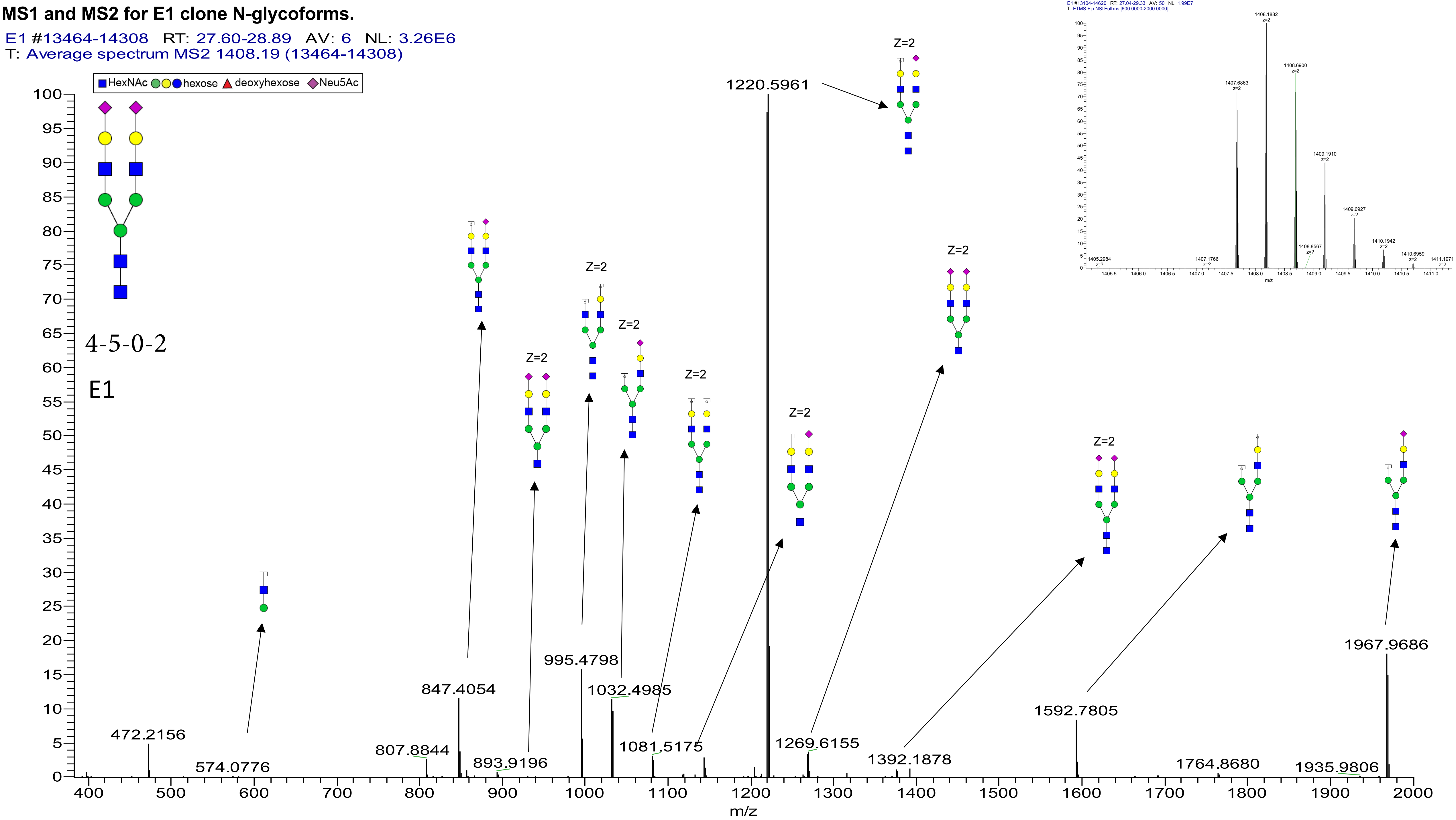

MS1 and MS2 for E1 clone N-glycoforms.

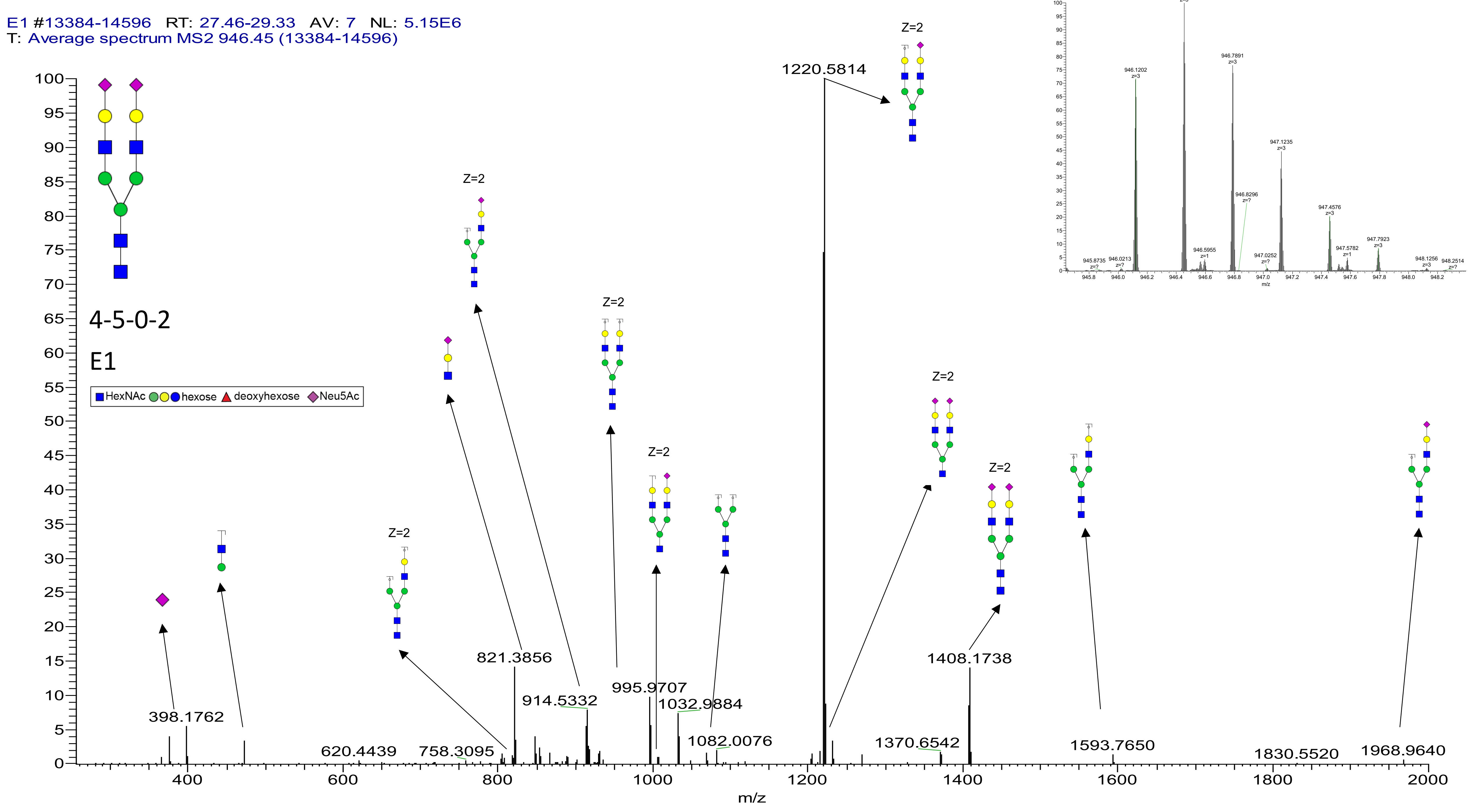

MS1 and MS2 for E1 clone N-glycoforms.

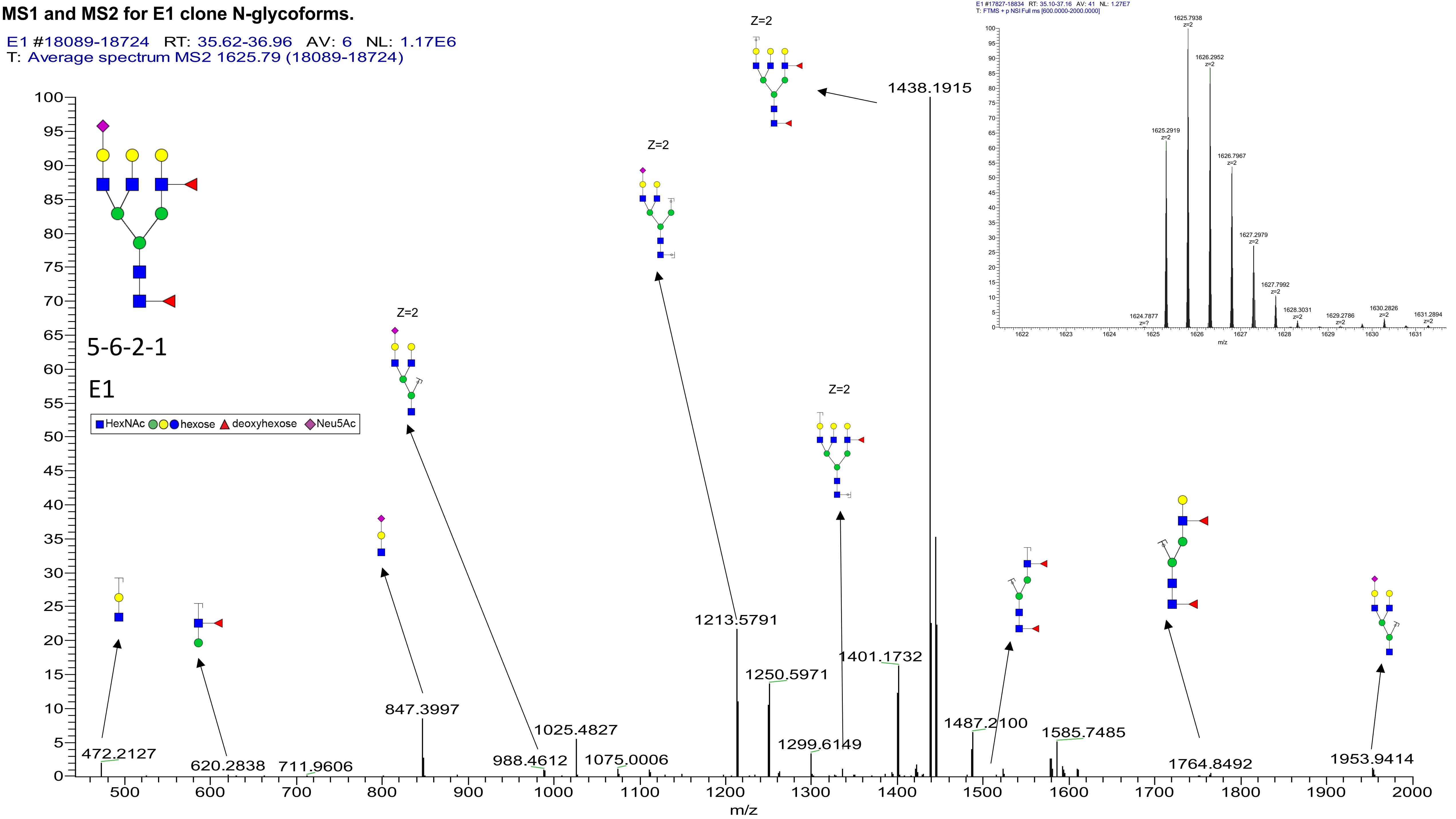

MS1 and MS2 for E1 clone N-glycoforms.

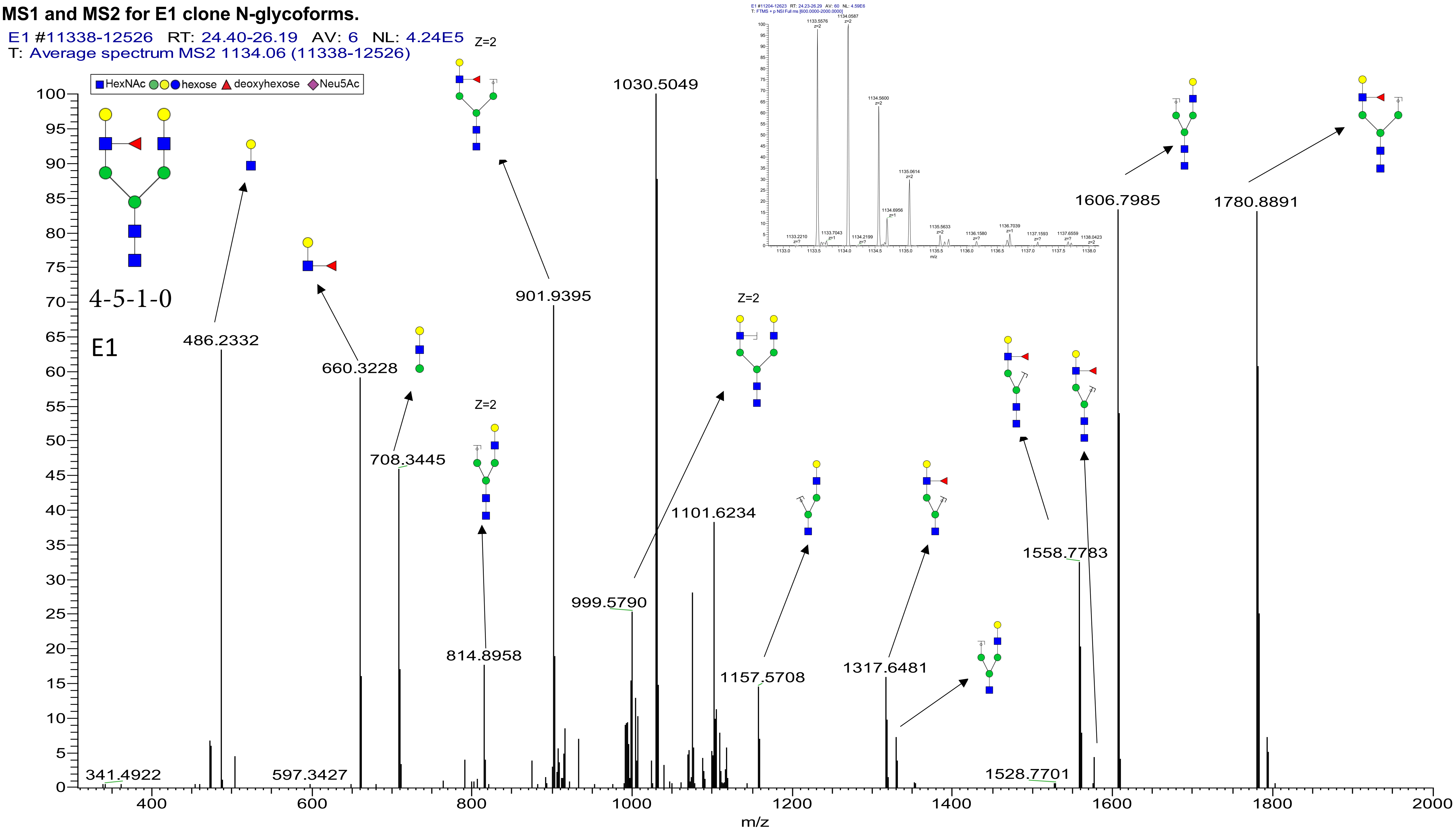

MS1 and MS2 for E1 clone N-glycoforms.

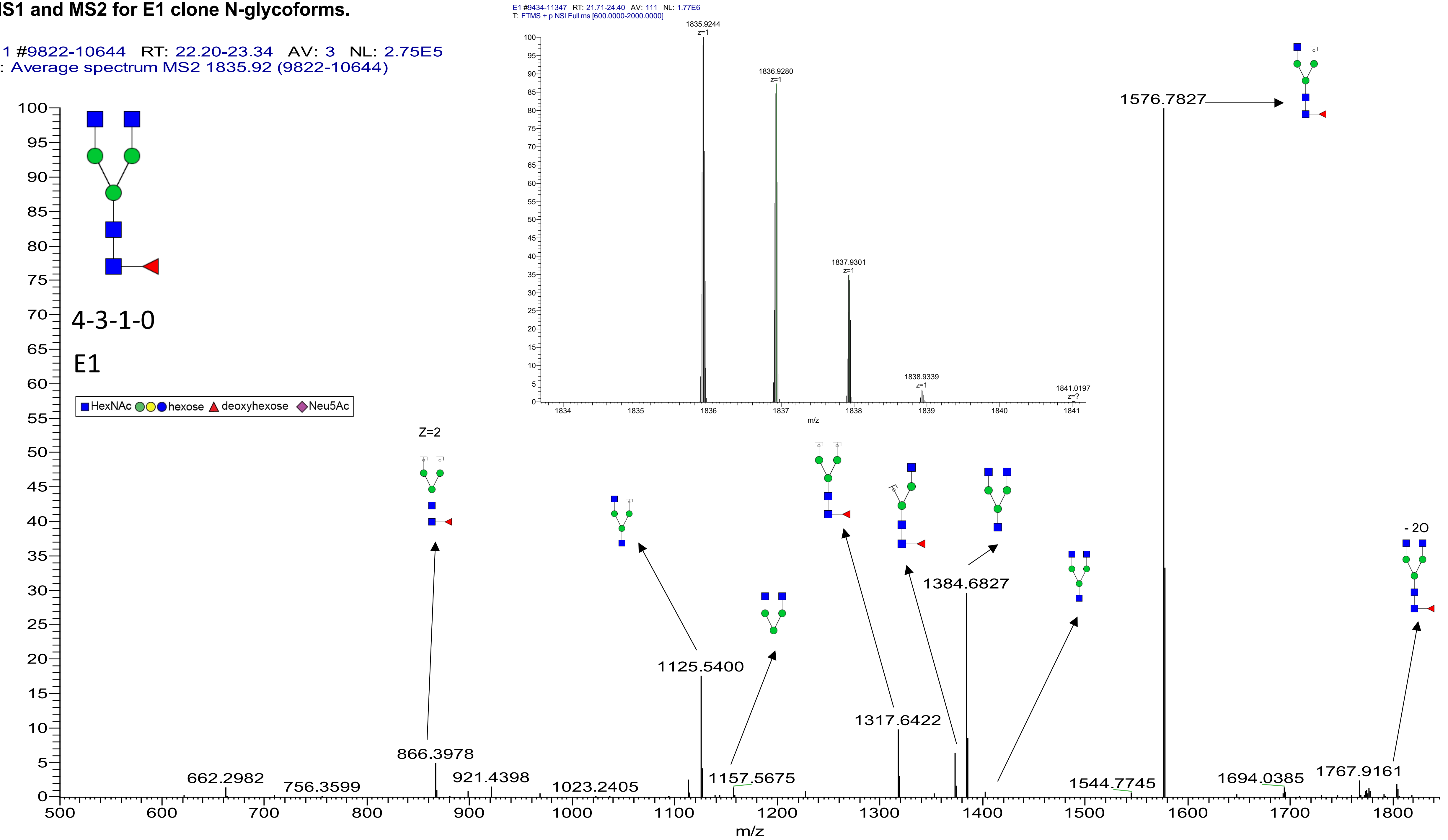

MS1 and MS2 for E1 clone N-glycoforms.

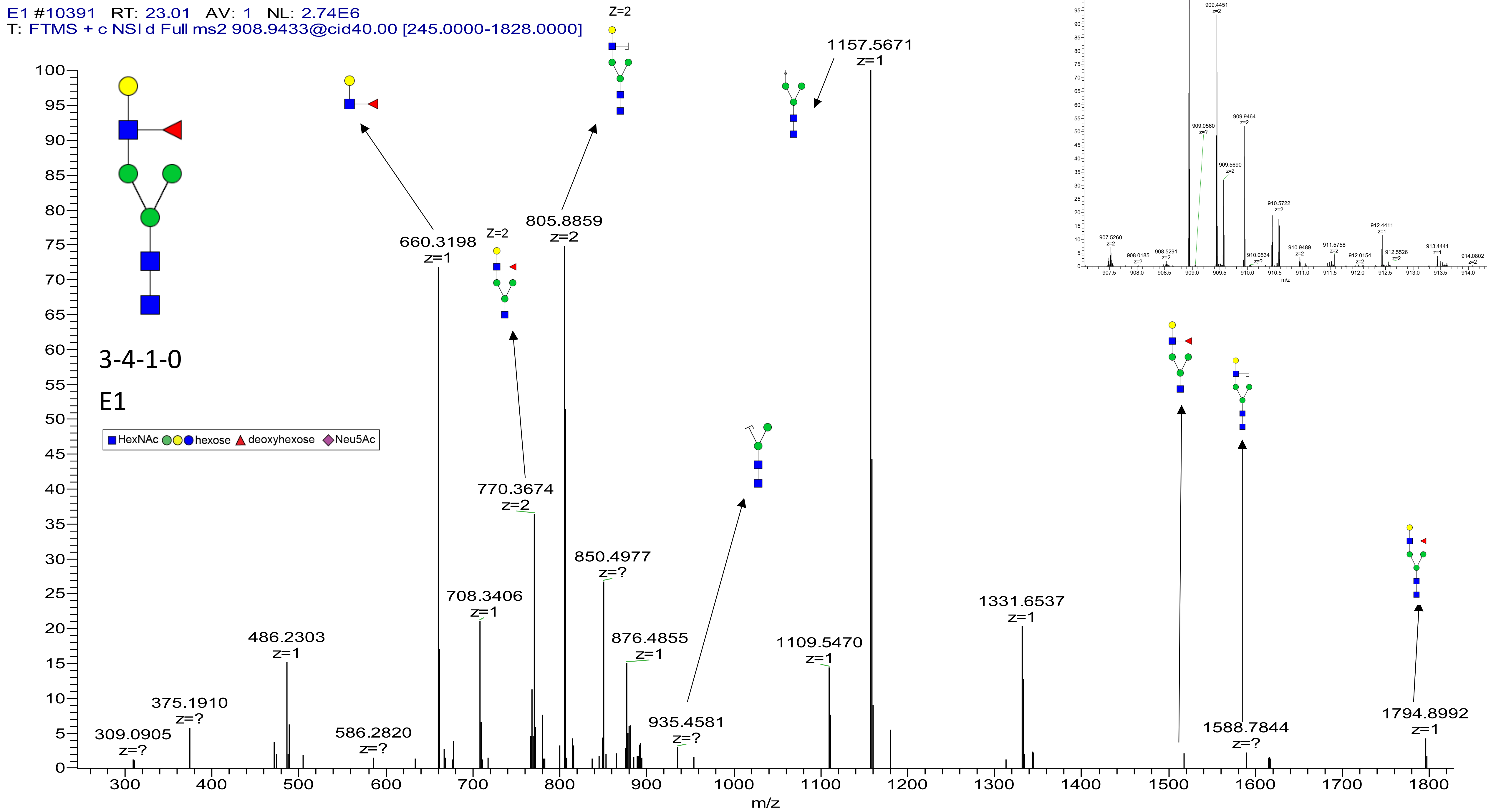

MS1 and MS2 for E1 clone N-glycoforms.

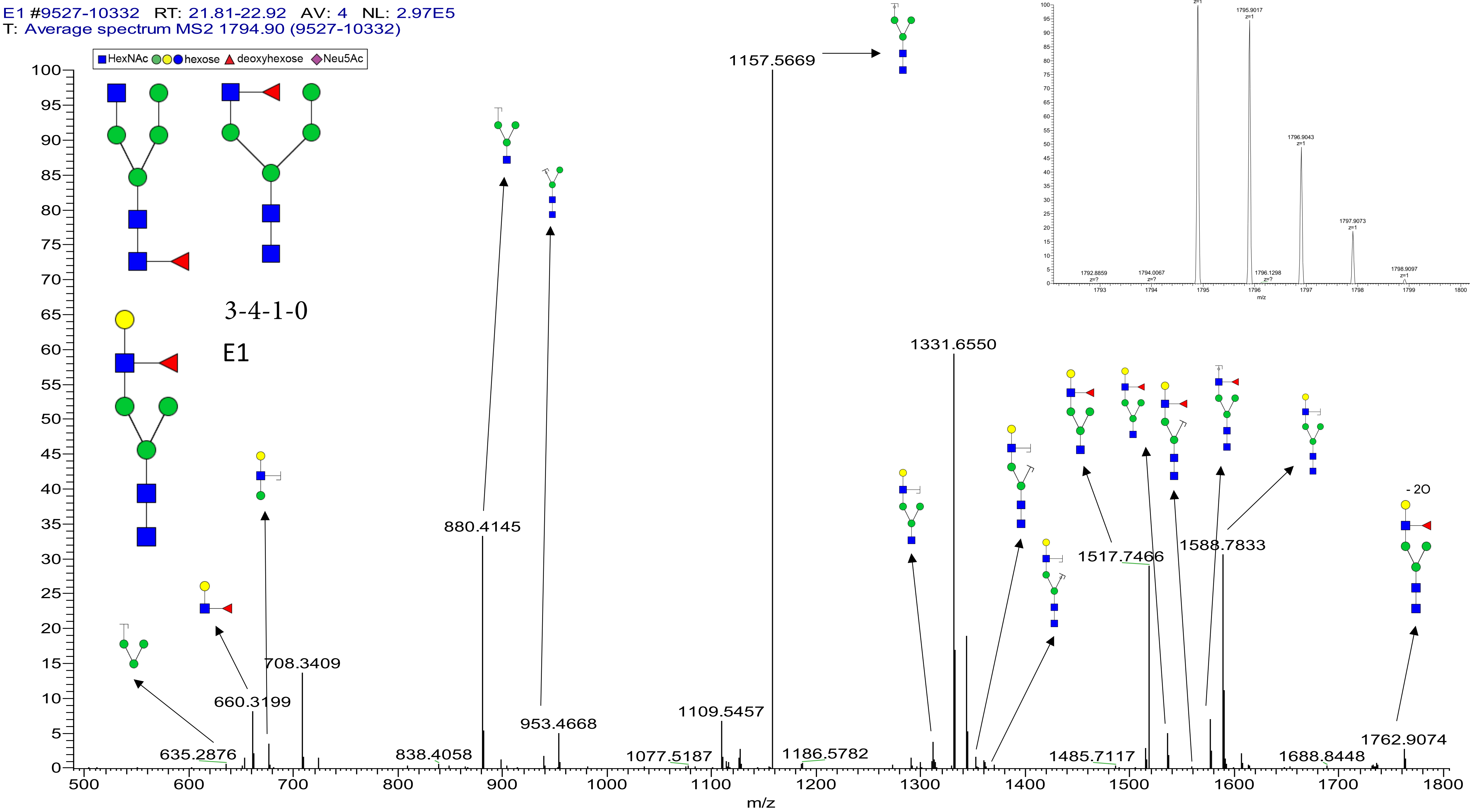

MS1 and MS2 for E1 clone N-glycoforms.

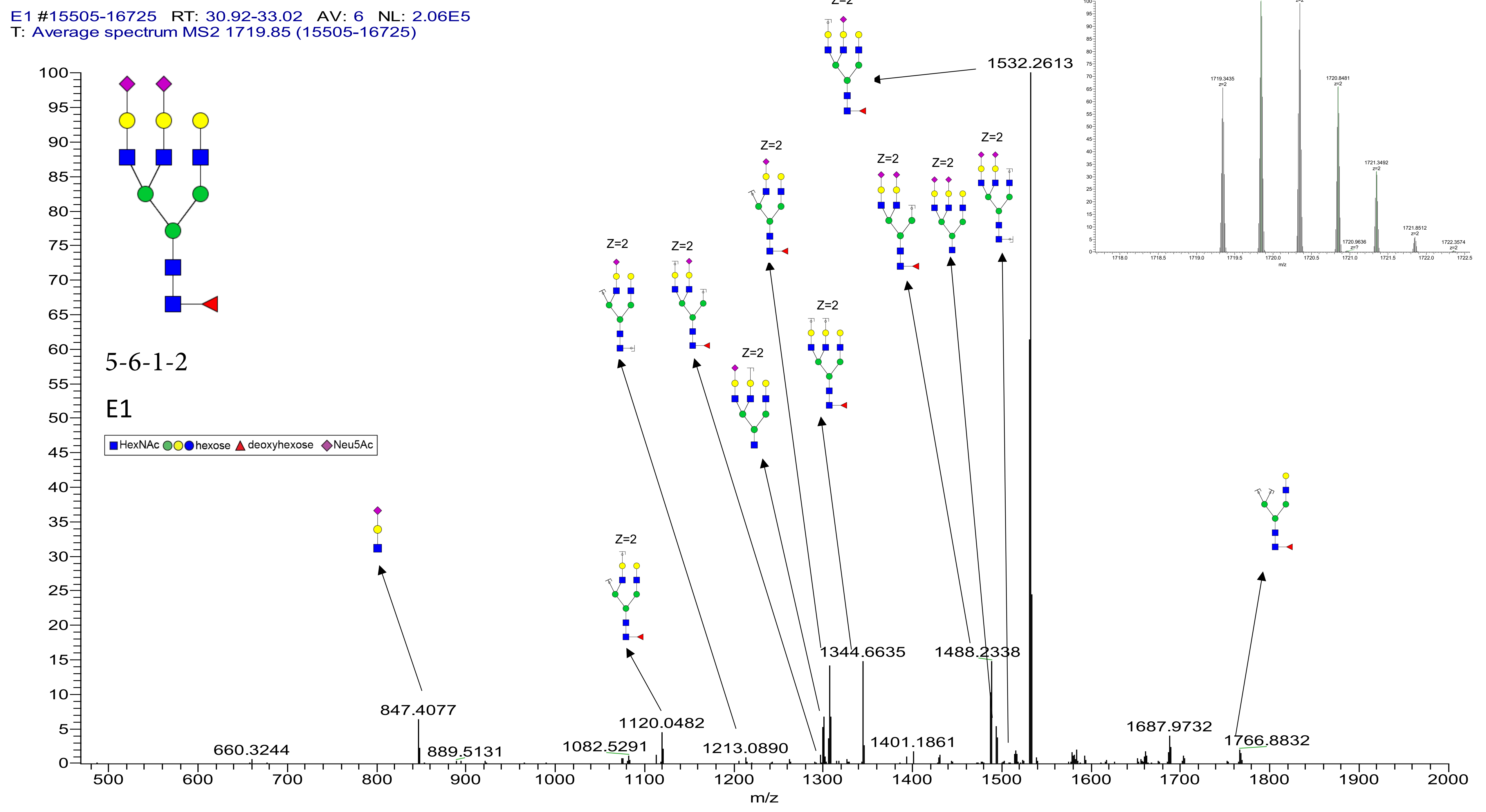

MS1 and MS2 for E1 clone N-glycoforms.

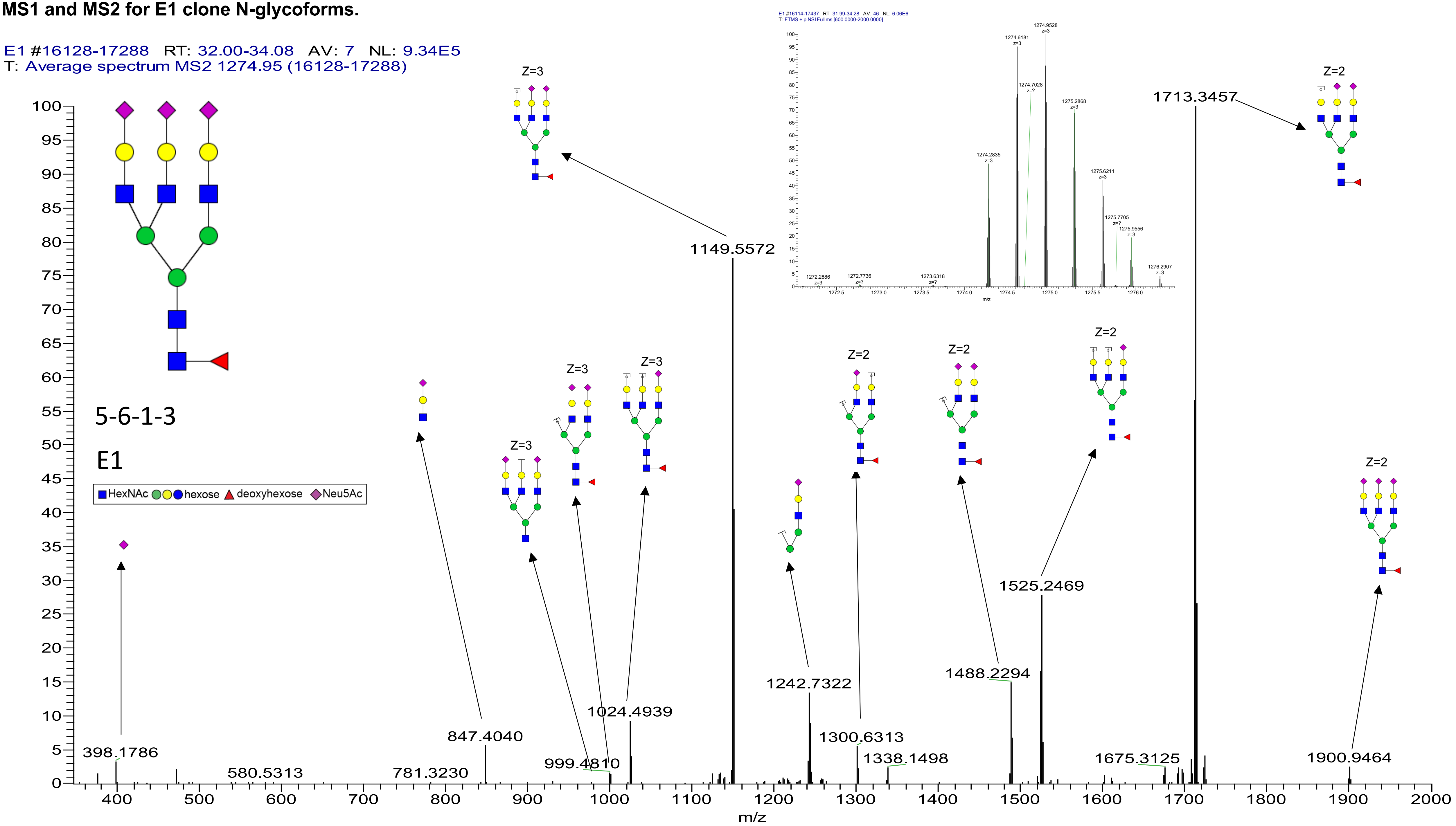

MS1 and MS2 for E1 clone N-glycoforms.

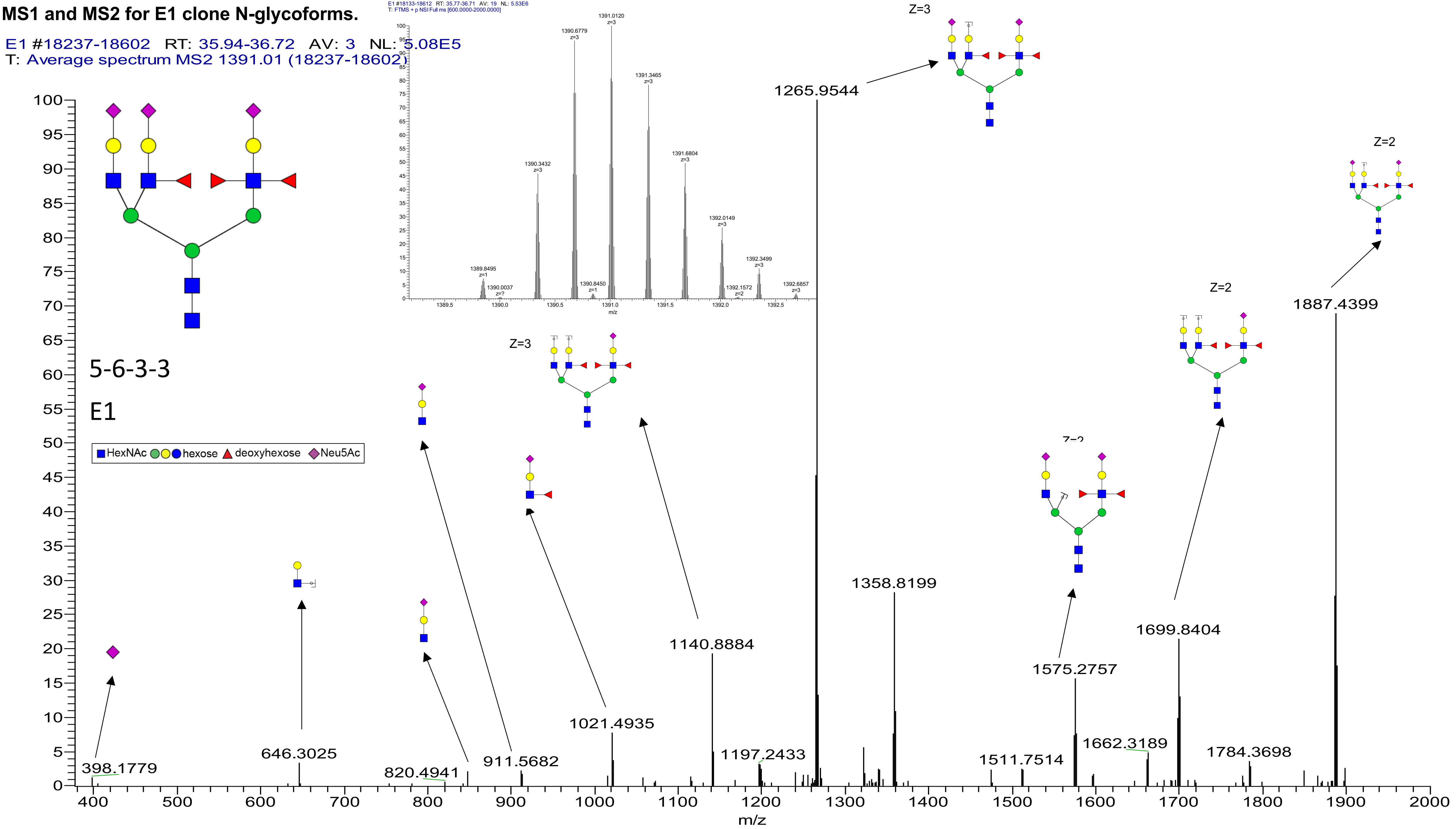

MS1 and MS2 for E1 clone N-glycoforms.

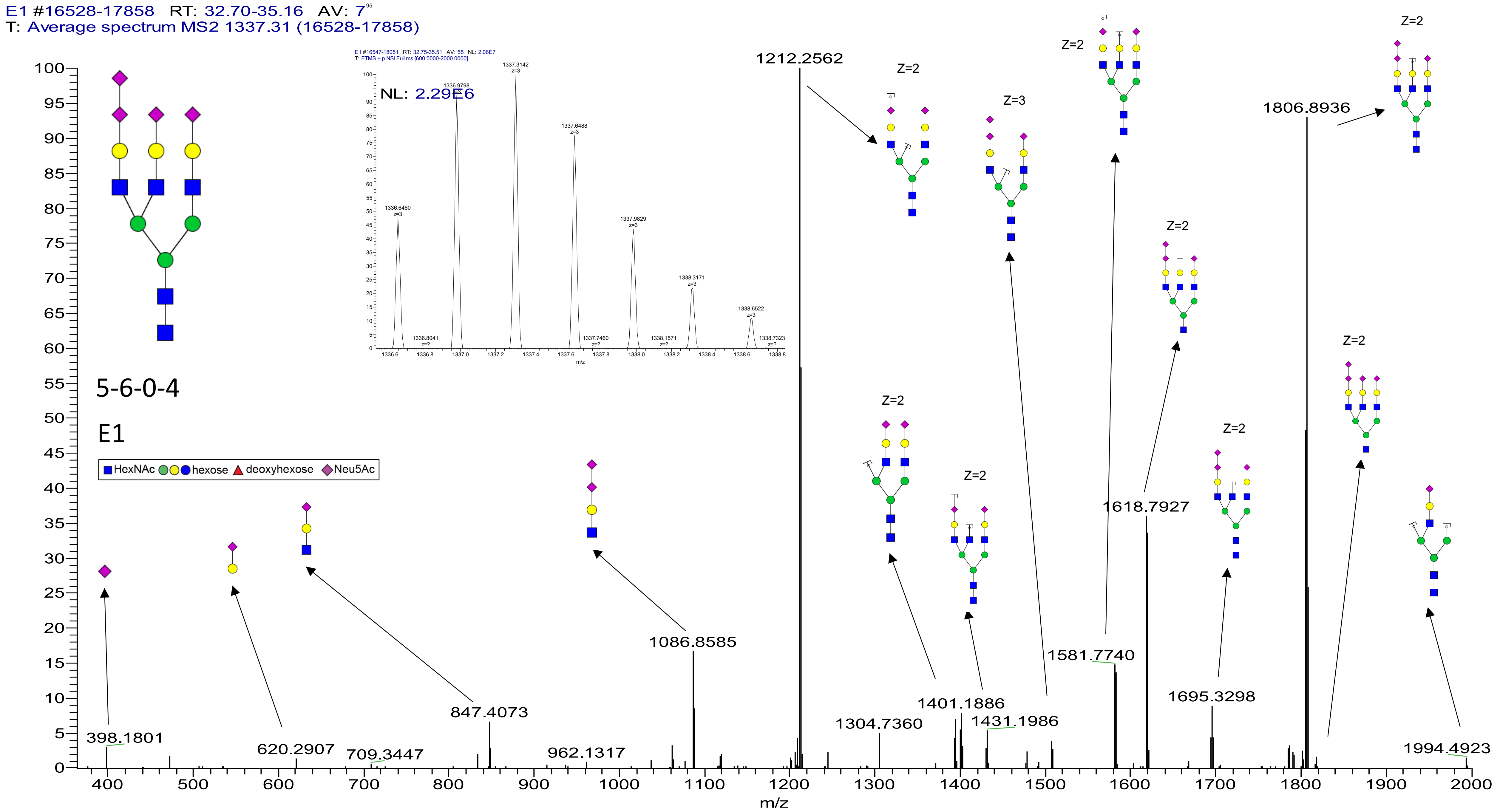

MS1 and MS2 for E1 clone N-glycoforms.

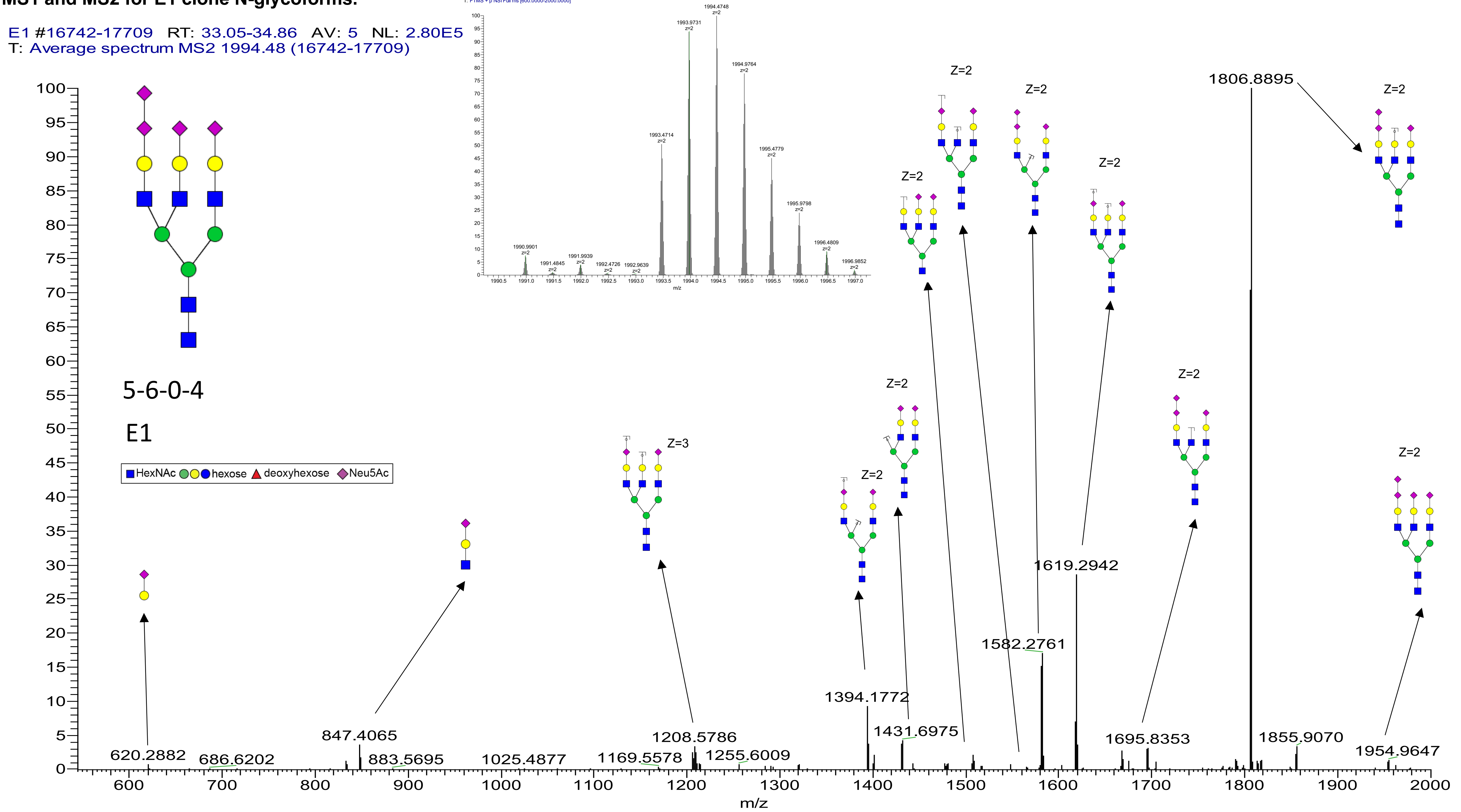

MS1 and MS2 for E1 clone N-glycoforms.

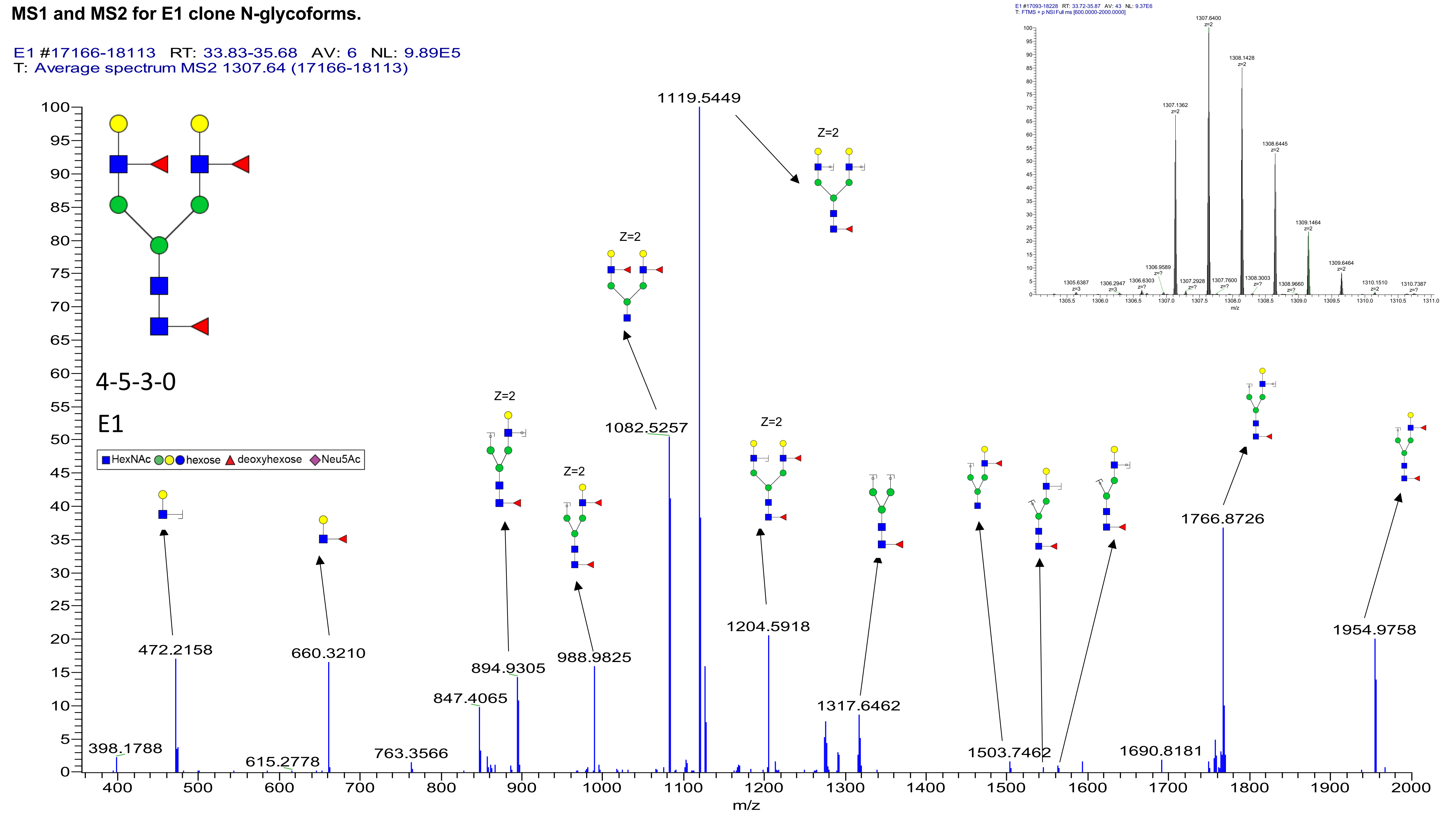

MS1 and MS2 for E1 clone N-glycoforms.

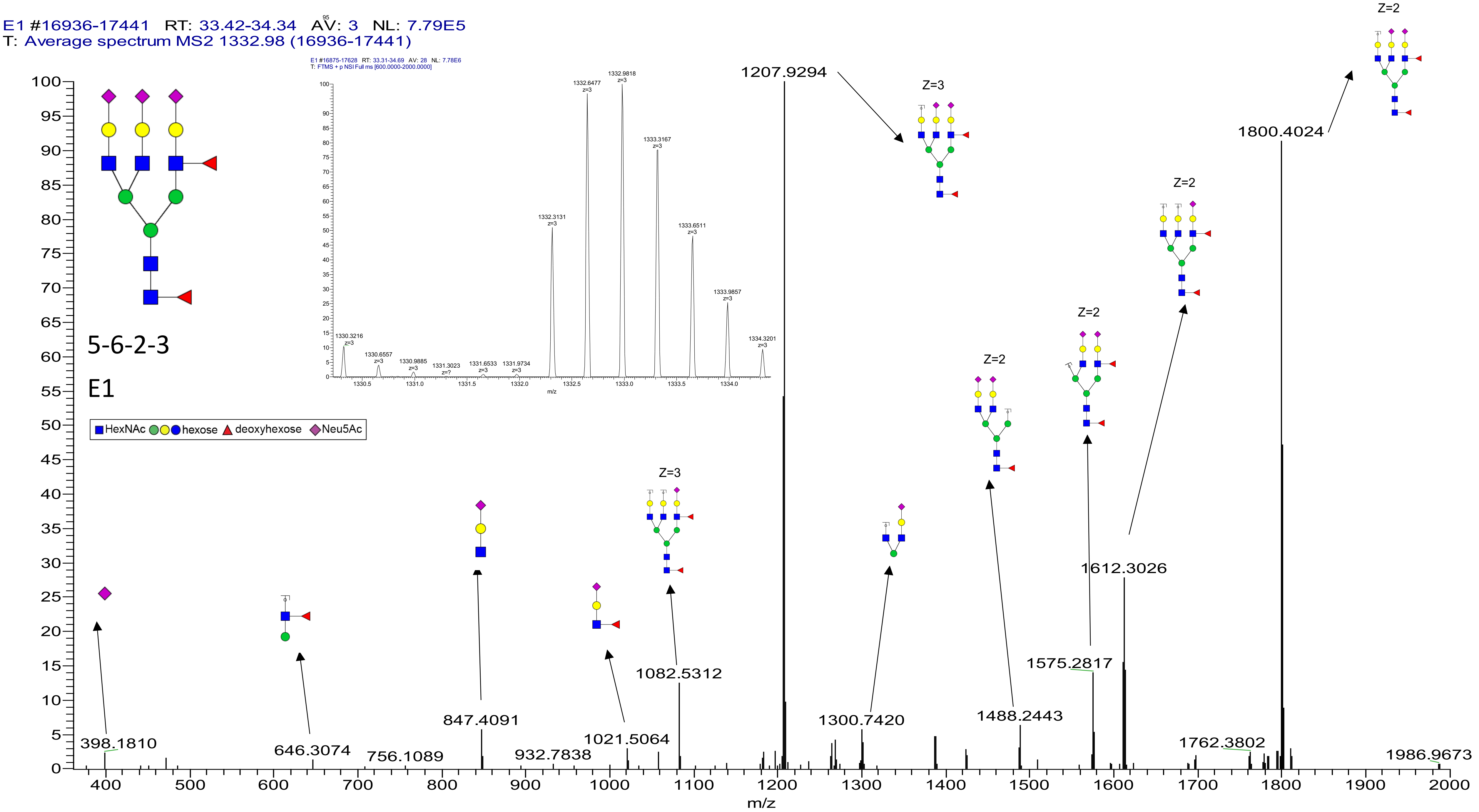

Supplement: Supplementary file 1 — Supplementary Information 1. [file 41598_2024_58541_MOESM1_ESM.pdf]
